# Spin-state crossover in photo-catalyzed nitrile dihydroboration *via* Mn-thiolate cooperation†

**DOI:** 10.1039/d2sc04339d

**Published:** 2022-10-20

**Authors:** Matthew R. Elsby, Changjin Oh, Mina Son, Scott Y. H. Kim, Mu-Hyun Baik, R. Tom Baker

**Affiliations:** Department of Chemistry and Biomolecular Sciences and Centre for Catalysis Research and Innovation, University of Ottawa Ottawa Ontario K1N 6N5 Canada rbaker@uottawa.ca; Department of Chemistry, Korea Advanced Institute of Science and Technology (KAIST) Daejeon 34141 Republic of Korea mbaik2805@kaist.ac.kr; Center for Catalytic Hydrocarbon Functionalizations, Institute for Basic Science (IBS) Daejeon 34141 Republic of Korea

## Abstract

The role of S-donors in ligand-assisted catalysis using first-row metals has not been broadly investigated. Herein is described a combined experimental and computational mechanistic study of the dihydroboration of nitriles with pinacolborane (HBpin) catalyzed by the Mn(i) complex, Mn(κ^3^-S^Me^NS)(CO)_3_, that features thioether, imine, and thiolate donors. Mechanistic studies revealed that catalysis requires the presence of UV light to enter and remain in the catalytic cycle and evidence is presented for loss of two CO ligands. Stoichiometric reactions showed that HBpin reduces the imine N=C of the ligand backbone in the absence of nitrile, forming an inactive off-cycle by-product. DFT calculations showed that the bifunctional thiolate donor, coordinative flexibility of the S^Me^NS ligand, and access to an open-shell intermediate are all crucuial to accessing low-energy intermediates during catalysis.

## Abstract 2

The role of a phosphine-free SNS-pincer ligand in metal–ligand cooperative hydroboration catalysis was investigated. The bifunctional thiolate donor and spin-state change to high-spin Mn are crucial to accessing low-energy activation barriers.

## Graphical abstract

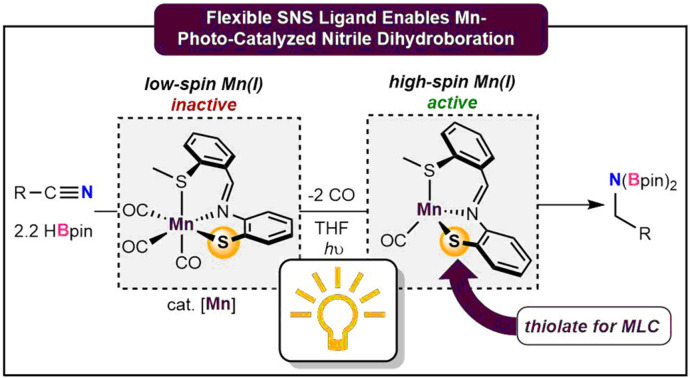

## Introduction

Homogeneous catalysts are important for carrying out highly selective synthesis of fine and commodity chemicals.^1^ Sustainable catalysis is a priority for green chemical processes,^2^ and the past decade has witnessed increased efforts towards the development of first-row metal catalysts to further this goal.^3^ The natural abundance^4^ and corresponding low-cost of the base metals areoften considered the main draws for their mainstream use. Nevertheless, these metals give access to diverse coordination geometries, odd–electron reaction pathways, and multiple spin states that could enable new catalytic pathways.^5^

**Fig. 1:**
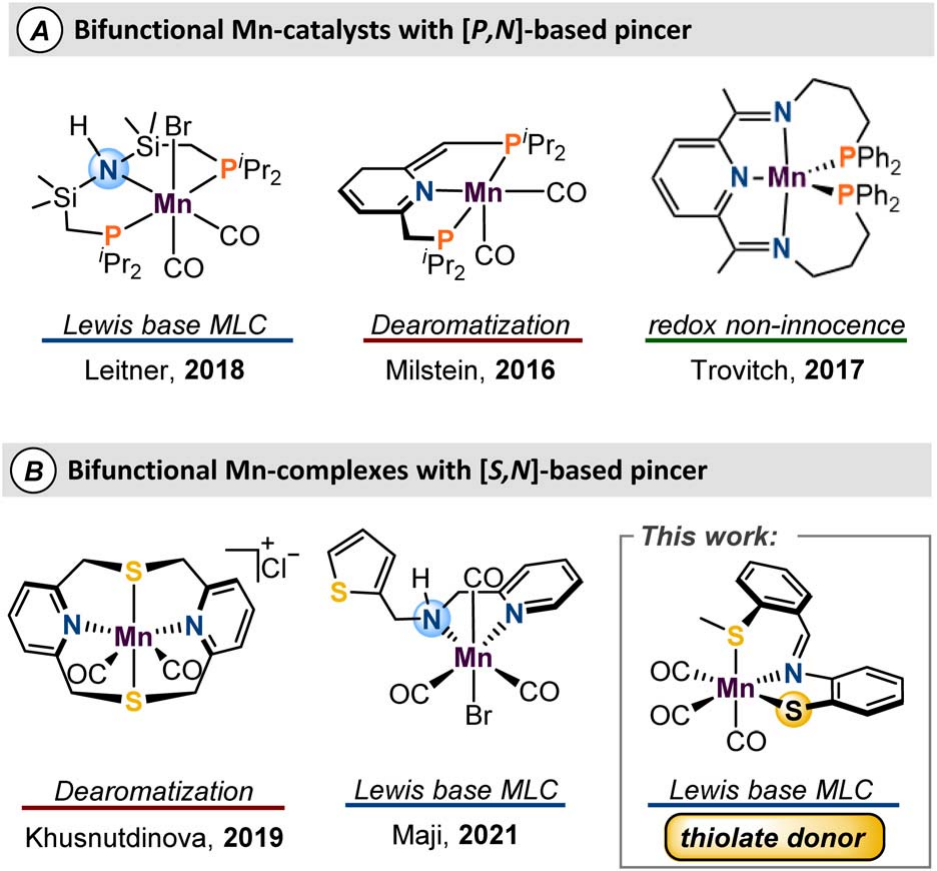
Notable manganese complexes featuring either P,N or S,N multidentate ligands.

Metal–ligand cooperativity (MLC)^6^ is a valuable strategy that provides unique reactivity when first-row metals are combined with bifunctional ligands.^7–10^ Amongst the base metals, iron catalysts are the most developed,^11^ while the interest in manganese catalysts has increased exponentially in recent years.^12^ Since Beller's seminal report of Mn-catalyzed hydrogenation of carbonyls and nitriles,^13^ development of manganese catalysts which use an MLC approach has experienced tremendous growth.^14–17^ A shared motif amongst many reports of MLC-type Mn catalysts is the use of phosphine-based bifunctional ligands which often serve to stabilize the Mn centre and promote a low-spin ground state (Fig. 1A). To push sustainable catalysis forward, there has been interest in the study and development of sulfur-based pincer ligands for their air stability, economy, and facile syntheses, relative to P-derived ligand sets. Despite recent progress (Figure 1B),^18^ use of ligand frameworks composed of biomimetic donors such as sulfur or oxygen is underdeveloped, and more work is needed to allow for optimal catalyst design.

We have been investigating base-metal catalysts that leverage MLC with simple SNS ligands featuring either an amido or thiolate Lewis base donor.^19^ In a recent study, divalent bis(amido) complex, Mn(S^Me^NS^Me^)_2_, was shown to hydroborate carbonyls through a novel inner-sphere, metal hydride-free reaction pathway.^20^ While this work helped to highlight alternative reaction pathways enabled by sulfur-based pincer ligands, the MLC donor featured was the common amido. Overarchingly, the expansion of bifunctional Lewis base donors beyond nitrogen is sparse.^21,22^

In this work we use a Mn(i) platform with a [S^Me^NS] ligand which features thioether, imine, and thiolate donors to examine the ability of the latter to participate in MLC. While the use of bifunctional thiolate donors for MLC has been reported with Ru^23^ and Ir,^24^ their well-defined use with first-row metal catalysts is limited to select examples with Fe and Ni.^25^ Herein we describe a Mn(i)-thiolate complex which catalyses nitrile dihydroboration upon UV irradiation through an MLC mechanism. Detailed experimental and computational mechanistic studies demonstrate the importance of ligand flexibility, borane activation by the thiolate donor, and electronic flexibility of the base metal to access low-energy intermediates.

## Results and discussion

### Catalyst synthesis and nitrile dihydroboration

**Fig. 2:**
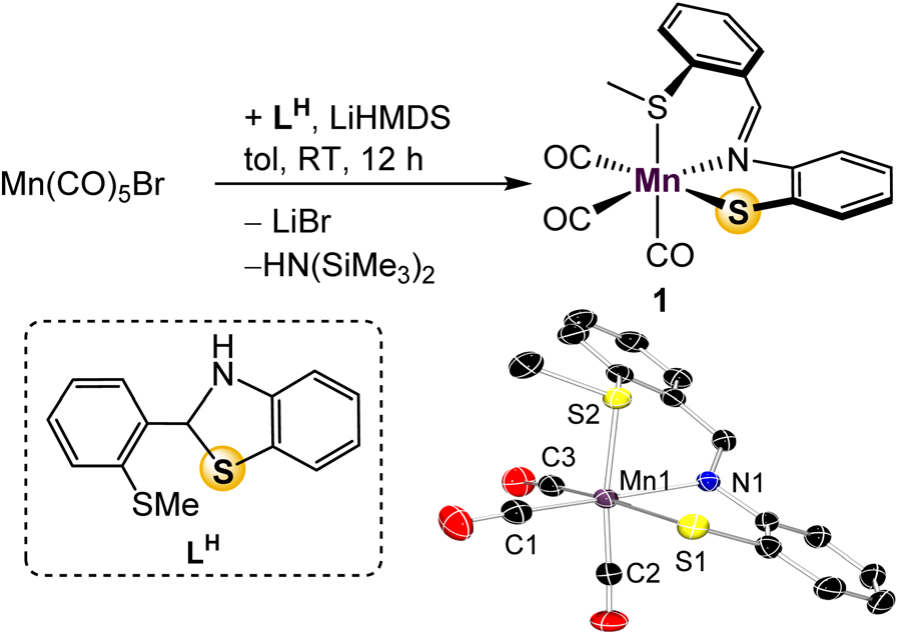
Synthesis and molecular depiction of 1. Thermal ellipsoids are set at 50% probability and hydrogen atoms are omitted for clarity. Selected bond lengths (Å): Mn–N 2.0587(16), Mn–S(1) 2.3898(6), Mn–S(2) 2.3592(6), Mn–C(1) 1.797(2), Mn–C(2) 1.802(2), Mn–C(3) 1.805(2).

In previous work with mono-ligated Fe-[SNS] complexes, it was found that electron-rich co-ligands encouraged C_aryl_–S cleavage of the thioether donor.^26^ To avoid this deleterious side reaction during the design of a mono-ligated Mn-[SNS] complex, we prepared the tricarbonyl complex. Treatment of Mn(CO)_5_Br with one equiv. of L^H^ and LiHMDS in toluene afforded Mn(S^Me^NS)(CO)_3_ (1) in 91% yield (Fig. 2). The thioether protons in the ^1^H NMR spectrum display a broadened singlet, likely due to thioether hemilability^19^ that allows for epimerization at sulfur. Observation of free CO in the ^13^C{^1^H} NMR spectra of solutions of 1 in CD_3_CN exposed to ambient light suggests lability of an ancillary carbonyl ligand as well,^27^ and UV-vis absorptions at 240 and 290 nm indicate that CO ligands may be labilized upon UV irradiation, potentially offering multiple opportunities to access unsaturated intermediates. Single crystal X-ray diffraction analysis revealed the expected pseudooctahedral geometry about Mn, where the [S^Me^NS] ligand is arranged in a facial manner. This is the first instance of our [SNS] ligand adopting this coordination pattern, suggesting potential for L to demonstrate coordinative fluxionality between *fac*- and *mer*-isomers that could also play a role in catalysis.

To examine the bifunctional activity of the thiolate donor in 1, its affinity to perform bifunctional B–H activation was tested. The stoichiometric reaction of 1 and HBpin in CD_3_CN resulted in no color change, and ^1^H NMR analysis revealed no reaction. Heating the reaction mixture to 80 °C also gave no reactivity and led to decomposition, as evidenced by observation of free L in solution. Alternatively, upon irradiation with UV-light, a color change was observed from dark burgundy to light yellow. The crude ^11^B NMR spectrum displayed a signal at 24 ppm, diagnostic of a B–N species, and further analysis of the ^1^H NMR spectrum revealed signals indicative of the dihydroboration of the acetonitrile-*d*_3_ solvent.

To investigate the potential of this system for nitrile dihydroboration, the reaction conditions were optimized with benzonitrile as the model substrate, and 2.2 equiv. of HBpin were used to ensure complete reduction (Table 1). Attempts to thermally facilitate the dihydroboration of benzonitrile were unsuccessful (entry 1); only upon UV-irradiation of the reaction mixture was product formation observed. While 1 is most soluble in acetonitrile, performing the dihydroboration of benzonitrile in this solvent results in the reduction of both benzo- and acetonitrile (entry 2). Reaction in either benzene-*d*_6_ or toluene-*d*_*8*_ did not yield satisfactory conversion (Entries 3 and 4). Reaction in THF afforded complete conversion to the dihydroboration product in reasonable time frames with as little as 1 mol% of 1 (entries 5 and 6). Further control reactions were performed to ensure the coordination of deprotonated [S^Me^NS] to Mn was crucial to a successful reaction (see ESI†). Evaluating the optimized reaction conditions makes it clear that the rate is influenced by the presence of polar, coordinating solvents, suggesting a solvent-stabilized intermediate may be involved in the reaction pathway.

**Table 1:** Optimization of reaction conditions^a^

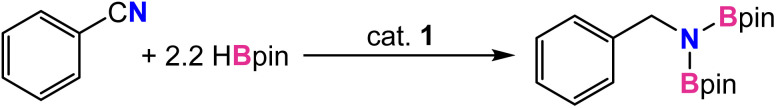						
Entry	mol%	Temp (°C)	*h*υ	Solvent	Time (h)	yield (%)
1	5	80	✗	MeCN	18	0
2	5	25	✓	MeCN	18	60
3	5	25	✓	C_6_D_6_	18	54
4	5	25	✓	Toluene	18	62
5	1	25	✓	THF	2	>99
6	1	25	✓	THF	6	>99

a Reaction conditions: nitrile (0.16 mmol), HBpin (0.34 mmol), 1 (see entry), THF (0.6 g) at room temperature with irradiation from UV-lamp.

Under the optimized conditions, additional substrates for the nitrile dihydroboration reaction were investigated (Table 2). Greater than 90% NMR yields were observed for most substrates and isolated products were easily obtained as crystalline solids by hexane extraction of the dried reaction mixture. Performing the reduction with 0.25 g of benzonitrile gave the *N*,*N*-diborylbenzylamine product in 87% isolated yield. Catalysis was not significantly hindered for benzonitrile substituted with electron-donating or -withdrawing groups (ii and iii) or for one heterocycle example (vi). A functional group in more sterically compromising positions hindered catalysis, with 3-chlorobenzene requiring a 2.5% catalyst loading to reach 90% yield (iv). For *p*-acetyl-benzonitrile, addition of 3.3 equiv. of HBpin afforded the trihydroborated product (v), efficiently reducing both the carbonyl and nitrile functionalities. Using 1 equiv. of HBpin with the same substrate afforded the ketone hydroboration product exclusively in 5 min without the need for irradiation. While it was not unexpected for 1 to be an active catalyst for carbonyl hydroboration, this reaction occurs thermally at room temperature, indicating it likely follows an alternative pathway from nitrile reduction. Indeed, a reaction mixture of catalytic 1, *p*-acetyl benzonitrile, and 3 equiv. of HBpin which was kept in the dark at room temperature afforded the ketone reduced product with 2 equiv. of unreacted HBpin, confirming that ketone reduction takes place thermally without the need for light. Finally, for aliphatic nitriles (vii–ix) it was found that acetonitrile, propionitrile, and isobutylnitrile were all successfully converted, albeit in slightly poorer yields than aromatic substrates. Aromatic substrates containing alkenes, esters, and amides were also examined. High catalyst loadings and poor conversions (30–50%) for alkenes, as well as no reactivity for esters and amides deterred further investigation (see ESI†).

**Table 2:** Catalytic nitrile dihydroboration using 1^a^

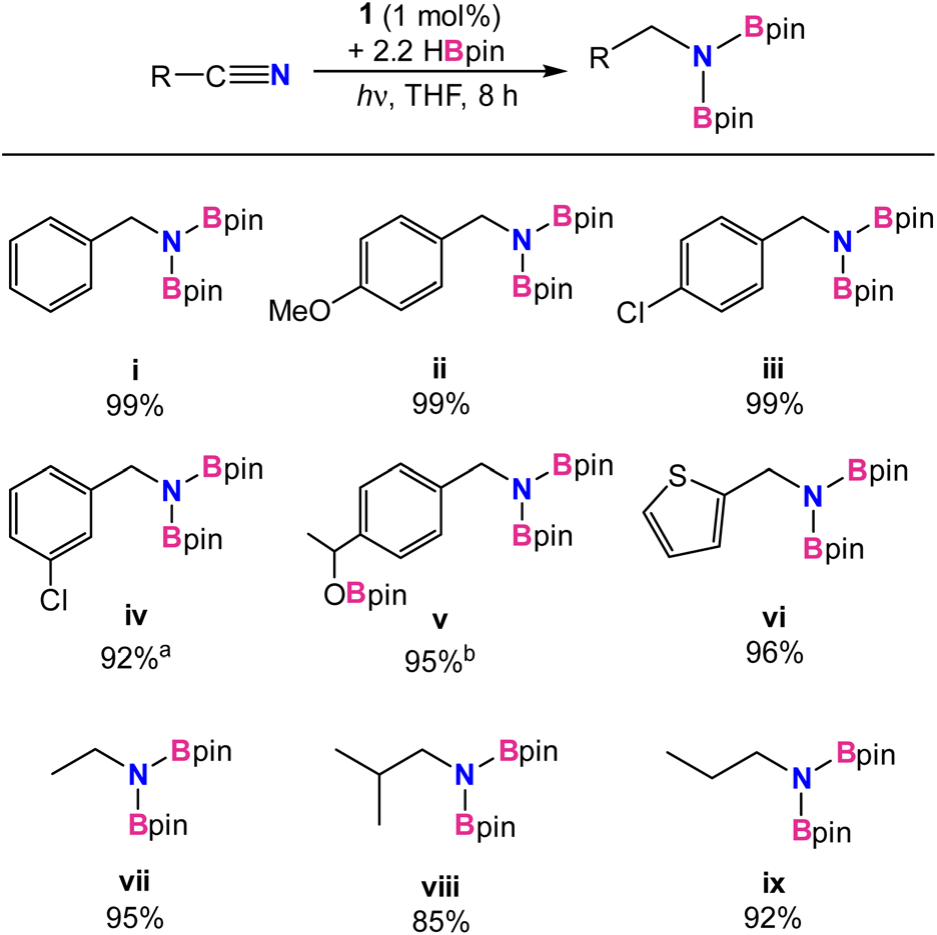

a Reaction conditions: nitrile (0.16 mmol), HBpin (0.34 mmol), 1 (1 mol%), THF (0.6 g) at room temperature with irradiation from UV-lamp for 2–8 h. ^a^ Conducted with 2.5% loading of 1. ^b^ Conducted with 3.3 equiv of HBpin. Yields were determined from ^1^H NMR integrals with respect to internal standard mesitylene after workup.

### Photoexcitation and carbonyl ligand dissociation

**Scheme 1:**
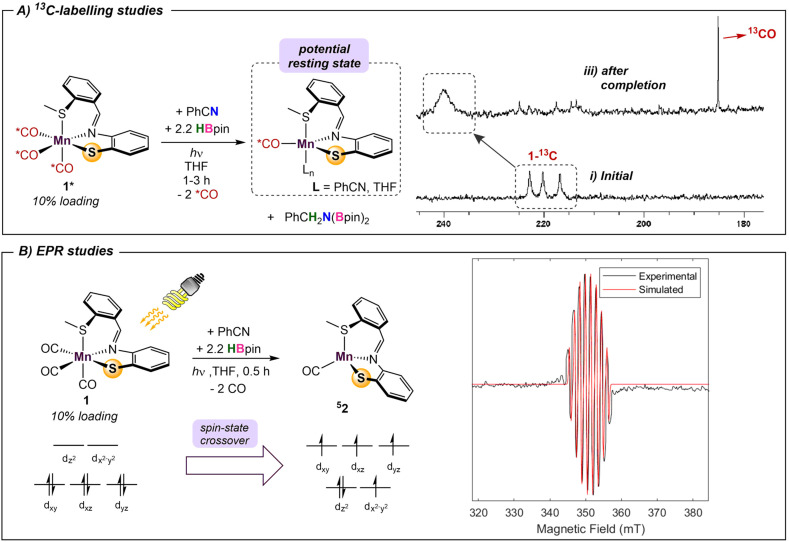
Spectroscopic and isotope-labelling experiments with 1.

The metal-pincer complex decorated with CO ligands is a widely used catalyst platform.^28^ Currently, many of these complexes use phosphine-based pincers and rely on elevated temperatures to kinetically access their respective reaction pathways. Despite the rich history of catalysis using photoinduced CO dissociation,^29^ use of UV irradiation in systems which use an MLC platform is rare. Photolysis may also provide potentially advantageous access to alternative reaction pathways involving open-shell intermediates. To further evaluate the capacity of UV-light in this system and understand the role of the bifunctional thiolate donor, a series of mechanistic experiments were performed. Initially, two parallel reaction mixtures of catalytic 1 with benzonitrile and 2.2 equiv of HBpin were irradiated until solution color change and product formation was observed. Subsequently, one of the samples was continually irradiated for a further 4 h, while the other was kept in the dark. Comparison of the two mixtures showed that the irradiated reaction went to completion, while catalysis in the “dark” mixture was halted, confirming that the persistent presence of light is needed to turn over the catalytic cycle. It is thus likely that dissociation of labile CO ligand(s) from 1 is the initial step, providing a vacant coordination site for substrate/solvent coordination. To gain insight into the number of CO ligands coordinated during catalysis, ^13^C-labeling studies were performed. The reaction of 2.2 equiv. of HBpin and 1 equiv. benzonitrile with a 10% loading of ^13^C-labelled 1* was monitored periodically by ^13^C{^1^H} NMR (Scheme 1A). The initial spectrum of 1* shows three distinct peaks in the M–CO region at 216, 220, and 222 ppm. Irradiation of the reaction mixture for 45 min initiated the expected color change and the onset of catalysis. The crude carbon NMR spectrum showed the disappearance of the three initial Mn–^13^CO signals and the emergence of a new very broad signal at 240 ppm (Fig. S7†). This single peak is indicative of either a single bound ^13^CO, or a highly symmetric intermediate with two coordinated ^13^CO's. Since the unsymmetrical nature of L likely precludes a high-symmetry environment, a species containing a single coordinated CO ligand is most likely. The broad signal at 240 ppm persisted throughout the reaction and remained detectable upon completion. Furthermore, observation of a new sharp signal at 184 ppm during catalysis was indicative of free ^13^CO in solution.

**Fig. 3:**
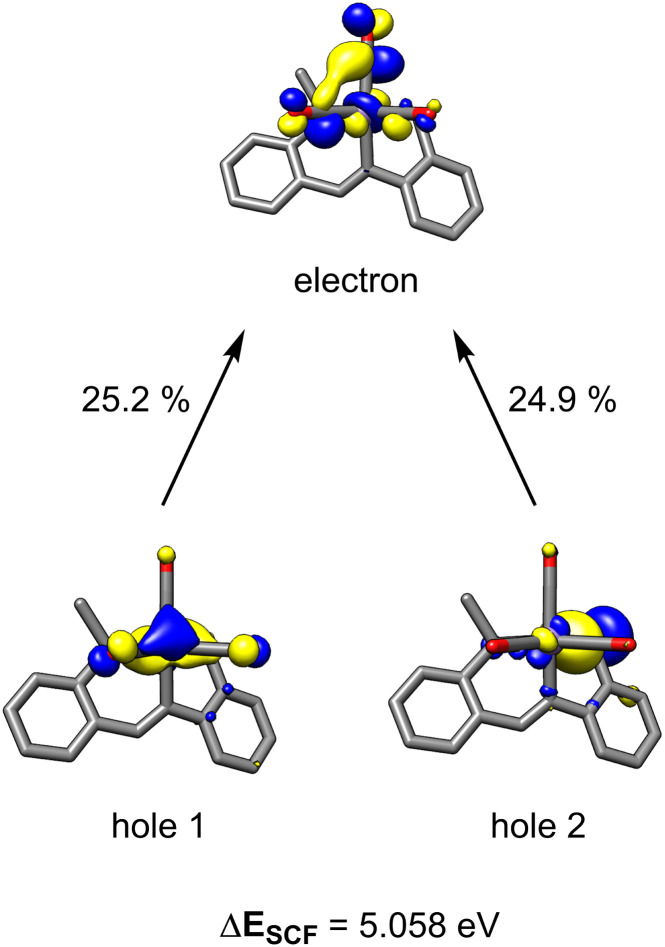
NTO pairs for the excitation responsible for CO dissociation. The top two contributing transitions are described. Other contributions are less than 15%.

Time-dependent density functional theory (TD-DFT) calculations were employed to probe the photoexcitation. Geometry optimization and TD-DFT were carried out at the B3LYP-D3/6-31G**, LACVP level of theory. In accordance with ^13^C-labeling studies, a metal-to-ligand charge transfer excitation into a metal-CO antibonding orbital was observed around 5.06 eV, supporting the dissociation of two CO ligands.^30^ Natural transition orbitals (NTOs) corresponding to the major transition from metal- and ligand-based orbitals to an orbital comprising an out-of-phase combination between metal and two COs are depicted in Fig. 3. With the dissociation of two strong-field carbonyl ligands, the d^6^ Mn centre may gain access to a high-spin configuration with a singly-bound CO, as suggested by the very broad ^13^C NMR resonance.^31^ Indeed, DFT investigation into intermediates containing a single carbonyl ligand consistently showed that a high-spin quintet ground state is energetically favored over the low-spin singlet or intermediate-spin triplet states (Fig. S30†). Electron paramagnetic resonance studies were performed to experimentally probe this result. The ambient temperature EPR spectrum of a solution of HBpin, benzonitrile, and catalytic 1 gave no signal. Upon irradiation of light for 45 min, the isotropic EPR spectrum yielded a multiplet at *g* = 1.99 which, after modeling, was attributed to hyperfine coupling to ^55^Mn (42.5 G) and ^14^N (42.1 G) (Scheme 1B). This emergence of a paramagnetic signal supports the initial DFT results indicating that the Mn centre preferentially adopts a high-spin state upon photoactivation.

### Catalytic cycle for nitrile dihydroboration

**Scheme 2:**
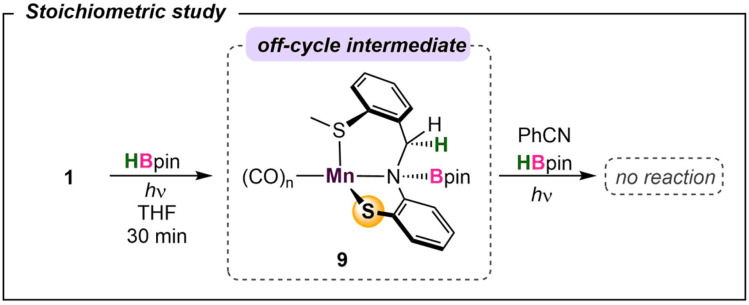
Off-pathway intermediate in the absence of nitrile.

**Fig. 4:**
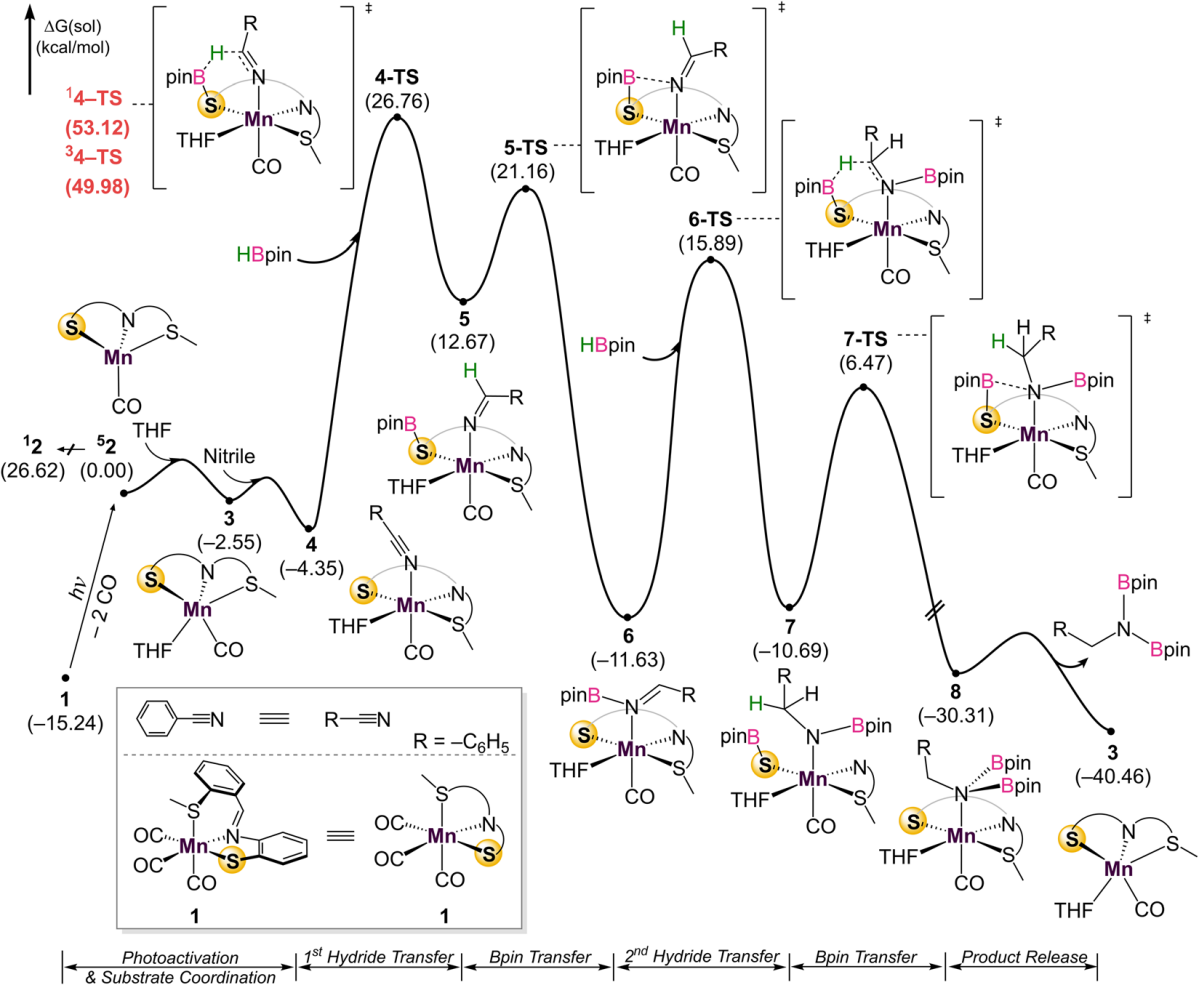
DFT-calculated energy profile for the dihydroboration of benzonitrile with catalyst 1.

To probe the reaction's initial steps associated with the dihydroboration, a stoichiometric amount of 1 treated with HBpin in THF-d_8_ in the absence of nitrile was irradiated, giving a color change from dark yellow to red.^32^ The resultant ^1^H NMR spectrum showed the disappearance of original peaks associated with 1 and the emergence of a new species (Fig. S25†). Specifically, the disappearance of the imine C–H resonance at *δ* 8.81 paired with the growth of a new singlet at *δ* 4.85 was diagnostic for borylation of the C–N imine unit forming 9 (Scheme 2). While a small resonance was observed in the hydride region (*δ* −8.59), it was not of sufficient intensity to be considered as a viable reaction intermediate, similar to previous work by Leitner.^15^ Given the literature precedence for imine hydroboration^33^ in addition to a previous report of the activity of a ligand backbone for H_2_ activation,^34^ the observed hydroboration of the imine backbone in the S^Me^NS ligand is not unexpected. Additionally, the hydroborated ligand was previously isolated and characterized in our lab,^35^ and its presence in this reaction mixture was confirmed by comparison of ^1^H and ^11^B NMR data. It is of note that this intermediate is preferentially formed over Mn–H derived from traditional bifunctional B–H activation by 41.2 kcal mol^−1^ (Fig. S29†). To further investigate 9 as a catalytic intermediate, 1 equiv. of benzonitrile and an additional equiv. of HBpin were added to the reaction mixture. Curiously, no product conversion was observed upon heating or irradiation. The lack of reactivity of 9 suggests it is an off-cycle product or an unproductive species that does not form under catalytic conditions. This is supported by DFT calculations showing that formation of 9 is exergonic by −17.1 kcal mol^−1^, confirming it to be a thermodynamic sink. In addition, while direct insertion of HBpin to the ligand imine moiety in the absence of nitrile is feasible with a barrier of 31.4 kcal mol^−1^ (Fig. S36† in ESI), the proposed pathway traversing 4-TS in Fig. 4 is energetically favored in the presence of nitrile. This suggests that the initial presence of both HBpin and nitrile are required in solution to enter the productive catalytic cycle. Finally, re-examining the crude catalysis solution by ^1^H NMR after product workup showed persistence of the imine C–H resonance, indicating that ligand imine hydroboration is not favored under catalytic conditions.

Given the challenges associated with monitoring the reaction in real-time, DFT calculations on the overall mechanism were carried out at the B3LYP-D3/cc-pVTZ(-f)/LACV3P//B3LYP-D3/6-31G**/LACVP level of theory^36^ with a self-consistent reaction field (*ε* = 7.6 for THF) approach to account for solvation (Fig. 4).^37^ Initial photoactivation and carbonyl dissociation forms the monocarbonyl species 2. With the loss of two carbonyls, which act as strong-field ligands, a high-spin configuration is favored. Thus, the high-spin quintet intermediate ^5^2 is preferred over the low-spin species ^1^2 by 26.6 kcal mol^−1^. While the intermediate-spin triplet ^3^2, located 4.4 kcal mol^−1^ higher than ^1^2, is relatively close to the ground quintet state, the preference for high-spin species is retained throughout the catalytic cycle. A detailed comparison of different spin states is given in the ESI (Fig. S30)†.

Coordination of the solvent THF to form 3 is followed by nitrile coordination to the metal centre, leading to stable intermediate 4. The calculations confirm experimental results that excluded significant participation of Mn–H, with HBpin instead coordinatingto the bifunctional thiolate sulfurand allowing for direct hydride transfer to the coordinated nitrile through 4-TS to form 5. An alternate pathway featuring the direct coordination of HBpin *in lieu* of solvent to form a Mn–S–B–O metallacycle was also considered, inspired by previous work (Fig. S31†).^20^ This pathway has a barrier of 41.5 kcal mol^−1^ due to the penalty of forming a sterically hindered 4-membered metallacycle, indicating that a solvent molecule must be incorporated. Hemilability of the thioether group was also interrogated (Fig. S32†). As the thioether arm dissociates, the phenyl linker interacts with a THF molecule. Two transition states featuring the simple cleavage of thioether and *η*^2^-coordination of imine were captured and the corresponding barriers are 41.5 and 38.1 kcal mol^−1^, respectively. Notably higher barriers with the labile thioether group suggest that the SNS ligand likely remains tridentate during reaction. This first hydride transfer through 4-TS represents the turnover limiting step with a 31.1 kcal mol^−1^ barrier, which looks too high at first sight for a room–temperature reaction. Extensive efforts did not indicate any technical problems and no alternative transition state with a lower energy could be located (See ESI† for details). Taking into account that the reaction does not proceed without continuous UV irradiation, we reason that the Mn-complex obtains additional energy from the Mn–CO bond cleavage process, as the excitation energy is dissipated locally through heat and molecular vibrations. Alternatively, if rebinding of the CO does not occur, the Mn-catalyst may absorb light and undergo non-radiative decay. In either case, the local vibrational temperature of the Mn-catalyst will be much higher than what is expected for an undisturbed ground state system. In other words, UV irradiation not only cleaves the Mn–CO bond, but also provides additional energy during hydroboration to overcome the reaction barrier.

**Fig. 5:**
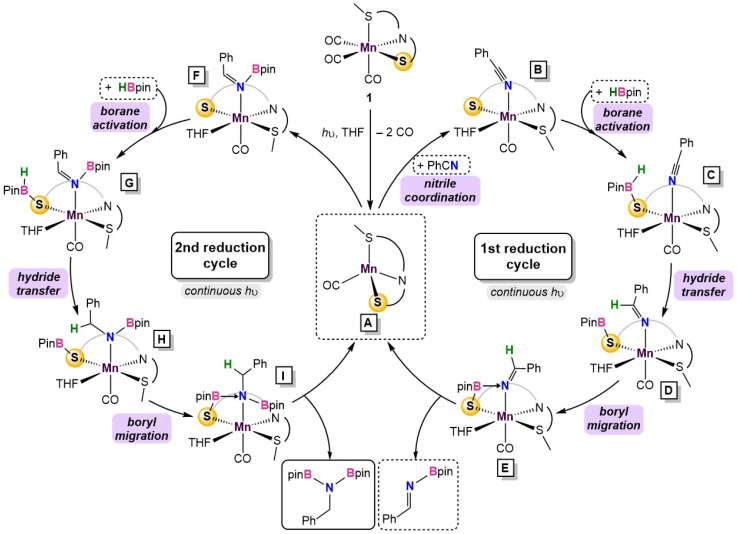
Summary of the catalytic cycle for the photodissociation and two borylation steps involved in the reaction.

Substitution of THF with a weakly coordinating solvent, benzene, gives a greater hydride transfer barrier of 34.0 kcal mol^−1^ which coincides well with the observed conversions in Table 1 (Fig. S34†). Closer inspection of the hydride transfer in 4-TS shows that a transient metallo-imine species is generated, with the CO ligand *trans* to the anionic iminato moiety to electronically stabilize the transition state as in structure 5. For the *trans* effect to successfully stabilize the intermediate, the SNS ligand must be arranged in a *mer*-configuration. Indeed, a comparison between *mer*- and *fac*-conformations shows the former is favored by 7.4 kcal mol^−1^, indicating that the coordinative flexibility of the SNS ligand is crucial for rearrangement to the lowest energy conformer (Fig. S35†). The resulting intermediate 5 then undergoes sequential Bpin transfer to generate a monoborylimine in 6 and complete the first reduction cycle. Evidence for the monoborylimine product was never observed experimentally, and it was found that steric hindrance is responsible for the selective hydride transfer to the monoborylimine over the imine of the ligand framework. In the absence of THF, the ligand imine is preferentially reduced (Fig. S36 and S37† in ESI), supporting the kinetic preference for reduction of the monoboryl-imine. Finally, undergoing a similar mechanism involving sequential hydride and Bpin transfer through 7 and 7-TS, the second reduction cycle furnishes intermediate 8 which releases product diborylamine, as depicted in the proposed full mechanism for both reduction cycles (Fig. 5).

## Conclusions

Catalytic dihydroboration of nitriles is an effective strategy to access synthetically valuable *N*,*N*,-diborylamine precursors^38^ and has been previously reported in systems using Mo,^39^ Th,^40^ Zn,^41^ Mg,^42^ and Ru^43^ catalysts with catalyst loadings between 1 and 10% and temperatures between 22 and 80 °C. Previous reports with first-row transition metal complexes are limited to Ni,^44^ Co^45^ and a single Mn example^46^ which required elevated reaction temperatures.^46^ In this work, a Mn(i) complex featuring an SNS-thiolate ligand was prepared to study the bifunctional activity of less studied thiolate donors. While Mn(κ^3^-S^Me^NS)(CO)_3_ complex (1) was an active catalyst for carbonyl hydroboration at room temperature, reduction of nitrile substrates required continuous photolysis to access additional coordination sites on the Mn centre. ^13^C-labeling studies revealed a Mn-species likely featuring one coordinated CO ligand, in good agreement with TD-DFT calculations. EPR studies combined with DFT calculations indicated that the Mn-centre undergoes a spin state change to preferentially adopt the high-spin configuration during catalysis. This feature is a benefit of first-row metals,^5^ whereby embracing the electronic flexibility of the metal allows for access to lower energy intermediates. Experimental mechanistic studies and calculations suggest the catalytically active species incorporating a single CO ligand is formed under UV light, and upon traversing two reduction cycles, diborylamine is released as the product (Fig. 5). The bifunctional S^*Me*^NS ligand is crucial to the success of the reaction in three aspects: (i) the weak field donors comprising the SNS ligand enable Mn to access a low-energy, high-spin intermediate, (ii) the thiolate donor allows for an MLC pathway for borane activation and Bpin transfer (5 and 5-TS in Fig. 5), and (iii) the coordinative flexibility of the S^Me^NS ligand enables facile rearrangement between *fac*- and *mer*-isomers which is integral to stabilization of the turnover-limiting hydride transfer step (4-TS in Fig. 4). The presence of coordinating solvent THF *in lieu* of non-coordinating solvent was also determined to be crucial for access to the sterically relaxed transition state, mitigating the need to form a sterically disfavored metallacycle intermediate.

The use of synthetically accessible ligands with biomimetic N-, O- and S-donors, in combination with first-row metals, is vital to meet the growing demand for more sustainable catalyst options. Although photolysis for CO loss is a well-established paradigm, its necessity in this work to access high-spin reaction intermediates highlights how the unique electronic flexibility of the base-metals can decrease activation barriers.^47^ Whereas our previously reported divalent Mn catalyst was shown to operate *via* an amido donor,^20^ this work uses a thiolate donor coordinated to Mn(i). This shows that this alternate inner-sphere, hydride-free reaction pathway does not discriminate on the basis of the bifunctional donor or even the metal d-electron count; it is instead likely a consequence of the weaker donors on the S^Me^NS ligand *vs.* P-containing pincer ligands. The combination of a first-row metal and a thiolate donor in MLC catalysis remains rare,^25^ and this is the first instance of a Mn catalyst using a bifunctional thiolate. As more phosphine-free ligand variants emerge,^48^ it will be critical to gain understanding of their associated reactivity patterns to enable optimal designs of sustainable and high-performing first-row metal catalysts.

## Data availability

All data are included in the ESI.†

## Author contributions

MRE and CO contributed equally to this work. MRE performed all experimental work associated with synthesis, catalysis, and mechanistic studies and contributed to writing the manuscript. CO and MS performed all computational work. SYHK assisted with EPR experiments and catalyst stability studies. RTB and MHB provided supervision, funding, ideation and contributed to writing the manuscript.

## Conflicts of interest

There are no conflicts to declare.

## Supplementary Material

SC-013-D2SC04339D-s001

SC-013-D2SC04339D-s002
