# Supplementary material for: Spin-state crossover in photo-catalyzed nitrile dihydroboration via Mn-thiolate cooperation
Source: Chem Sci. 2022 Oct 20;13(42):12550–9. doi: 10.1039/d2sc04339d (PMC9629026; doi:10.1039/d2sc04339d)
Supplement: SC-013-D2SC04339D-s001 [file SC-013-D2SC04339D-s001.pdf]

Electronic Supplementary Material (ESI) for Chemical Science.
This journal is © The Royal Society of Chemistry 2022

Spin-state crossover in photo-catalyzed nitrile dihydroboration via Mn-thiolate cooperation

Matthew R. Elsby,[#,†] Changjin Oh,[#,‡] Mina Son,[‡] Scott Y. H. Kim,[†] Mu-Hyun Baik*[,‡] and R.Tom Baker*[,†]

[†] Department of Chemistry and Biomolecular Sciences and Centre for Catalysis Research and Innovation,University of Ottawa, Ottawa, Ontario K1N 6N5, Canada

[†] Department of Chemistry, Korea Advanced Institute of Science and Technology (KAIST) and Center for Catalytic Hydrocarbon Functionalizations Institute for Basic Science (IBS), Daejeon 34141, Korea

rbaker@uottawa.ca; mbaik2805@kaist.ac.kr

Electronic Supplementary Information:

Table of Contents:

I. General Considerations..S2

II. Synthetic Procedures..S2

III. Catalysis Procedures..S3

IV. Mechanistic Studies..S6

V. Spectroscopic Data..S8

VI. Crystallographic Details…..S23

VII. DFT Calculations..S24

VIII. References..S81

I. General Considerations.

Unless otherwise stated, all reactions were carried out under an atmosphere of dry, oxygen-free dinitrogen by means of standard Schlenk or glovebox techniques. Benzene–d_6 and acetonitrile-d_3 were degassed by three freeze-pump-thaw cycles, and subsequently dried by running through a column of activated alumina. THF-d_8 was run through a small plug of activated alumina before use. Toluene, hexanes, diethyl ether, acetonitrile, and THF were dried on columns of activated alumina using a J. C. Meyer (formerly Glass Contour) solvent purification system and stored over activated 4 Å molecular sieves. Photolysis was performed with a High Intensity UVA/UVB Tekizoo Sun Lamp. ^{1}H, ^{13}C{^{1}H}, and ^{11}B NMR spectra were recorded on Bruker AvanceII and AvanceIII spectrometers operating at 300 or 400, and 500 MHz respectively, with respect to proton nuclei. ^{1}H NMR spectra were referenced to residual protons (C_6D_6, δ 7.15), (CD_3CN, δ 1.96), (C_4D_8O, δ 3.58) with respect to tetramethylsilane at δ 0.00. ^{13}C{^{1}H} NMR spectra were referenced to relative solvent resonances (C_6D_6, δ 128.26, CD_3CN, δ 118.26). EPR spectra were recorded on a Bruker Elexsys E580 X-band spectrometer and were modelled using EasySpin (v 5.25), a package developed by Stoll and Schweiger based on Matlab.[1] Optimized values were searched for the following parameters: isotropic g value (g_{iso}), zero field splitting parameters (D and E), hyperfine coupling constants (a_{Mn} and a_N), and isotropic peak-to-peak line width (ΔB). FT-IR data were collected on a Thermo Scientific Nicolet 6700 spectrometer. All reagents were purchased from commercial suppliers. The [S^{Me}N^{H}S] ligand (**L^H**) was synthesized according to a literature procedure.[2]

II. Synthetic Protocols

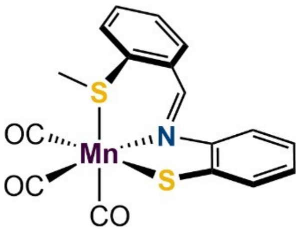

Synthesis of [Mn(κ^3-SNS^{Me})(CO)$_3$] (1). A vial equipped with a magnetic stir bar was charged with $Mn(CO)_5Br$ (0.200 g, 0.730 mmol) and 5 mL of toluene. A solution of S^{Me}N^{H}S (0.188 g, 0.730 mmol) and lithium[bis(trimethylsilyl)amide] (0.122 g, 0.730 mmol) in 2 mL of toluene was added dropwise to the suspension, giving an immediate color change from yellow-orange to reddish-brown. The solution was stirred for 16 h, filtered through Celite, and concentrated under vacuum. The residue was washed with Et_2O (3 x 2 mL), hexane (3 x 2 mL), and dried under vacuum to yield 0.253 g of a red-brown powder (92% yield). Crystals suitable for single crystal X-ray diffraction were obtained from a concentrated acetonitrile solution at -30 °C. ^{1}H NMR (CD_3CN, 22 °C, 300 MHz): δ 2.47 (br s, 3H, S–C*H*$_3$); 6.94 (ddd, 1H, Ar–*H*, $^4J_{HH}$ = 1.5 Hz, $^3J_{HH}$ = 7 Hz, $^3J_{HH}$ = 8.5 Hz); 7.05 (ddd, 1H, Ar–*H*, $^4J_{HH}$ = 1.5 Hz, $^3J_{HH}$ = 7 Hz, $^3J_{HH}$ = 8 Hz); 7.42 (dd, 1H, Ar–*H*, $^4J_{HH}$ = 1.5 Hz, $^3J_{HH}$ = 8

Hz); 7.49 (dd, 1H, Ar–*H*, $^{4}J_{HH}$ = 1.5 Hz, $^{3}J_{HH}$ = 8 Hz); 7.64 (multiplet, 2H, Ar–*H*); 7.81 (multiplet, 2H, Ar–*H*); 8.72 (s, 1H, imine–*H*). ^{13}C{^{1}H} NMR (CD_3CN, 22 °C, 75 MHz): δ 19.5 (s, S–*C*H_3); 110.5 (s, Ar–*C*); 120.6 (s, Ar–*C*); 122.4 (s, Ar–*C*); 126.1 (s, Ar–*C*); 127.6 (s, Ar–*C*); 129.1 (s, Ar–*C*); 131.6 (s, Ar–*C*); 133.5 (s, Ar–*C*); 134.9 (s, Ar–*C*); 151.5 (s, Ar–*C*); 15.0 (s, Ar–*C*); 164.3 (s, N=*C*H); 216.6 (s, Mn–*C*O); 220.1 (s, Mn–*C*O); 222.7 (s, Mn–*C*O). IR (ATM, cm^{-1}): 1898, 1922, 2009 (CO). HRMS (ESI-QTOF, C_6H_5Cl): Calcd for $C_{17}H_{12}MnNO_3S_2$ m/z 397.9717 ([M^{+}]). Found m/z 397.9736.

III. Catalysis Protocols

General Procedure 'A' for Dihydroboration of Nitriles.

A catalyst stock solution was first prepared by dissolving **1** (10 mg) in C_6D_6 (1 mL). A vial containing **1** (0.001 g, 100 μL, 0.003 mmol, 1 mol%) in 0.6 g of THF was charged first with nitrile substrate (0.32 mmol), and subsequently with 2.2 equiv. pinacolborane (0.090 g, 102 μL, 0.70 mmol) to give a dark yellow-brown solution. The solution was charged to an NMR tube, removed from the glovebox, and placed 15 cm away from a UV light sun lamp for the appropriate reaction time. Yield was determined by ^{1}H NMR in reference to internal standard mesitylene. In the glovebox, volatile materials were removed under reduced pressure. Hexane was added to extract the product, the solution was filtered through Celite, and placed in the freezer at -35 °C overnight to afford white crystals.

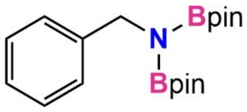

Dihydroboration of benzonitrile. Conducted according to *General Procedure A* using benzonitrile (0.032 g, 32 μL, 0.32 mmol), pinacolborane (0.090 g, 102 μL, 0.70 mmol), and catalytic **1**. The solution was placed under white light lamp with a cooling fan for 4 h (99% NMR yield). ^{1}H NMR shifts matched with literature values.[3]

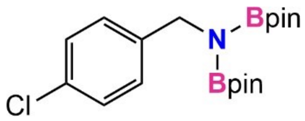

Dihydroboration of 4-chlorobenzonitrile. Conducted according to *General Procedure A* using 4-chlorobenzonitrile (0.044 g, 0.32 mmol), pinacolborane (0.090 g, 102 μL, 0.70 mmol), and catalytic **1**. The solution was placed under white light lamp with a cooling fan for 4h (99% NMR yield). ^{1}H NMR shifts matched with literature values.[3]

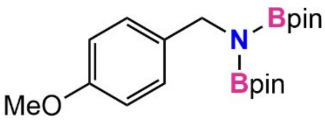

Dihydroboration of 4-methoxybenzonitrile. Conducted according to *General Procedure A* using 4-methoxybenzonitrile (0.042 g, 0.32 mmol), pinacolborane (0.090 g, 102 µL, 0.70 mmol), and catalytic **1**. The solution was placed under white light lamp with a cooling fan for 4 h (99% NMR yield). ^{1}H NMR shifts matched with literature values.[3]

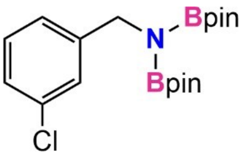

Dihydroboration of 3-chlorobenzonitrile. Conducted according to *General Procedure A* using 3-chlorobenzonitrile (0.044 g, 0.32 mmol), pinacolborane (0.090 g, 102 µL, 0.70 mmol), and catalytic **1**. The solution was placed under white light lamp with a cooling fan for 6 h (92% NMR yield). ^{1}H NMR shifts matched with literature values.[3]

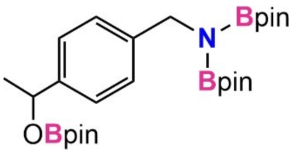

Trihydroboration of 4-acetly-benzonitrile. Conducted according to *General Procedure A* using 4-acetyl-benzonitrile (0.022 g, 29 µL, 0.32 mmol), pinacolborane (0.135 g, 153 µL, 1.06 mmol, 3.3 equiv), and catalytic **1**. The solution was placed under white light lamp with a cooling fan for 5 h (95% NMR yield). ^{1}H NMR shifts matched with literature values.[3]

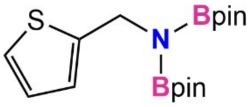

Dihydroboration of 2-cyanyl-thiophene. Conducted according to *General Procedure A* using 2-cyanyl-thiophene (0.035 g, 0.32 mmol), pinacolborane (0.090 g, 102 µL, 0.70 mmol), and catalytic **1**. The solution was placed under white light lamp with a cooling fan for 5 h (96% NMR yield). ^{1}H NMR shifts matched with literature values.[3]

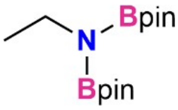

Dihydroboration of acetonitrile. Conducted according to *General Procedure A* using acetonitrile (0.013 g, 17 µL, 0.32 mmol), pinacolborane (0.090 g, 102 µL, 0.70 mmol), and catalytic **1**. The solution was placed under white light lamp with a cooling fan for 8h (95% NMR yield). ^{1}H NMR shifts matched with literature values.[3]

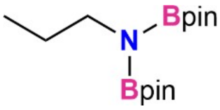

Dihydroboration of propionitrile. Conducted according to *General Procedure A* using propionitrile (0.017 g, 22 µL, 0.32 mmol), pinacolborane (0.090 g, 102 µL, 0.70 mmol), and catalytic **1**. The solution was placed under white light lamp with a cooling fan for 8 h (92% NMR yield). ^{1}H NMR shifts matched with literature values.[3]

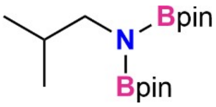

Dihydroboration of isobutylnitrile. Conducted according to *General Procedure A* using isobutylnitrile (0.022 g, 29 µL, 0.32 mmol), pinacolborane (0.090 g, 102 µL, 0.70 mmol), and catalytic **1**. The solution was placed under white light lamp with a cooling fan for 8 h (85% NMR yield). ^{1}H NMR shifts matched with literature values.[3]

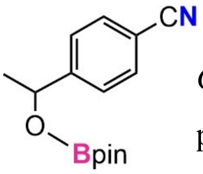

Hydroboration of 4-acetyl-benzonitrile. Conducted according to a modified *General Procedure A* using 4-acetyl-benzonitrile (0.022 g, 29 µL, 0.32 mmol), pinacolborane (0.090 g, 102 µL, 0.70 mmol), and catalytic **1**. Reactants were mixed in a vial and subsequently charged to an NMR tube. After approx. 5 minutes at room temperature the crude ^{11}B NMR showed full conversion to the carbonyl-reduced products (99% NMR yield). ^{1}H NMR shifts matched with literature values.[4]

Hydroboration of 4-methoxystyrene. Conducted according to *General Procedure A* using 4-methoxystyrene (0.042 g, 42 µL, 0.32 mmol), pinacolborane (0.041 g, 45 µL, 0.32 mmol), and catalytic **1**. The solution was placed under white light lamp with a cooling fan for 16 h (85% NMR yield).^{11}B NMR showed incomplete conversion to the reduced product, featuring a mixture of unreacted pinacolborane and hydroborated product (23% yield by NMR). Increasing catalyst loading to 5% gave a slight increase in product conversion (48%), but not appreciable enough to pursue further substrates. See Figure S23.

Larger Scale Dihydroboration of benzonitrile. Conducted according to *General Procedure A* in a 10 mL scintillation vial using: benzonitrile (0.250 g, 250 µL, 2.42 mmol), pinacolborane (0.683 g, 775 µL, 5.32 mmol) and catalytic **1** (0.048 g, 0.121 mmol). The solution was irradiated with a UV light sun lamp for 10 hours. Workup according to *General Procedure A* yielded 0.75 g of diborylamine product (86% yield).

Control Studies

Attempted dihydroboration with HL1 and/or $MnCO_5Br$. A solution of catalytic $MnCO_5Br$ (0.01 g, 0.025 mmol) in THF was charged with benzonitrile (0.032 g, 32 µL, 0.32 mmol) and pinacolborane (0.090 g, 102 µL, 0.70 mmol). Another solution of catalytic $MnCO_5Br$ (0.01 g, 0.025 mmol) and $S^{Me}N^{H}S$ (0.009 g, 0.025 mmol) in THF was charged with benzonitrile (0.032 g, 32 µL, 0.32 mmol) and pinacolborane (0.090 g, 102 µL, 0.70 mmol). Both reactions were irradiated with a UV light sun lamp for 16 h. Analysis of the crude ^{11}B NMR spectra showed less than 10% conversion to the reduced diborylamine product.

Hydroboration of 4-acetyl-benzonitrile in absence of light. Conducted according to a modified *General Procedure A* using 4-acetyl-benzonitrile (0.022 g, 29 µL, 0.32 mmol), pinacolborane (0.135 g, 153 µL, 1.06 mmol, 3.3 equiv),and catalytic **1**. A solution of 4-acetyl benzonitrile and **1** was added to an NMR tube wrapped in aluminum foil to protect from light. Pinacolborane was charged to the light-protected reaction mixture. The NMR tube was enclosed in a box to avoid possible light exposure and kept overnight. Analysis of the crude NMR the next day showed conversion to exclusively the carbonyl reduced product with ca. 2 equiv. of unreacted HBpin.

IV. Mechanistic Studies

Stoichiometric Studies

Stoichiometric reaction of 1 and HBpin. A dark yellow-brown solution of **1** (0.01 g, 0.025 mmol) and HBpin (0.003 g, 3.5 µL, 0.025 mmol) in 0.5 mL of THF-d_8 was prepared and charged to an NMR tube. The solution was irradiated for 30 minutes which initiated a color change to a dark red-orange. Analysis of the resultant crude ^{1}H NMR showed the disappearance of the characteristic imine C–*H* resonance at δ 8.81, accompanied by the growth of a new singlet at δ 4.85 indicative of a C–H_2 group. See **Figure S24**.

Attempted stoichiometric dihydroboration. A dark yellow-brown solution of **1** (0.01 g, 0.025 mmol) and HBpin (0.003 g, 3. µL, 0.025 mmol) in 0.5 mL of THF was prepared and charged to an NMR tube. The solution was irradiated for 30 minutes which initiated a color change to a dark red-orange and analysis of the ^{1}H NMR confirmed borylation of the imine. Benzonitrile (0.003 g, 3 µL, 0.025 mmol) and an additional equivalent of HBpin (0.003 g, 3.5 µL, 0.025 mmol) were then added to the reaction mixture, which was subsequently irradiated for an additional 4 h. No color change was seen and formation of nitrile dihydroboration product was not observed.

Analysis of crude catalysis mixture. The dihydroboration of benzonitrile was conducted according to *General Procedure A* except with a larger catalyst loading of **1** (0.015 g). After catalysis completion, the sample was worked up according to *General Procedure A*. After hexanes product extraction, the remaining crude residue was dried under reduced pressure, redissolved in CD_3CN, and analyzed by ^{1}H NMR. Analysis of the crude ^{1}H NMR showed the retention of the imine C–H resonance.

Stoichiometric reaction of 1 and benzonitrile. A dark yellow-brown solution of **1** (0.01 g, 0.025 mmol) and benzonitrile (0.003 g, 3 μL, 0.025 mmol) in 0.5 mL of acetonitrile-d_3 was prepared and charged to an NMR tube. The reaction mixture was irradiated for 2 h and no color change was observed and no change in the ^{1}H NMR spectrum was observed.

^{13}C-labelling Studies

General Preparation of ^{13}C-labelled [Mn(κ^3-SNS^{Me})(CO)$_3$] (1*). A solution of **1** (0.020 g, 0.05 mmol) in 0.6 mL THF was charged to an NMR tube equipped with a rubber septum. The NMR tube was charged with ca. 2 mL of ^{13}CO and exposed to a white-light lamp for 30 minutes. ^{13}C{^{1}H} NMR confirmed successful transfer of the ^{13}CO to the Mn complex as indicated by observed signals at 216, 220, and 222 ppm (**Figure S2**).

Dihydroboration of benzonitrile catalyzed by ^{13}C-1. A sample of **^{13}C-1** was prepared (0.01 g, 0.025 mmol, 10 mol%) in 0.6 mL THF. Benzonitrile (0.015 g, 12 μL, 0.143 mmol) and HBpin (0.041 g, 46 μL, 0.32 mmol, 2.2 equiv) were added to the solution. The NMR tube was exposed to white-light and monitored by ^{13}C{^{1}H} NMR every hour until reaction completion. The resulting ^{13}C{^{1}H} NMR spectra are shown in **Figure S24**.

EPR Studies

Observation of paramagnetic intermediate. A catalytic reaction mixture of benzonitrile, HBpin, and 10% **1** was prepared according to *General Procedure A*. An initial EPR spectrum of the mixture afforded no signal. The reaction mixture in the EPR tube was irradiated for 45 min and an EPR spectrum was recorded with continuous irradiation in the cavity. The resulting EPR spectrum is shown in **Figure S25**. The spectrum was modelled with the Easyspin tool in MATLAB as a fit for hyperfine coupling to ^{55}Mn and ^{14}N with the following parameters:

```
%%
clc
Sys.g = 1.995;
Sys.Nucs = '55Mn,14N'; %nuclei
Sys.A = [42.5 41.2]; %MHz
Sys.lwpp = 0.5; %mT for random distribution
Exp.mwFreq = 9.8; %GHz
Exp.Range = [280 420]; %sweepwidth
[C,spp] = garlic(Sys,Exp);
%%
plot(B,spc,'black',C,spp,'r');
xlabel 'Magnetic Field (mT)';
```

V. NMR Spectra

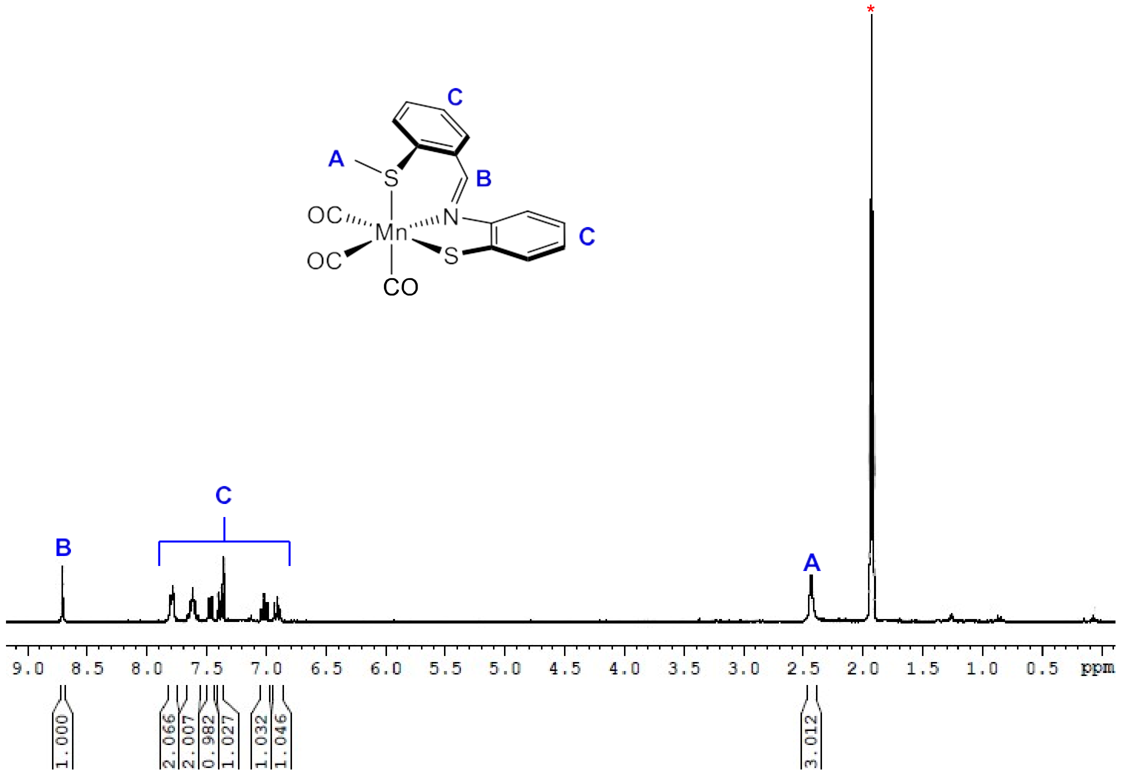

Figure S1. ^{1}H spectrum of [Mn(κ^{3}-SNS^{Me})(CO)$_3$] **(1)**. * is protic impurity in acetonitrile-d_3.

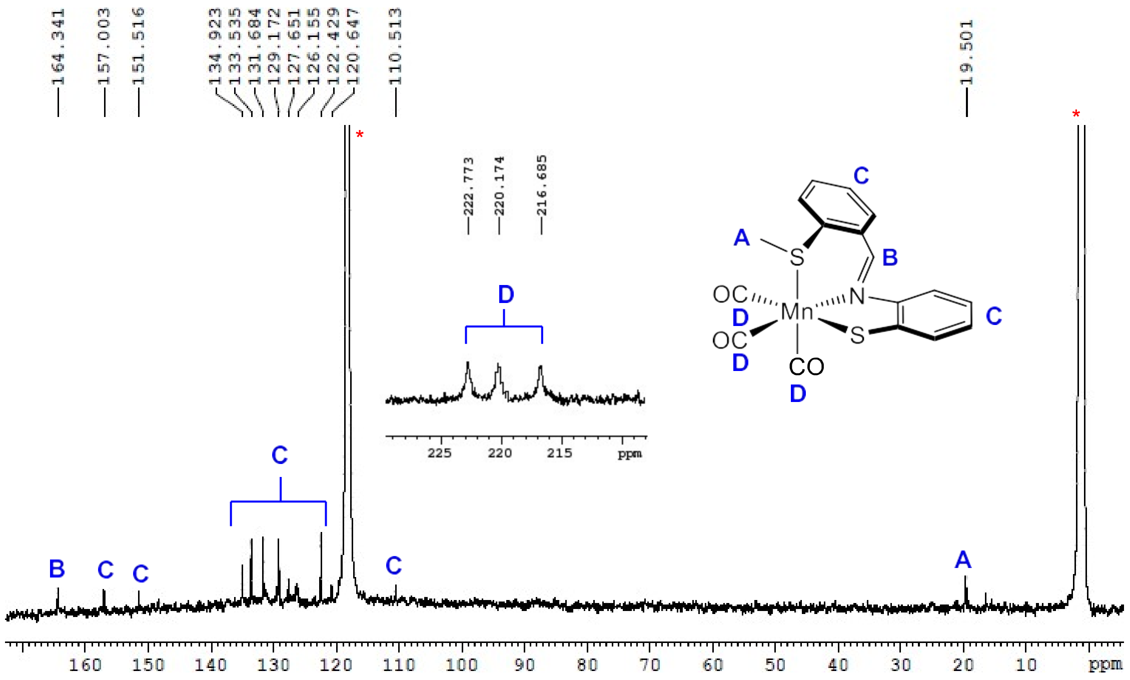

Figure S2. ^{13}C{^{1}H} spectrum of [Mn(κ^{3}-SNS^{Me})(CO)$_3$] **(1)**. * is acetonitrile-d_3.

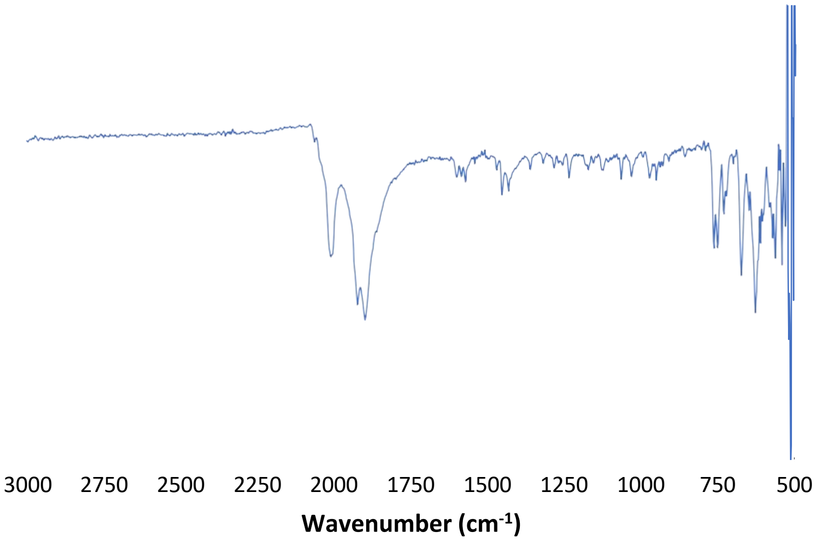

Figure S3. FT-IR (solid) spectrum of **1**.

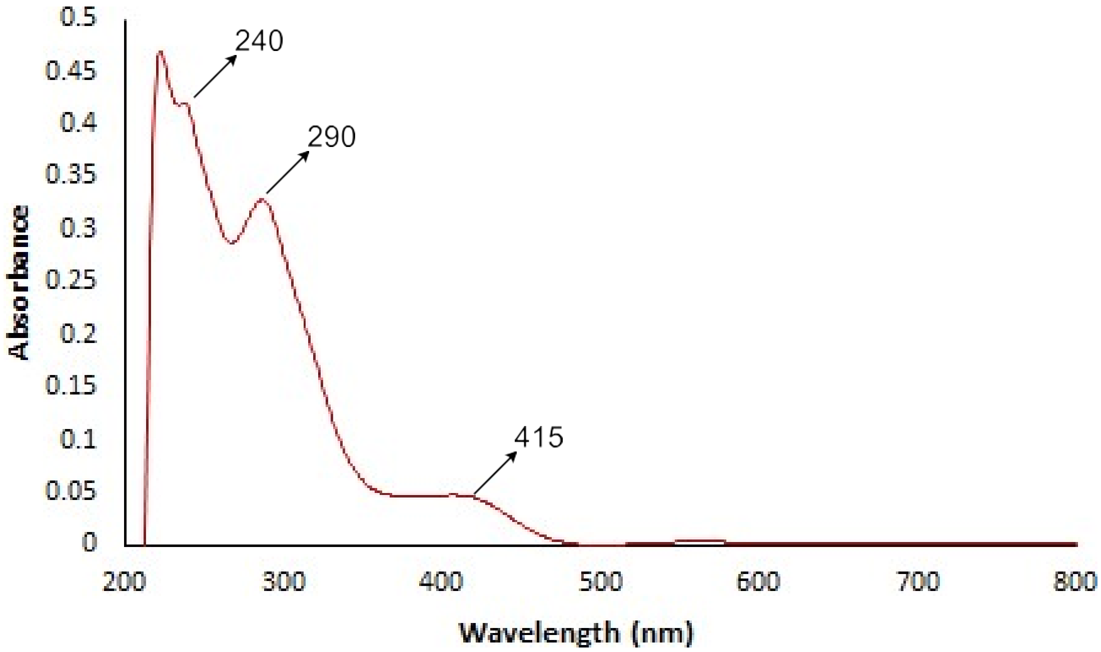

Figure S4. UV-vis spectrum of **1**.

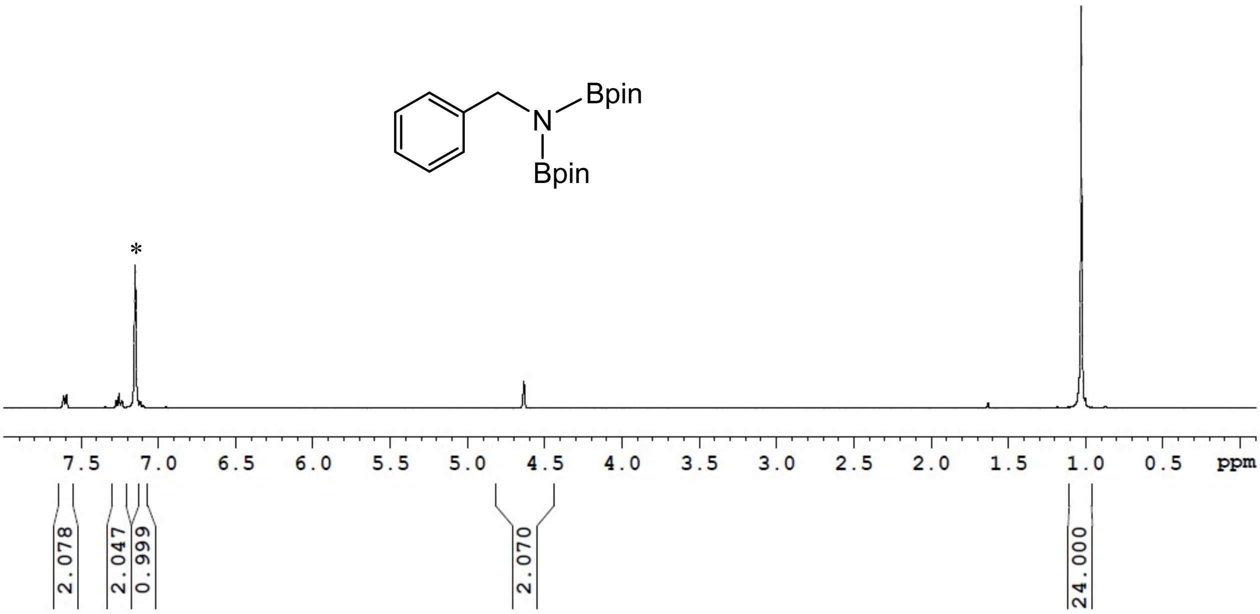

Figure S5. ^{1}H spectrum of benzonitrile dihydroboration product. * is protic impurity in benzene-d_6.

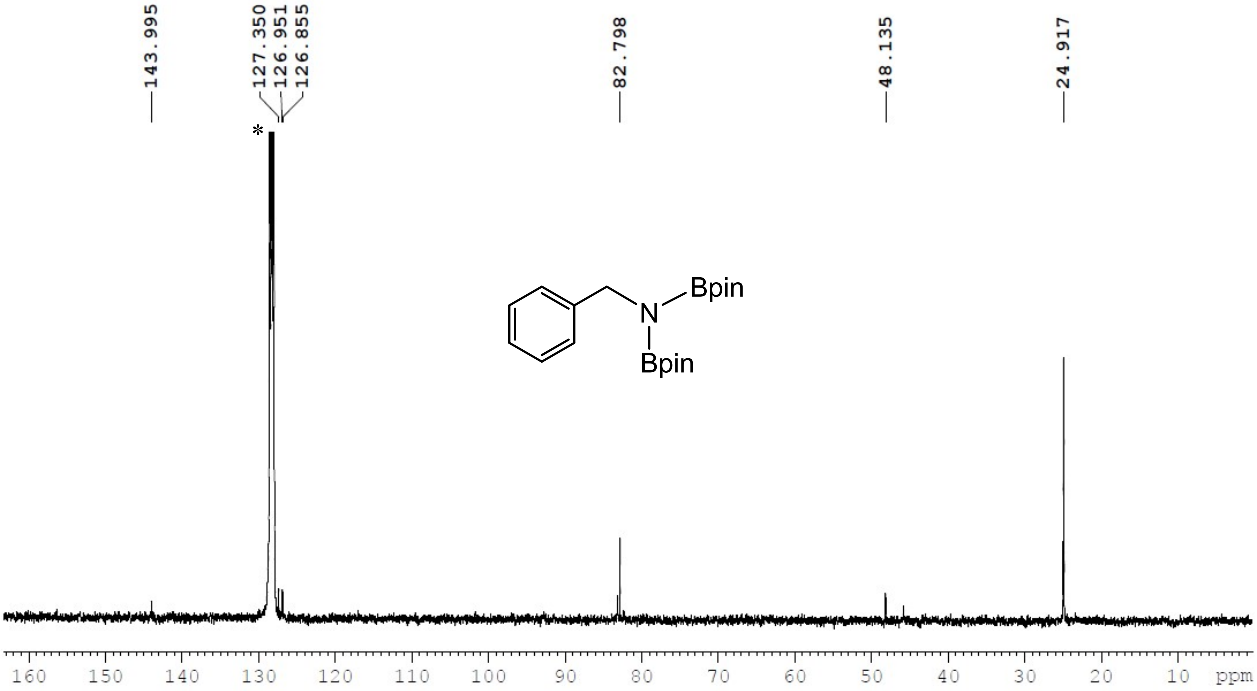

Figure S6. $^{13}C\{^{1}H\}$ spectrum of benzonitrile dihydroboration product. * is benzene-d_6.

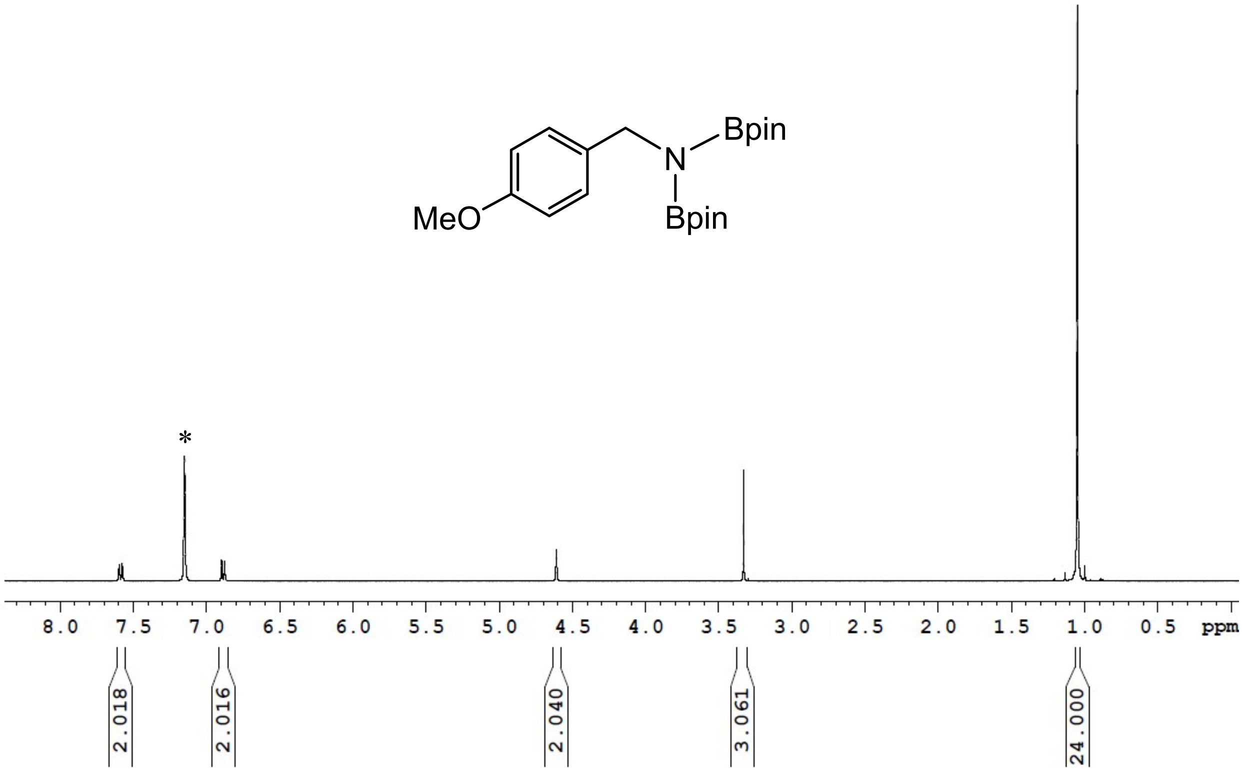

Figure S7. ^{1}H spectrum of 4-methoxy benzonitrile dihydroboration product. * is protic impurity in benzene-d_6.

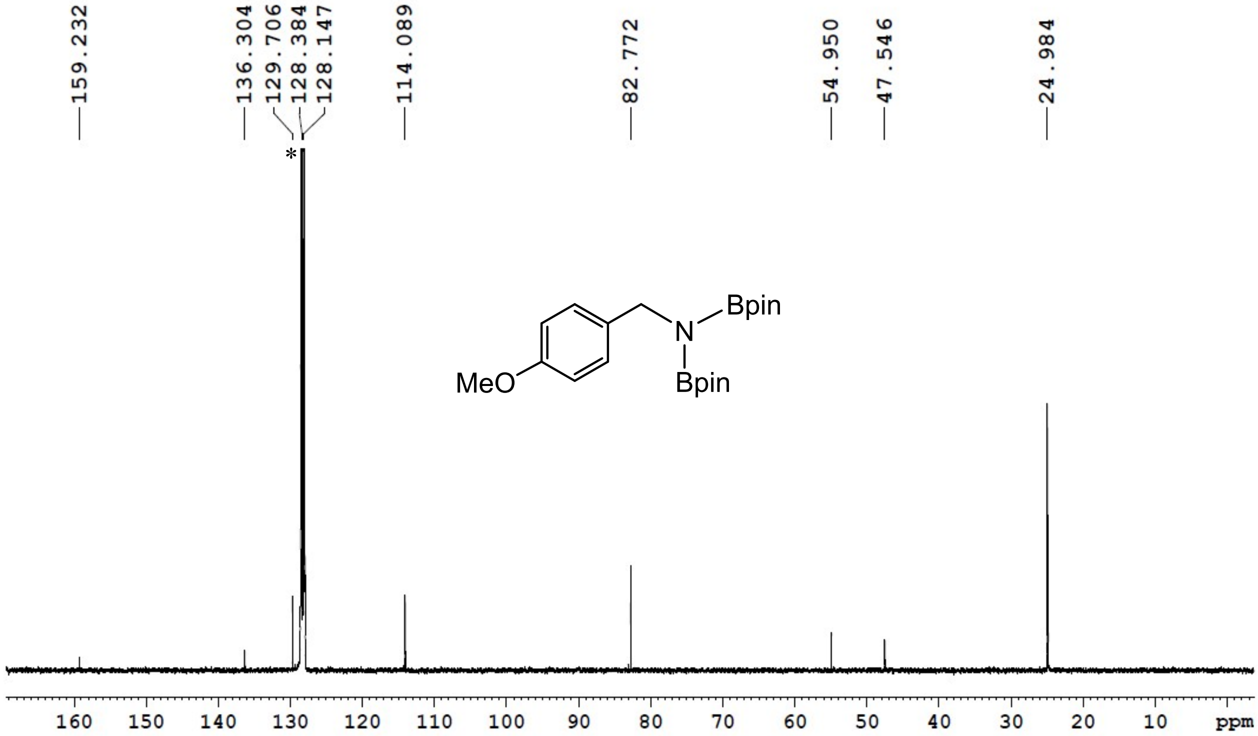

Figure S8. $^{13}C\{^{1}H\}$ spectrum of 4-methoxy benzonitrile dihydroboration product. * is benzene-d_6.

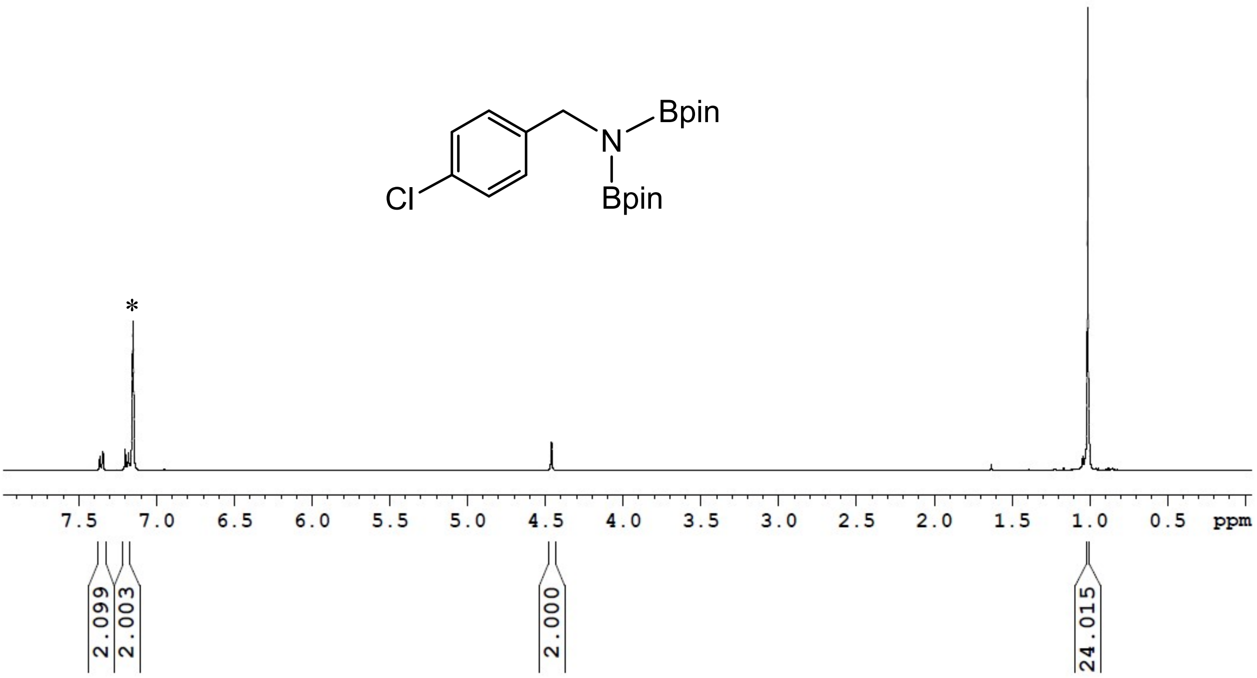

Figure S9. ^{1}H spectrum of 4-chloro benzonitrile dihydroboration product. * is protic impurity in benzene-d_6.

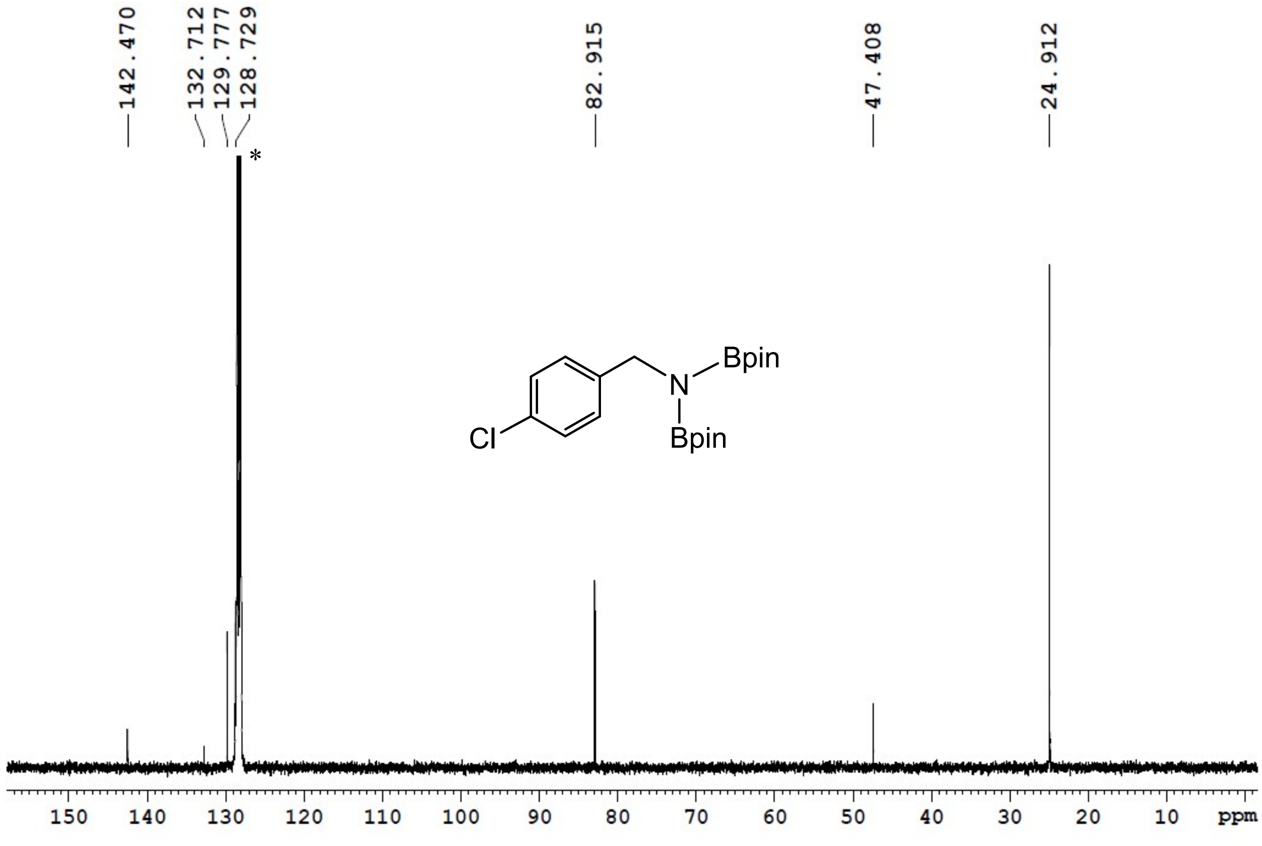

Figure S10. $^{13}C\{^{1}H\}$ spectrum of 4-chloro benzonitrile dihydroboration product. * is benzene-d_6.

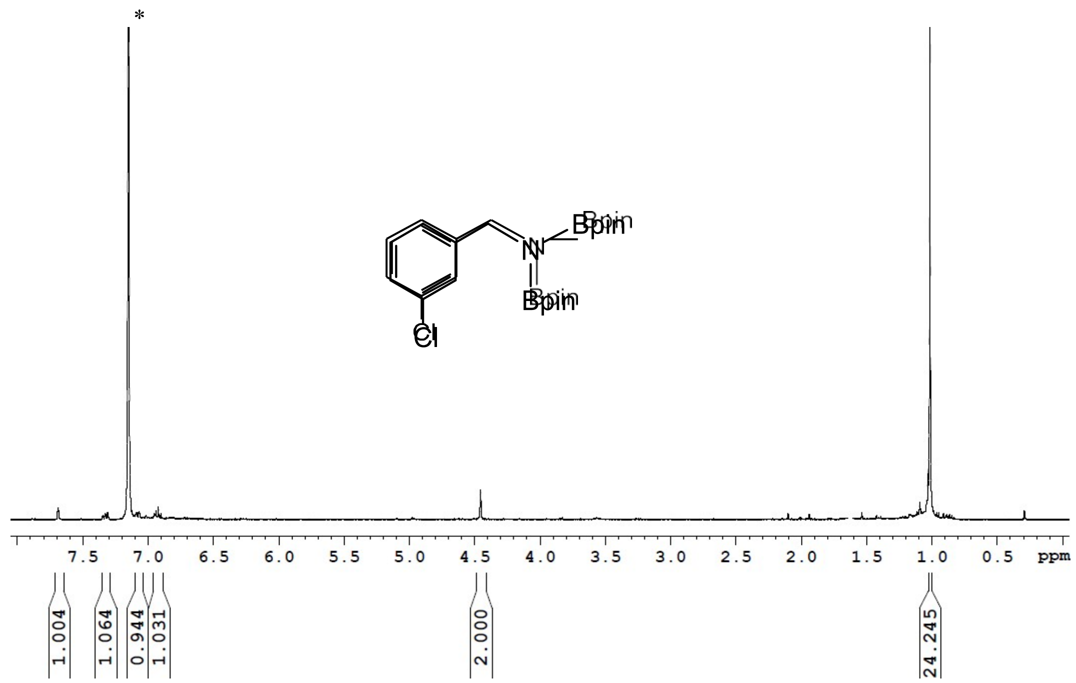

Figure S11. ^{1}H spectrum of 3-chloro benzonitrile dihydroboration product. * is protic impurity in benzene-d_6.

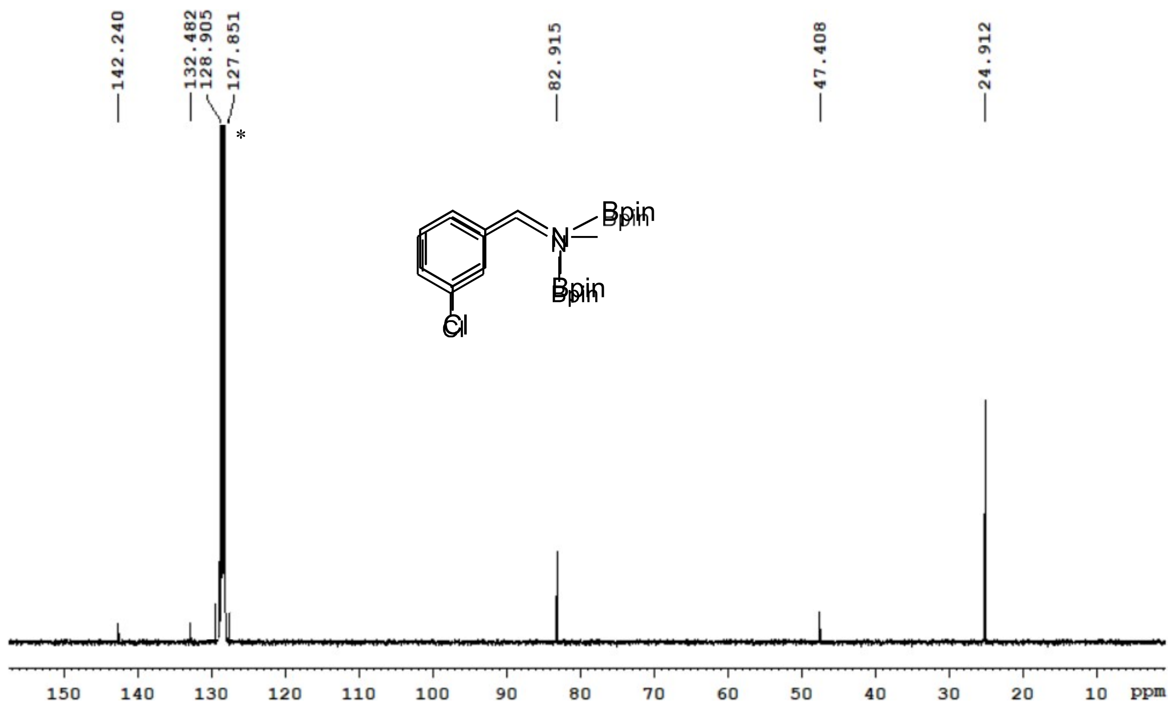

Figure S12. $^{13}C\{^{1}H\}$ spectrum of 3-chloro benzonitrile dihydroboration product. * is benzene-d_6.

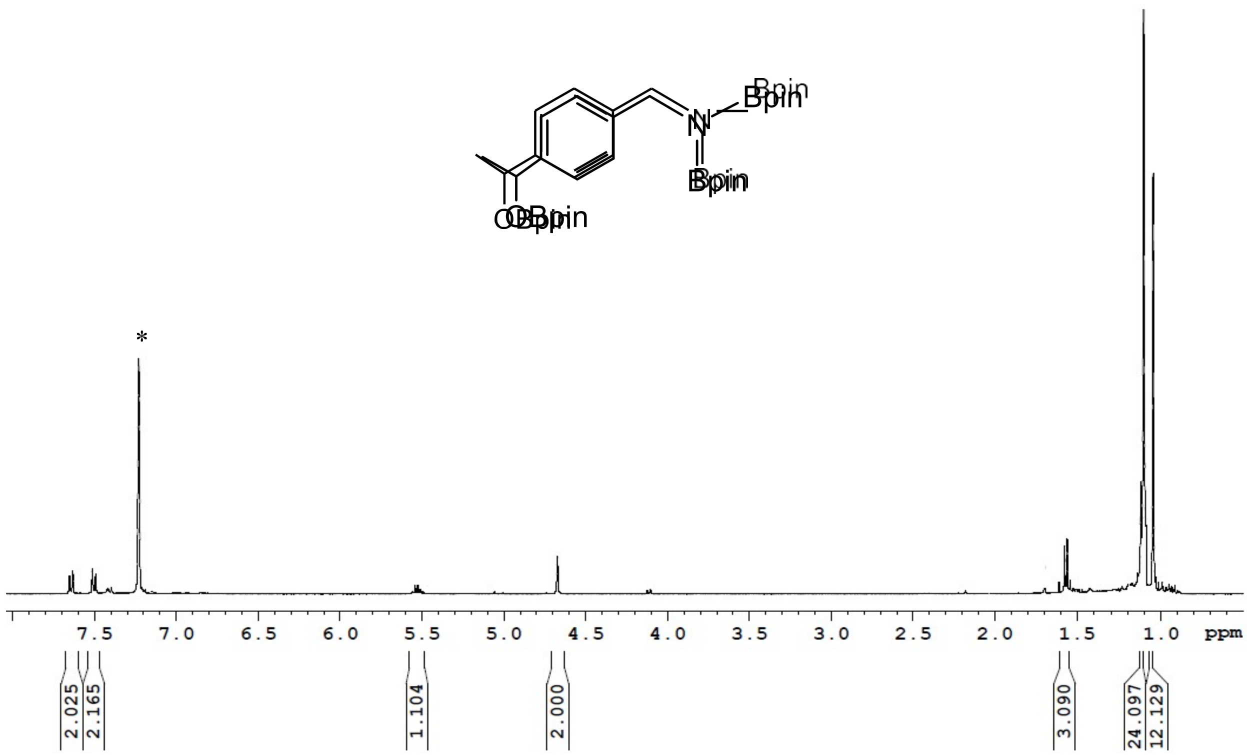

Figure S13. ^1H spectrum of 4-acetyl benzonitrile trihydroboration product. * is protic impurity in benzene-d_6.

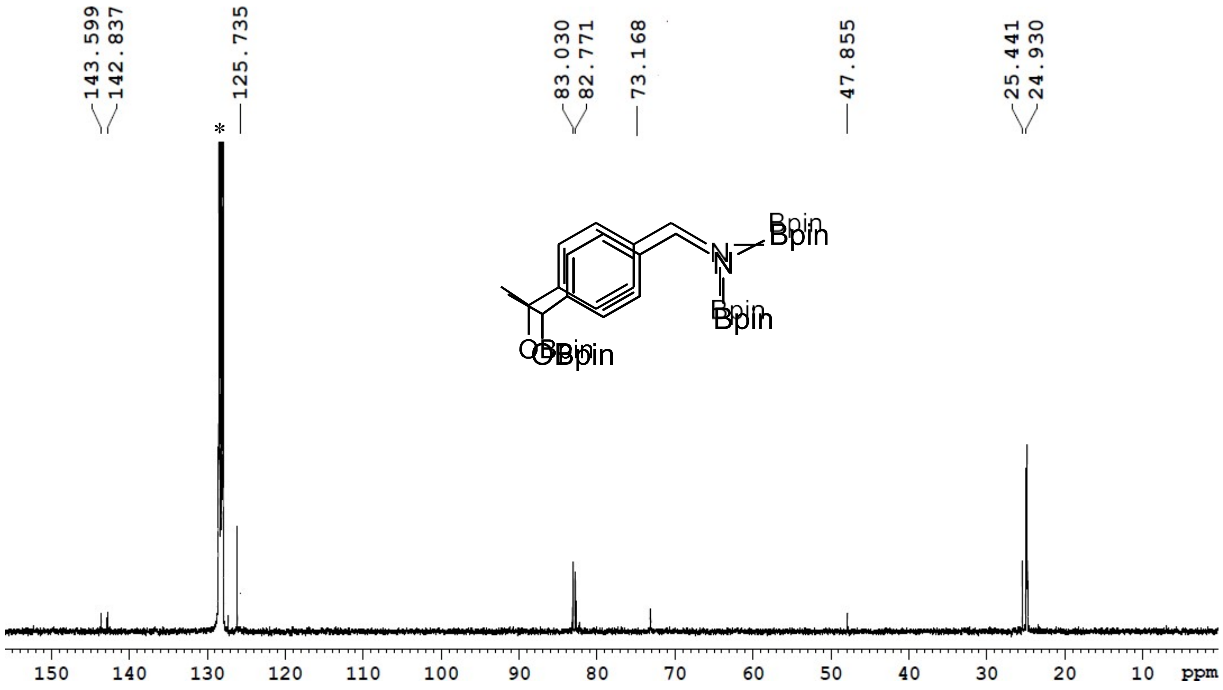

Figure S14. $^{13}C\{^1H\}$ spectrum of 4-acetyl benzonitrile trihydroboration product. * is benzene-d_6.

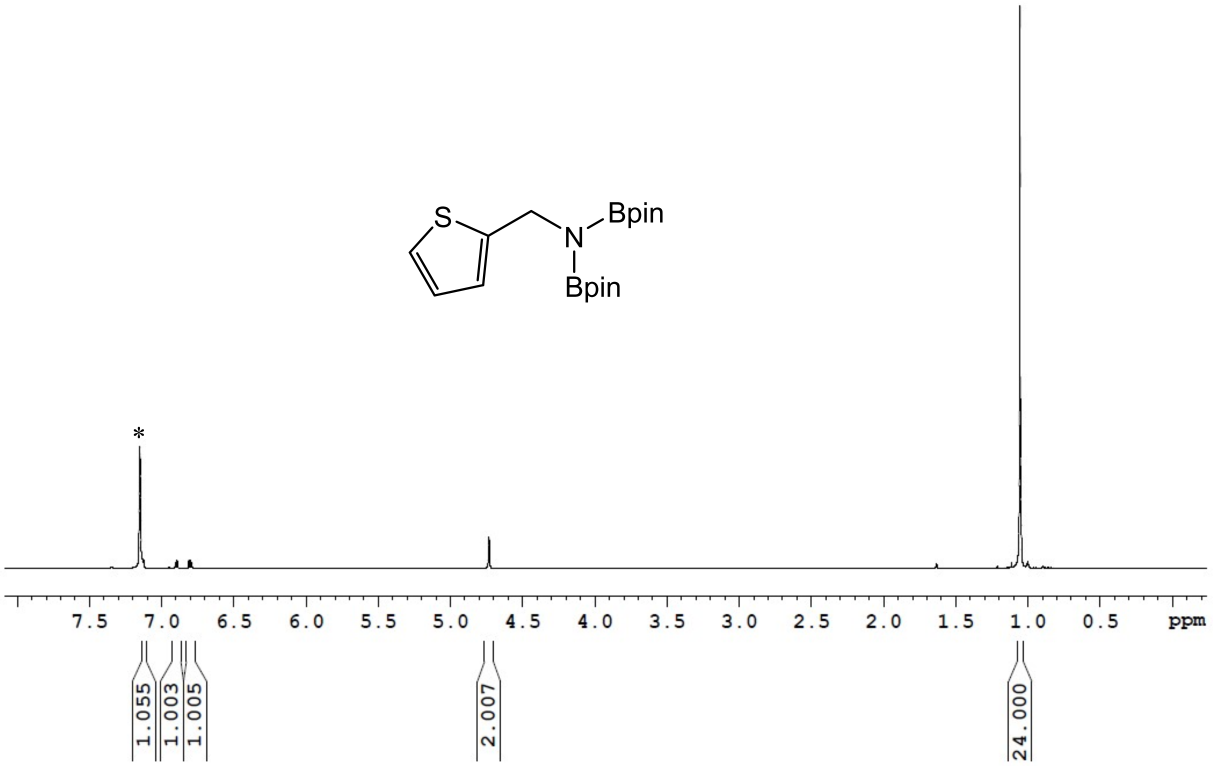

Figure S15. ^{1}H spectrum of 2-cyanyl-thiophene dihydroboration product. * is protic impurity in benzene-d_6.

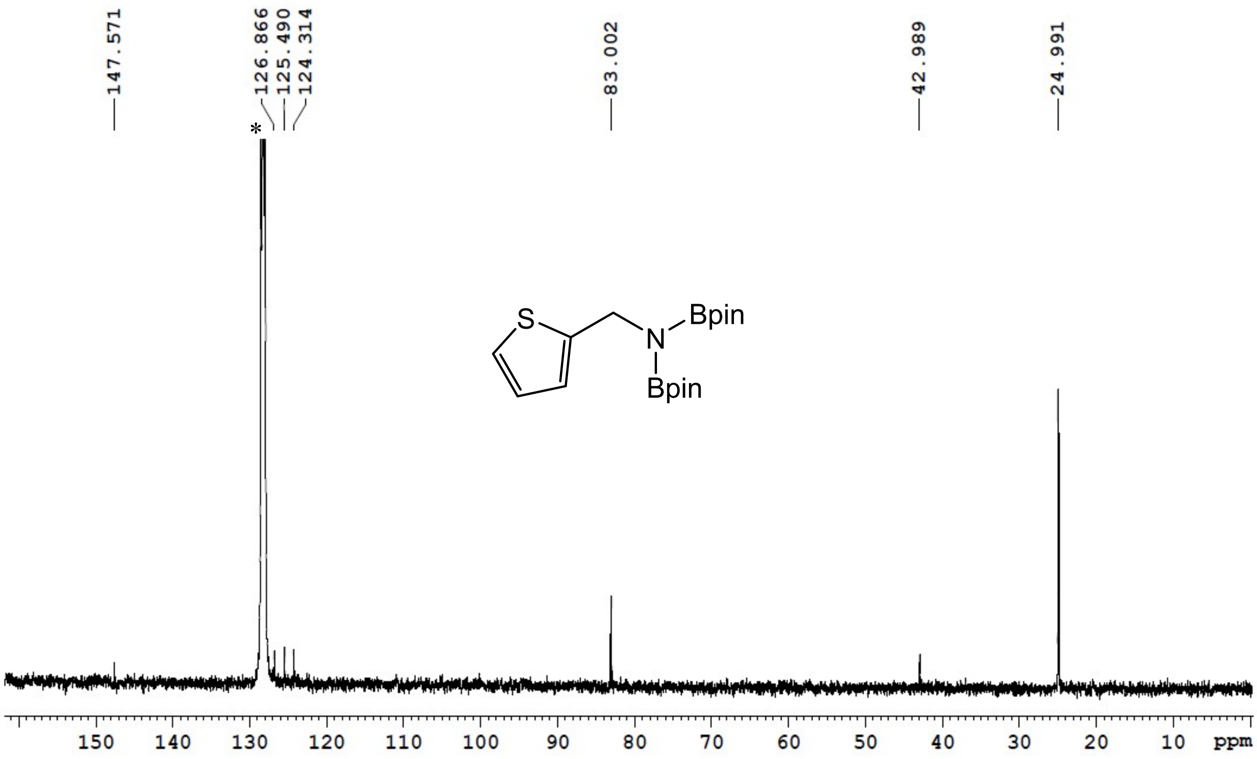

Figure S16. $^{13}C\{^{1}H\}$ spectrum of 2-cyanyl-thiophene benzonitrile dihydroboration product. * is benzene-d_6.

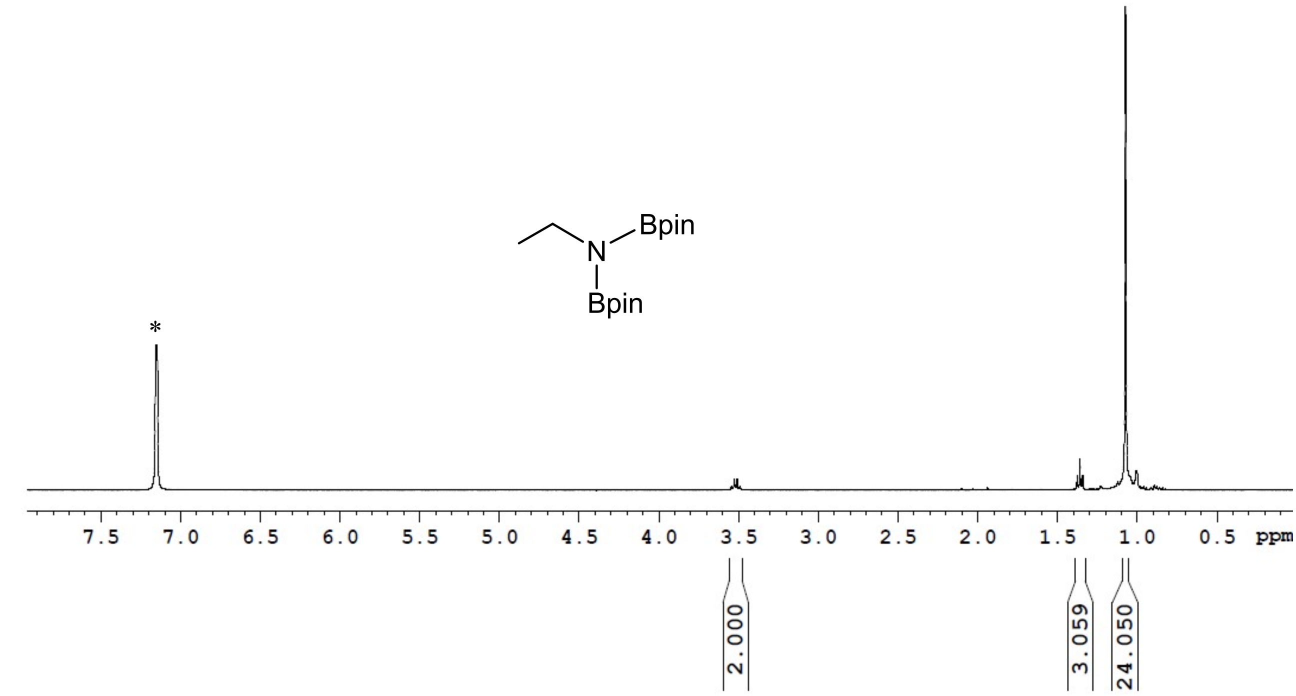

Figure S17. ^{1}H spectrum of acetonitrile dihydroboration product. * is protic impurity in benzene-d_6.

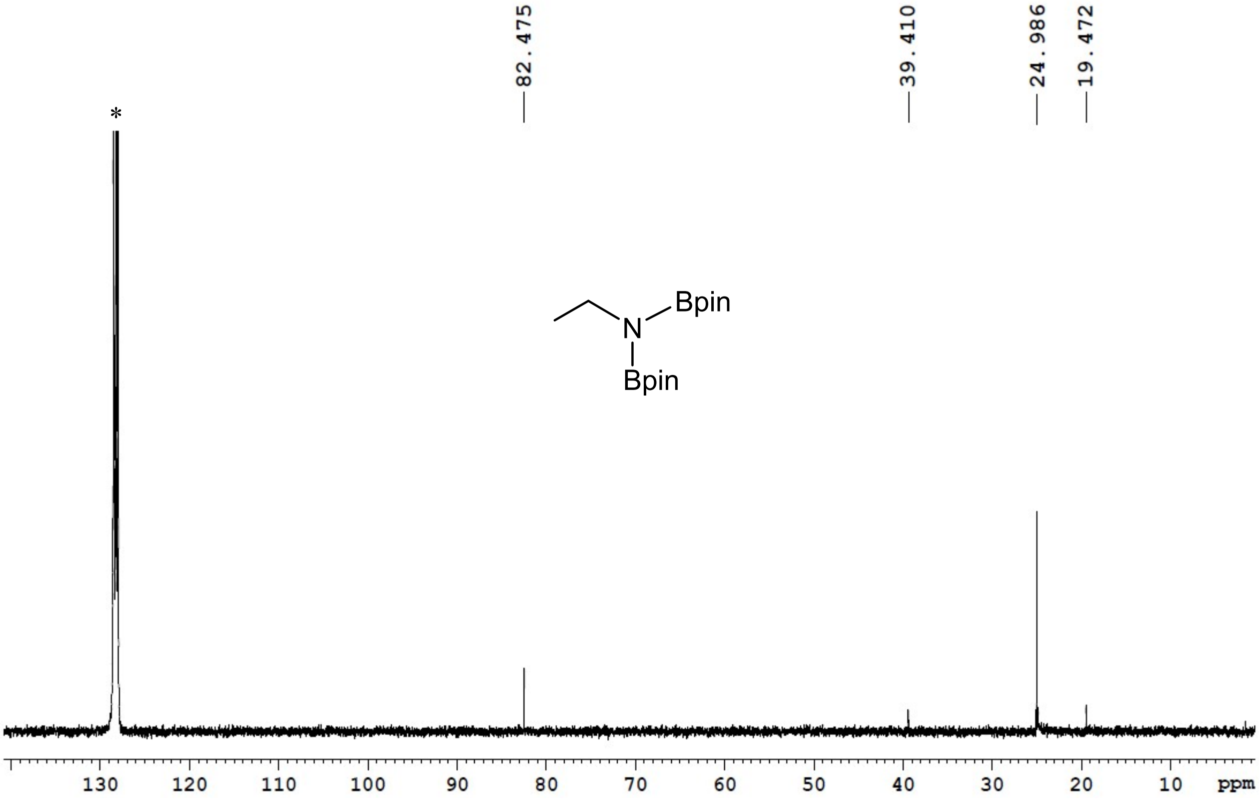

Figure S18. $^{13}C\{^{1}H\}$ spectrum of acetonitrile dihydroboration product. * is benzene-d_6.

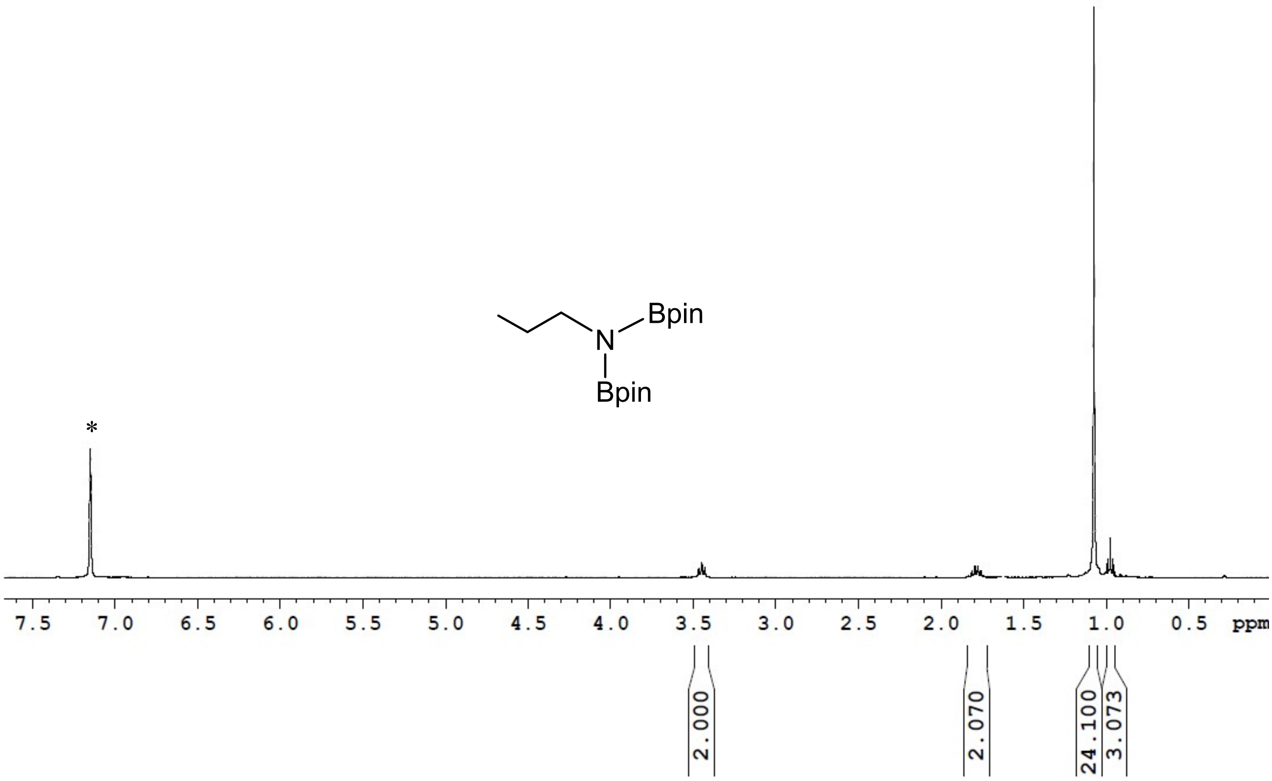

Figure S19. ^{1}H spectrum of propionitrile dihydroboration product. * is protic impurity in benzene-d_6.

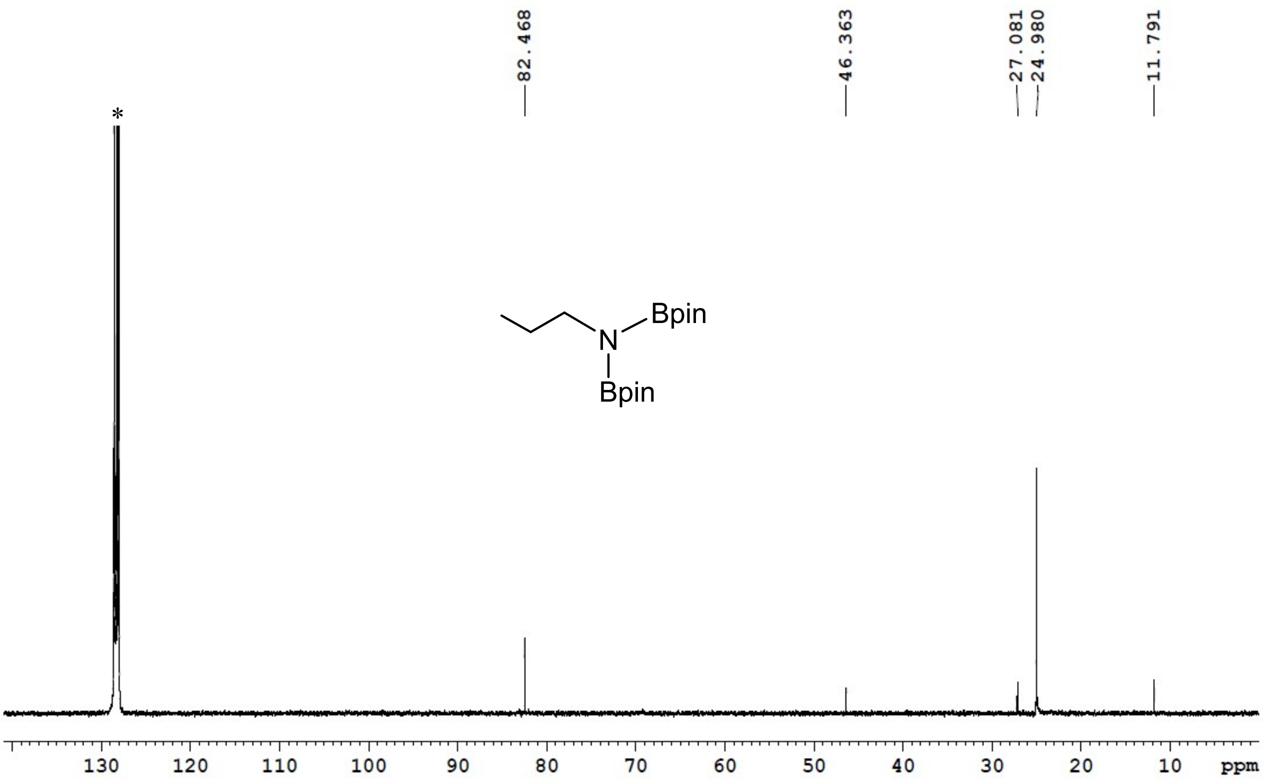

Figure S20. $^{13}C\{^{1}H\}$ spectrum of propionitrile dihydroboration product. * is benzene-d_6.

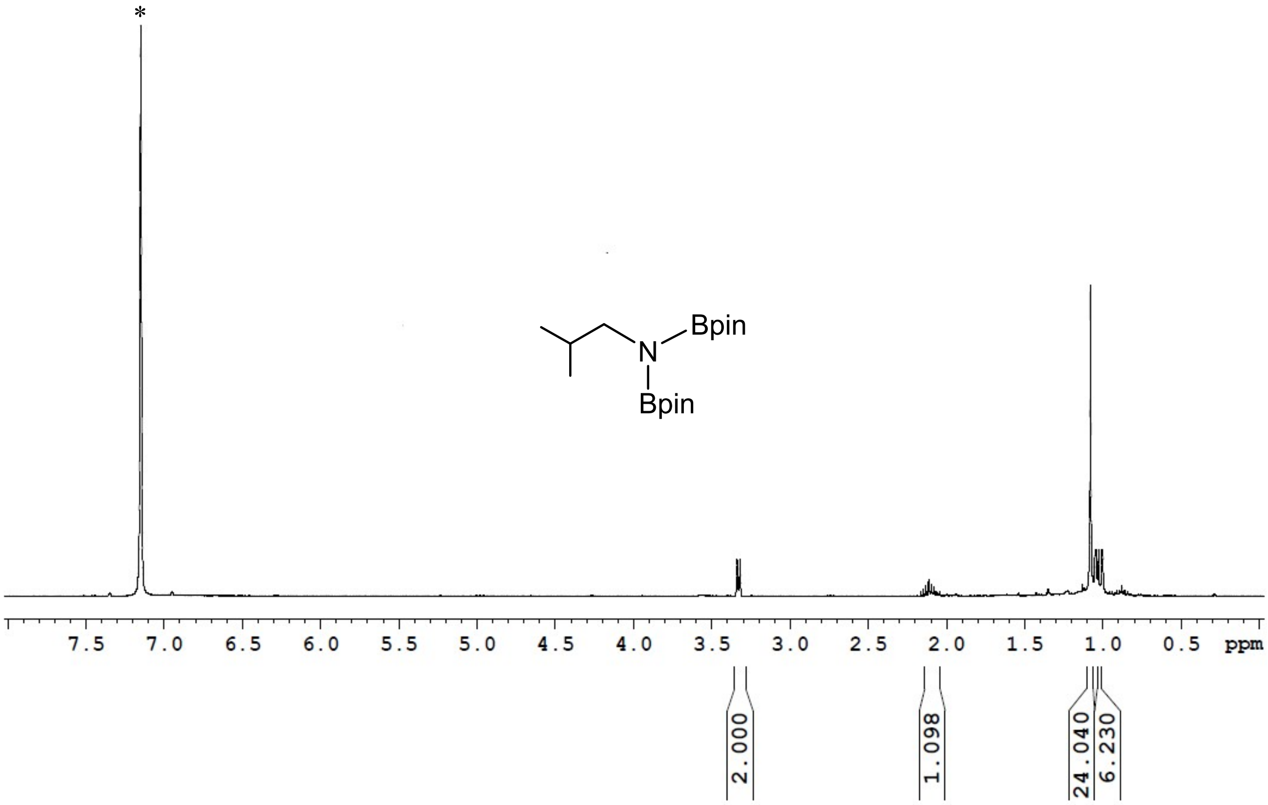

Figure S21. ^{1}H spectrum of isobutylnitrile dihydroboration product. * is protic impurity in benzene-d_6.

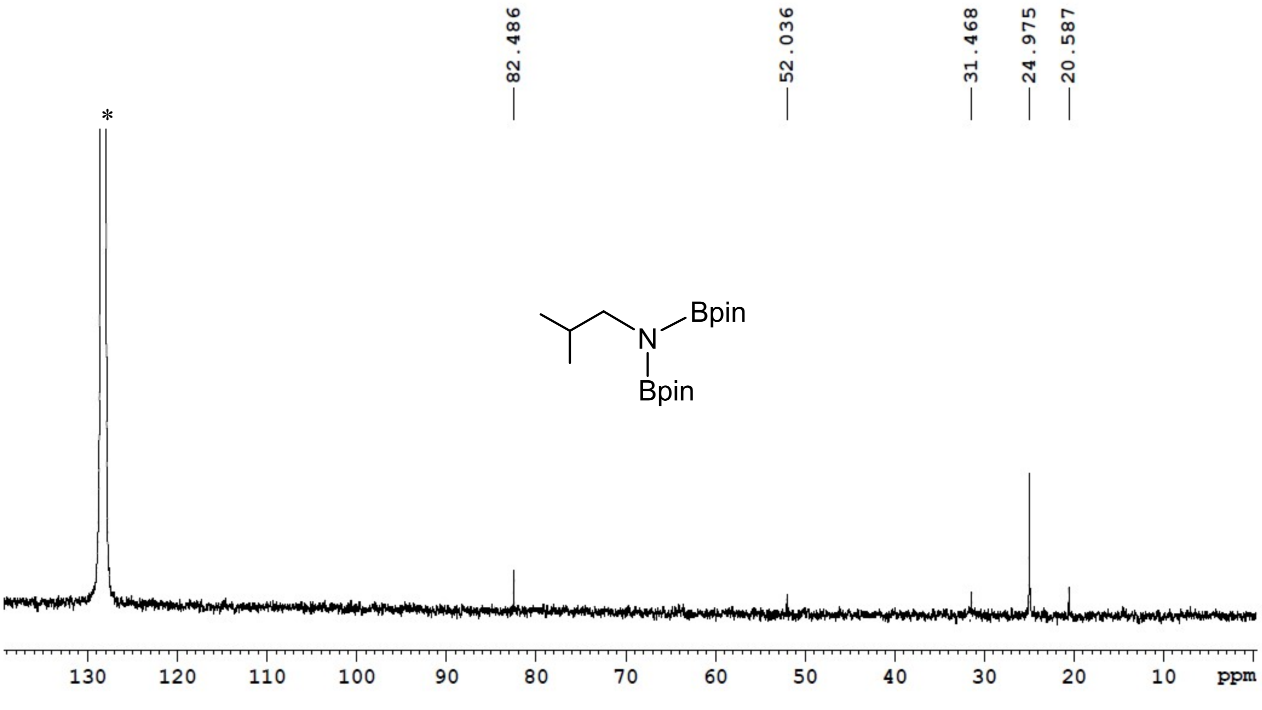

Figure S22. $^{13}C\{^{1}H\}$ spectrum of isobutylnitrile dihydroboration product. * is benzene-d^6.

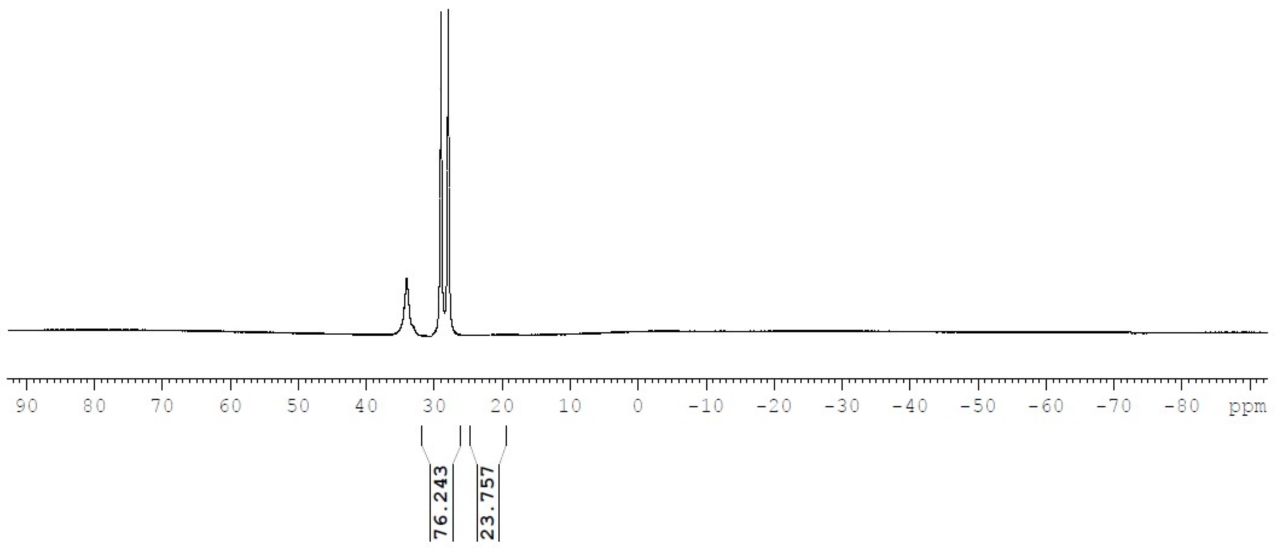

Figure S23. Crude ^{11}B NMR spectrum (96 MHz, THF) of 4-methoxystyrene hydroboration product.

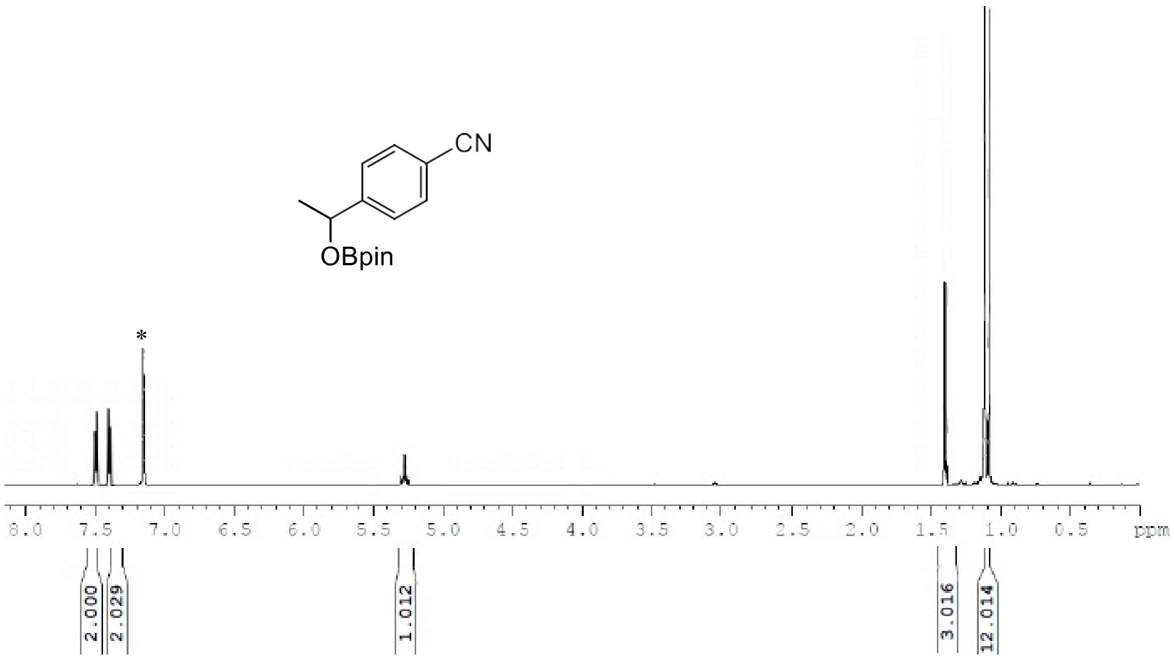

Figure S24. ^{1}H spectrum of 4-acetyl benzonitrile hydroboration product. * is protic impurity in benzene-d_6.

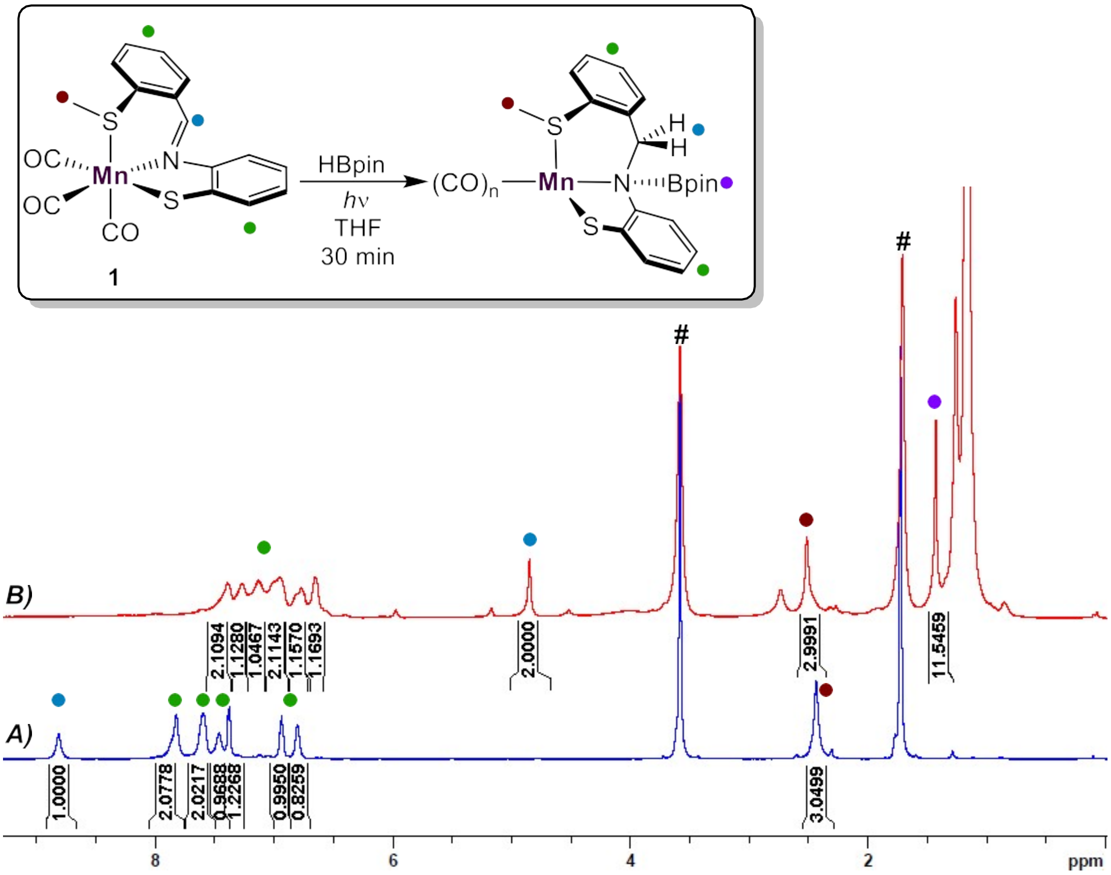

Figure S25. Stacked plot of ^{1}H NMR spectra showing A) complex **1** in THF-d_8, and B) reaction of **1** and 1.2 equiv. of HBpin – irradiated for 30 min. # is THF.

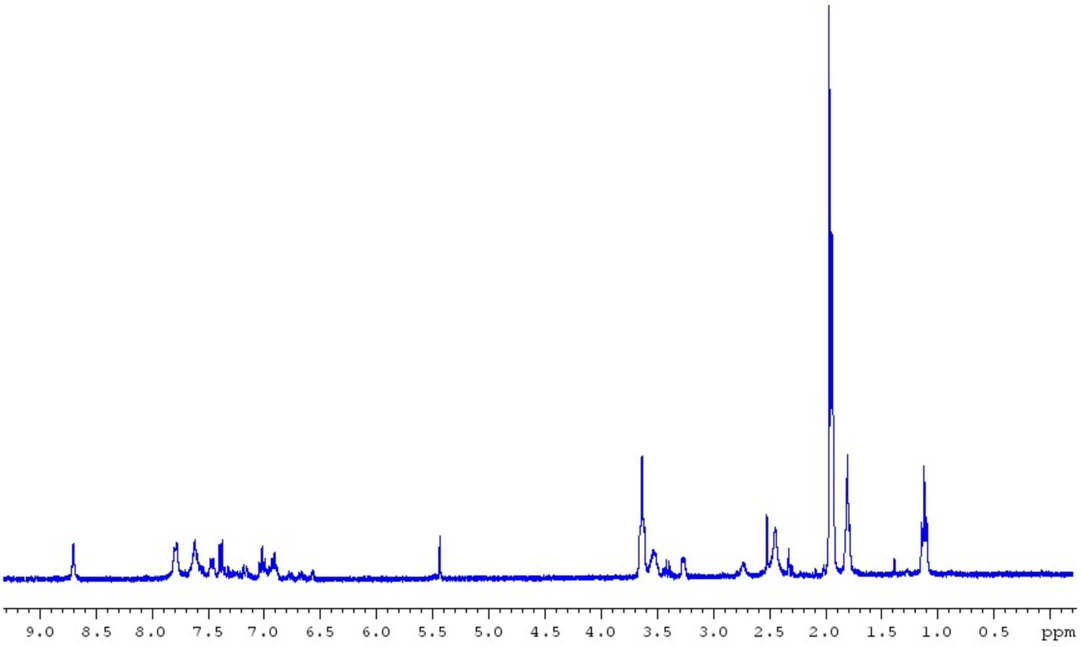

Figure S26. Crude ^{1}H NMR spectrum of crude catalyst residue after reaction workup showing retention of imine C–H resonance.

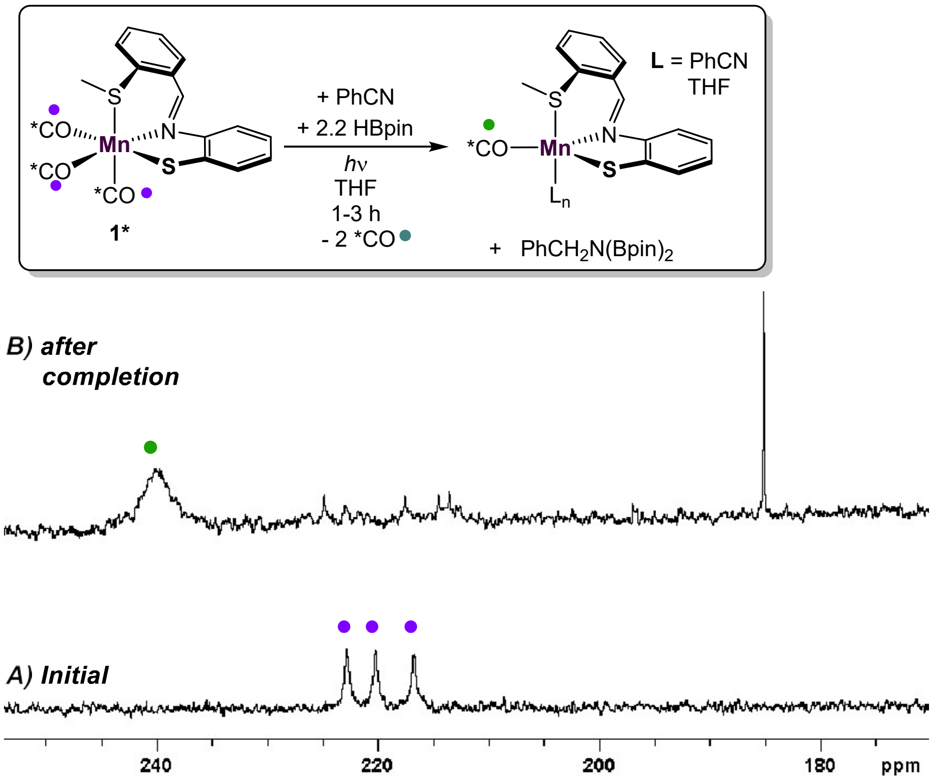

Figure S27. Stacked plot of $^{13}C\{^{1}H\}$spectra showing reaction of 10 mol% ^{13}C-labelled **1*** with benzonitrile and 2.2 equiv. of HBpin. **(A)** (Blue trace) Initial $^{13}C\{^{1}H\}$ spectrum of reaction mixture prior to light exposure showing unreacted **1***. **(B)** $^{13}C\{^{1}H\}$ spectrum of reaction mixture after completion, showing disappearance of **1*** and growth of a new species at 240 ppm.

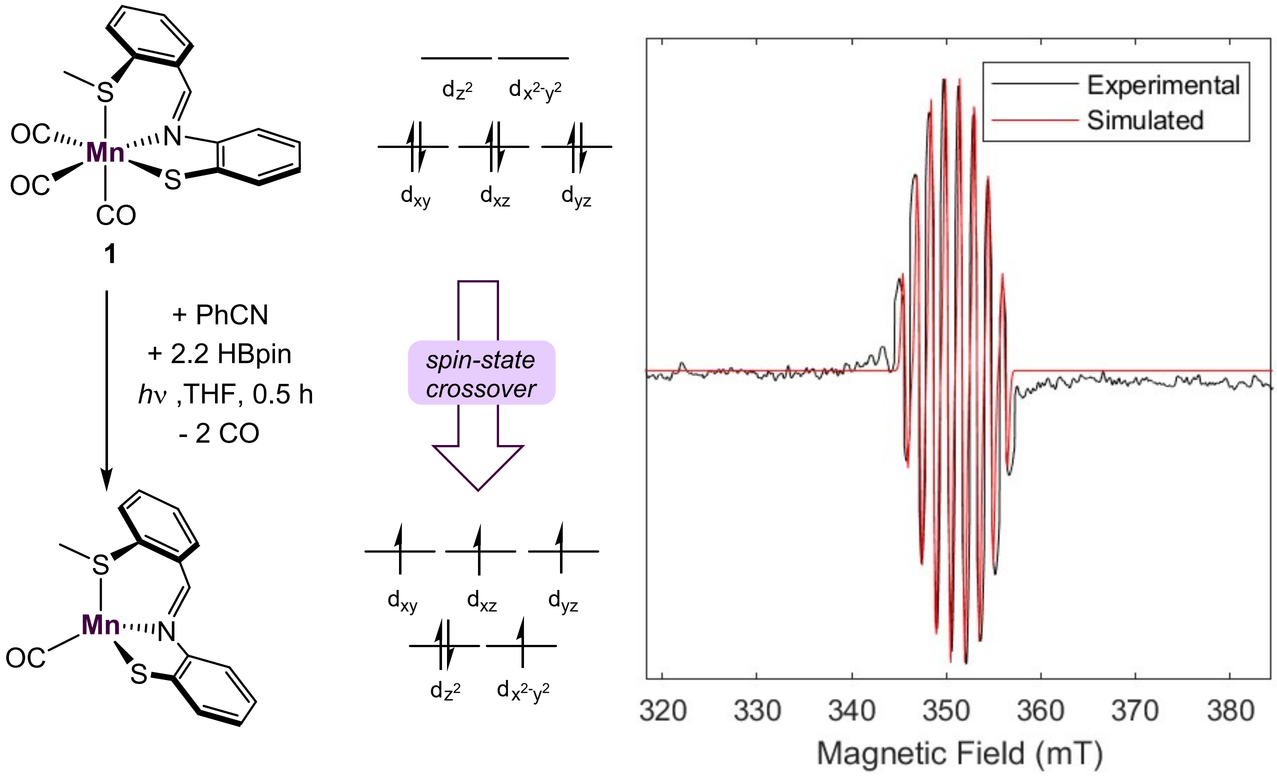

Figure S28. 298 K isotropic EPR spectrum of catalytic reaction mixture in THF after irradiation for 45 min.

VI. Crystallographic Details

Crystals of **1** were mounted on thin glass fibers using cyanoacrylate glue and cooled to 200 ± 2 K during data collection. The data were collected on a Bruker single-crystal diffractometer equipped with a sealed Mo tube source (wavelength 0.71073 Å) and APEX II CCD detector. The raw data collection and processing were performed with the Bruker APEX II software package.[5] Semi-empirical absorption correction based on equivalent reflections was applied.[6] Systematic absences and unit cell parameters were consistent with monoclinic $P2_1/n$ (#14) for **1**. The structure was solved by intrinsic phasing and refined with a full-matrix least-squares procedure based on F^2, using SHELXL.[7] All non-hydrogen atoms were refined anisotropically. The hydrogen atoms bonded to carbon atoms were placed in idealized positions.

Refinement details for Mn(κ^3-S^{Me}NS)(CO)$_3$ (1).

The structure was refined without additional restraints / constraints. No disorder was present.

Table S1. Crystal data and structure refinement for Mn(κ^3-S^{Me}NS)(CO)$_3$ (1).

Identification code	tb271_fin
Empirical formula	$C_{17}H_{12}MnNO_3S_2$
Formula weight	397.34
Temperature/K	200(2)
Crystal system	monoclinic
Space group	$P2_1/n$
a/Å	9.0462(6)
b/Å	18.5124(13)
c/Å	9.7604(7)
α/°	90
β/°	93.8678(19)
γ/°	90
Volume/Å^3	1630.8(2)
Z	4
ρ_{calc}g/cm^3	1.618
μ/mm^{-1}	1.080
F(000)	808.0
Crystal size/mm^3	0.390 × 0.180 × 0.100
Radiation	MoKα (λ = 0.71073)
2Θ range for data collection/°	4.4 to 56.604
Index ranges	$-12 \le h \le 11$, $-24 \le k \le 24$, $-13 \le l \le 12$
Reflections collected	19706
Independent reflections	4034 [R_{int} = 0.0407, R_{sigma} = 0.0347]
Data/restraints/parameters	4034/0/218

Goodness-of-fit on F^2	1.025
Final R indexes [I>=2σ (I)]	R_1 = 0.0326, wR_2 = 0.0732
Final R indexes [all data]	R_1 = 0.0525, wR_2 = 0.0835
Largest diff. peak/hole / e $Å^{-3}$	0.42/-0.30

Table S2. Bond Lengths for Mn(κ^3-S^{Me}NS)(CO)$_3$ (1).

Atom	Atom	Length/Å	Atom	Atom	Length/Å
C1	C2	1.395(3)	C11	C12	1.386(3)
C1	C6	1.402(3)	C12	C13	1.382(3)
C1	S1	1.754(2)	C13	S2	1.784(2)
C2	C3	1.386(3)	C14	S2	1.794(2)
C3	C4	1.379(3)	C15	O1	1.146(3)
C4	C5	1.386(3)	C15	Mn1	1.797(2)
C5	C6	1.390(3)	C16	O2	1.149(3)
C6	N1	1.440(2)	C16	Mn1	1.805(2)
C7	N1	1.285(3)	C17	O3	1.143(3)
C7	C8	1.461(3)	C17	Mn1	1.802(2)
C8	C9	1.390(3)	N1	Mn1	2.0587(16)
C8	C13	1.409(3)	S1	Mn1	2.3898(6)
C9	C10	1.383(3)	S2	Mn1	2.3592(6)
C10	C11	1.376(3)			

VII. DFT Calculations

All calculations were carried out using DFT[8] as implemented in the Jaguar 9.1 suite[9] of *ab initio* quantum chemistry programs. Geometry optimizations were performed with the B3LYP functional including Grimme's D3 dispersion correction.[10-15] The 6-31G** basis set was used for main group atoms and Mn was represented using the Los Alamos LACVP basis set that includes relativistic effective core potentials. The energies of optimized structures were reevaluated by additional single-point calculations on each optimized geometry using Dunning's correlation consistent triple-ζ basis set cc-pVTZ(-f)[16] which includes a double set of polarization functions. For Mn, we used a modified version of LACVP, designated as LACV3P, in which the exponents were decontracted to match the effective core potential with the triple-ζ quality. For boron, the 6-311G** basis set was used. Solvation energies were evaluated by a self-consistent reaction field (SCRF)[17-20] approach based on accurate numerical solutions of the Poisson-Boltzmann equation. In the results reported, solvation calculations were carried out at the same level of theory as the geometry optimization, employing a dielectric constant of ε = 2.284 for benzene. As is the case for all continuum models, the

solvation energies are subject to empirical parameterization of the atomic radii that are used to generate the solute surface. We employed the standard set of Van der Waals radii in Jaguar for H (1.150 Å), B(2.042 Å), C(1.900 Å), N(1.600 Å), O (1.600 Å), S(1.900 Å) and Mn (1.480 Å). Analytical vibrational frequencies within the harmonic approximation were computed with the 6-31G**/LACVP basis set to confirm proper convergence to well-defined minima or saddle points on the potential energy surface. The intermediates were confirmed with no vibrational frequency, while transition states showed a single imaginary frequency.

The energy components have been computed with the following protocol. The free energy in solution-phase, G(sol), has been calculated as follows, with T = 298 K to match the experimental room temperature conditions:

$G(\text{sol}) = G(\text{gas}) + G_{\text{solv}}$

$G(\text{gas}) = H(\text{gas}) - TS(\text{gas})$

$H(\text{gas}) = E(\text{SCF}) + \text{ZPE}$

$\Delta E(\text{SCF}) = \Sigma\ \text{E(SCF) for products} - \Sigma\ E(\text{SCF}) \text{ for reactants}$

$\Delta G(\text{sol}) = \Sigma\ G(\text{sol}) \text{ for products} - \Sigma\ G(\text{sol}) \text{ for reactants}$

Time dependent-density functional theory (TD-DFT) calculations were used to model dissociation of carbonyls upon UV light irradiation. Calculations were carried out with the Q-Chem 5.0 software. Using geometry obtained from the above method, TD-DFT calculations were carried out with the B3LYP-D3 functional and 6-31G**/LACVP basis set.

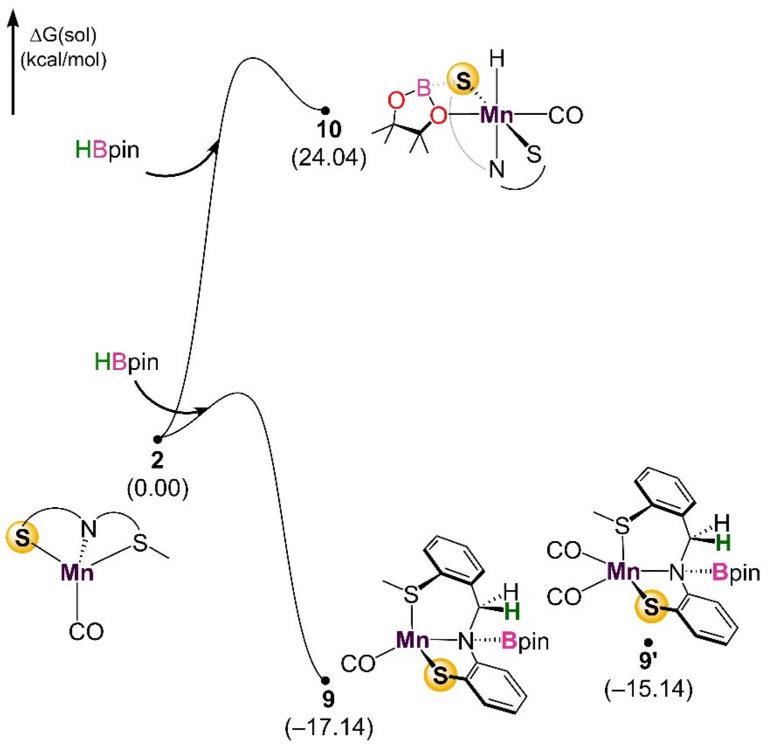

Figure S29. Thermodynamics of the off-cycle intermediate **9** and a proposed Mn–H species.

Two undesirable intermediates **9** and **10** are depicted in **Figure S29**. In accordance with the catalytic incapability of the off-cycle intermediate **9**, HBpin insertion to imine gives rise to a highly exergonic pathway. The intermediate **9** will be a thermodynamic sink considering further hydride transfer steps. Additional CO can bind to **9** resulting in **9`**, while the two energetically close intermediates are unable to participate in the catalytic cycle. Manganese hydride species, **10** is 23.8 kcal/mol higher in energy than **2**. Coordination of nitrile followed by a Bpin moiety transfer step will furnish a more unreasonable pathway than the proposed mechanism in terms of energy. We envision that the instability of the Manganese hydride species is attributed to the SNS ligand scaffold generating a weak ligand field. The catalytic pathway initiates with removing two COs which are strong field ligands. Regarding the Mn(I) center supported by the soft SNS ligand, the hydride moiety which contains dense electrons is placed in a deleterious environment.

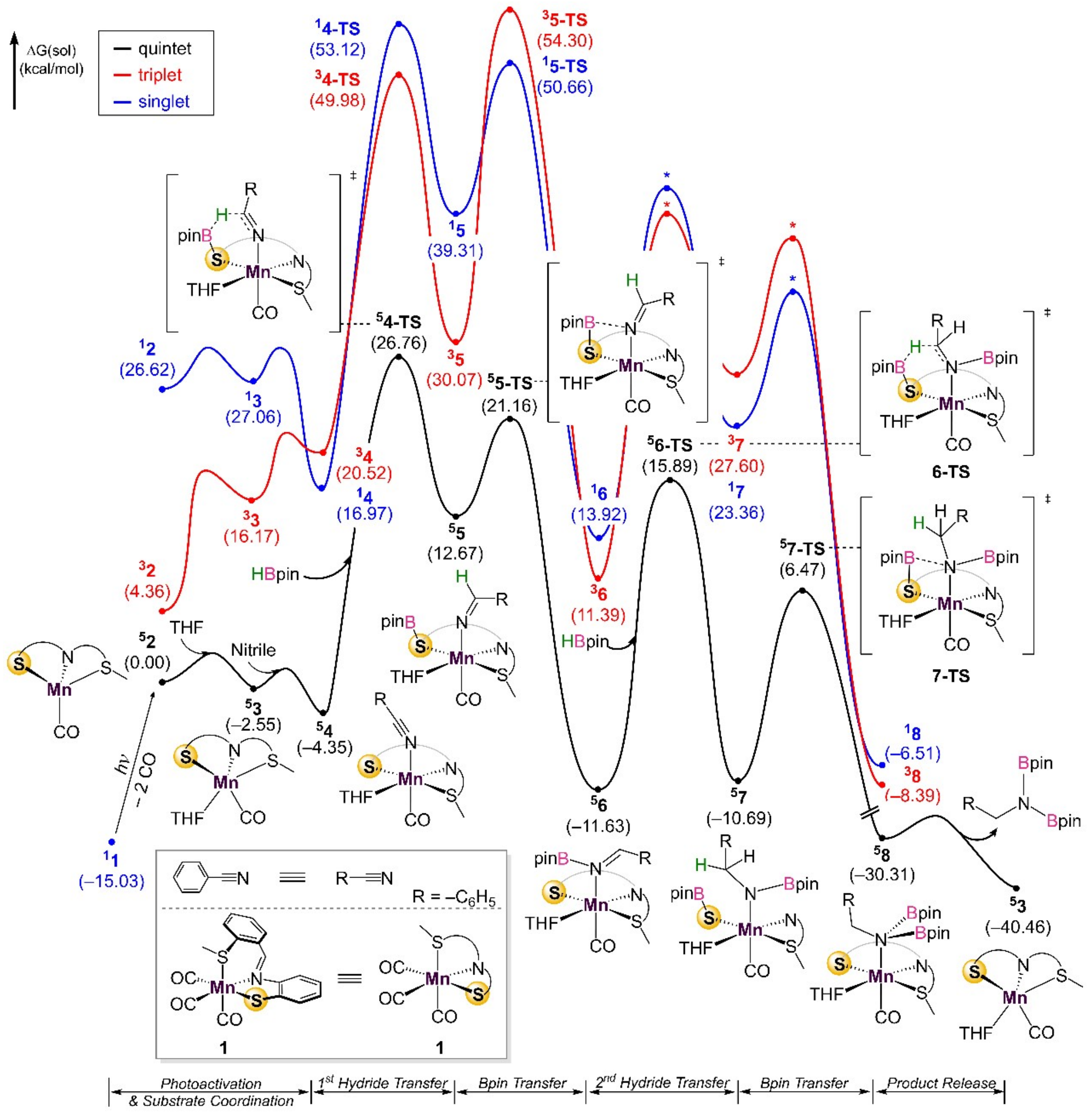

Figure S30. Energy profile including all spin states.

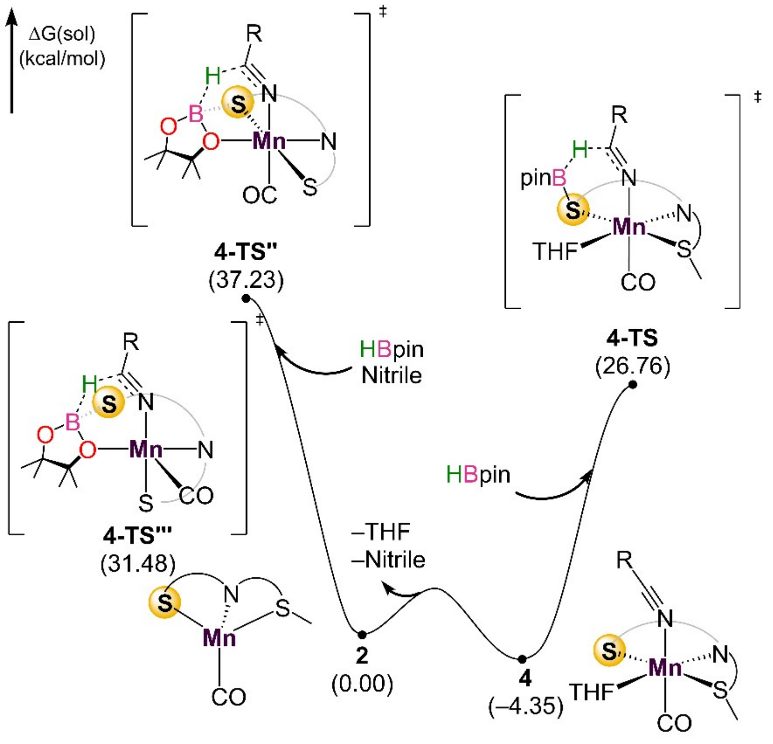

Figure S31. Energy profile for the hydride transfer in the presence and absence of coordinating THF.

The oxygen atom of HBpin can bind to the Mn center in lieu of THF as depicted in **4-TS``**. In the outer sphere mechanism traversing **4-TS**, the π-accepting CO ligand should be in the trans position to the transient amido moiety. In compliance with the formation of a sterically hindered Mn–S–B–O metallacycle in **4-TS``**, a conformer featuring a distinct position of the CO ligand trans to the thiolate moiety, **4-TS```** is favored by 5.8 kcal/mol. Due to the strong trans effect of the CO ligand, the thiolate moiety departs from the Mn center resulting in 3.6 Å of Mn–S distance. Relieved structural hindrance is reflected in the lower barrier of 35.8 kcal/mol, while the proposed pathway traversing **4-TS** having barrier of 31.1 kcal/mol is still favored. The amido species generated during hydride transfer in **4-TS```** is not stabilized by an electron-withdrawing ligand. Moreover, the Mn(I) species adopts a trigonal bipyramidal geometry giving rise to the 16-electron species as Mn–S bond cleaves, while **4-TS** affords 18-electron species comprising the stabilized amido moiety.

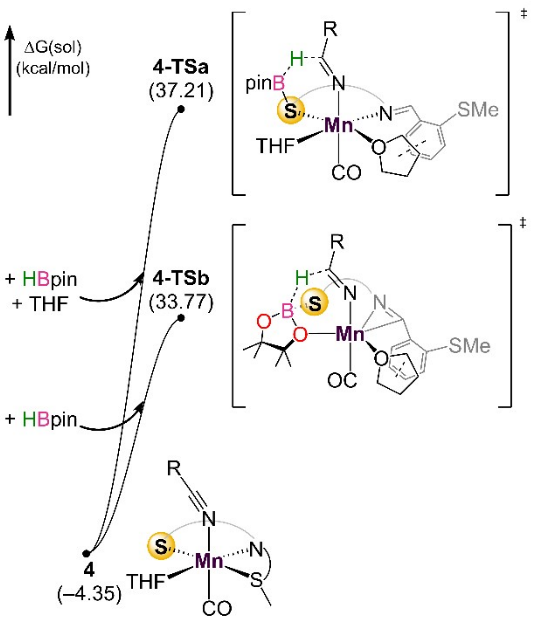

Figure S32. Possible pathways incorporating the cleavage of the thioether group.

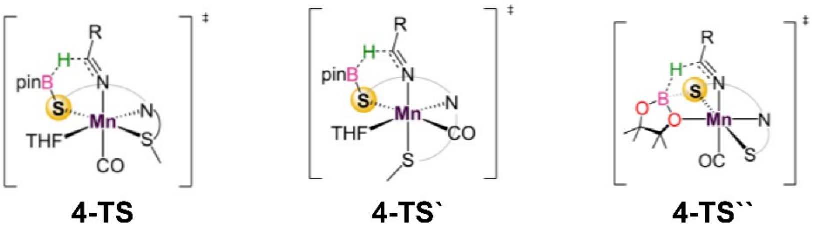

	B3LYP-D3	M06
**4-TS**	26.76	30.04
**4-TS`**	33.92	38.10
**4-TS``**	34.89	33.66

B3LYP-D3 & M06/cc-pVTZ(-f)//B3LYP-D3 & M06/LACVP**PCM (Ɛ = 7.6 for THF)
Energies in kcal•mol^{-1}
Referenced to [SNS]Mn(CO)(THF)

Figure S33. Comparison between the B3LYP-D3 functional and M06 functional.

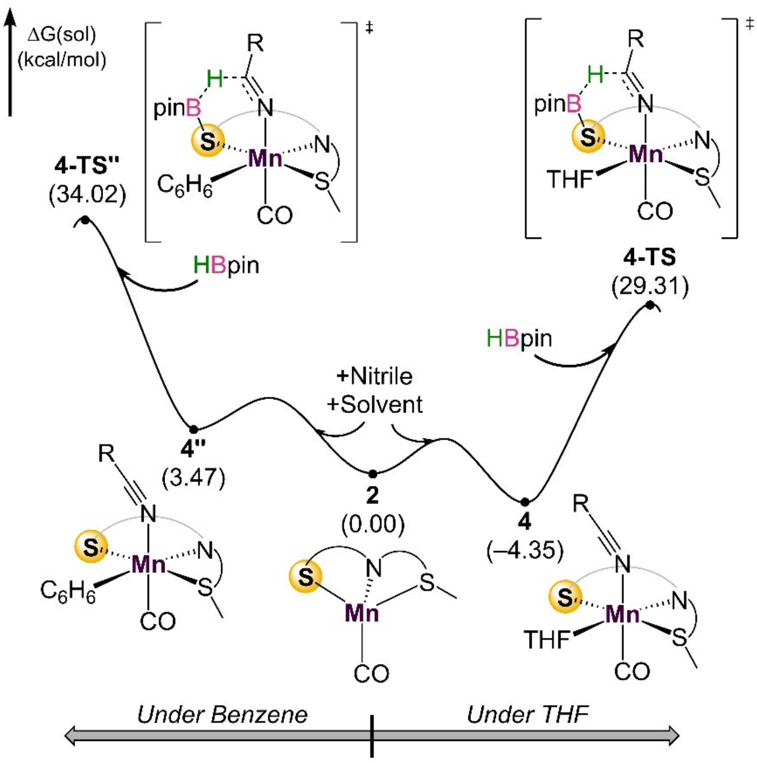

Figure S34. Comparison of the energy profiles for the reaction under THF and benzene as the solvent.

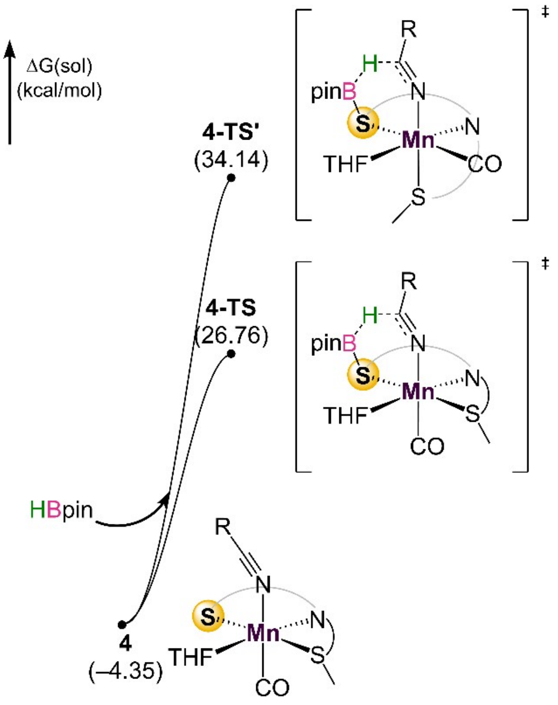

Figure S35. Comparison between *fac*- and *mer*-conformations in the turnover-limiting transition state.

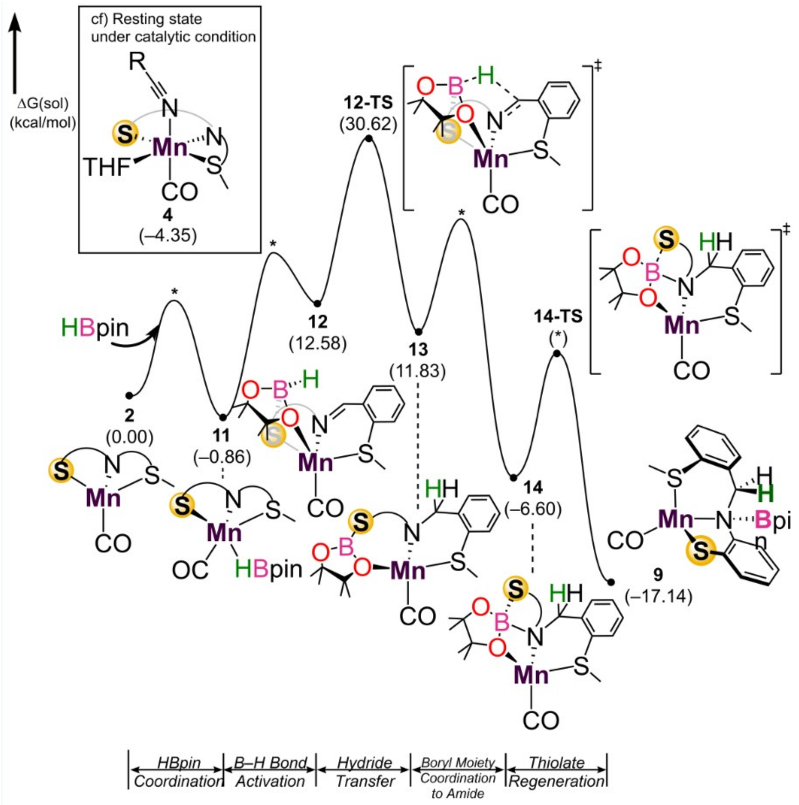

Figure S36. Off-cycle pathway in the absence of benzonitrile.

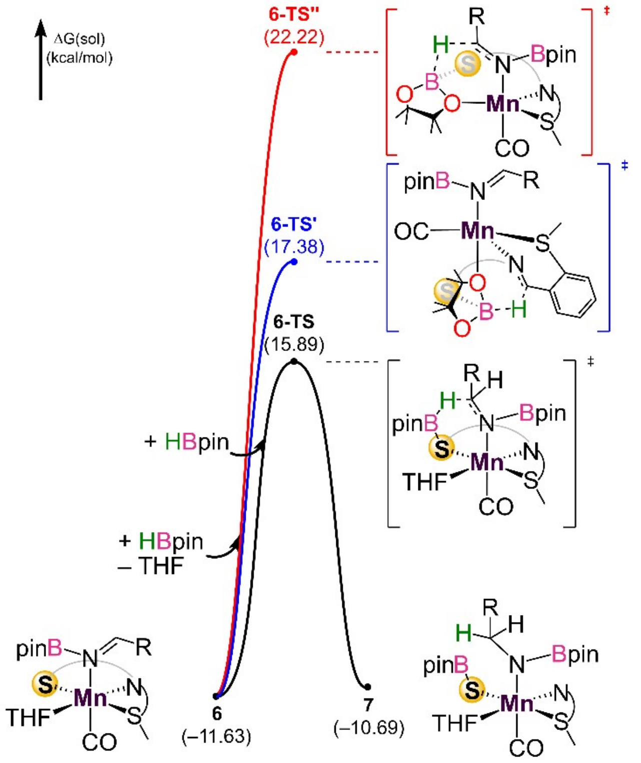

Figure S37. Comparison of transition states related to THF dissociation in intermediate **6**.

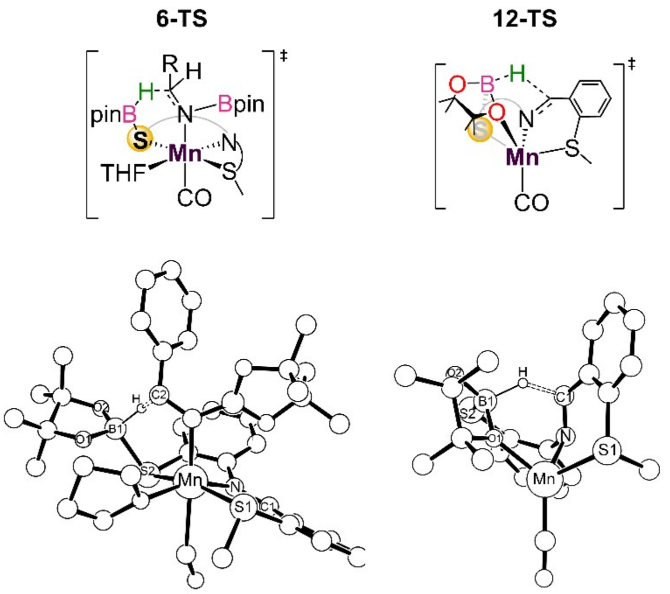

Figure S38. Comparison of 3D structures of **6-TS** and **12-TS**.

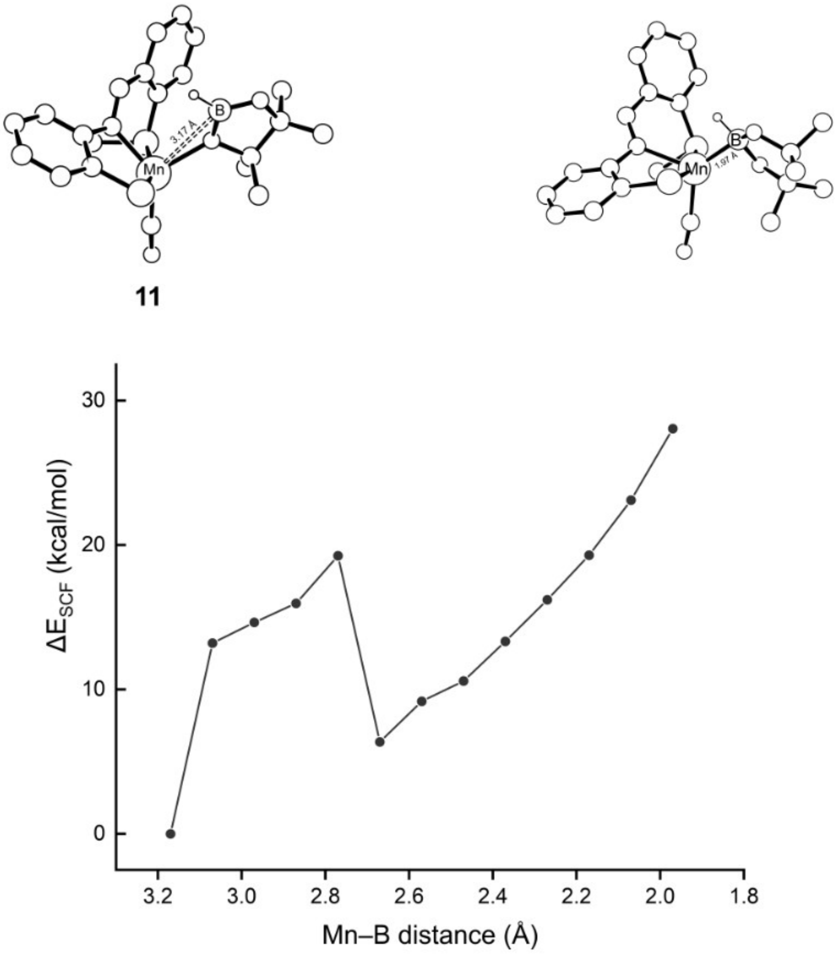

Figure S39. Scanning the potential energy surface of the Mn–B in **11**. Forced geometries are unstable and an adduct could not be optimized.

Table S3 Computed energy components for DFT optimized structures

	E_{SCF} (eV)	ZPE (kcal/mol)	S_{gas} (cal/mol·K)	G_{solv} (kcal/mol)
Label	B3LYP-D3/ cc-pVTZ(-f), LACV3P	B3LYP-D3/ 6-31G**, LACVP	B3LYP-D3/ 6-31G**, LACVP	B3LYP-D3/ 6-31G**, LACVP
Benzene	-6322.006	63.16	64.09	-2.30
Benzonitrile	-8832.844	62.36	77.13	-6.64
CO	-3084.527	3.15	47.24	0.00
Diborylamine (product)	-31259.999	308.87	175.52	-8.57
HBpin	-11211.936	120.07	90.99	-4.11
Off-cycle intermediate, 9	-55009.910	268.56	196.53	-13.45
Off-cycle intermediate, 9’	-58094.859	272.97	212.01	-12.46
Mn–H species, 10	-55007.832	261.43	199.82	-12.08
THF	-6327.710	73.37	72.17	-3.00
1	-49967.473	157.97	158.41	-12.77
2	-43796.262	144.76	152.64	-13.91
^{3}2	-43796.222	146.54	143.33	-15.03
^{1}2	-43795.191	146.38	140.66	-17.18
3	-50124.836	219.11	181.93	-10.96
^{3}3	-50124.270	220.76	171.66	-12.35
^{1}3	-50123.898	221.55	165.65	-12.62
4	-58958.301	281.25	229.29	-13.74
^{3}4	-58957.422	281.90	226.35	-13.01
^{1}4	-58957.827	285.05	206.11	-16.40
4’’	-58952.280	270.71	227.52	-12.64
4-TS	-70170.020	401.65	260.80	-9.74
^{3}4-TS	-63840.801	327.80	223.89	-9.73
^{1}4-TS	-63840.674	330.01	212.40	-15.16

4-TS'	-70169.685	401.79	256.36	-11.63
4-TS''	-70163.979	391.06	259.77	-9.51
4-TS'''	-63841.398	327.03	228.67	-12.06
4-TSa	-76497.964	476.08	291.76	-10.11
4-TSb	-70169.490	401.30	259.59	-14.83
5	-70170.604	403.47	271.63	-9.02
$^{3}5$	-70170.281	405.70	251.88	-9.54
$^{1}5$	-70169.978	406.46	244.90	-10.12
5-TS	-70170.458	403.60	255.16	-8.94
$^{3}5$-TS	-70169.428	406.29	241.56	-8.64
$^{1}5$-TS	-70169.560	406.28	240.76	-9.46
6	-70171.500	404.85	263.68	-16.42
$^{3}6$	-70170.759	405.50	264.45	-13.24
$^{1}6$	-70170.895	408.61	243.93	-16.81
6-TS	-81383.325	525.13	302.74	-11.26
6-TS’	-75054.799	449.65	273.71	-12.96
6-TS’’	-75054.763	450.32	264.63	-12.32
7	-81384.497	527.19	309.92	-10.73
$^{3}7$	-81383.115	527.29	298.63	-10.12
$^{1}7$	-81383.593	531.77	284.36	-12.07
7-TS	-81383.854	527.17	300.66	-11.14
8	-81385.333	529.09	306.50	-14.09
$^{3}8$	-81384.562	529.90	303.23	-13.97
$^{1}8$	-81384.644	532.29	290.02	-16.54
11	-55008.981	265.15	202.88	-13.51
12	-55008.567	265.26	198.82	-10.93

12-TS	-55007.865	264.64	190.96	-10.60
13	-55008.701	267.43	196.90	-11.13
14	-55009.578	268.29	190.56	-12.08

Table S4 Coordinates and vibrational frequencies for optimized structure

Coordinates

==============================

Benzene

================================

C1 2.850831 0.621417 2.515819
C2 1.541382 0.520834 2.990118
C3 3.355939 1.860838 2.117780
H4 1.148406 -0.443438 3.299794
H5 4.374694 1.939092 1.748774
C6 0.737040 1.659674 3.066378
C7 2.551596 2.999677 2.194039
H8 -0.281715 1.581419 3.435384
H9 2.944572 3.963950 1.884363
C10 1.242147 2.899094 2.668338
H11 0.616368 3.785113 2.727668
H12 3.476610 -0.264602 2.456489

================================

Benzonitrile

================================

C1 -1.356772 -0.000004 -3.530825
C2 -2.514299 -0.355166 -2.834338
C3 -0.199246 0.355152 -2.834328
H4 -3.413275 -0.631047 -3.376948
H5 0.699736 0.631035 -3.376931
C6 -2.521015 -0.357164 -1.441759
C7 -0.192562 0.357137 -1.441754
H8 -3.414855 -0.631366 -0.891723
H9 0.701272 0.631334 -0.891703
C10 -1.356802 -0.000016 -0.741197
H11 -1.356768 0.000004 -4.616845
C12 -1.356735 0.000006 0.693504
N13 -1.356608 0.000049 1.856712

================================

CO

================================

C1 -0.293499 0.001892 -2.165194
O2 -0.830929 0.125413 -3.161392

================================

Diborylamine

================================

C1 -5.331882 0.197436 -2.942489
C2 -5.378927 1.307495 -1.827688
O3 -3.977584 1.658762 -1.686571
C4 -6.108842 2.578547 -2.281694
C5 -5.905872 0.845054 -0.473550
O6 -4.183976 0.608677 -3.727321
C7 -6.553298 0.143099 -3.855053
C8 -5.017514 -1.196470 -2.384712
B9 -3.383559 1.366610 -2.892335
H10 -2.012096 0.410873 -4.883020
H11 -5.746888 2.906529 -3.260625
H12 -7.190572 2.425322 -2.341968
H13 -5.907392 3.375066 -1.560511
H14 -6.423249 -0.649871 -4.597280
H15 -7.458137 -0.073498 -3.277249
H16 -6.692971 1.083820 -4.389763
H17 -5.890820 1.681178 0.231333
H18 -6.937875 0.489088 -0.562191
H19 -5.291444 0.044089 -0.059362
H20 -4.150529 -1.162818 -1.718725
H21 -5.866311 -1.612892 -1.833851
H22 -4.781355 -1.864220 -3.217775
C23 -3.163293 4.437554 -6.485276
C24 -2.921726 4.100146 -7.819427
C25 -2.280319 2.892947 -8.115683
H26 -3.232086 4.765479 -8.620485
H27 -3.665476 5.370767 -6.240303
C28 -2.765256 3.580690 -5.455264
H29 -2.972505 3.848629 -4.421999
C30 -2.124811 2.371093 -5.741823
H31 -2.090186 2.613870 -9.151199
C32 -1.888209 2.039549 -7.085419
H33 -1.392659 1.099672 -7.325395
C34 -1.648312 1.417480 -4.653602
N35 -2.078862 1.785781 -3.298245
C36 -0.448725 4.066184 -0.952034
C37 0.354409 4.276680 -2.288241
O38 -1.621453 3.343421 -1.409054
C39 -0.904894 5.349882 -0.265002
C40 0.280816 3.157062 0.044506
O41 -0.027174 3.105566 -3.055660
C42 -0.116495 5.501753 -3.084219
C43 1.871473 4.291791 -2.135950
B44 -1.265321 2.714798 -2.581280
H45 -1.206253 5.510933 -3.178073
H46 0.202838 6.437463 -2.615031
H47 0.305478 5.451285 -4.091504
H48 2.338420 4.431002 -3.115333
H49 2.185413 5.115960 -1.486526
H50 2.239476 3.353972 -1.716377
H51 -1.458781 5.102744 0.645208
H52 -0.043434 5.966014 0.013909
H53 -1.561504 5.935333 -0.910763
H54 0.632974 2.245170 -0.446234
H55 1.137745 3.662349 0.500564
H56 -0.417687 2.869875 0.834955
H57 -0.554033 1.384387 -4.676394

================================

HBpin

================================

C1 1.732851 3.125565 2.270194
C2 1.678059 3.555808 3.786512
B3 1.521066 5.383667 2.441474
H4 1.417159 6.521531 2.113864
O5 1.304723 4.339117 1.585048
O6 1.866451 4.999959 3.708139
C7 3.153034 2.824263 1.775798
H8 3.840873 3.631171 2.042615
H9 3.533496 1.887314 2.192981
H10 3.137358 2.739934 0.685998
C11 2.780080 2.970677 4.665079

H12 2.667749 3.338863 5.688617
H13 2.716251 1.877657 4.685592
H14 3.771397 3.257599 4.311313
C15 0.787884 1.990831 1.885264
H16 0.885061 1.779129 0.816856
H17 1.034739 1.078713 2.438507
H18 -0.253111 2.250311 2.083459
C19 0.305220 3.330532 4.433076
H20 -0.493724 3.750811 3.816160
H21 0.104906 2.266684 4.589758
H22 0.283572 3.834958 5.402530
================================

Off-cycle intermediate, 9

================================

Mn1 -1.038075 -0.275055 -0.493579
C2 -2.886073 0.103722 -0.065677
O3 -4.004996 0.306791 0.191284
N4 0.310911 -1.346103 0.988567
C5 1.618770 -0.685438 0.992607
C6 1.983102 0.228719 -0.017674
C7 3.286398 0.764706 0.041190
C8 4.176402 0.419298 1.051185
C9 3.794828 -0.475836 2.055588
C10 2.514609 -1.019176 2.014362
H11 2.196621 -1.722970 2.781033
H12 4.479031 -0.741328 2.855577
H13 5.170989 0.857649 1.059606
H14 3.584554 1.470254 -0.728182
S15 0.906392 0.749833 -1.331910
C16 0.409230 -2.800323 0.605015
C17 1.123090 -3.024705 -0.709904
C18 0.481661 -2.901834 -1.957191
C19 1.190980 -3.114265 -3.142341
C20 2.551903 -3.413845 -3.100195
C21 3.205455 -3.520247 -1.874650
C22 2.486834 -3.334910 -0.695622
H23 2.994128 -3.399565 0.262421
H24 4.267261 -3.743192 -1.834966
H25 3.096052 -3.561604 -4.028637
H26 0.694665 -3.036373 -4.102824
S27 -1.264931 -2.455148 -1.990064
C28 -1.400718 -1.517566 -3.559083
H29 -2.350300 -0.981244 -3.503824
H30 -0.574383 -0.805650 -3.617488
H31 -1.409865 -2.188841 -4.419246
H32 0.943543 -3.341257 1.393858
C33 -1.154200 -0.142602 4.131200
C34 -1.949532 -1.488835 3.886975
O35 -1.182562 -2.115532 2.813629
C36 -1.967785 -2.446088 5.074201
C37 -3.368353 -1.275368 3.353606
O38 -0.513592 0.089742 2.846157
C39 -0.020298 -0.278287 5.153889
C40 -2.027491 1.063937 4.457517
B41 -0.470224 -1.116125 2.204455
H42 -0.609376 -3.195682 0.590878
H43 -0.959301 -2.742824 5.366831
H44 -2.460935 -1.981405 5.934063
H45 -2.524004 -3.349562 4.808917
H46 0.596117 0.623567 5.114946
H47 -0.404609 -0.397750 6.170960
H48 0.621453 -1.132417 4.918779
H49 -3.769132 -2.237901 3.023121
H50 -4.027736 -0.874848 4.129051
H51 -3.379122 -0.594419 2.501316
H52 -2.732105 1.273857 3.651350
H53 -2.588064 0.894829 5.382851
H54 -1.396494 1.946408 4.594602
================================

Off-cycle intermediate, 9'

================================

Mn	-0.875688358	-0.041342832	-0.643521367
C	-1.474169940	1.355516870	0.670474165
O	-1.716776656	2.260125491	1.336145510
N	0.748396042	-1.541970602	1.289364314
C	1.921937625	-0.698144251	1.349989643
C	2.265839628	0.205161556	0.319944309
C	3.440036136	0.967762990	0.490786765
C	4.240782610	0.844596016	1.620303974
C	3.892976024	-0.053155297	2.632856093
C	2.738358121	-0.814686021	2.483268244
H	2.442961535	-1.517133979	3.259232645
H	4.506370062	-0.154709966	3.523226790
H	5.135356103	1.455206042	1.711942493
H	3.710752257	1.670932834	-0.290865991
S	1.358316260	0.433890585	-1.191880937
C	0.999660949	-2.919047198	0.794272286
C	1.195952669	-3.008657783	-0.702650200
C	0.118200741	-2.891784174	-1.603174074
C	0.332826412	-2.936564964	-2.981294620
C	1.627127294	-3.099397351	-3.477389298
C	2.703303833	-3.213727969	-2.602682514
C	2.478701803	-3.170789244	-1.227307240
H	3.315888272	-3.240450297	-0.538230380
H	3.712635194	-3.325392789	-2.986727128
H	1.786585752	-3.124225011	-4.551491265
H	-0.487818795	-2.827313965	-3.679917951
S	-1.511463862	-2.578199956	-0.893844259
C	-2.631908471	-2.664373580	-2.330688126
H	-3.634887203	-2.496486321	-1.932658553
H	-2.402062917	-1.890574525	-3.064609487
H	-2.582781483	-3.655477613	-2.786018482
H	1.890549153	-3.317163741	1.296089011
C	-1.403747803	-0.508576513	4.049833620
C	-2.184750938	-1.730216787	3.434822111
O	-1.163980226	-2.361144569	2.615154267
C	-2.692187697	-2.747223466	4.450465779
C	-3.316537508	-1.310187110	2.488219338
O	-0.438328114	-0.207919630	3.006683086
C	-0.596083136	-0.881462443	5.298984350
C	-2.249376019	0.732262855	4.316617888
B	-0.253216603	-1.371894039	2.294119063
H	0.151016099	-3.536360677	1.101319159
H	-1.870625649	-3.180789276	5.023136779
H	-3.396972456	-2.278619207	5.145243113
H	-3.210233196	-3.559617383	3.932638695
H	0.089871056	-0.062105388	5.529753922
H	-1.243420395	-1.050576123	6.164594175

H -0.001525332 -1.783351666 5.125379971
H -3.668223470 -2.193056866 1.947104638
H -4.159117716 -0.873043196 3.031356336
H -2.961054852 -0.582597436 1.752664650
H -2.712727231 1.110837210 3.405628601
H -3.033555130 0.509526426 5.048033812
H -1.616886825 1.526557899 4.722849123
O -2.303558261 1.061502329 -3.251119758
C -1.860544582 0.638963582 -2.275199127

================================

Mn–H species, 10

================================

Mn1 -1.452986 -0.822408 -0.987755
C2 -1.424118 -3.383376 -0.844131
O3 -1.643035 -4.492815 -0.732747
N4 0.589123 -0.865196 -0.499679
C5 1.208622 0.054397 0.347620
C6 0.503375 0.763689 1.354168
C7 1.151063 1.706755 2.155769
C8 2.508261 1.981525 1.992937
C9 3.219079 1.299120 1.004049
C10 2.583205 0.361815 0.199170
H11 3.135554 -0.129099 -0.596135
H12 4.274318 1.507690 0.846918
H13 2.996535 2.717023 2.624646
H14 0.578675 2.220491 2.922351
S15 -1.243524 0.439728 1.711234
C16 1.361522 -1.901706 -0.931475
C17 1.185346 -2.699258 -2.096744
C18 0.362569 -2.417845 -3.244693
C19 0.237251 -3.349047 -4.281444
C20 0.908146 -4.569450 -4.250608
C21 1.765359 -4.843983 -3.169498
C22 1.904930 -3.936077 -2.140803
H23 2.557460 -4.170783 -1.303079
H24 2.319438 -5.778287 -3.138099
H25 0.786985 -5.281664 -5.060345
H26 -0.391568 -3.097693 -5.130968
S27 -0.527373 -0.875028 -3.471312
C28 0.838944 0.355387 -3.475715
H29 0.350300 1.347078 -3.793134
H30 1.291476 0.432550 -2.425869
H31 1.618029 0.022599 -4.263683
H32 2.191067 -2.198789 -0.286089
C33 -3.500274 2.887267 -0.591190
C34 -2.259670 2.635077 -1.533589
O35 -1.538531 1.572460 -0.814425
C36 -2.619988 2.116091 -2.920185
C37 -1.286018 3.811326 -1.615347
O38 -3.002552 2.456516 0.721705
C39 -4.700486 1.991396 -0.909910
C40 -3.920337 4.348188 -0.484076
B41 -1.980089 1.600668 0.496928
H42 -3.169962 -0.948457 -0.920250
H43 -3.112077 1.143507 -2.859574
H44 -3.283761 2.821547 -3.429964
H45 -1.717747 2.007422 -3.526625
H46 -5.438139 2.097312 -0.109659
H47 -5.171219 2.283568 -1.853452
H48 -4.401252 0.940141 -0.966369
H49 -0.371644 3.478714 -2.114477
H50 -1.713388 4.641289 -2.185141
H51 -1.015166 4.170283 -0.618774
H52 -3.113471 4.976740 -0.102679
H53 -4.226988 4.726357 -1.465113
H54 -4.771500 4.434936 0.196133

================================

THF

================================

H1 -0.970467 -0.001838 -3.396327
O2 0.386955 0.367352 -1.865922
C3 0.145626 -0.323432 -0.636131
C4 -1.327212 -0.756304 -0.663309
C5 -1.958525 0.378171 -1.484006
C6 -0.855536 0.651458 -2.516544
H7 0.840807 -1.168195 -0.560065
H8 0.340907 0.352010 0.212195
H9 -1.757754 -0.868401 0.335593
H10 -1.435846 -1.711810 -1.189407
H11 -2.119036 1.259854 -0.852778
H12 -2.914854 0.110269 -1.941598
H13 -0.843669 1.690071 -2.868432

================================

1

================================

Mn1 -0.009895 -0.007277 -0.021713
C2 1.284727 0.179190 1.234747
O3 2.105600 0.307977 2.031955
C4 -0.245064 1.783746 -0.104581
O5 -0.396920 2.924648 -0.206327
C6 -1.279145 -0.220139 1.245823
O7 -2.072422 -0.328572 2.081037
N8 0.391661 -2.032683 -0.307678
C9 1.784801 -2.324656 -0.428184
C10 2.562018 -1.351757 -1.097389
C11 3.921867 -1.641902 -1.310214
C12 4.483275 -2.827093 -0.846066
C13 3.708513 -3.761046 -0.145662
C14 2.355230 -3.508939 0.056005
H15 1.747561 -4.207205 0.625032
H16 4.159510 -4.664233 0.252934
H17 5.541623 -3.014907 -1.005790
H18 4.534816 -0.910009 -1.827131
S19 1.819020 0.154641 -1.627502
C20 -0.454420 -2.996707 -0.453718
C21 -1.914914 -2.856066 -0.468366
C22 -2.588609 -1.745118 -1.033076
C23 -3.983880 -1.714370 -1.032310
C24 -4.717147 -2.763298 -0.472611
C25 -4.065460 -3.872557 0.063406
C26 -2.674470 -3.918962 0.043336
H27 -2.153779 -4.782100 0.450146
H28 -4.633080 -4.695184 0.485956
H29 -5.801788 -2.711034 -0.470220
H30 -4.516849 -0.877848 -1.467478
S31 -1.590463 -0.460387 -1.808055
C32 -2.762248 0.932104 -1.927157
H33 -2.159415 1.793014 -2.221198

H34 -3.517651 0.743579 -2.691869
H35 -3.222850 1.128619 -0.956921
H36 -0.079233 -4.010728 -0.611155
================================
2
================================
Mn1 0.025025 -0.797409 0.603353
C2 -1.372434 -0.715737 1.962152
O3 -2.188727 -0.592090 2.779395
N4 0.586500 -2.275138 -0.837257
C5 1.964397 -2.367655 -1.124419
C6 2.834453 -1.423265 -0.509119
C7 4.210912 -1.525969 -0.795105
C8 4.706691 -2.491747 -1.659815
C9 3.837182 -3.393254 -2.289938
C10 2.478161 -3.322368 -2.023469
H11 1.799307 -3.987365 -2.548629
H12 4.216518 -4.126814 -2.994395
H13 5.773746 -2.533640 -1.860677
H14 4.885489 -0.818679 -0.323042
S15 2.289303 -0.141489 0.582522
C16 -0.254650 -3.238876 -1.062303
C17 -1.705013 -3.110250 -0.891747
C18 -2.444101 -1.894002 -0.992605
C19 -3.819527 -1.911925 -0.739902
C20 -4.477635 -3.096482 -0.407579
C21 -3.773618 -4.298952 -0.348409
C22 -2.407501 -4.295199 -0.599930
H23 -1.845957 -5.224207 -0.542829
H24 -4.282287 -5.226270 -0.106074
H25 -5.545433 -3.073791 -0.211740
H26 -4.400471 -1.001178 -0.810469
S27 -1.614791 -0.379366 -1.515239
C28 -2.813698 0.922732 -1.067616
H29 -2.272148 1.861265 -1.205480
H30 -3.690676 0.919105 -1.718246
H31 -3.100159 0.825174 -0.017939
H32 0.098969 -4.229610 -1.361774
================================
$^{3}2$
================================
Mn 0.070054014 -0.456076579 0.140849883
C -0.296538606 1.053400254 1.064813320
O -0.585126749 2.027301997 1.638639762
N 0.482411247 -2.268661345 -0.698938143
C 1.862094105 -2.550461547 -0.954236142
C 2.815040230 -1.736017335 -0.306887436
C 4.175711869 -2.032327732 -0.489360600
C 4.577801052 -3.066255768 -1.329910330
C 3.627833725 -3.825210541 -2.023135256
C 2.274233807 -3.562538160 -1.836864442
H 1.538583591 -4.115481302 -2.413149555
H 3.939003848 -4.598583985 -2.718500002
H 5.637341033 -3.263847707 -1.468688765
H 4.913920572 -1.422642196 0.022640374
S 2.282246184 -0.368698921 0.672203003
C -0.339767466 -3.280041617 -0.791186017
C -1.796248376 -3.310736370 -0.775429667
C -2.687474011 -2.207459663 -0.803196307
C -4.067474998 -2.426252641 -0.868171498
C -4.592932443 -3.717149215 -0.859468443
C -3.733489381 -4.816098609 -0.818547462
C -2.362831470 -4.606085044 -0.792998178
H -1.692062741 -5.460977251 -0.779034301
H -4.129189711 -5.826820314 -0.815355436
H -5.669027396 -3.858841293 -0.889548553
H -4.747511936 -1.584040316 -0.920744264
S -2.052574169 -0.531894050 -0.826852677
C -3.327173367 0.380039788 0.124405335
H -2.867524116 1.332315568 0.389841895
H -4.213105004 0.556253951 -0.487099709
H -3.580828411 -0.172004452 1.030603632
H 0.100900403 -4.269663531 -0.915055692
================================
$^{1}2$
================================
Mn 0.085546701 -0.329479090 0.033956827
C -0.677462913 -0.804581900 1.547009401
O -1.159740913 -1.137214797 2.556514660
N 0.508308852 -2.162205929 -0.617750073
C 1.881532003 -2.393090336 -0.908189547
C 2.812674484 -1.421350292 -0.464640891
C 4.175849831 -1.633777669 -0.743220957
C 4.600638497 -2.749578308 -1.452796834
C 3.669490649 -3.684198773 -1.927089344
C 2.319398131 -3.497429488 -1.663301727
H 1.595815368 -4.187986252 -2.084724927
H 3.993886558 -4.535035843 -2.518084447
H 5.659074878 -2.884746115 -1.658252904
H 4.894935488 -0.899613276 -0.393464176
S 2.280488299 0.009878331 0.419320191
C -0.326958704 -3.161973787 -0.635408979
C -1.783817860 -3.109629671 -0.608645071
C -2.600166800 -1.975786653 -0.866046126
C -3.988664101 -2.116873863 -0.914392279
C -4.597653255 -3.344637422 -0.652878167
C -3.813251005 -4.466369635 -0.383431324
C -2.430425555 -4.344590440 -0.386319413
H -1.814902552 -5.220718164 -0.199651302
H -4.274692607 -5.428163774 -0.183693099
H -5.680875209 -3.418832776 -0.664400952
H -4.612947529 -1.261246510 -1.144741326
S -1.842803063 -0.377129748 -1.209774364
C -3.066521777 0.779423815 -0.485126632
H -2.558571106 1.745382455 -0.442494487
H -3.955724103 0.872335779 -1.110916092
H -3.323925032 0.451561465 0.523283852
H 0.078384547 -4.173145163 -0.688318335
================================
3
================================
Mn1 -0.698809 -0.305275 0.595453
C2 -2.686290 -0.548225 0.948889
O3 -3.801989 -0.707985 1.215550
N4 0.695295 -1.919450 0.156151
C5 1.883587 -1.685317 0.899285
C6 2.248721 -0.336150 1.156308
C7 3.416101 -0.113116 1.908909

C8 4.186845 -1.167575 2.394468
C9 3.800070 -2.491650 2.159556
C10 2.650566 -2.741929 1.414642
H11 2.318626 -3.764183 1.254592
H12 4.378050 -3.317548 2.563132
H13 5.081212 -0.955527 2.974235
H14 3.708139 0.913292 2.109231
S15 1.293625 1.039452 0.555337
C16 0.680400 -2.881323 -0.721146
C17 -0.476148 -3.261468 -1.521098
C18 -1.455860 -2.373447 -2.041423
C19 -2.575064 -2.894348 -2.705960
C20 -2.734306 -4.266161 -2.883969
C21 -1.741002 -5.145211 -2.437908
C22 -0.626453 -4.640335 -1.782564
H23 0.140672 -5.320742 -1.420102
H24 -1.838095 -6.214227 -2.601175
H25 -3.610957 -4.644478 -3.401252
H26 -3.310775 -2.206191 -3.110443
S27 -1.300952 -0.581377 -2.029078
C28 0.322011 -0.317457 -2.841697
H29 0.388807 0.756671 -3.023798
H30 1.132547 -0.607903 -2.174060
H31 0.356909 -0.863308 -3.786310
H32 1.577577 -3.492020 -0.862652
H33 -0.630294 0.238156 4.480876
O34 -0.518452 -0.978944 2.795174
C35 -0.627822 -2.368218 3.192982
C36 0.064344 -2.474696 4.561171
C37 0.982335 -1.239666 4.578621
C38 0.136016 -0.212809 3.834399
H39 -1.687982 -2.641326 3.221969
H40 -0.129997 -2.964061 2.423072
H41 0.610394 -3.415880 4.669796
H42 -0.672140 -2.415265 5.370266
H43 1.902243 -1.433624 4.020173
H44 1.245093 -0.914822 5.589349
H45 0.703324 0.575104 3.335469
================================

$^{3}3$

================================

Mn	-0.809991432	-0.587127150	0.661844291
C	-2.356935233	-1.487816331	0.909335973
O	-3.355438021	-2.069964203	1.096104465
N	0.702935693	-2.051421188	0.149578698
C	1.890447468	-1.808215844	0.884636079
C	2.203591615	-0.446599665	1.145692596
C	3.382540748	-0.187842254	1.869360244
C	4.192279962	-1.222554739	2.333898985
C	3.850486305	-2.559108064	2.095127465
C	2.700413854	-2.845968653	1.362487624
H	2.402658070	-3.876064845	1.183829417
H	4.466683103	-3.365044488	2.481732316
H	5.088832802	-0.985921476	2.901038293
H	3.646762090	0.845930345	2.071684136
S	1.149433871	0.847580990	0.565413594
C	0.692613813	-2.924442355	-0.801218267
C	-0.483728321	-3.198918123	-1.629799293
C	-1.449926496	-2.233383721	-2.012817130
C	-2.574178206	-2.627270968	-2.744416928
C	-2.752227274	-3.958174688	-3.120389009
C	-1.780832570	-4.906805290	-2.797092941
C	-0.656617310	-4.521609692	-2.075070492
H	0.095809995	-5.261102668	-1.811707522
H	-1.899968072	-5.941421261	-3.104228683
H	-3.635351156	-4.245683557	-3.683021604
H	-3.306190168	-1.878186472	-3.028965462
S	-1.288056492	-0.466035892	-1.673355692
C	0.226385313	-0.075748180	-2.631504215
H	0.400289952	0.993378020	-2.508301504
H	1.079842959	-0.612361898	-2.219949057
H	0.056772364	-0.324921974	-3.681021361
H	1.591940143	-3.510615554	-1.021884957
H	-0.703213874	0.693237217	4.246528000
O	-0.458125637	-0.750788438	2.771635930
C	-0.557583894	-2.069931243	3.374261512
C	0.040953242	-1.934995028	4.781160362
C	0.938240952	-0.692168567	4.652347572
C	0.116571733	0.186932021	3.717930516
H	-1.607314424	-2.375268030	3.368354587
H	0.015618648	-2.754467500	2.743094148
H	0.589510476	-2.833232136	5.077336085
H	-0.748798222	-1.760033914	5.520151307
H	1.889335306	-0.947106137	4.174846284
H	1.142916470	-0.208773715	5.611867697
H	0.686835591	0.911650578	3.137975376

================================

$^{1}3$

================================

Mn	-0.739963744	-0.610489471	0.644402062
C	-2.169355243	-1.669145136	0.900081955
O	-3.100597883	-2.343013211	1.104997629
N	0.592142399	-1.955341477	0.173481945
C	1.799617408	-1.744597933	0.909294900
C	2.172403011	-0.393138712	1.127305315
C	3.367735869	-0.162704387	1.833001778
C	4.134140538	-1.218332977	2.323252772
C	3.728808899	-2.544616770	2.131964410
C	2.560460576	-2.802930754	1.416546826
H	2.214170958	-3.823588780	1.277363951
H	4.309925561	-3.365067017	2.541414478
H	5.046273100	-1.005450112	2.874765336
H	3.677777170	0.864265169	2.001755054
S	1.143435987	0.906078387	0.526823306
C	0.571974130	-2.877529832	-0.743334819
C	-0.566573739	-3.150715275	-1.617978897
C	-1.492962571	-2.169245412	-2.052011264
C	-2.559728631	-2.528529998	-2.875872696
C	-2.724391122	-3.850862135	-3.292660111
C	-1.794033596	-4.817889129	-2.910534592
C	-0.722531643	-4.464040331	-2.096133683
H	0.000290219	-5.218606278	-1.795430053
H	-1.903999536	-5.845638049	-3.243563355
H	-3.566962237	-4.118024488	-3.923489456
H	-3.261401657	-1.763242283	-3.193018503
S	-1.321207537	-0.428734604	-1.605336518
C	0.140782328	-0.002867016	-2.631860543
H	0.347917456	1.052523667	-2.455041963

H 1.003168480 -0.583873466 -2.305159314
H -0.093719921 -0.184936302 -3.682716282
H 1.460966981 -3.491669341 -0.897170689
H -0.734107405 0.747248325 4.206418511
O -0.399740814 -0.720635255 2.769659697
C -0.474618101 -2.014633212 3.425211042
C 0.129141437 -1.819995585 4.824561605
C 0.969139238 -0.541486271 4.661685478
C 0.114070370 0.268560118 3.695333229
H -1.519380091 -2.337329035 3.442548032
H 0.102225772 -2.713602920 2.814636707
H 0.720662588 -2.685200557 5.135884332
H -0.661441433 -1.665260310 5.567257634
H 1.932988176 -0.767819879 4.195803827
H 1.146247272 -0.019892430 5.606616878
H 0.653005071 1.006456683 3.101943803

================================

4"

================================

Mn1 1.626446 1.096149 -0.003494
N2 0.790107 0.543544 -1.807433
C3 0.501123 1.672248 -2.609018
C4 0.240737 2.926164 -1.985018
C5 -0.029036 4.034375 -2.802277
C6 -0.043123 3.934340 -4.193480
C7 0.228109 2.707788 -4.801302
C8 0.498580 1.591793 -4.012559
H9 0.741684 0.642152 -4.480870
H10 0.238700 2.618488 -5.884298
H11 -0.261672 4.811499 -4.796790
H12 -0.249552 4.985365 -2.324881
S13 0.249654 3.105211 -0.208766
C14 0.292307 -0.641707 -2.201807
C15 0.484115 -1.913864 -1.585853
C16 1.456467 -2.292084 -0.595375
C17 1.415904 -3.563142 -0.005175
C18 0.458960 -4.506285 -0.364096
C19 -0.460459 -4.184401 -1.379612
C20 -0.436478 -2.938437 -1.969675
H21 -1.167935 -2.697767 -2.737791
H22 -1.198407 -4.915493 -1.699471
H23 0.442800 -5.481119 0.113101
H24 2.169726 -3.813449 0.736365
S25 2.837333 -1.258059 -0.103202
C26 3.722719 -1.026257 -1.693988
H27 4.585656 -0.392843 -1.481224
H28 4.042935 -1.997006 -2.076629
H29 3.064421 -0.520584 -2.400641
H30 -0.444623 -0.625097 -3.006607
N31 3.594639 2.110223 -0.550741
C32 3.843399 3.187425 -0.916791
C33 4.034168 4.528116 -1.359191
C34 2.914051 5.380151 -1.360323
C35 5.294537 4.979496 -1.785535
C36 3.071682 6.691481 -1.796890
C37 5.429783 6.294986 -2.216738
C38 4.321829 7.148547 -2.222813
H39 1.956820 4.996069 -1.018617
H40 6.144211 4.304879 -1.778107
H41 2.214004 7.357004 -1.806054
H42 6.398361 6.655577 -2.549000
H43 4.434869 8.173918 -2.562978
C44 -0.126356 0.040549 1.155312
O45 -1.158843 -0.346200 1.430704
C46 2.617455 0.054030 3.270884
C47 1.444162 0.005028 4.023504
C48 2.951941 1.223728 2.576655
H49 1.179183 -0.902521 4.558810
H50 3.867578 1.277228 1.995711
C51 0.600925 1.120935 4.082469
C52 2.105366 2.337636 2.632111
H53 -0.317637 1.074711 4.660781
H54 2.354326 3.237034 2.078561
C55 0.925627 2.283357 3.383511
H56 0.260862 3.141136 3.406055
H57 3.265934 -0.814590 3.209590

================================

4-TS'''

================================

Mn -1.865365146 -2.946136580 -0.007813434
C -3.918122067 -3.400660930 -0.208476781
O -5.005527086 -3.657919388 -0.446624211
N -0.784077185 -2.364233241 -1.640685549
C 0.353482305 -3.151663163 -1.908481352
C 1.477938818 -3.103744900 -1.035494880
C 2.577825285 -3.927729657 -1.300090702
C 2.616760849 -4.771747358 -2.409370632
C 1.542330103 -4.775855665 -3.296797854
C 0.434305688 -3.972004841 -3.051759155
H -0.376537625 -3.962554045 -3.771572256
H 1.564538426 -5.398321259 -4.187539732
H 3.483005226 -5.403070405 -2.583463876
H 3.415918906 -3.896618745 -0.611633534
S 1.621532212 -1.986952012 0.366954987
C -1.962638866 -2.618943480 -2.257982049
C -2.925333299 -1.559432702 -2.634128001
C -3.281957327 -0.439106818 -1.842239853
C -4.202970492 0.498362789 -2.320374084
C -4.796598689 0.349043942 -3.574016209
C -4.475768207 -0.762121923 -4.353977149
C -3.558097545 -1.698074459 -3.884115129
H -3.304848509 -2.555747996 -4.502360197
H -4.936569950 -0.900394252 -5.327911656
H -5.511184644 1.086175291 -3.927890238
H -4.468024355 1.340746122 -1.688446600
S -2.724917857 -0.262956938 -0.141815639
C -1.083504151 0.538742064 -0.328222663
H -0.707635141 0.705864777 0.683111625
H -0.414447373 -0.137932186 -0.857751710
H -1.205817281 1.492928847 -0.844236678
H -2.091177999 -3.548099248 -2.820645057
C -0.005532555 -2.035507765 3.873832962
C -1.311528620 -2.470770698 3.124732598
O -0.840812822 -2.423682771 1.741500433
C -1.742861318 -3.905258512 3.449347776
C -2.495952035 -1.520397952 3.257662053
O 1.039670995 -2.627268040 3.078692805
C 0.099462319 -2.565659060 5.301365929

C 0.203913537 -0.514973078 3.846463253
B 0.580693898 -2.794321879 1.755801340
H 0.564263071 -4.103932205 1.397370875
H -0.900286540 -4.597340389 3.398216974
H -2.185301637 -3.962433419 4.447845971
H -2.486909986 -4.232192665 2.717230394
H 1.028956712 -2.212090424 5.756261653
H -0.737969567 -2.210325189 5.911921807
H 0.109905566 -3.657013132 5.318429531
H -3.338758217 -1.894364798 2.666808773
H -2.815259149 -1.452418577 4.302617897
H -2.253901840 -0.519767053 2.897330730
H 0.102419356 -0.133915972 2.826290736
H -0.505225658 0.009813738 4.494490028
H 1.218554064 -0.296913519 4.188976663
C 2.595399804 -8.414596948 1.210352876
C 1.515999840 -8.513749386 0.326360339
C 2.793768997 -7.244919875 1.947120297
H 3.281902766 -9.249447957 1.321227393
H 1.365339198 -9.421613111 -0.251186850
C 0.633948794 -7.448594794 0.182059995
H 3.633357447 -7.168646177 2.631731858
C 1.918627832 -6.169447294 1.802954252
H -0.209502661 -7.499603731 -0.498866289
C 0.834980305 -6.273639893 0.924668489
H 2.063668535 -5.249322945 2.362081485
C -0.112934389 -5.174379104 0.746450279
N -1.184423363 -4.937142918 0.247100150

================================

4-TS''

================================

Mn1 1.716427 0.850060 0.214219
N2 0.625647 0.446990 -1.517467
C3 0.252970 1.642056 -2.163962
C4 -0.079280 2.788475 -1.397916
C5 -0.365916 4.000487 -2.033714
C6 -0.341840 4.092630 -3.426074
C7 -0.034089 2.965314 -4.189394
C8 0.259947 1.755869 -3.566222
H9 0.542074 0.889631 -4.157148
H10 -0.008101 3.028710 -5.274009
H11 -0.564463 5.040253 -3.908051
H12 -0.606313 4.866801 -1.426484
S13 -0.135886 2.659116 0.393338
B14 0.889473 4.272326 0.963933
O15 0.298301 5.528044 0.654013
C16 0.424882 6.358601 1.830220
C17 0.456884 5.293153 2.982551
O18 1.163973 4.201459 2.355840
C19 -0.947226 4.797009 3.360740
H20 -1.510996 4.505874 2.470743
H21 -0.854677 3.917135 4.004850
H22 -1.513419 5.559863 3.904440
C23 1.226111 5.709419 4.233353
H24 1.187767 4.905362 4.975150
H25 2.275373 5.906748 4.006267
H26 0.785949 6.606453 4.682278
C27 1.741809 7.141853 1.726847
H28 1.862840 7.841477 2.559712
H29 2.599189 6.463695 1.710178
H30 1.745142 7.713550 0.794500
C31 -0.759784 7.319572 1.871677
H32 -1.706511 6.783292 1.787401
H33 -0.760616 7.895947 2.803450
H34 -0.694035 8.022727 1.035848
C35 0.194656 -0.709546 -2.052115
C36 0.562455 -2.030007 -1.659339
C37 1.696531 -2.436312 -0.876239
C38 1.846011 -3.773013 -0.481452
C39 0.925558 -4.749483 -0.848019
C40 -0.153779 -4.388641 -1.675227
C41 -0.316843 -3.077522 -2.071364
H42 -1.167899 -2.808395 -2.692743
H43 -0.867283 -5.141061 -2.000919
H44 1.058434 -5.777190 -0.524381
H45 2.718360 -4.045653 0.106301
S46 3.042289 -1.339974 -0.424913
C47 3.663257 -0.771361 -2.055552
H48 4.413946 -0.007409 -1.843727
H49 4.100895 -1.610899 -2.598870
H50 2.844975 -0.320832 -2.616565
H51 -0.605459 -0.645864 -2.791886
H52 1.963150 4.048265 0.237716
N53 3.200131 2.209650 -0.298330
C54 3.160940 3.401098 -0.411991
C55 3.866269 4.552039 -0.957858
C56 3.222313 5.776283 -1.172771
C57 5.221583 4.389944 -1.294180
C58 3.940362 6.837678 -1.721315
C59 5.929120 5.459100 -1.832297
C60 5.289523 6.684069 -2.046232
H61 2.175817 5.883214 -0.907727
H62 5.696428 3.428762 -1.123841
H63 3.444630 7.788720 -1.892163
H64 6.978662 5.338610 -2.085188
H65 5.845455 7.517414 -2.466506
C66 0.275981 -0.508050 1.440332
O67 -0.647394 -1.089228 1.756683
C68 2.656046 1.535559 2.892523
C69 3.446447 0.422648 2.583465
C70 3.161764 -0.821687 3.159806
H71 4.273260 0.526934 1.886923
H72 2.861657 2.505108 2.454088
C73 1.581671 1.404863 3.781293
H74 3.764102 -1.687980 2.902391
C75 2.095707 -0.946072 4.051284
H76 0.967747 2.275535 3.981455
C77 1.306902 0.168151 4.363641
H78 1.870255 -1.911300 4.496621
H79 0.470322 0.062563 5.049081

================================

4-TS'

================================

Mn1 0.678655 1.104300 0.432616
N2 -0.305527 0.484752 -1.308233
C3 -0.324049 1.593743 -2.183670
C4 -0.670525 2.869063 -1.667955
C5 -0.677916 3.989239 -2.504179

C6 -0.335292 3.871372 -3.851944
C7 0.027794 2.625173 -4.366657
C8 0.034589 1.503498 -3.542036
H9 0.358806 0.545094 -3.935429
H10 0.319675 2.525724 -5.408740
H11 -0.342863 4.750646 -4.489652
H12 -0.949268 4.953815 -2.087397
S13 -1.022571 3.046164 0.090226
B14 0.083263 4.626716 0.529101
O15 -0.419300 5.885897 0.115791
C16 -0.128489 6.828469 1.169755
C17 -0.106359 5.906453 2.441887
O18 0.417880 4.669951 1.911942
C19 -1.510865 5.615440 2.989640
H20 -2.174491 5.275044 2.190092
H21 -1.441489 4.818207 3.736656
H22 -1.954230 6.494442 3.467921
C23 0.809964 6.389486 3.564019
H24 0.753413 5.703630 4.414750
H25 1.850241 6.434931 3.236095
H26 0.508029 7.383062 3.912462
C27 1.240326 7.465728 0.888170
H28 1.484041 8.241885 1.620362
H29 2.033711 6.713873 0.895207
H30 1.221721 7.924110 -0.104362
C31 -1.216155 7.899019 1.174098
H32 -2.210495 7.451255 1.215781
H33 -1.094331 8.573841 2.028814
H34 -1.151733 8.493750 0.258061
C35 -0.704284 -0.696520 -1.808228
C36 -0.812061 -1.960442 -1.148464
C37 -0.767738 -2.257310 0.254134
C38 -0.883584 -3.580973 0.700976
C39 -1.022575 -4.647666 -0.181560
C40 -1.099483 -4.382693 -1.558939
C41 -1.010607 -3.083295 -2.012657
H42 -1.064111 -2.888257 -3.081123
H43 -1.229395 -5.195216 -2.268422
H44 -1.093408 -5.662560 0.197556
H45 -0.878317 -3.784819 1.767218
S46 -0.707601 -0.965728 1.510279
C47 0.455877 -1.697434 2.731465
H48 -0.064671 -2.361358 3.423390
H49 1.237722 -2.248104 2.204253
H50 0.901636 -0.861774 3.271140
H51 -1.005887 -0.716976 -2.855534
H52 1.158454 4.270760 -0.188199
C53 1.233765 2.579827 4.601724
C54 2.511084 2.918419 3.814397
C55 2.647521 1.700547 2.908192
H56 3.387406 3.067231 4.451837
H57 2.342333 3.808678 3.203221
C58 0.350591 1.937481 3.529865
H59 1.453407 1.867525 5.406003
H60 0.756259 3.457637 5.044321
O61 1.278878 1.341821 2.570569
H62 -0.299858 1.141515 3.906085
H63 -0.236041 2.683295 2.992314
H64 3.109152 0.847320 3.424355
H65 3.170405 1.891203 1.970017
N66 2.280973 2.336774 -0.242200
C67 2.297759 3.493029 -0.572980
C68 3.114080 4.458374 -1.308076
C69 2.574129 5.660082 -1.779859
C70 4.455744 4.134790 -1.572036
C71 3.375602 6.536463 -2.509669
C72 5.250008 5.019025 -2.294766
C73 4.711384 6.221096 -2.765108
H74 1.535688 5.891748 -1.572234
H75 4.850351 3.191451 -1.207525
H76 2.955630 7.467387 -2.879724
H77 6.288695 4.771295 -2.495589
H78 5.333751 6.908383 -3.331555
C79 2.248223 -0.716466 0.001237
O80 2.663570 -1.544037 -0.671763
=================================
4-TS
=================================
Mn1 1.385144 0.869650 0.393029
N2 0.472577 0.428750 -1.447391
C3 0.166555 1.603012 -2.156762
C4 -0.240208 2.771118 -1.462384
C5 -0.464850 3.965471 -2.152949
C6 -0.306289 4.023343 -3.538056
C7 0.073540 2.875348 -4.236093
C8 0.306454 1.683789 -3.556034
H9 0.644946 0.803936 -4.095051
H10 0.204526 2.909693 -5.314582
H11 -0.480792 4.957358 -4.064360
H12 -0.764403 4.845815 -1.593434
S13 -0.464935 2.703089 0.319385
B14 0.577125 4.282956 0.909950
O15 0.018292 5.550100 0.596619
C16 0.214948 6.396498 1.749104
C17 0.230441 5.353366 2.922832
O18 0.862669 4.211955 2.303051
C19 -1.181265 4.928923 3.353497
H20 -1.781680 4.643956 2.485408
H21 -1.103978 4.059787 4.014479
H22 -1.699632 5.726248 3.895410
C23 1.052986 5.770015 4.139259
H24 0.991654 4.999123 4.913430
H25 2.104666 5.905174 3.879894
H26 0.672292 6.704974 4.564374
C27 1.561567 7.119025 1.595189
H28 1.734022 7.831548 2.407791
H29 2.389206 6.405191 1.571567
H30 1.565337 7.668168 0.649729
C31 -0.924180 7.410693 1.803722
H32 -1.895892 6.916423 1.753837
H33 -0.874237 8.003570 2.723812
H34 -0.849369 8.094912 0.953103
C35 0.085974 -0.743766 -1.996098
C36 0.390336 -2.061264 -1.550497
C37 1.403814 -2.480174 -0.617496
C38 1.480315 -3.816798 -0.202479
C39 0.606763 -4.786198 -0.685582
C40 -0.346319 -4.416712 -1.653915

C41 -0.437505 -3.106299 -2.072447
H42 -1.193814 -2.831351 -2.804198
H43 -1.018529 -5.161839 -2.071028
H44 0.682213 -5.812821 -0.341188
H45 2.260080 -4.095184 0.501394
S46 2.684723 -1.398502 0.019375
C47 3.546626 -0.865886 -1.512909
H48 4.259056 -0.095181 -1.212954
H49 4.055371 -1.718624 -1.966296
H50 2.820035 -0.428714 -2.197692
H51 -0.636664 -0.687665 -2.812464
H52 1.659556 4.036493 0.189023
C53 1.714114 1.856310 4.719619
C54 3.000160 2.319064 4.013907
C55 3.214008 1.197668 3.003687
H56 3.847394 2.440375 4.694864
H57 2.812458 3.256659 3.484019
C58 0.900864 1.250512 3.570160
H59 1.941099 1.098180 5.477920
H60 1.174837 2.673833 5.204642
O61 1.875934 0.856159 2.559234
H62 0.329900 0.362693 3.859423
H63 0.242704 1.990321 3.111476
H64 3.669351 0.307801 3.459168
H65 3.781382 1.488108 2.117756
N66 2.945956 2.200983 -0.116439
C67 2.871991 3.380527 -0.325109
C68 3.578484 4.493497 -0.951463
C69 2.921065 5.681199 -1.293084
C70 4.943146 4.327191 -1.243310
C71 3.633051 6.700907 -1.922990
C72 5.645563 5.354669 -1.863428
C73 4.991456 6.542848 -2.204403
H74 1.867206 5.793131 -1.062170
H75 5.428360 3.393284 -0.977607
H76 3.125057 7.622020 -2.193640
H77 6.702008 5.230006 -2.083720
H78 5.542557 7.343163 -2.690391
C79 -0.493040 -0.588633 1.266786
O80 -1.494624 -1.120376 1.317681

===============================

[3]4-TS

===============================

Mn	-1.178217636	-1.030557521	-1.158373220
C	-1.632711728	-2.768484144	-0.190650377
O	-1.876656986	-3.703539504	0.426430611
N	0.713007402	-0.976447470	-0.661094771
C	1.181231126	0.143283844	0.059869800
C	0.252860566	0.978864131	0.718993984
C	0.682020279	2.073952788	1.469147362
C	2.041781601	2.377494807	1.560343159
C	2.967287787	1.587311931	0.874592730
C	2.545377949	0.489962645	0.127180494
H	3.270286019	-0.085429111	-0.438002442
H	4.025293834	1.831584412	0.909213230
H	2.370131505	3.233165882	2.142641461
H	-0.060211372	2.697633124	1.956773920
S	-1.488043246	0.640341933	0.534787583
C	1.592870911	-1.988480261	-0.870207010
C	1.492658571	-3.055506994	-1.808684352
C	0.596499775	-3.138889892	-2.920996944
C	0.597378481	-4.248566758	-3.767127447
C	1.476713022	-5.311772732	-3.560952030
C	2.389273510	-5.240787143	-2.497539815
C	2.399486863	-4.144602518	-1.654574231
H	3.107590662	-4.107755595	-0.830247959
H	3.092573770	-6.051892097	-2.329207289
H	1.458227874	-6.172988233	-4.221228350
H	-0.098967094	-4.271443771	-4.600641547
S	-0.543374099	-1.817301347	-3.320558086
C	0.591973334	-0.489821463	-3.885693165
H	-0.015952494	0.414254793	-3.925856095
H	1.383310288	-0.356725425	-3.148087655
H	1.001517256	-0.740785006	-4.866198889
H	2.442565053	-2.040204633	-0.191962880
C	-1.431103094	4.126734499	-1.599487234
C	-0.599369920	3.094524458	-2.460916055
O	-1.205609573	1.830530724	-2.110098570
C	-0.734035563	3.294208422	-3.972626382
C	0.884914926	3.036155357	-2.081355961
O	-1.877755746	3.335626408	-0.476639757
C	-2.682315429	4.640267206	-2.328968248
C	-0.619369451	5.301223317	-1.059285648
B	-1.937159173	2.003544076	-0.903213584
H	-3.152670519	1.502004610	-1.065642085
H	-1.761414817	3.138040273	-4.306306442
H	-0.418235242	4.302451502	-4.261595796
H	-0.093874286	2.583118201	-4.503397862
H	-3.308447656	5.179556447	-1.612155387
H	-2.428494496	5.320722840	-3.147461899
H	-3.269842154	3.813601779	-2.736337550
H	1.340368409	2.170799933	-2.570297473
H	1.416442594	3.935937560	-2.407722847
H	1.019837297	2.917770217	-1.007170172
H	0.185004451	4.954549397	-0.408293785
H	-0.185641307	5.885625851	-1.877933876
H	-1.269998732	5.959240801	-0.475785836
C	-6.761028070	0.608719005	-3.964980105
C	-7.249623795	1.863685861	-3.587620574
C	-6.581829580	2.614311991	-2.617803337
H	-8.149894791	2.255614655	-4.052298239
H	-7.279465287	0.025917050	-4.720805092
C	-5.606618942	0.103508957	-3.376808172
H	-5.208755627	-0.866621392	-3.656438939
C	-4.933730983	0.862954149	-2.402779037
H	-6.961650100	3.588564059	-2.325458830
C	-5.423127681	2.118235939	-2.023389322
H	-4.886923731	2.692030021	-1.274351279
C	-3.723674108	0.299889928	-1.824302338
N	-3.044218253	-0.685324371	-1.774057672

===============================

[1]4-TS

===============================

Mn	-1.175227139	-0.832263899	-1.394599926
C	-1.609689511	-2.420784518	-0.744159670
O	-1.940749756	-3.443759056	-0.298020591
N	0.718428332	-0.875155243	-0.722415265
C	1.128698460	0.189799096	0.134038201

C 0.150921271 0.875613720 0.878560300
C 0.519564900 1.928692332 1.719923286
C 1.854254114 2.319856916 1.818187743
C 2.825548301 1.663038578 1.058528062
C 2.465543556 0.611113217 0.220857634
H 3.215403693 0.143330044 -0.409060890
H 3.862140443 1.984617896 1.100254680
H 2.130550535 3.146758366 2.465789838
H -0.252750337 2.452646003 2.273642177
S -1.579605248 0.466037995 0.636226813
C 1.572417838 -1.858452781 -0.869182219
C 1.443564015 -3.006033059 -1.753264786
C 0.577559111 -3.111607978 -2.871734727
C 0.521735232 -4.291592277 -3.610837808
C 1.318283449 -5.387897181 -3.271907707
C 2.206701197 -5.287578982 -2.200938387
C 2.272379565 -4.110223922 -1.464807341
H 2.959655789 -4.039423141 -0.625263892
H 2.842733598 -6.126599748 -1.935131851
H 1.248028246 -6.306678061 -3.846182402
H -0.156405418 -4.347841536 -4.456930677
S -0.464885831 -1.750567787 -3.414930302
C 0.847255601 -0.659350825 -4.094780048
H 0.334884430 0.175156746 -4.572363945
H 1.483188410 -0.289417594 -3.288820999
H 1.435245774 -1.205133608 -4.835725136
H 2.452655100 -1.882502135 -0.225023749
C -1.487253071 3.852083158 -1.657041404
C -0.570642340 2.786635553 -2.388295818
O -1.074765550 1.525173065 -1.857629272
C -0.722803123 2.794818147 -3.907893017
C 0.904911370 2.885313446 -1.998257434
O -1.884920899 3.170997832 -0.437799118
C -2.762724451 4.186617906 -2.440940578
C -0.763797540 5.137107719 -1.262723768
B -1.843991547 1.815690669 -0.705614010
H -3.233970157 1.283221056 -1.040893983
H -1.719553146 2.468128937 -4.208662172
H -0.543995319 3.799628002 -4.304850060
H 0.011368529 2.128244830 -4.364727396
H -3.432459597 4.758008264 -1.792569221
H -2.543352186 4.790265731 -3.326410755
H -3.292475599 3.286067647 -2.755543146
H 1.431741421 2.007478468 -2.380943628
H 1.368734265 3.779943382 -2.424960804
H 1.029330378 2.902960975 -0.914974461
H 0.055400184 4.936493041 -0.570385172
H -0.364252932 5.643880616 -2.147679088
H -1.467376066 5.814562740 -0.770842334
C -6.847708326 0.877250132 -3.864866169
C -7.394364336 2.025593469 -3.281298428
C -6.735181604 2.644568447 -2.217654736
H -8.328974045 2.434633011 -3.655548032
H -7.359511334 0.393532674 -4.692674310
C -5.648695308 0.351847234 -3.393127263
H -5.206307437 -0.537292693 -3.831292414
C -4.979257521 0.976064117 -2.327703577
H -7.156283685 3.535495431 -1.759551167
C -5.532899653 2.122361274 -1.742298366
H -5.007291363 2.603624315 -0.921346390
C -3.697480145 0.430079731 -1.851365872
N -3.020649187 -0.563102076 -2.097200260
================================
4
================================
Mn1 1.392584 0.941011 0.369926
N2 0.866888 0.580164 -1.602221
C3 0.636687 1.776780 -2.313851
C4 0.233235 2.948893 -1.609255
C5 0.003757 4.122433 -2.343460
C6 0.176752 4.172200 -3.726379
C7 0.597854 3.030730 -4.410163
C8 0.823633 1.849751 -3.706531
H9 1.176109 0.965109 -4.229544
H10 0.757410 3.057561 -5.484914
H11 -0.011390 5.099016 -4.262140
H12 -0.326847 5.007468 -1.806288
S13 0.013194 2.958593 0.162433
C14 0.478544 -0.573046 -2.187358
C15 0.653504 -1.901721 -1.706185
C16 1.513977 -2.369592 -0.650892
C17 1.465893 -3.703645 -0.223999
C18 0.608750 -4.629229 -0.809382
C19 -0.193867 -4.214816 -1.890087
C20 -0.160236 -2.906622 -2.322300
H21 -0.803773 -2.599344 -3.143534
H22 -0.850217 -4.925815 -2.385347
H23 0.582994 -5.655209 -0.455894
H24 2.135067 -4.014923 0.573711
S25 2.755431 -1.350137 0.138320
C26 3.812283 -0.875556 -1.286045
H27 4.624260 -0.264945 -0.887244
H28 4.206897 -1.776563 -1.759196
H29 3.222746 -0.286014 -1.989010
H30 -0.157564 -0.484495 -3.069893
C31 1.714325 1.985788 4.669840
C32 3.142687 1.931068 4.099150
C33 3.046204 0.748335 3.141299
H34 3.909844 1.793649 4.866131
H35 3.368449 2.843802 3.536666
C36 0.850064 1.614313 3.456203
H37 1.597110 1.245074 5.468672
H38 1.449299 2.965816 5.075295
O39 1.725707 0.866886 2.561784
H40 -0.006111 0.980007 3.707916
H41 0.495197 2.490206 2.905464
H42 3.122158 -0.214084 3.664582
H43 3.763203 0.770820 2.318972
N44 3.407569 2.071251 0.062552
C45 3.655089 3.058966 -0.501893
C46 3.844305 4.303207 -1.168972
C47 2.820354 5.262228 -1.066317
C48 5.005655 4.554427 -1.918957
C49 2.972443 6.478728 -1.724358
C50 5.139323 5.778122 -2.567549
C51 4.125748 6.737491 -2.470222
H52 1.928201 5.024251 -0.494205
H53 5.779972 3.796864 -1.989471

H54 2.183594 7.222363 -1.661721
H55 6.030869 5.983832 -3.152244
H56 4.234695 7.689406 -2.982909
C57 -0.628104 -0.366728 0.924455
O58 -1.683690 -0.780094 0.852423

===============================

^{3}4

===============================

Mn 1.291443990 0.940972369 0.422200165
N 0.697365874 0.735881961 -1.452128505
C 0.346269286 1.935171797 -2.106326948
C -0.074830609 3.028358341 -1.303116762
C -0.488900994 4.210683738 -1.929037967
C -0.453704068 4.343045557 -3.320520368
C 0.008740753 3.284844599 -4.108033326
C 0.405134909 2.090898998 -3.503368981
H 0.786036667 1.274704893 -4.110178318
H 0.067619360 3.386750573 -5.188172630
H -0.770639469 5.274383622 -3.782439868
H -0.831596435 5.034711506 -1.309358024
S -0.065745991 2.827757661 0.454899356
C 0.403032512 -0.425532631 -2.093374333
C 0.707286104 -1.751464952 -1.686504419
C 1.658344340 -2.156436807 -0.689642609
C 1.795514876 -3.496669072 -0.315988206
C 1.027302325 -4.494174487 -0.910709762
C 0.131384060 -4.134530714 -1.936224100
C -0.013585048 -2.815983561 -2.314328582
H -0.724913486 -2.556655949 -3.095080690
H -0.459164509 -4.900172572 -2.432688317
H 1.135609408 -5.529161121 -0.602747941
H 2.529393886 -3.753580867 0.443330088
S 2.787370330 -0.986644236 0.050073118
C 3.777313414 -0.499025808 -1.417650082
H 4.527641320 0.213140090 -1.074449847
H 4.252794609 -1.389052806 -1.833429423
H 3.121618663 -0.024905858 -2.148201345
H -0.258336234 -0.343130442 -2.954985038
C 1.527052187 1.480800160 4.770321941
C 3.003165377 1.182564006 4.456437271
C 2.919937816 0.520216096 3.079135148
H 3.483032451 0.536678976 5.196576482
H 3.572500849 2.116611075 4.396592952
C 0.998525817 1.900604507 3.402811789
H 1.017169575 0.574029592 5.115596582
H 1.392990144 2.259712584 5.525496997
O 1.725925453 1.067828117 2.462466528
H -0.065680612 1.714604277 3.245051466
H 1.211498481 2.949976720 3.172290381
H 2.795809723 -0.566300870 3.141805548
H 3.765953357 0.745234445 2.426131347
N 3.351215740 2.255193782 0.180843188
C 3.690802252 3.112346338 -0.528330757
C 4.052206350 4.149220878 -1.441178495
C 3.107022821 4.567229863 -2.393377887
C 5.331350406 4.727930610 -1.388322019
C 3.456212122 5.568623357 -3.294837711
C 5.662378837 5.727385055 -2.297659902
C 4.727579515 6.145559874 -3.250256006
H 2.121120166 4.119491738 -2.412987031
H 6.048024901 4.390171779 -0.646409259
H 2.727037294 5.893412215 -4.030383715
H 6.648591187 6.180309064 -2.264348186
H 4.992239503 6.928218878 -3.955601868
C -0.495356282 -0.528449475 1.139785138
O -1.543075038 -0.971929333 1.108553903

===============================

^{1}4

===============================

Mn 1.160592577 0.815644592 0.414641893
N 0.603329654 0.652814383 -1.511261467
C 0.271007105 1.888262340 -2.140837232
C -0.240207177 2.932761801 -1.321230471
C -0.638253672 4.122933770 -1.972359349
C -0.482742707 4.294417093 -3.343567586
C 0.096372567 3.284203133 -4.126114632
C 0.464240780 2.088207518 -3.518251699
H 0.942613027 1.309532342 -4.105729754
H 0.269168089 3.433346474 -5.187533919
H -0.788170841 5.230856740 -3.804053964
H -1.058143263 4.920006901 -1.365564401
S -0.350110605 2.740055487 0.409698870
C 0.424519065 -0.436430450 -2.208614259
C 0.754506918 -1.802928939 -1.817302848
C 1.660042705 -2.196441865 -0.800161887
C 1.864932866 -3.546566285 -0.517615492
C 1.187915310 -4.539768578 -1.229150338
C 0.319242683 -4.173435898 -2.257382463
C 0.119250201 -2.828352448 -2.548925333
H -0.562555929 -2.549056579 -3.348435085
H -0.204561186 -4.933205241 -2.830228272
H 1.346423435 -5.586140897 -0.986037989
H 2.564063590 -3.816961354 0.267970138
S 2.627891651 -0.998719660 0.133619990
C 3.821185173 -0.532824274 -1.186353327
H 4.515763779 0.184876941 -0.747241285
H 4.359151896 -1.418086659 -1.530345379
H 3.283844358 -0.055117716 -2.006911360
H -0.082664701 -0.356319387 -3.170906769
C 1.820770321 1.841301702 4.691300199
C 3.236660606 2.040653334 4.122685759
C 3.211563734 1.165615583 2.863562822
H 4.031591207 1.751628387 4.815943553
H 3.385462558 3.091765649 3.850024868
C 0.987010093 1.793275009 3.414568293
H 1.752899100 0.889544903 5.230764650
H 1.509463533 2.643889157 5.365310364
O 1.827146653 1.100030320 2.463510051
H 0.055947385 1.227244126 3.491346664
H 0.763468882 2.793587797 3.025234075
H 3.554125393 0.141927611 3.058609385
H 3.791232777 1.579786032 2.033858716
N 2.548710651 2.124696358 -0.152907708
C 3.192915163 2.966399061 -0.634861008
C 3.854739257 4.031223843 -1.309915994
C 3.087693244 4.867989363 -2.143797980
C 5.236897408 4.238984218 -1.161595194
C 3.716636654 5.910141617 -2.817893898

C	5.848069166	5.286515223	-1.844186011
C	5.090613955	6.121969832	-2.670319264
H	2.021822456	4.691650564	-2.245964496
H	5.814412668	3.582529094	-0.518672568
H	3.130572186	6.559593199	-3.460645333
H	6.915551404	5.451216137	-1.733731938
H	5.572414299	6.938317482	-3.200474213
C	-0.192229351	-0.227222409	0.995145803
O	-1.087713792	-0.854914826	1.391448642

==============================

4-TSa

==============================

Mn	-1.497526944	0.996972254	1.219288788
C	-3.072224541	2.071270182	-0.348739673
O	-3.749685262	2.777642087	-0.928621884
N	0.068148679	0.480131732	-0.058463072
C	1.247020898	1.215115318	0.025447031
C	1.242549585	2.557896863	0.491222186
C	2.420338309	3.306117252	0.527752175
C	3.635713731	2.752878045	0.121056055
C	3.657345043	1.442166016	-0.356222768
C	2.487130558	0.693146074	-0.414481294
H	2.511368852	-0.306605334	-0.835300864
H	4.588604136	1.000741620	-0.703251594
H	4.545534710	3.344396756	0.166035283
H	2.379836959	4.317956914	0.914770999
S	-0.279831778	3.315453589	1.078758694
C	0.167329268	-0.874005270	-0.185551220
C	-0.693003191	-1.752013565	-0.909340763
C	-1.669875465	-1.419177087	-1.913665313
C	-2.444541879	-2.427378328	-2.500380099
C	-2.293096511	-3.773695019	-2.166869997
C	-1.296976122	-4.127683767	-1.242699852
C	-0.526404249	-3.148310625	-0.645306179
H	0.233355407	-3.436210556	0.078489832
H	-1.129038704	-5.172009071	-0.991375388
H	-2.908267649	-4.530189109	-2.644591535
H	-3.163755380	-2.139947632	-3.262719045
S	-1.979624579	0.233086293	-2.528112102
C	-0.354633969	0.729108830	-3.212485992
H	-0.410132879	1.803924267	-3.401335803
H	0.432975276	0.547345209	-2.483645821
H	-0.154805103	0.193687304	-4.143241677
H	1.000692295	-1.365496383	0.327042240
C	1.072459591	5.345972847	4.267670087
C	-0.495380268	5.416612822	4.229567626
O	-0.842435727	4.093070127	3.765745659
C	-1.157703960	5.654646022	5.584504839
C	-1.015959844	6.427562314	3.197308837
O	1.363118164	4.486636756	3.147285957
C	1.595444010	4.667947760	5.543277591
C	1.783335226	6.679485691	4.051425433
B	0.241616324	3.635446106	2.969149865
H	0.541699642	2.376650349	3.251006885
H	-0.935264809	4.844834366	6.282215697
H	-0.813979471	6.598391442	6.022188181
H	-2.243860831	5.713520772	5.465609687
H	2.659294086	4.449126581	5.418507588
H	1.473331020	5.307471997	6.423242048
H	1.080441031	3.720338148	5.721627828
H	-2.093400463	6.281528014	3.070700882
H	-0.845025424	7.460562070	3.516553236
H	-0.535431775	6.272923594	2.227637427
H	1.546106458	7.099075346	3.072111121
H	1.501781623	7.402438472	4.825202522
H	2.866393537	6.531345907	4.101483372
C	2.649002711	-0.615633694	6.105979047
C	3.770753519	0.217258815	6.048354484
C	3.790207561	1.307244511	5.176002611
H	4.628363534	0.014287073	6.683943635
H	2.633043820	-1.463518245	6.785219968
C	1.551158828	-0.362519240	5.289354659
H	4.663822945	1.950917419	5.128222835
C	2.689359279	1.580217105	4.365581460
H	0.675961068	-1.004740352	5.308035415
C	1.568845095	0.741974629	4.421899838
H	2.684777659	2.435319442	3.697386002
C	0.422626306	0.958962575	3.539311655
N	-0.483994976	0.388949603	2.995894182
H	-4.008487842	-0.864946694	-0.488505770
O	-2.875907893	-0.734744063	1.234660048
C	-2.581761948	-1.955490958	1.961840185
C	-3.585953592	-2.973606411	1.428867703
C	-4.813769202	-2.094823117	1.133365626
C	-4.172475870	-0.815038791	0.592330737
H	-1.537786333	-2.203424501	1.768052250
H	-2.709574752	-1.755622166	3.032330009
H	-3.790394489	-3.771376109	2.148637516
H	-3.207439490	-3.421030235	0.505827123
H	-5.371577146	-1.886828644	2.054347606
H	-5.502188408	-2.543934311	0.412443296
H	-4.720411426	0.098890249	0.840621330
H	-4.607115692	3.414482413	2.147547017
C	-3.694591255	3.194630443	2.719335049
O	-3.334076671	1.806094466	2.499129633
C	-3.726945875	1.001394370	3.641008036
C	-4.510204673	1.936999214	4.567933505
H	-4.319167774	0.156792315	3.277523654
H	-2.811083882	0.619294020	4.102782632
C	-3.913423934	3.310322805	4.223886121
H	-4.390701981	1.666100256	5.620815901
H	-5.579937278	1.909363797	4.328928790
H	-2.942353257	3.445076428	4.705261801
H	-4.565817931	4.147162066	4.490524661
H	-2.870325837	3.812896753	2.363029449

==============================

4-TSb

==============================

Mn	-1.390842889	-2.259556072	-1.600034513
C	-2.600148832	-3.421307061	0.321377965
O	-3.094871486	-3.669096350	1.312766100
N	0.276972996	-3.278988313	-1.005391396
C	1.461953735	-2.599345820	-0.729176235
C	1.533266097	-1.648735558	0.328072681
C	2.742058721	-0.994552988	0.583405296
C	3.885624673	-1.244099930	-0.175352492
C	3.825700646	-2.186115342	-1.202120831
C	2.638065316	-2.858144927	-1.466269165

H 2.610346534 -3.619860758 -2.238778204
H 4.708982427 -2.408966686 -1.795419625
H 4.807581024 -0.710554813 0.035818939
H 2.769562803 -0.265440094 1.387276649
S 0.119104285 -1.240872612 1.361642538
C 0.048462968 -3.836928724 -2.233290665
C -0.726340586 -5.058400003 -2.398418479
C -1.066685595 -5.954449968 -1.336445155
C -1.918399131 -7.035390050 -1.612532645
C -2.386208956 -7.294158144 -2.902263370
C -1.992369418 -6.469819837 -3.959768398
C -1.183595728 -5.372684273 -3.698927396
H -0.887838534 -4.711881357 -4.510894268
H -2.318216829 -6.679527264 -4.975084505
H -3.035979751 -8.147193191 -3.075943408
H -2.214383399 -7.709110998 -0.817778991
S -0.348572043 -5.707921089 0.287949015
C -0.761717288 -7.281779288 1.124820987
H -0.236055011 -7.235590064 2.081661386
H -1.832686573 -7.381366732 1.324603621
H -0.403931856 -8.149209462 0.563482482
H 0.594407871 -3.481813842 -3.113892115
C -2.459182516 1.646363344 0.132865496
C -3.164435537 0.294991495 -0.281760394
O -2.038543475 -0.620125007 -0.370928387
C -4.088253808 -0.258791166 0.806917000
C -3.879824373 0.326319405 -1.629902677
O -1.291415322 1.201937114 0.843956991
C -3.286633978 2.520067954 1.075067129
C -2.013537143 2.483174056 -1.078215751
B -0.869618900 -0.015379378 0.272339400
H -0.040505750 0.181387889 -0.790120090
H -3.559191587 -0.327096819 1.760615086
H -4.977468380 0.365375638 0.935729314
H -4.415631390 -1.263806808 0.526321710
H -2.734853426 3.438492972 1.295036469
H -4.241831461 2.797368620 0.615641543
H -3.481287044 2.010787058 2.020288680
H -4.301749047 -0.660623817 -1.841157723
H -4.691592290 1.060904217 -1.623070251
H -3.184011989 0.570623552 -2.434282669
H -1.455510612 1.888409211 -1.802850112
H -2.868912009 2.934884711 -1.590307630
H -1.358913133 3.284312587 -0.724591246
C 3.211362188 1.634212304 -3.777943516
C 3.597050173 2.542471936 -2.787350390
C 2.877768211 2.625151326 -1.593344762
H 4.460730699 3.182227230 -2.946588109
H 3.774480062 1.564859703 -4.704552336
C 2.107108063 0.813022360 -3.577192872
H 3.179998213 3.329102232 -0.823539646
C 1.775019473 1.800122895 -1.380814987
H 1.789814215 0.097463658 -4.328998912
C 1.386233282 0.896985193 -2.375170432
H 1.210936262 1.844502827 -0.454035042
C 0.233546746 0.013667539 -2.199177442
N -0.471134658 -0.747322031 -2.816253707
H -2.448428320 -2.862863158 -4.698918019
O -3.166167311 -2.751890341 -2.750739753
C -4.096791429 -3.848488957 -2.502596054
C -4.803269410 -4.098861314 -3.839978094
C -4.629804847 -2.761900806 -4.583445701
C -3.233387505 -2.340042247 -4.140540274
H -3.530198593 -4.711351938 -2.147898079
H -4.783514711 -3.510257727 -1.718729065
H -5.849854043 -4.384261210 -3.703827390
H -4.293293857 -4.903341223 -4.377549398
H -5.370984129 -2.028366012 -4.246115644
H -4.711850861 -2.861106015 -5.669226266
H -3.034210674 -1.267686614 -4.163952555

====================================

5-TS

====================================

Mn1 0.993121 1.271788 0.506947
N2 0.071881 0.700691 -1.305614
C3 -0.637405 1.750064 -1.902943
C4 -1.369360 2.682124 -1.125649
C5 -2.023012 3.760974 -1.723814
C6 -1.976060 3.943569 -3.104613
C7 -1.270904 3.027680 -3.889216
C8 -0.615330 1.952573 -3.300392
H9 -0.032828 1.268174 -3.909681
H10 -1.219148 3.158831 -4.967179
H11 -2.484252 4.788395 -3.559693
H12 -2.562320 4.460966 -1.095017
S13 -1.497440 2.448239 0.658691
B14 -0.623369 3.959993 1.291613
O15 -0.602311 5.167057 0.644032
C16 -0.198201 6.170052 1.625605
C17 -0.572592 5.467295 2.983275
O18 -0.410254 4.053443 2.654010
C19 -2.044716 5.654890 3.371119
H20 -2.705263 5.414226 2.533422
H21 -2.282319 4.976483 4.195101
H22 -2.248012 6.680210 3.694329
C23 0.331888 5.821244 4.158904
H24 -0.011095 5.308264 5.062107
H25 1.367485 5.534344 3.974514
H26 0.297523 6.898763 4.351557
C27 1.305501 6.426656 1.484998
H28 1.624645 7.231205 2.154508
H29 1.884915 5.528739 1.702691
H30 1.527634 6.725310 0.457572
C31 -0.976749 7.448243 1.328705
H32 -2.050475 7.261658 1.274158
H33 -0.787867 8.202683 2.099910
H34 -0.654209 7.855899 0.366519
C35 0.059586 -0.493727 -1.947911
C36 0.822583 -1.652976 -1.637961
C37 2.011521 -1.743688 -0.832763
C38 2.564238 -2.990022 -0.512668
C39 2.008798 -4.177269 -0.980475
C40 0.895118 -4.110641 -1.839498
C41 0.335193 -2.892870 -2.161046
H42 -0.541305 -2.859710 -2.804470
H43 0.466863 -5.022399 -2.247972
H44 2.451022 -5.132212 -0.714562

H45 3.464772 -3.015418 0.095217
S46 2.939417 -0.312277 -0.284575
C47 3.340816 0.496960 -1.883297
H48 4.026369 1.316191 -1.663992
H49 3.815139 -0.234066 -2.540666
H50 2.429165 0.892838 -2.330555
H51 -0.708147 -0.631667 -2.711874
H52 1.297022 4.708216 -0.876339
C53 1.309255 2.208162 4.659094
C54 2.484813 2.749757 3.825812
C55 2.892877 1.520000 3.028060
H56 3.296285 3.158945 4.434629
H57 2.129062 3.508118 3.124231
C58 0.665965 1.168573 3.722780
H59 1.673025 1.732382 5.576374
H60 0.595665 2.987650 4.929536
O61 1.631814 0.915328 2.662460
H62 0.441000 0.220690 4.223935
H63 -0.240218 1.552957 3.249708
H64 3.477549 0.804250 3.623381
H65 3.414013 1.744616 2.097573
N66 1.683216 3.103795 0.379176
C67 2.026454 3.981883 -0.463900
C68 3.407295 4.180529 -0.988361
C69 3.627605 5.046247 -2.067173
C70 4.503161 3.514275 -0.418945
C71 4.911103 5.223967 -2.585643
C72 5.785465 3.693338 -0.928624
C73 5.992502 4.547186 -2.018460
H74 2.780872 5.571390 -2.505608
H75 4.316657 2.848662 0.418357
H76 5.069004 5.888927 -3.430854
H77 6.626900 3.170439 -0.480555
H78 6.993107 4.685550 -2.419287
C79 -0.395160 -0.698585 1.232882
O80 -1.208513 -1.501503 1.138606
==============================

^{3}5-TS

==============================

Mn	0.576846317	1.004377524	0.615334292
N	0.013995049	0.759326714	-1.299067193
C	-0.751122662	1.777654344	-1.867664200
C	-1.440388276	2.690588049	-1.037865887
C	-2.175002167	3.747161446	-1.570450854
C	-2.252577325	3.924433827	-2.952125732
C	-1.576178107	3.034552946	-3.791067294
C	-0.836195054	1.981402093	-3.263684094
H	-0.271084091	1.331814431	-3.924232736
H	-1.608490047	3.173322493	-4.868855229
H	-2.820069032	4.752755688	-3.365201805
H	-2.667975737	4.439625919	-0.896371695
S	-1.318696328	2.486772189	0.746085216
B	-0.007530895	3.836837305	1.076189711
O	-0.151740571	5.025893089	0.374276996
C	0.347705440	6.092995656	1.219788072
C	0.174310260	5.474484820	2.653725364
O	0.364746296	4.054415782	2.402480893
C	-1.245815409	5.644662600	3.207016823
H	-1.990587487	5.326358332	2.472667927
H	-1.356622026	5.018384600	4.096860979
H	-1.447964887	6.683167405	3.486050581
C	1.202648958	5.944361230	3.678465809
H	0.982088787	5.512486561	4.659324823
H	2.213778238	5.649335718	3.392581342
H	1.171672683	7.034729736	3.778230774
C	1.814489187	6.358017406	0.869699175
H	2.195338948	7.236291966	1.400243830
H	2.438835413	5.499069344	1.115729095
H	1.902792428	6.534830069	-0.204911610
C	-0.489522871	7.339524088	0.947130958
H	-1.557329116	7.133811777	1.039632822
H	-0.222810620	8.145099750	1.639976436
H	-0.299104650	7.689604196	-0.071596014
C	0.055322598	-0.423234279	-1.974074518
C	0.875593307	-1.553686364	-1.735119341
C	1.972325983	-1.655900663	-0.813907228
C	2.653195058	-2.860725858	-0.630031198
C	2.313451708	-4.001122115	-1.355619697
C	1.272175872	-3.920521255	-2.300406927
C	0.581806684	-2.740200243	-2.481076773
H	-0.229645682	-2.698148896	-3.204042029
H	1.005355789	-4.793020656	-2.890624974
H	2.855558885	-4.928901857	-1.203283002
H	3.473369663	-2.892386610	0.082273334
S	2.575004165	-0.229804388	0.073609608
C	3.399344630	0.647148285	-1.307165800
H	3.962583637	1.480114007	-0.891496910
H	4.069663346	-0.060445442	-1.797940310
H	2.644117742	1.016806052	-2.001181195
H	-0.677994143	-0.546718521	-2.771278895
H	1.122194023	3.474074125	-1.575310669
C	0.962078119	1.369445650	5.058988972
C	2.111490433	2.283560849	4.599111839
C	2.360112893	1.830968207	3.158606200
H	3.006815655	2.194685100	5.221085712
H	1.783505079	3.325304163	4.603237699
C	0.128358518	1.259657777	3.785634198
H	1.341440094	0.383733863	5.351480668
H	0.390193358	1.781463863	5.895418579
O	1.105659894	1.233407334	2.721459604
H	-0.464869395	0.345197800	3.709525874
H	-0.515987882	2.134275526	3.649247372
H	3.124722406	1.048669154	3.085420553
H	2.584032232	2.637488266	2.461565591
N	1.640522408	2.759045297	0.326497466
C	1.871417398	3.371359143	-0.769666648
C	3.155293588	4.035299555	-1.107184394
C	3.288186147	4.703172001	-2.331935402
C	4.247633434	4.002763592	-0.225395891
C	4.486397152	5.333107382	-2.669670772
C	5.443474679	4.628154616	-0.560809641
C	5.565550990	5.296921349	-1.784780660
H	2.441625029	4.728091155	-3.015119966
H	4.123244851	3.480352448	0.719058262
H	4.580156831	5.851713179	-3.620092516
H	6.284775356	4.599526756	0.126901299
H	6.500032541	5.786454146	-2.046055851
C	-0.202084633	-0.583308459	1.136072164

O -0.709800029 -1.548232192 1.513237939
==============================
[1]5-TS
==============================
Mn 0.648680884 1.039640409 0.496232473
N -0.123681562 0.655357440 -1.281213381
C -0.923475311 1.674936760 -1.843982952
C -1.604618931 2.568963823 -0.996027993
C -2.423013977 3.571631674 -1.511739173
C -2.565246772 3.721532270 -2.891707002
C -1.869186569 2.864808361 -3.746831547
C -1.057325493 1.856214737 -3.233062384
H -0.488265711 1.222000248 -3.904999071
H -1.946879734 2.988864071 -4.823681025
H -3.197368764 4.508498217 -3.291697342
H -2.925087381 4.247302211 -0.827931264
S -1.359439194 2.397374901 0.776405365
B -0.300567730 3.924209140 1.036223746
O -0.514710323 5.071068655 0.313724181
C -0.014517705 6.191606844 1.098459639
C 0.048930925 5.581620174 2.551613441
O 0.223715545 4.159278208 2.294519549
C -1.268913838 5.721528567 3.323940384
H -2.112030931 5.357257566 2.730312350
H -1.210536969 5.116293263 4.233008066
H -1.463015611 6.759648533 3.609427031
C 1.214446239 6.081517205 3.400240930
H 1.159826976 5.648354480 4.403123282
H 2.175185529 5.808414804 2.960598771
H 1.173912259 7.170960477 3.503529653
C 1.357225635 6.604675000 0.557986471
H 1.704729028 7.522943530 1.041678134
H 2.106642764 5.828436322 0.707793982
H 1.276799955 6.789048971 -0.516398157
C -1.000763085 7.345689776 0.931585059
H -2.022170416 7.038921554 1.162707527
H -0.728575314 8.184498596 1.581091112
H -0.978647806 7.695595297 -0.104391242
C -0.076190874 -0.500102901 -1.964857294
C 0.786168036 -1.622126429 -1.730261349
C 1.976429275 -1.627041644 -0.942921037
C 2.707467890 -2.797558565 -0.748829973
C 2.308635602 -3.998767468 -1.337493583
C 1.173011202 -4.009400259 -2.158610742
C 0.437944941 -2.851810441 -2.352522770
H -0.449044782 -2.877626505 -2.981347117
H 0.860675961 -4.930225891 -2.643752437
H 2.883168171 -4.905162028 -1.173611208
H 3.607792673 -2.757513860 -0.142077722
S 2.627256417 -0.106838923 -0.252628668
C 3.120106208 0.718390240 -1.817218167
H 3.789645418 1.537129187 -1.563429990
H 3.638080935 -0.015748653 -2.436778582
H 2.233712470 1.106914513 -2.316293329
H -0.829844956 -0.650105088 -2.737581921
H 1.382020703 4.370596529 -1.292849014
C 1.328350440 1.432611468 4.902455398
C 2.337149937 2.482791080 4.405792775
C 2.473950864 2.158373581 2.912910225
H 3.296808492 2.442136057 4.928719574
H 1.919843497 3.485673340 4.527585482
C 0.396326848 1.317057365 3.700430114
H 1.823998143 0.473218535 5.089338506
H 0.803290252 1.733701474 5.813588507
O 1.275660340 1.420705592 2.561769803
H -0.128968794 0.362959547 3.624612576
H -0.326284517 2.140797199 3.667360875
H 3.325635768 1.503167721 2.696970555
H 2.520642692 3.040465573 2.276414144
N 1.527902858 2.740269421 -0.008169079
C 2.015397040 3.678079011 -0.695401335
C 3.466295227 4.038607776 -0.781485096
C 3.880426423 5.058457534 -1.649572505
C 4.433470328 3.392520319 0.004859888
C 5.224885693 5.420246871 -1.740072854
C 5.776327064 3.752131276 -0.081181078
C 6.178738190 4.768890041 -0.954402154
H 3.134441880 5.567810966 -2.257086379
H 4.105227606 2.600388637 0.670228925
H 5.530034058 6.210306002 -2.421643661
H 6.514262252 3.241002098 0.532246421
H 7.226182407 5.050361254 -1.020554895
C -0.033874630 -0.530116617 1.138131609
O -0.497770480 -1.501150007 1.568062044
===================================
5
===================================
Mn1 1.079667 1.033751 0.470636
N2 0.014096 0.488897 -1.280328
C3 -0.574990 1.601515 -1.885283
C4 -1.183177 2.625719 -1.114315
C5 -1.738506 3.754399 -1.718816
C6 -1.693967 3.911686 -3.102960
C7 -1.095941 2.918187 -3.882119
C8 -0.552030 1.787229 -3.286606
H9 -0.050713 1.040512 -3.894101
H10 -1.040398 3.031257 -4.961917
H11 -2.119909 4.798159 -3.563036
H12 -2.194145 4.517599 -1.098841
S13 -1.278275 2.408749 0.678439
B14 -0.642316 3.999221 1.336342
O15 -0.441607 5.130354 0.609523
C16 0.091214 6.133514 1.535624
C17 -0.415929 5.593926 2.923236
O18 -0.463757 4.145680 2.688493
C19 -1.850066 6.021827 3.252942
H20 -2.522055 5.830166 2.411622
H21 -2.205888 5.443932 4.110179
H22 -1.900494 7.085121 3.504534
C23 0.507369 5.881967 4.100308
H24 0.081654 5.466704 5.018444
H25 1.497825 5.451267 3.955511
H26 0.614755 6.963216 4.236430
C27 1.613718 6.119349 1.394976
H28 2.074898 6.875304 2.037601
H29 2.030772 5.139630 1.635212
H30 1.882088 6.335528 0.359165
C31 -0.467751 7.493608 1.130706

H32 -1.556962 7.481653 1.064688
H33 -0.167038 8.260906 1.852015
H34 -0.070521 7.772969 0.151201
C35 -0.082155 -0.693255 -1.940140
C36 0.609936 -1.908898 -1.683648
C37 1.801466 -2.116159 -0.903661
C38 2.290715 -3.409326 -0.678952
C39 1.669926 -4.533079 -1.217093
C40 0.546072 -4.352598 -2.045278
C41 0.047264 -3.087367 -2.272595
H42 -0.834033 -2.965630 -2.898458
H43 0.063523 -5.210578 -2.505839
H44 2.067433 -5.524513 -1.024052
H45 3.193868 -3.522549 -0.085275
S46 2.798039 -0.787011 -0.236004
C47 3.353749 0.098493 -1.745830
H48 3.861620 0.998670 -1.395387
H49 4.024796 -0.540740 -2.322374
H50 2.482183 0.388464 -2.333312
H51 -0.861887 -0.761907 -2.701551
H52 1.375711 3.760204 -1.348643
C53 1.285309 2.205614 4.663379
C54 2.505868 2.698587 3.863523
C55 2.886393 1.466371 3.055400
H56 3.318249 3.067493 4.496621
H57 2.208673 3.485697 3.166533
C58 0.645683 1.158503 3.729280
H59 1.602110 1.742598 5.603771
H60 0.584119 3.010219 4.891196
O61 1.605899 0.913797 2.668019
H62 0.429492 0.212046 4.237844
H63 -0.267626 1.527406 3.257918
H64 3.432121 0.723816 3.654730
H65 3.420313 1.691450 2.130803
N66 2.262971 2.590187 0.141668
C67 2.259306 3.517888 -0.720031
C68 3.424646 4.408689 -1.003206
C69 3.334349 5.388832 -1.999648
C70 4.616170 4.291332 -0.272140
C71 4.410938 6.237273 -2.262957
C72 5.691074 5.136199 -0.530903
C73 5.591440 6.113978 -1.527872
H74 2.409901 5.481113 -2.567094
H75 4.662897 3.526090 0.498200
H76 4.330730 6.994237 -3.039146
H77 6.610142 5.038968 0.042519
H78 6.431108 6.774335 -1.728981
C79 -0.407029 -0.865019 1.350356
O80 -1.189778 -1.685534 1.285681

================================

$^{3}5$

================================

Mn	0.851621863	0.842516116	0.608764693
N	0.024098702	0.577657340	-1.236809864
C	-0.624752276	1.677578622	-1.817674677
C	-1.257768470	2.679000649	-1.038995452
C	-1.872768747	3.777487608	-1.643245063
C	-1.863717556	3.930929971	-3.027414318
C	-1.230681446	2.965486553	-3.812462476
C	-0.623869182	1.865065421	-3.219755118
H	-0.090563053	1.148025630	-3.834827984
H	-1.194415740	3.076091816	-4.893184471
H	-2.340190993	4.794079815	-3.482272369
H	-2.348574159	4.523429178	-1.017171707
S	-1.305577156	2.506073410	0.753234082
B	-0.611743620	4.104349058	1.326569528
O	-0.428035050	5.209533388	0.554260770
C	0.165022453	6.232789109	1.417392449
C	-0.260978895	5.741919461	2.850738177
O	-0.362122298	4.291838249	2.662648825
C	-1.656005959	6.221009407	3.266636325
H	-2.390719046	6.024320661	2.480664208
H	-1.965452467	5.678634164	4.164155736
H	-1.660357310	7.292346607	3.488082443
C	0.752810732	6.032363439	3.951528406
H	0.373382748	5.674507889	4.913060410
H	1.709734698	5.546650393	3.757639982
H	0.919714988	7.111433364	4.035139695
C	1.675305172	6.192731408	1.189622229
H	2.185497913	6.969103836	1.767314430
H	2.088800992	5.219958213	1.458874981
H	1.888250789	6.350907923	0.130444784
C	-0.404173567	7.586216284	1.006614442
H	-1.495551618	7.584297792	1.007453979
H	-0.052925325	8.370448062	1.685634912
H	-0.064791104	7.832696335	-0.003076209
C	-0.007732413	-0.575932590	-1.952513599
C	0.728446707	-1.784557137	-1.773723658
C	1.913580717	-2.010278429	-0.997488381
C	2.468537416	-3.288943980	-0.892355994
C	1.912555760	-4.383335040	-1.551080457
C	0.786646376	-4.179534366	-2.364997476
C	0.223055083	-2.923733383	-2.473725533
H	-0.658671445	-2.787404691	-3.095652019
H	0.349567512	-5.011950277	-2.910205119
H	2.358682069	-5.367995003	-1.452050909
H	3.366333664	-3.413557201	-0.293276097
S	2.805833081	-0.691848534	-0.184269777
C	3.471468663	0.202632947	-1.638476508
H	4.083798461	1.023361822	-1.263006905
H	4.073828146	-0.483555461	-2.236628019
H	2.645823349	0.609785274	-2.223422305
H	-0.784111306	-0.635765239	-2.715752733
H	1.556254538	2.903238010	-1.705297821
C	1.426164673	2.369580739	4.581359758
C	2.629544247	2.688054618	3.674271283
C	2.877880514	1.348694543	2.995294863
H	3.498375613	3.063166630	4.223339870
H	2.344853820	3.408277420	2.902087945
C	0.653233037	1.304370090	3.780213952
H	1.764798861	1.963583189	5.540645586
H	0.803189394	3.243868167	4.772764915
O	1.544242126	0.862991026	2.717200383
H	0.375800955	0.433075107	4.383519114
H	-0.240366197	1.711445695	3.304733788
H	3.388840789	0.630774021	3.651834158
H	3.391348705	1.424333818	2.037467528
N	1.958120566	2.296219930	0.242112603

C 2.161792812 3.007679090 -0.788836150
C 3.242855560 4.016005069 -0.900316783
C 3.288295128 4.868858532 -2.012727780
C 4.237832954 4.134832704 0.083807844
C 4.290507724 5.832568723 -2.131020019
C 5.239647263 5.093039584 -0.034430944
C 5.268613884 5.949177924 -1.141361248
H 2.522432814 4.777477758 -2.780358795
H 4.197082159 3.467008704 0.939236995
H 4.309727029 6.492325335 -2.994749519
H 6.004169429 5.176087082 0.734216062
H 6.051486662 6.697436762 -1.232724384
C -0.270608384 -0.644344303 1.181060814
O -1.001536272 -1.501177463 1.421383351

==============================

^{1}5

==============================

Mn 0.756088458 0.811167732 0.526251171
N 0.086161325 0.590213672 -1.334376772
C -0.578836198 1.711507141 -1.915526447
C -1.192399684 2.659965967 -1.077469554
C -1.859399409 3.762138211 -1.613233365
C -1.889291304 3.964023967 -2.991682040
C -1.245233670 3.057170302 -3.835510301
C -0.595665406 1.946465696 -3.303177340
H -0.051296517 1.278967318 -3.962001215
H -1.229866148 3.222188998 -4.908932750
H -2.396049517 4.833989929 -3.398905571
H -2.325695689 4.476582129 -0.944882083
S -1.083274746 2.385796836 0.693182024
B -0.436656576 4.010159519 1.295155620
O -0.360181641 5.143986782 0.548211370
C 0.216530017 6.174284564 1.417865004
C -0.145589000 5.630945274 2.847656266
O -0.166829623 4.181544213 2.626320624
C -1.556340512 6.017236845 3.305543451
H -2.296335097 5.798811186 2.530299697
H -1.813091347 5.434750025 4.194488803
H -1.617063310 7.080376141 3.556528257
C 0.873087214 5.961338142 3.932617424
H 0.548019041 5.545515657 4.890527159
H 1.859064997 5.558602419 3.701617596
H 0.958331265 7.047232324 4.046936510
C 1.719889763 6.193755756 1.145862991
H 2.216960395 6.982722604 1.718140634
H 2.175375491 5.233322421 1.391415015
H 1.896332429 6.369148319 0.082459103
C -0.420382507 7.511738837 1.059564067
H -1.510474357 7.464756014 1.093750867
H -0.081034571 8.291519114 1.749830856
H -0.121971882 7.799518620 0.047871274
C 0.108302439 -0.507760964 -2.083277836
C 0.762701795 -1.766316984 -1.801244483
C 1.806665011 -1.986326356 -0.862367241
C 2.327542935 -3.266054105 -0.665167693
C 1.848796936 -4.357846083 -1.390570583
C 0.848596927 -4.155353623 -2.347407586
C 0.326990188 -2.885627635 -2.550658830
H -0.456535870 -2.741135505 -3.290776902
H 0.472565635 -4.989969732 -2.932233289
H 2.258063560 -5.348349071 -1.217121280
H 3.121167665 -3.400069325 0.064142985
S 2.537210388 -0.639321615 0.068427152
C 3.565527194 0.093802041 -1.262876049
H 4.110115608 0.928980153 -0.822020235
H 4.253378132 -0.664615686 -1.641347592
H 2.919730591 0.474901674 -2.055167278
H -0.496926170 -0.519244203 -2.988830711
H 1.665347241 2.809290578 -1.824783185
C 1.476643078 2.275507572 4.623098442
C 2.667984318 2.660680919 3.725943067
C 2.866124069 1.397931750 2.899935483
H 3.558379226 2.955904191 4.289777191
H 2.382556307 3.464250748 3.042831492
C 0.675002687 1.293620739 3.742574098
H 1.826877133 1.782294598 5.536458490
H 0.871195977 3.138037111 4.908314715
O 1.522613352 0.964918815 2.618003664
H 0.410579286 0.369450197 4.270450808
H -0.234288653 1.744931330 3.343504086
H 3.392524471 0.609234215 3.457813912
H 3.338804624 1.579180619 1.935682847
N 1.954592459 2.308718695 0.185569304
C 2.205042326 2.964461605 -0.867585816
C 3.263665622 4.006249584 -0.980366585
C 3.301965676 4.849021830 -2.099694074
C 4.237098067 4.171168510 0.017434308
C 4.271375922 5.846853439 -2.212000187
C 5.209916913 5.160090089 -0.094401415
C 5.229276533 6.006627312 -1.208727185
H 2.551575076 4.723643618 -2.878495822
H 4.196572675 3.515496842 0.882541912
H 4.280371104 6.500279565 -3.081080289
H 5.958162607 5.276079452 0.686330090
H 5.987423248 6.780720071 -1.294576347
C -0.263400166 -0.590771241 1.126340758
O -0.933875856 -1.474129860 1.469103311

===================================

6-TS

===================================

Mn1 1.591806 0.980288 0.417515
N2 0.729391 0.377520 -1.386449
C3 0.396104 1.482997 -2.183611
C4 -0.013325 2.710891 -1.600803
C5 -0.254271 3.834550 -2.396603
C6 -0.099547 3.773440 -3.782168
C7 0.299890 2.573164 -4.373306
C8 0.547617 1.452334 -3.588218
H9 0.916342 0.543155 -4.049612
H10 0.441482 2.511873 -5.449830
H11 -0.284889 4.655791 -4.388161
H12 -0.543523 4.762364 -1.915064
S13 -0.205363 2.832942 0.181915
B14 0.927471 4.407879 0.599768
O15 0.436789 5.639529 0.098282
C16 0.694441 6.630942 1.108694
C17 0.598381 5.781523 2.427062
O18 1.156597 4.516370 2.009965

C19 -0.853827 5.531709 2.858868
H20 -1.446487 5.166586 2.015871
H21 -0.865372 4.766107 3.641465
H22 -1.324252 6.436563 3.256835
C23 1.420586 6.313556 3.597059
H24 1.295519 5.661514 4.467889
H25 2.483547 6.347837 3.349479
H26 1.093290 7.320390 3.878315
C27 2.103864 7.197534 0.873354
H28 2.329010 8.030649 1.547594
H29 2.865487 6.424494 1.002678
H30 2.170225 7.553900 -0.158311
C31 -0.348479 7.738135 0.977723
H32 -1.360261 7.328459 0.974206
H33 -0.259795 8.458057 1.799092
H34 -0.198400 8.274775 0.035927
C35 0.586871 -0.836481 -1.955976
C36 1.219350 -2.080627 -1.674044
C37 2.220612 -2.426417 -0.701952
C38 2.802316 -3.702575 -0.694507
C39 2.411122 -4.700505 -1.578901
C40 1.406146 -4.404344 -2.518494
C41 0.842623 -3.148534 -2.556723
H42 0.081554 -2.933878 -3.303417
H43 1.077755 -5.161496 -3.225978
H44 2.876385 -5.680665 -1.544826
H45 3.584882 -3.910122 0.031314
S46 2.831683 -1.319605 0.567038
C47 2.181611 -2.125929 2.087582
H48 2.247957 -1.384991 2.886869
H49 1.139303 -2.414002 1.939946
H50 2.776004 -3.007991 2.331427
H51 -0.105452 -0.887707 -2.798418
H52 2.012894 4.027147 0.015596
C53 1.718147 2.308097 4.684093
C54 3.071624 2.652546 4.038815
C55 3.292568 1.448457 3.128790
H56 3.878563 2.783735 4.765228
H57 2.964694 3.560979 3.440214
C58 0.950196 1.665500 3.524598
H59 1.851853 1.597070 5.507599
H60 1.196378 3.187711 5.070223
O61 1.969068 1.127133 2.625859
H62 0.298113 0.840912 3.826993
H63 0.383492 2.407928 2.961271
H64 3.675594 0.581194 3.685200
H65 3.928199 1.625309 2.259203
C66 -0.408100 -0.319612 1.183228
O67 -1.349968 -0.923738 0.975637
C68 3.441660 3.397575 0.144318
H69 3.417038 3.676874 1.200695
C70 5.388906 6.119786 -2.525803
C71 4.361331 5.289806 -2.989907
H72 4.052067 5.333847 -4.031070
C73 3.722072 4.417375 -2.115573
C74 5.761638 6.084419 -1.181370
H75 5.889063 6.800567 -3.210101
H76 6.555120 6.732509 -0.818077
C77 5.114457 5.215534 -0.299443
H78 2.899735 3.791906 -2.456534
C79 4.107880 4.366470 -0.766719
H80 5.403217 5.181434 0.748843
N81 3.323943 2.109051 -0.174143
B82 4.081112 1.486349 -1.226191
C83 4.565194 0.177596 -3.026572
C84 5.889368 0.617721 -2.302767
O85 3.563724 0.487192 -2.023784
C86 4.246155 1.041517 -4.253706
C87 4.464064 -1.303950 -3.372690
O88 5.426081 1.702982 -1.453551
C89 6.983010 1.151050 -3.222587
C90 6.445876 -0.471613 -1.376484
H91 7.862784 1.422625 -2.631539
H92 7.281325 0.388115 -3.950101
H93 6.650186 2.040581 -3.760228
H94 5.662742 -0.867657 -0.723113
H95 6.876418 -1.300593 -1.947181
H96 7.229437 -0.037141 -0.748883
H97 4.329575 2.105766 -4.014692
H98 4.915400 0.814131 -5.089347
H99 3.216849 0.848900 -4.564657
H100 3.494879 -1.505311 -3.837480
H101 5.253317 -1.590252 -4.076287
H102 4.532736 -1.932479 -2.484007

================================

6-TS'

================================

Mn	-2.673910423	0.008975325	-1.177273716
C	-4.261847689	0.556733313	0.699917957
O	-4.976157393	1.003107989	1.461969846
N	-1.435307203	-1.401351158	-0.210646064
C	-1.587727486	-1.190495336	1.190266965
C	-2.643520689	-1.737889656	1.960972488
C	-2.656433200	-1.484205744	3.347443519
C	-1.699582865	-0.677749672	3.950083016
C	-0.691869316	-0.089848947	3.175196919
C	-0.648395806	-0.349564921	1.812372751
H	0.124298776	0.081760400	1.183236009
H	0.056436162	0.550273537	3.633418469
H	-1.746977234	-0.497452430	5.020301849
H	-3.455625646	-1.912574701	3.946653859
S	-4.079843308	-2.621131891	1.350893557
C	-1.409560919	-2.693343288	-0.593529627
C	-0.916392889	-3.183636573	-1.919896506
C	-0.825702794	-2.470151578	-3.135429907
C	-0.393567819	-3.128733685	-4.296746181
C	-0.045841252	-4.476862194	-4.282721874
C	-0.129745729	-5.191106366	-3.087640577
C	-0.558955276	-4.545300427	-1.934099292
H	-0.639915302	-5.109132670	-1.008258953
H	0.133061254	-6.244102497	-3.052756617
H	0.283543024	-4.961817333	-5.196837145
H	-0.337966442	-2.563409486	-5.222143856
S	-1.283276384	-0.744373114	-3.360160198
C	0.234810345	0.105053451	-2.772166252
H	0.054098738	1.173905910	-2.902931056
H	0.386352720	-0.132614338	-1.717638431
H	1.088344850	-0.210007183	-3.374825084

H -1.175527523 -3.407805537 0.202353525
H -3.393110409 1.617444500 -3.770868586
C 0.127895750 4.532270398 -4.102670398
C -0.072530594 4.656922599 -5.482750664
C -1.143419552 3.975088222 -6.079424832
H 0.592307923 5.271080771 -6.083133016
H 0.956275176 5.053829586 -3.628581937
C -0.716539584 3.750784202 -3.325530196
H -0.547597778 3.679762190 -2.257797877
C -1.811792667 3.054156365 -3.909396506
H -1.309552180 4.059449395 -7.150722371
C -1.992135691 3.190704534 -5.315167937
H -2.817160874 2.663154572 -5.789477735
C -2.689791836 2.202652149 -3.177805964
N -2.647219239 1.918310529 -1.834572632
C -1.949142734 3.460475824 1.377904355
C -2.587167128 4.660133443 0.579033118
O -2.478665837 4.217012197 -0.796529333
C -1.852706168 5.989702289 0.723306645
C -4.082467368 4.841919245 0.872457218
O -2.251567762 2.336328313 0.515542421
C -0.421430877 3.544944360 1.474765913
C -2.554846439 3.202773639 2.754422974
B -2.448703404 2.835250393 -0.778851074
H 0.022001110 3.726258742 0.491052406
H -0.101980517 4.340132462 2.154839688
H -0.043978748 2.590505531 1.850512072
H -2.082268511 2.324611375 3.202134853
H -2.391043206 4.062768197 3.412438034
H -3.626506285 3.009089052 2.693015283
H -2.361960139 6.756520324 0.132470827
H -1.840807266 6.313779954 1.769631591
H -0.824175798 5.917510799 0.365225644
H -4.616193814 3.890411143 0.790448961
H -4.253143000 5.251895421 1.872558649
H -4.503173228 5.531527560 0.135572699
B -3.991293473 -2.824940192 -0.554141639
O -4.047517951 -1.642996078 -1.433912137
O -4.974310326 -3.726651091 -0.991175735
C -5.243553520 -1.772962091 -2.259483137
C -5.407810952 -3.329782875 -2.306415422
C -6.386053846 -1.068056171 -1.520731044
C -4.985786297 -1.103714263 -3.604292505
C -4.477969308 -3.988164298 -3.336927648
C -6.845018045 -3.803474380 -2.506742602
H -3.452805546 -3.622142389 -3.237551688
H -4.470937507 -5.066707565 -3.159099299
H -4.816850785 -3.807211701 -4.361745077
H -6.869893531 -4.896050855 -2.546626083
H -7.485549900 -3.481093071 -1.683995788
H -7.253223274 -3.417322125 -3.447195946
H -7.311596741 -1.090260033 -2.103325303
H -6.565831770 -1.533259825 -0.548672057
H -6.112471003 -0.021205381 -1.357891607
H -4.091192580 -1.496810061 -4.088591585
H -5.840601122 -1.249918751 -4.272525839
H -4.850947546 -0.026549046 -3.462309051
H -2.735982984 -3.249306100 -0.716416578
==============================

6-TS"
==============================
Mn -2.572215288 0.182683712 -1.143417114
C -0.702189830 1.193461391 -0.151475974
O 0.367572675 1.240991858 0.252728154
N -1.444601949 -1.457898673 -0.654725616
C -1.660274624 -1.852546798 0.691194775
C -2.971375981 -1.968714465 1.222334292
C -3.140279616 -2.355004919 2.558269062
C -2.046552669 -2.610426826 3.386015188
C -0.753297488 -2.476958638 2.872787471
C -0.567398848 -2.105080597 1.544055475
H 0.435633684 -1.971054139 1.149198934
H 0.111306171 -2.653853109 3.507009272
H -2.203476027 -2.903877689 4.419897664
H -4.151603150 -2.441873724 2.943111612
S -4.432531780 -1.648308015 0.222132373
C -0.552709262 -2.187879932 -1.344841168
C -0.067979094 -2.052817907 -2.687981297
C -0.230542453 -0.994353208 -3.641612947
C 0.286688501 -1.116961162 -4.937399657
C 0.998320199 -2.239429263 -5.349222772
C 1.199993245 -3.278079004 -4.426598654
C 0.684695970 -3.179343449 -3.149879703
H 0.839056218 -4.001166489 -2.454776944
H 1.754958698 -4.167223917 -4.713750959
H 1.380781707 -2.307075021 -6.362894150
H 0.116660540 -0.302827217 -5.636985287
S -1.008792484 0.588800087 -3.306647313
C 0.499640660 1.610766619 -3.039020362
H 0.176908007 2.619118804 -2.767294263
H 1.120697409 1.194743379 -2.244027035
H 1.070061009 1.649815506 -3.969053205
H -0.186654464 -3.082986781 -0.839934315
H -4.948804001 2.106013464 -1.080036688
C -8.135454901 1.828302441 -4.085166914
C -9.228053287 1.928144354 -3.222221052
C -9.042585048 1.764511396 -1.847668054
H -10.219269114 2.130984473 -3.618411391
H -8.271629098 1.956058343 -5.155741836
C -6.864801307 1.564551106 -3.577666388
H -6.012787464 1.493633974 -4.245695324
C -6.672546516 1.400270122 -2.199302770
H -9.890185405 1.832277905 -1.171327636
C -7.771609784 1.506730937 -1.338096999
H -7.626964934 1.358780849 -0.271711283
C -5.283770886 1.244620358 -1.663006902
N -4.303321843 0.582824326 -2.283526337
C -3.845590144 -2.495715879 -4.157208711
C -5.387700130 -2.247850664 -4.372271147
O -5.688485604 -1.236624745 -3.371532602
C -6.275316115 -3.459719483 -4.114986058
C -5.711686351 -1.620932183 -5.733663793
O -3.408512301 -1.204015113 -3.653836556
C -3.547317463 -3.531023945 -3.068329113
C -3.054851544 -2.813607775 -5.422545288
B -4.512855041 -0.583865635 -3.095283699
H -4.090758738 -3.301055641 -2.149345438
H -3.810871826 -4.540290555 -3.398982614

H -2.479370129 -3.503161661 -2.841644711
H -2.000025449 -2.947202353 -5.170251220
H -3.422215461 -3.737856396 -5.881437731
H -3.125126687 -2.006288718 -6.154123896
H -7.322439999 -3.190320002 -4.280996628
H -6.017874481 -4.277935681 -4.796198202
H -6.174227683 -3.811258417 -3.087065351
H -5.066666845 -0.758763336 -5.928296614
H -5.588108998 -2.339490543 -6.549368447
H -6.748848192 -1.274402072 -5.723326914
B -4.822769555 0.177440771 0.744597666
O -3.626254306 1.008702084 0.646536329
O -5.455157763 0.468842443 1.967075376
C -3.522104584 1.829690072 1.843205344
C -4.995823580 1.760368032 2.404618487
C -2.501320909 1.180061741 2.781766465
C -3.061021592 3.228134181 1.438367234
C -5.916076885 2.836478594 1.804721928
C -5.084462452 1.809406974 3.929246178
H -5.869587913 2.852431754 0.713494161
H -6.947237097 2.610663413 2.090344366
H -5.666047004 3.836098723 2.173720113
H -6.133805655 1.774226705 4.235218872
H -4.571697421 0.959506727 4.381729671
H -4.645699193 2.735725751 4.316147633
H -2.339214346 1.796000540 3.671698445
H -2.824837068 0.185846750 3.089311894
H -1.544093850 1.069253769 2.269146193
H -3.678539393 3.636496786 0.635848088
H -3.102433993 3.909870755 2.294385415
H -2.026597319 3.195382302 1.085028063
H -5.564657976 0.494035860 -0.326734695

===================================

6

===================================

Mn1 -1.318165 0.075284 -1.442982
C2 -1.364095 -2.962047 -1.667899
O3 -1.854116 -3.944631 -1.282780
N4 0.482811 -0.838878 -0.259768
C5 0.300974 -0.624376 1.135824
C6 -1.021751 -0.560361 1.642945
C7 -1.166318 -0.421213 3.036409
C8 -0.066246 -0.326419 3.883540
C9 1.233062 -0.345871 3.364147
C10 1.406981 -0.496377 1.991016
H11 2.406052 -0.481793 1.563243
H12 2.094313 -0.232063 4.015369
H13 -0.223469 -0.211941 4.952933
H14 -2.171795 -0.385999 3.444721
S15 -2.447096 -0.704070 0.608013
C16 1.374029 -1.709993 -0.596873
C17 1.724534 -2.195060 -1.935025
C18 1.387075 -1.623875 -3.188283
C19 1.678763 -2.322710 -4.366480
C20 2.311671 -3.565969 -4.337554
C21 2.693805 -4.114594 -3.113349
C22 2.409576 -3.428516 -1.938642
H23 2.691667 -3.867445 -0.984841
H24 3.206310 -5.070768 -3.071382
H25 2.525456 -4.086244 -5.266306
H26 1.418969 -1.867968 -5.317599
S27 0.548569 -0.060524 -3.406826
C28 1.663719 1.134196 -2.565714
H29 1.418526 2.104728 -2.999804
H30 1.479564 1.151107 -1.491164
H31 2.698466 0.870168 -2.790534
H32 1.891333 -2.250688 0.203488
H33 -2.429986 1.605823 -3.781764
C34 0.679144 4.839157 -4.747458
C35 0.392429 4.737966 -6.114328
C36 -0.617025 3.854571 -6.528162
H37 0.935814 5.335652 -6.840105
H38 1.447301 5.530853 -4.408144
C39 -0.005331 4.076650 -3.808981
H40 0.207279 4.210419 -2.755586
C41 -1.019063 3.158082 -4.209255
H42 -0.857982 3.764671 -7.585046
C43 -1.306615 3.087351 -5.603206
H44 -2.079125 2.398621 -5.941215
C45 -1.708123 2.280734 -3.319945
N46 -1.442901 2.031769 -2.003779
C47 -0.018099 3.425041 1.019661
C48 -0.940831 4.603287 0.530883
O49 -1.173219 4.265251 -0.858337
C50 -0.304566 5.988347 0.597190
C51 -2.310265 4.605872 1.224211
O52 -0.457501 2.333591 0.180494
C53 1.467374 3.653035 0.705281
C54 -0.197969 3.037866 2.483326
B55 -1.040545 2.888850 -0.962569
H56 1.606520 3.935901 -0.342937
H57 1.905293 4.431145 1.338168
H58 2.005466 2.717027 0.882611
H59 0.485715 2.226147 2.740477
H60 0.014323 3.892435 3.135522
H61 -1.211845 2.686158 2.677321
H62 -1.012655 6.736462 0.228771
H63 -0.040568 6.244021 1.629195
H64 0.594789 6.044025 -0.019410
H65 -2.762513 3.610239 1.192050
H66 -2.236068 4.923328 2.268931
H67 -2.970387 5.298317 0.694317
C68 -4.946187 -1.584526 -3.568521
C69 -3.825410 -2.323622 -4.319509
C70 -2.645787 -1.364440 -4.157049
H71 -3.605975 -3.278077 -3.826956
H72 -4.059267 -2.523629 -5.368521
C73 -4.192475 -1.010549 -2.370614
H74 -5.768113 -2.238603 -3.265686
H75 -5.355799 -0.779118 -4.188351
O76 -2.856938 -0.722610 -2.874349
H77 -4.101680 -1.733841 -1.552574
H78 -4.600281 -0.078041 -1.974364
H79 -1.673512 -1.864051 -4.133078
H80 -2.624338 -0.587014 -4.929824

===============================

^{3}6

===============================

Mn -0.902484880 -0.248558834 -1.563958274
C -1.765170375 -2.968952059 -1.292858941
O -2.074216163 -3.674130712 -0.454833838
N 0.215241037 -1.082108466 -0.152136427
C -0.052751837 -0.687085210 1.175263937
C -1.319337517 -0.113439193 1.455064814
C -1.642188307 0.225219314 2.773559686
C -0.721088341 0.048722921 3.809813612
C 0.544096760 -0.468573518 3.527524391
C 0.874892959 -0.831602285 2.221584415
H 1.870479855 -1.204505035 2.001225898
H 1.280597800 -0.581204740 4.318750446
H -0.987944918 0.329099712 4.825337284
H -2.625088013 0.640324919 2.979926689
S -2.437657683 0.164351471 0.109008600
C 1.038681065 -2.151517362 -0.316867718
C 1.514805088 -2.734074779 -1.520503143
C 1.487062764 -2.166792227 -2.840180879
C 1.917999975 -2.894134033 -3.954550101
C 2.410271649 -4.190419888 -3.827720644
C 2.505892566 -4.752421440 -2.538193379
C 2.083689977 -4.046011127 -1.431552019
H 2.149365245 -4.503592837 -0.446996881
H 2.910745072 -5.753065330 -2.410853349
H 2.732958754 -4.744716943 -4.703291776
H 1.876082827 -2.420683127 -4.932376631
S 0.957512749 -0.488166821 -3.118054202
C 2.253634895 0.470669030 -2.234497469
H 1.922792531 1.509031203 -2.204842891
H 2.329090972 0.097055965 -1.213578014
H 3.199148615 0.368004225 -2.770183528
H 1.263002260 -2.722820273 0.582730498
H -1.453579902 1.333487335 -4.143411856
C -1.129140212 5.877707690 -4.561538217
C -1.258060056 5.741250466 -5.947115888
C -1.380267751 4.471787739 -6.513952120
H -1.268914080 6.622900238 -6.581756907
H -1.046717302 6.867039219 -4.120613911
C -1.114865540 4.756176064 -3.737790846
H -1.046783770 4.874533500 -2.663769813
C -1.232679029 3.471018217 -4.298621214
H -1.483989564 4.359311870 -7.589246508
C -1.371981876 3.347657978 -5.693060778
H -1.465432569 2.356260157 -6.130531578
C -1.216333611 2.211588258 -3.534024183
N -0.942540002 1.967690830 -2.308262523
C 0.642760390 3.596033761 0.552178109
C -0.792891118 4.263940206 0.574553978
O -1.337241205 3.893877563 -0.731468148
C -0.787184057 5.787581882 0.664736805
C -1.731861952 3.671303389 1.625697242
O 0.487191804 2.529273852 -0.425439729
C 1.735924404 4.517606902 -0.000507817
C 1.076290822 2.968741730 1.872870034
B -0.621309357 2.815089676 -1.175291854
H 1.444372027 4.940567769 -0.967255137
H 1.958933018 5.338294188 0.687506666
H 2.647843959 3.932639246 -0.149284729
H 2.068769300 2.523950893 1.758087146
H 1.130363702 3.729039135 2.659527407
H 0.392846500 2.178743160 2.184309063
H -1.815789434 6.157839749 0.639990179
H -0.329849371 6.113294543 1.604655639
H -0.241583231 6.243862732 -0.163738361
H -1.782911090 2.586387508 1.537945211
H -1.407980726 3.933387132 2.637135560
H -2.737617614 4.070118109 1.465615415
C -4.479447085 -1.112417429 -4.069449662
C -3.478093428 -2.230345686 -4.406661087
C -2.137865505 -1.525836229 -4.211881447
H -3.572100300 -3.052949345 -3.689128467
H -3.595577336 -2.635275198 -5.415445103
C -3.792481812 -0.422198105 -2.892303898
H -5.472362821 -1.485909848 -3.804966205
H -4.586192516 -0.425307915 -4.917057765
O -2.365885084 -0.570294546 -3.142381373
H -4.015130129 -0.905231007 -1.936268658
H -3.999034836 0.647366797 -2.802852744
H -1.327375774 -2.188421202 -3.902818161
H -1.828936950 -0.971317581 -5.107989912

=================================

^{1}6

=================================

Mn -1.079457044 -0.726965092 -1.655553113
C -1.414521633 -2.467010109 -1.512538530
O -1.684063077 -3.599294246 -1.422328168
N 0.412533460 -0.950952837 -0.289499305
C 0.194018020 -0.403738474 1.012634769
C -1.143133535 -0.134775715 1.400963023
C -1.369757665 0.201782291 2.755507013
C -0.324179997 0.342427720 3.659173415
C 1.002628358 0.186242839 3.232979592
C 1.249079802 -0.181178329 1.915846880
H 2.274587474 -0.268865310 1.569672848
H 1.829751500 0.363750669 3.913733518
H -0.537973341 0.609167462 4.691225753
H -2.395158269 0.357226014 3.078513511
S -2.450275045 -0.208243276 0.246741180
C 1.425600691 -1.779822481 -0.376383525
C 1.876679638 -2.561647226 -1.518551268
C 1.519719493 -2.374044081 -2.875018779
C 1.977176214 -3.242458626 -3.864841144
C 2.806446916 -4.318193268 -3.541710644
C 3.204941760 -4.498864516 -2.216174280
C 2.753734577 -3.630070714 -1.229532163
H 3.061071175 -3.785170092 -0.198293765
H 3.864233636 -5.320005019 -1.949913379
H 3.146036912 -4.996270428 -4.318859702
H 1.685286142 -3.063817764 -4.895539825
S 0.538823442 -0.964787065 -3.377833927
C 1.814823555 0.345072046 -3.103574023
H 1.499332058 1.239629272 -3.640716683
H 1.883672334 0.561417592 -2.036359072
H 2.768426111 -0.004546580 -3.503005457
H 1.955333799 -2.028915602 0.541889933
H -1.403512090 1.051683133 -4.047539419
C 0.392198682 5.220858236 -4.008311882
C -0.459776997 5.692271894 -5.010998645

C -1.507917921 4.887542291 -5.467270013
H -0.299732575 6.675094086 -5.444856438
H 1.224425998 5.830708366 -3.667765178
C 0.183942147 3.961959059 -3.449448804
H 0.855554364 3.584307590 -2.685638511
C -0.882702109 3.159072124 -3.882565105
H -2.165982885 5.246128594 -6.253656503
C -1.706578773 3.621611922 -4.919964965
H -2.516548828 2.991329017 -5.280118404
C -1.097311282 1.811063365 -3.324117619
N -0.927076974 1.426014916 -2.109177518
C 0.144395285 3.744762415 0.564862254
C -1.391730212 4.094710861 0.375695616
O -1.742406447 3.373007677 -0.836671287
C -1.680616161 5.576591371 0.139838387
C -2.289396774 3.548007418 1.488203186
O 0.302651306 2.519841378 -0.218254503
C 1.094876247 4.785320612 -0.040421421
C 0.555596728 3.454255134 2.002665283
B -0.786049186 2.417543037 -1.035590780
H 0.815309255 5.049669405 -1.061162289
H 1.107466572 5.700733425 0.558212090
H 2.106624258 4.369606037 -0.056509303
H 1.615601892 3.187588902 2.035054008
H 0.405162549 4.341993725 2.626935112
H -0.008316864 2.623744434 2.421454783
H -2.756854543 5.720632240 0.008863140
H -1.358326985 6.175062744 0.998416282
H -1.180447741 5.947544555 -0.756776277
H -2.133535842 2.477688833 1.625832245
H -2.109216188 4.065186943 2.435391350
H -3.333779158 3.697965443 1.201086869
C -4.680659115 0.228578508 -3.998761771
C -4.387483210 -1.158040721 -4.605266877
C -2.964552264 -1.474075725 -4.103304311
H -5.098614837 -1.893223712 -4.214546098
H -4.455612168 -1.174705228 -5.696437518
C -3.923013693 0.151657351 -2.678596218
H -5.748506840 0.423192819 -3.867160872
H -4.259565327 1.025027864 -4.624506088
O -2.702019317 -0.530984490 -3.036034245
H -4.461072526 -0.437146246 -1.924858607
H -3.634274028 1.106542145 -2.238415362
H -2.864935185 -2.489836842 -3.710306450
H -2.191306436 -1.316380120 -4.862135005

================================

7-TS

================================

Mn1 1.530776 0.994120 0.502949
N2 0.780345 -0.002171 -1.197371
C3 0.065014 0.794580 -2.103874
C4 -0.496350 2.039421 -1.716950
C5 -1.119902 2.880379 -2.639149
C6 -1.225499 2.515503 -3.978508
C7 -0.699194 1.287150 -4.381514
C8 -0.066804 0.452354 -3.469957
H9 0.402988 -0.454562 -3.829926
H10 -0.755724 0.984997 -5.424206
H11 -1.707041 3.178390 -4.691009
H12 -1.518742 3.829021 -2.293987
S13 -0.489841 2.545676 0.004232
B14 0.680917 4.001187 0.136065
O15 0.944456 4.926539 -0.833256
C16 0.851500 6.232535 -0.185855
C17 1.068965 5.902835 1.353941
O18 0.767750 4.477510 1.424777
C19 0.098005 6.616045 2.296266
H20 -0.940951 6.373816 2.067467
H21 0.299056 6.305760 3.326314
H22 0.227989 7.701636 2.238616
C23 2.501196 6.108517 1.846194
H24 2.582900 5.732688 2.870649
H25 3.222561 5.576687 1.229473
H26 2.766656 7.170134 1.853548
C27 1.897728 7.163380 -0.792514
H28 1.830008 8.157960 -0.338283
H29 2.910761 6.788220 -0.654987
H30 1.712163 7.264623 -1.865687
C31 -0.556785 6.752415 -0.507555
H32 -1.325702 6.074222 -0.126378
H33 -0.728882 7.748895 -0.090052
H34 -0.667525 6.807079 -1.593986
C35 0.937441 -1.307756 -1.530264
C36 1.960465 -2.239643 -1.183478
C37 3.015810 -2.156318 -0.210911
C38 4.039166 -3.113840 -0.173373
C39 4.052631 -4.209699 -1.027473
C40 2.990062 -4.361541 -1.936493
C41 1.988176 -3.417503 -2.000795
H42 1.196464 -3.537007 -2.736620
H43 2.961984 -5.217516 -2.605774
H44 4.859142 -4.934626 -0.981573
H45 4.831483 -2.990668 0.560217
S46 3.055324 -0.975984 1.131694
C47 2.155149 -1.931533 2.422595
H48 1.821184 -1.224689 3.183234
H49 1.286104 -2.413079 1.972035
H50 2.819337 -2.682510 2.853934
H51 0.227769 -1.707684 -2.254547
H52 3.377704 3.146177 1.761022
C53 0.820494 2.777316 4.655706
C54 2.345527 2.762885 4.431113
C55 2.539410 1.423681 3.731252
H56 2.920456 2.845847 5.357929
H57 2.639707 3.573030 3.756928
C58 0.272661 1.987945 3.447675
H59 0.565932 2.276250 5.598077
H60 0.411482 3.792817 4.689458
O61 1.419367 1.340686 2.823419
H62 -0.450304 1.214616 3.734756
H63 -0.170406 2.643123 2.698887
H64 2.495797 0.588495 4.447244
H65 3.449960 1.336821 3.137935
C66 -0.550382 -0.518273 1.263696
O67 -1.633630 -0.827869 1.008522
C68 3.877356 2.766150 0.859699
H69 4.458481 1.880259 1.172770
C70 6.745696 5.806543 -0.312291

C71 5.527182 5.697359 -0.984537
H72 5.291954 6.375367 -1.801643
C73 4.604448 4.717052 -0.615479
C74 7.032157 4.924443 0.731738
H75 7.467206 6.566247 -0.601040
H76 7.980935 4.993993 1.258382
C77 6.108506 3.942754 1.093144
H78 3.652583 4.636068 -1.131434
C79 4.884737 3.824817 0.425896
H80 6.346534 3.246917 1.896090
N81 2.847053 2.438962 -0.122717
B82 3.370393 2.095336 -1.404466
C83 3.637671 2.044138 -3.672809
C84 4.793099 1.231978 -2.979415
O85 2.674295 2.182718 -2.603636
C86 4.066031 3.462187 -4.081535
C87 2.979484 1.342925 -4.856959
O88 4.679242 1.656530 -1.604566
C89 6.199419 1.557764 -3.477081
C90 4.548661 -0.282209 -3.008891
H91 6.929943 0.943430 -2.941574
H92 6.293527 1.345309 -4.547990
H93 6.448796 2.605893 -3.300972
H94 3.535315 -0.519231 -2.676258
H95 4.695888 -0.698377 -4.010688
H96 5.247063 -0.771020 -2.324085
H97 4.578776 3.968768 -3.260455
H98 4.730389 3.449679 -4.951832
H99 3.170234 4.037783 -4.332670
H100 2.153533 1.954440 -5.230576
H101 3.703127 1.197627 -5.666819
H102 2.570164 0.373735 -4.572240
================================
7
================================
Mn1 1.967688 1.009179 0.264827
N2 0.910139 0.542188 -1.498346
C3 0.486741 1.655360 -2.236658
C4 0.003740 2.824351 -1.588926
C5 -0.439473 3.934913 -2.305693
C6 -0.380006 3.941976 -3.699517
C7 0.129477 2.824343 -4.364760
C8 0.551440 1.708860 -3.650761
H9 0.985004 0.870169 -4.181580
H10 0.210716 2.822841 -5.448558
H11 -0.720385 4.812786 -4.251658
H12 -0.809913 4.801065 -1.771531
S13 -0.027222 2.778578 0.211099
B14 0.429267 4.446600 0.807157
O15 0.286938 5.617370 0.136015
C16 0.780019 6.676151 1.026321
C17 0.668995 5.992984 2.441488
O18 0.819641 4.571535 2.114700
C19 -0.715104 6.141678 3.082154
H20 -1.506798 5.861375 2.381280
H21 -0.779985 5.480556 3.950940
H22 -0.892963 7.168588 3.414286
C23 1.765993 6.377522 3.426437
H24 1.613184 5.854587 4.375030
H25 2.754215 6.115962 3.045261
H26 1.740713 7.454279 3.623587
C27 2.216785 6.981033 0.607375
H28 2.616107 7.831631 1.168264
H29 2.870774 6.121650 0.756246
H30 2.240811 7.217788 -0.458085
C31 -0.110115 7.898792 0.832323
H32 -1.167082 7.655366 0.954194
H33 0.156030 8.682087 1.549789
H34 0.033732 8.297355 -0.175493
C35 0.796403 -0.655889 -2.129027
C36 1.364227 -1.937511 -1.871994
C37 2.198818 -2.415319 -0.803059
C38 2.720031 -3.715216 -0.825996
C39 2.425603 -4.617214 -1.842683
C40 1.578499 -4.194070 -2.882211
C41 1.074769 -2.911677 -2.887652
H42 0.437675 -2.601177 -3.712518
H43 1.325226 -4.871668 -3.693581
H44 2.846094 -5.617774 -1.832338
H45 3.378240 -4.017585 -0.015655
S46 2.629423 -1.465695 0.651387
C47 1.552761 -2.242822 1.923221
H48 1.487665 -1.533517 2.750943
H49 0.556078 -2.429755 1.521795
H50 1.995253 -3.182055 2.261545
H51 0.147220 -0.656933 -3.004335
H52 3.483811 3.575779 1.315564
C53 1.205124 2.329310 4.496068
C54 2.673245 2.546682 4.073319
C55 2.977507 1.276245 3.286780
H56 3.348721 2.693465 4.920942
H57 2.735566 3.410684 3.407963
C58 0.636956 1.408334 3.392006
H59 1.146909 1.840778 5.474046
H60 0.654020 3.271131 4.548711
O61 1.756049 1.040006 2.547235
H62 0.187805 0.494125 3.797782
H63 -0.095954 1.903243 2.754708
H64 3.167743 0.419154 3.950073
H65 3.775676 1.355323 2.546955
C66 -0.749698 -0.313840 0.862594
O67 -1.616550 -0.753907 0.258006
C68 4.188108 3.215288 0.549132
H69 5.177710 3.193714 1.034466
C70 4.193983 6.242504 -2.556981
C71 3.236415 5.224834 -2.560973
H72 2.467625 5.189749 -3.328406
C73 3.258270 4.242478 -1.572457
C74 5.168831 6.264512 -1.557241
H75 4.185723 7.007217 -3.329647
H76 5.925252 7.045737 -1.552091
C77 5.181505 5.278871 -0.567876
H78 2.528155 3.443010 -1.588080
C79 4.224354 4.259489 -0.560459
H80 5.947740 5.296778 0.204824
N81 3.770110 1.893459 0.112545
B82 4.551917 1.246266 -0.854109
C83 4.915800 -0.181010 -2.603108

C84 6.204784 0.683080 -2.333247
O85 4.186339 -0.006848 -1.370372
C86 4.040295 0.385648 -3.729958
C87 5.160200 -1.670418 -2.828506
O88 5.707165 1.725271 -1.464612
C89 6.811048 1.334756 -3.573762
C90 7.275901 -0.088350 -1.551370
H91 7.693728 1.916469 -3.290480
H92 7.118099 0.576400 -4.302738
H93 6.100486 2.014054 -4.048409
H94 6.836455 -0.567542 -0.671550
H95 7.756535 -0.856193 -2.166031
H96 8.040653 0.616073 -1.211411
H97 3.836651 1.449122 -3.572214
H98 4.504700 0.255509 -4.712800
H99 3.086500 -0.147228 -3.717578
H100 4.206325 -2.180126 -2.988472
H101 5.792225 -1.828365 -3.709551
H102 5.640647 -2.130149 -1.963022
================================
^{3}7
================================

Mn	1.806398420	0.974764260	0.303164440
N	0.951970538	0.584706092	-1.444446841
C	0.513234131	1.703677363	-2.183974192
C	-0.047960533	2.829559351	-1.533919414
C	-0.532898591	3.928280337	-2.239150521
C	-0.425214988	3.968031825	-3.629886477
C	0.177906182	2.899565219	-4.294331176
C	0.639096679	1.792520200	-3.588823387
H	1.146455010	0.997832946	-4.121339294
H	0.305572086	2.930199503	-5.373233747
H	-0.794434571	4.829377274	-4.178435039
H	-0.964977613	4.761477634	-1.699063132
S	-0.088366988	2.747147443	0.258782357
B	0.424325232	4.404577107	0.850469529
O	0.313398903	5.564861005	0.155627572
C	0.834703108	6.630585308	1.020537960
C	0.720978696	5.977969682	2.452317150
O	0.813803497	4.545112900	2.155475719
C	-0.645357972	6.190904209	3.111455126
H	-1.457460642	5.915846348	2.432180911
H	-0.716634643	5.557242867	4.000007304
H	-0.784035633	7.232203548	3.416238898
C	1.847959490	6.338077183	3.412741559
H	1.697327087	5.825781410	4.367435254
H	2.819989464	6.042413826	3.015476066
H	1.860150882	7.416389905	3.601917124
C	2.273739675	6.900794473	0.586541613
H	2.693780230	7.753000327	1.129463664
H	2.911658304	6.032717161	0.746347058
H	2.296422503	7.116573837	-0.483729822
C	-0.031449432	7.867143936	0.807975075
H	-1.091856729	7.650721736	0.949156624
H	0.261539206	8.662123567	1.501695799
H	0.108315759	8.236894790	-0.211387597
C	0.898914517	-0.593584828	-2.127256592
C	1.460622450	-1.874088486	-1.875345432
C	2.217922920	-2.342053547	-0.754065639
C	2.764850715	-3.628038637	-0.736885040
C	2.567468281	-4.523732323	-1.783880677
C	1.797500558	-4.107735530	-2.884439784
C	1.267127544	-2.835133206	-2.922106541
H	0.689680257	-2.526756667	-3.790722247
H	1.625170513	-4.784100144	-3.717760326
H	3.006514060	-5.515826997	-1.751155208
H	3.364411332	-3.925214537	0.119902717
S	2.504195196	-1.343426531	0.694684957
C	1.414434807	-2.182052224	1.912706647
H	1.293016261	-1.490345407	2.748257631
H	0.446152975	-2.403338647	1.463591808
H	1.883916042	-3.108332140	2.249303494
H	0.306784261	-0.566961724	-3.039033903
H	3.368069164	3.577214250	1.271478235
C	1.298699041	2.303925483	4.518227578
C	2.756641797	2.512126595	4.057436615
C	3.030023880	1.245865002	3.253521618
H	3.456546469	2.654031692	4.885698982
H	2.803534899	3.377222107	3.393132917
C	0.693227604	1.406461959	3.415553849
H	1.260169479	1.800591963	5.489836894
H	0.759566684	3.250616505	4.599872100
O	1.788977426	1.030659567	2.544413063
H	0.238068744	0.493976111	3.818630293
H	-0.046076941	1.919366251	2.801502867
H	3.227231271	0.380946617	3.904705289
H	3.806273111	1.322723495	2.491258417
C	-0.928930870	-0.322326696	0.806956844
O	-1.616668357	-0.776667964	0.023302494
C	4.046690485	3.181566403	0.500062836
H	5.040316267	3.143913470	0.977705428
C	4.173770089	6.149063084	-2.658583211
C	3.180646543	5.165895949	-2.650551923
H	2.418470807	5.142393074	-3.425068141
C	3.159721836	4.202789990	-1.642745494
C	5.142378619	6.154967794	-1.652038101
H	4.199790004	6.898082097	-3.446266445
H	5.928421603	6.906572200	-1.657288650
C	5.112349400	5.188438779	-0.644974169
H	2.407668846	3.424979622	-1.649844514
C	4.117017895	4.206384352	-0.623726258
H	5.877986316	5.189625927	0.128644431
N	3.603659040	1.857548111	0.089848738
B	4.414051436	1.220163974	-0.866239793
C	4.886182681	-0.173321871	-2.613139191
C	6.145063830	0.710474461	-2.278782659
O	4.102440072	-0.031128749	-1.412186601
C	4.049535640	0.394452551	-3.769982642
C	5.168995420	-1.654336502	-2.847712782
O	5.585951559	1.731607810	-1.424100974
C	6.796070485	1.388033146	-3.482536189
C	7.194443054	-0.048382422	-1.456245905
H	7.656601520	1.978658243	-3.152897655
H	7.147203097	0.644647652	-4.207217025
H	6.097359938	2.063802653	-3.979241238
H	6.724291794	-0.548196465	-0.604288848
H	7.719953939	-0.797869154	-2.056751757
H	7.927038834	0.667492503	-1.072201231

H	3.826507194	1.453401616	-3.607685179
H	4.556899645	0.282884720	-4.733801191
H	3.103006750	-0.151338264	-3.805407716
H	4.233351294	-2.180086406	-3.054961858
H	5.841061887	-1.788018815	-3.702946406
H	5.621298759	-2.116364338	-1.968479664

==============================

[1]7

==============================

Mn	1.311748072	0.747833155	0.356036658
N	0.894451284	0.488708153	-1.579616684
C	0.601244098	1.662060634	-2.341818336
C	0.043388953	2.793608360	-1.715191766
C	-0.312825815	3.917345311	-2.462947240
C	-0.038679668	3.964136280	-3.829448417
C	0.599417161	2.888187581	-4.446001739
C	0.914325775	1.750375558	-3.708543949
H	1.459630961	0.942281016	-4.178550264
H	0.869228929	2.936494178	-5.497031527
H	-0.295221342	4.853255730	-4.398221536
H	-0.758000525	4.769991252	-1.966970234
S	-0.104792816	2.702540263	0.069400472
B	0.337094352	4.399096507	0.647095501
O	-0.070433151	5.556331214	0.061729271
C	0.509387972	6.667363066	0.821340400
C	0.922169562	5.976526526	2.188642152
O	0.974277653	4.557310619	1.842152345
C	-0.131515786	6.103011755	3.291488509
H	-1.109614793	5.759742250	2.943411805
H	0.164103859	5.476293200	4.137776700
H	-0.225238604	7.134971485	3.641094759
C	2.301471305	6.372168764	2.708801286
H	2.518467411	5.815441271	3.625416126
H	3.080537127	6.146605759	1.978602184
H	2.334214192	7.441268099	2.942657697
C	1.691556057	7.184643597	0.003620540
H	2.163414196	8.043947029	0.489427958
H	2.440190602	6.409904130	-0.147994455
H	1.334421363	7.497220337	-0.981052933
C	-0.560212455	7.748102530	0.951802451
H	-1.478445845	7.363802279	1.398963781
H	-0.191375705	8.578409882	1.562922573
H	-0.802872546	8.138994375	-0.040027500
C	0.834008676	-0.627235769	-2.249715830
C	1.311266866	-1.957646354	-1.904676450
C	2.098661755	-2.352864194	-0.794557479
C	2.579255385	-3.664417034	-0.714600621
C	2.270404768	-4.614581659	-1.683533268
C	1.482214278	-4.248241015	-2.776806777
C	1.025998761	-2.943528684	-2.879516825
H	0.433065883	-2.655517467	-3.744187617
H	1.237572826	-4.971426083	-3.549064505
H	2.652615627	-5.626996528	-1.591588819
H	3.216207003	-3.941955293	0.119707012
S	2.590522238	-1.219368305	0.502073840
C	1.923690367	-2.134585944	1.952550253
H	1.920892197	-1.415411627	2.772301191
H	0.900381565	-2.455524019	1.757122388
H	2.559035080	-2.986843358	2.199888092
H	0.395803511	-0.588712835	-3.247385675
H	2.808646548	3.271723222	1.168011218
C	1.792750241	2.294299983	4.529910125
C	3.211947801	2.328202638	3.926680984
C	3.180461449	1.119021613	2.998244842
H	4.000215292	2.263687425	4.682461596
H	3.358177751	3.239185767	3.339809048
C	0.931913920	1.703537742	3.393937593
H	1.768947795	1.647132430	5.413332208
H	1.439438503	3.284952117	4.829827361
O	1.859843229	1.161790128	2.426261512
H	0.263612728	0.902229729	3.729268103
H	0.345849053	2.466708567	2.883035752
H	3.307637682	0.182771932	3.564941788
H	3.879724947	1.131417936	2.164407320
C	-0.143393131	-0.164427532	0.847953187
O	-1.076759555	-0.814559704	1.130702329
C	3.551007802	2.944398049	0.434554680
H	4.482005318	2.779152109	1.008780433
C	4.425291323	6.321685640	-2.160085744
C	3.501561830	5.352393128	-2.556232666
H	2.997736758	5.436044895	-3.516269939
C	3.219198254	4.265657498	-1.727822019
C	5.062842202	6.192219989	-0.923605873
H	4.652458746	7.164469521	-2.808050483
H	5.793418748	6.933222359	-0.607154285
C	4.772937026	5.104457711	-0.097907677
H	2.530438175	3.495159124	-2.049231946
C	3.846654683	4.125697358	-0.484922254
H	5.292473003	4.995609746	0.852956130
N	3.075233370	1.735641399	-0.217101303
B	3.986775164	1.192156134	-1.116609662
C	4.852317454	-0.065910125	-2.843989835
C	6.007263702	0.704715038	-2.103981201
O	3.762690024	0.049272198	-1.915734113
C	4.427169777	0.631705111	-4.145111819
C	5.114413125	-1.547796632	-3.104667732
O	5.283896264	1.688534466	-1.352793599
C	7.002408516	1.418340379	-3.018477129
C	6.753637349	-0.185427880	-1.098297542
H	7.764897350	1.918000726	-2.412674012
H	7.505707121	0.709080022	-3.685581628
H	6.505526551	2.180914370	-3.621422207
H	6.043348308	-0.707714970	-0.450067144
H	7.389278865	-0.925977849	-1.595056263
H	7.384532880	0.450231255	-0.470127414
H	4.193087892	1.683397373	-3.956581111
H	5.202181862	0.573501872	-4.916062593
H	3.527810037	0.141758242	-4.530284984
H	4.240083585	-1.994411390	-3.588150619
H	5.980885368	-1.684523277	-3.761189849
H	5.287323587	-2.089607749	-2.173254782

==============================

8

==============================

Mn1 1.127143 1.014059 0.580701
N2 0.728641 0.065940 -1.218888
C3 -0.118542 0.819358 -2.067323
C4 -0.914394 1.871856 -1.534513

C5 -1.711061 2.625232 -2.407786
C6 -1.738302 2.373146 -3.777997
C7 -0.947818 1.351772 -4.305442
C8 -0.147801 0.592632 -3.457279
H9 0.509639 -0.162666 -3.877610
H10 -0.936575 1.156354 -5.374622
H11 -2.368969 2.974985 -4.427092
H12 -2.325014 3.417883 -1.991052
S13 -0.934492 2.250666 0.207328
B14 1.939688 3.987057 -0.034902
O15 1.560925 4.793685 -1.063639
C16 0.881407 5.953930 -0.519541
C17 1.180035 5.858208 1.045184
O18 1.686422 4.506492 1.213402
C19 -0.055518 6.010265 1.930393
H20 -0.811716 5.262900 1.684755
H21 0.230834 5.882006 2.979113
H22 -0.494513 7.006872 1.819740
C23 2.299020 6.787223 1.529598
H24 2.504934 6.573867 2.582874
H25 3.223341 6.615893 0.972394
H26 2.018730 7.840592 1.439969
C27 1.476335 7.187810 -1.201465
H28 1.025548 8.106032 -0.811161
H29 2.558346 7.244939 -1.065132
H30 1.273552 7.136282 -2.274802
C31 -0.597710 5.817089 -0.886932
H32 -1.021233 4.906126 -0.461331
H33 -1.174085 6.683554 -0.547721
H34 -0.680704 5.745057 -1.974594
C35 0.956055 -1.214774 -1.586207
C36 2.012639 -2.097767 -1.223403
C37 3.046528 -1.945707 -0.237792
C38 4.126800 -2.839689 -0.181494
C39 4.213758 -3.939361 -1.025346
C40 3.171129 -4.160888 -1.945829
C41 2.118258 -3.276677 -2.031750
H42 1.342888 -3.446314 -2.775110
H43 3.200491 -5.023772 -2.606134
H44 5.060192 -4.616060 -0.963777
H45 4.903328 -2.664287 0.558818
S46 2.993252 -0.755563 1.092729
C47 2.304313 -1.806855 2.439186
H48 1.966601 -1.148380 3.240360
H49 1.457135 -2.375981 2.054848
H50 3.077508 -2.486308 2.801723
H51 0.272460 -1.631125 -2.327192
H52 3.397778 2.904780 1.796979
C53 0.868765 3.094408 4.649402
C54 2.383288 2.874583 4.495182
C55 2.410044 1.522022 3.791610
H56 2.923353 2.867660 5.446079
H57 2.813238 3.644375 3.845651
C58 0.306024 2.511466 3.345580
H59 0.492344 2.547555 5.521076
H60 0.599454 4.147111 4.768671
O61 1.332732 1.599377 2.832451
H62 -0.619033 1.943510 3.476767
H63 0.162708 3.274107 2.582437
H64 2.215143 0.705597 4.502777
H65 3.331072 1.301516 3.248882
C66 -0.533162 -0.715109 1.301093
O67 -1.436253 -1.379118 1.098414
C68 3.830042 2.674503 0.823912
H69 4.192288 1.645850 0.853002
C70 7.259162 5.263844 0.254810
C71 6.092481 5.504477 -0.469586
H72 6.045738 6.335527 -1.168651
C73 4.976821 4.678429 -0.308560
C74 7.305247 4.185076 1.141117
H75 8.126896 5.905016 0.126922
H76 8.212181 3.980864 1.704283
C77 6.192813 3.360222 1.296562
H78 4.086013 4.879677 -0.894685
C79 5.014050 3.596480 0.578624
H80 6.240561 2.512409 1.977584
N81 2.739766 2.769580 -0.181584
B82 3.153960 2.323622 -1.510525
C83 3.539254 2.410094 -3.732629
C84 4.412463 1.266321 -3.083092
O85 2.575912 2.708762 -2.683212
C86 4.324659 3.706082 -3.975717
C87 2.774397 2.007388 -4.988359
O88 4.269295 1.538178 -1.657899
C89 5.898753 1.322190 -3.424787
C90 3.855454 -0.132800 -3.338989
H91 6.418713 0.497907 -2.927903
H92 6.048477 1.219324 -4.504895
H93 6.353167 2.257514 -3.093125
H94 2.795680 -0.177668 -3.089900
H95 3.988188 -0.426349 -4.384686
H96 4.375312 -0.853795 -2.703336
H97 4.890500 3.999661 -3.087135
H98 5.020482 3.602573 -4.814148
H99 3.614566 4.505294 -4.205083
H100 2.220309 2.870434 -5.368825
H101 3.463535 1.666781 -5.768503
H102 2.047396 1.224064 -4.773390

================================

^{3}8

================================

Mn	0.987421374	0.792714829	0.717401653
N	0.663594826	0.049428478	-1.083036444
C	-0.148886797	0.811257968	-1.964380648
C	-0.952940243	1.835888899	-1.419511793
C	-1.776234421	2.594744305	-2.258026293
C	-1.788385781	2.378367700	-3.635678857
C	-0.967662072	1.392403088	-4.185246173
C	-0.158294341	0.617239925	-3.358202131
H	0.499444147	-0.120818171	-3.803861987
H	-0.943315237	1.232438390	-5.259980989
H	-2.423404929	2.985525021	-4.275330573
H	-2.404642158	3.363873678	-1.819333963
S	-0.864110517	2.123049263	0.319645328
B	2.010406096	4.050266967	-0.109424749
O	1.606873251	4.876958012	-1.121543742
C	0.861575804	5.975495445	-0.541051886
C	1.279249148	5.915032107	0.987914852

O	1.723115171	4.541820119	1.151483928
C	0.137541940	6.174516336	1.967832366
H	-0.685915911	5.474523568	1.817822362
H	0.502899679	6.065208531	2.994203465
H	-0.246139635	7.193483452	1.852084466
C	2.485089133	6.796242059	1.335778207
H	2.795284142	6.581869242	2.362889556
H	3.334665512	6.578080442	0.683879239
H	2.244768348	7.860728597	1.261395574
C	1.283524270	7.254483679	-1.263580512
H	0.796401749	8.129904283	-0.821006790
H	2.364995320	7.400071149	-1.229440028
H	0.984231345	7.191133069	-2.313574010
C	-0.622516142	5.698632718	-0.789391632
H	-0.933118008	4.775184819	-0.297907903
H	-1.247561079	6.526167005	-0.439169721
H	-0.777572569	5.569821982	-1.863740697
C	0.990345982	-1.188384301	-1.539670418
C	2.074654055	-2.040140951	-1.206869576
C	3.066212566	-1.857488401	-0.193967086
C	4.192740518	-2.685043829	-0.114798952
C	4.363559187	-3.756214600	-0.984463998
C	3.368823773	-4.004135638	-1.950762726
C	2.269714364	-3.178561449	-2.053405947
H	1.530829257	-3.365707987	-2.829004210
H	3.473112440	-4.842832626	-2.634251695
H	5.242217828	-4.389395405	-0.912560453
H	4.935988457	-2.485089996	0.653253510
S	2.859098492	-0.665954877	1.111875555
C	2.321611725	-1.808690097	2.453583809
H	1.935358117	-1.214041607	3.280581979
H	1.532928000	-2.457136193	2.070799071
H	3.176366438	-2.405315123	2.776169371
H	0.367288821	-1.569894454	-2.345999031
H	3.418733893	2.910157980	1.714343992
C	0.752622633	3.074208642	4.524456009
C	2.260831191	2.830643062	4.359347853
C	2.260428524	1.485190214	3.645743269
H	2.807990735	2.802944043	5.305676750
H	2.697384827	3.599018171	3.712919814
C	0.187156951	2.552162372	3.201482849
H	0.364772355	2.504516553	5.376601449
H	0.502402161	4.127540620	4.676364087
O	1.170995064	1.582362819	2.694584017
H	-0.770336525	2.034816991	3.293797256
H	0.119387297	3.333262930	2.448139146
H	2.055615266	0.667415378	4.350449893
H	3.170421641	1.258823269	3.090110391
C	-0.623581793	-0.945963486	1.378156046
O	-1.531523152	-1.564732634	1.075701587
C	3.870608675	2.694172476	0.745559026
H	4.195477865	1.651920184	0.753540794
C	7.411896678	5.171304009	0.391685671
C	6.296872224	5.450506012	-0.397677887
H	6.317762081	6.283652908	-1.095575774
C	5.147616397	4.661307464	-0.302468837
C	7.371211690	4.092248225	1.278232059
H	8.306290062	5.783529110	0.315068981
H	8.236688708	3.859500231	1.893411589
C	6.224717101	3.305054169	1.368177343
H	4.293369065	4.890828661	-0.931734556
C	5.098585301	3.578145378	0.582354205
H	6.203791951	2.457144232	2.050843530
N	2.839520611	2.881953059	-0.289996662
B	3.205477056	2.393814891	-1.599122612
C	3.575945777	2.446728541	-3.824820115
C	4.422629469	1.285113452	-3.175612583
O	2.615937826	2.759558580	-2.780070253
C	4.394062423	3.724653838	-4.061088491
C	2.817578901	2.065258759	-5.091400979
O	4.302019535	1.574873948	-1.755188014
C	5.905848208	1.290604554	-3.536414160
C	3.819391977	-0.098864583	-3.411046107
H	6.405279304	0.452457353	-3.041510622
H	6.040118382	1.177717158	-4.617727788
H	6.393538123	2.212420791	-3.214513342
H	2.760394710	-0.105781189	-3.151784369
H	3.933182467	-0.412123012	-4.453428088
H	4.321565312	-0.826600563	-2.769438313
H	4.963132400	3.999543809	-3.168454347
H	5.091158694	3.608599574	-4.896771124
H	3.704971875	4.542463204	-4.288993948
H	2.255388977	2.930543435	-5.454556724
H	3.514304635	1.750787074	-5.876216252
H	2.104813484	1.263224901	-4.901994054

================================

^{1}8

================================

Mn	0.867480576	0.603195944	0.792513226
N	0.615442958	0.005531822	-1.137586053
C	-0.177093463	0.830277706	-1.986767019
C	-0.943943466	1.857302238	-1.390288749
C	-1.764249960	2.647572377	-2.222979408
C	-1.790347125	2.466659818	-3.598103327
C	-0.977574879	1.489857075	-4.192955750
C	-0.179537033	0.687732916	-3.390442453
H	0.486458629	-0.026601588	-3.862163285
H	-0.947754889	1.376061358	-5.272562802
H	-2.422765003	3.100326799	-4.214347150
H	-2.376827324	3.415801125	-1.761886929
S	-0.850004704	2.129909528	0.329264883
B	1.980231779	4.041919165	-0.110384887
O	1.555173056	4.835820883	-1.141475837
C	0.851776055	5.971394033	-0.580957358
C	1.297833793	5.953455852	0.941393813
O	1.739060857	4.586294484	1.136803605
C	0.176683920	6.246704345	1.936806576
H	-0.652394625	5.546039126	1.827552639
H	0.565018321	6.164857063	2.957096100
H	-0.205191077	7.263966796	1.800598279
C	2.509063320	6.844949340	1.241628864
H	2.839025568	6.659192123	2.267974893
H	3.347014076	6.610227271	0.581866912
H	2.265092172	7.906636631	1.141666498
C	1.292940977	7.215241173	-1.352576754
H	0.833077236	8.116920813	-0.934227164
H	2.378083404	7.336325406	-1.336482215
H	0.978491011	7.123486217	-2.396119955

C -0.643764888 5.725183175 -0.789721435
H -0.962765470 4.820362355 -0.268987733
H -1.241051606 6.575490374 -0.445700662
H -0.825409871 5.575805642 -1.857117970
C 0.937412551 -1.169795450 -1.621260628
C 2.012350885 -2.070203558 -1.246922811
C 2.961596077 -1.912016944 -0.210388099
C 4.084468236 -2.745953490 -0.147750716
C 4.257301154 -3.795032063 -1.045581448
C 3.298846650 -4.007758740 -2.042696405
C 2.212423564 -3.152943783 -2.140343262
H 1.495891016 -3.292091911 -2.945800459
H 3.413233234 -4.822320777 -2.751466662
H 5.129438363 -4.437506021 -0.968651862
H 4.828403974 -2.569513483 0.623999098
S 2.798054437 -0.666640340 1.065605298
C 2.553810499 -1.829717120 2.474794119
H 2.416801018 -1.229992431 3.372776173
H 1.658465587 -2.425830568 2.297049839
H 3.431286098 -2.468030100 2.587186329
H 0.403008196 -1.508204739 -2.508384030
H 3.336746396 2.884383484 1.744989251
C 0.858053841 3.142133822 4.504117965
C 2.348617850 2.776140497 4.405643457
C 2.274280405 1.449651943 3.658436362
H 2.842078456 2.686992260 5.377751216
H 2.884807841 3.515892564 3.802452134
C 0.276561951 2.598006186 3.188698540
H 0.402756473 2.649567873 5.370182301
H 0.692429564 4.218388609 4.602071103
O 1.273428556 1.670650310 2.648860703
H -0.661137890 2.050816134 3.317513733
H 0.156461436 3.371020894 2.435749835
H 1.955407230 0.639566625 4.331442675
H 3.199201901 1.158271740 3.156986662
C -0.182739302 -0.647259245 1.413634530
O -0.891146216 -1.485836286 1.816105121
C 3.815919388 2.688040556 0.786964937
H 4.156796971 1.651343871 0.792953967
C 7.328153818 5.221745556 0.512928361
C 6.251166579 5.444755546 -0.344101007
H 6.292785465 6.249124499 -1.074314072
C 5.112437429 4.638044475 -0.272699382
C 7.257962047 4.181532265 1.443190278
H 8.214463378 5.847891560 0.456299038
H 8.092999911 3.993946894 2.113579480
C 6.121609706 3.376787908 1.508256452
H 4.285803369 4.828512710 -0.949872173
C 5.033047818 3.591990847 0.653587214
H 6.079324493 2.560744190 2.227766495
N 2.809786556 2.869790054 -0.272067532
B 3.200730218 2.376194063 -1.564674655
C 3.618530007 2.434603141 -3.783883009
C 4.470073870 1.286802301 -3.117443150
O 2.626707108 2.719743378 -2.763972397
C 4.419964456 3.727835020 -3.994709701
C 2.901821886 2.049455280 -5.073850163
O 4.312204809 1.568247885 -1.702028286
C 5.960697431 1.316247338 -3.447152229
C 3.893624902 -0.106474296 -3.370699781
H 6.465271955 0.486260944 -2.943419224
H 6.120898753 1.209835341 -4.525664461
H 6.424881964 2.245353123 -3.111886689
H 2.825991748 -0.122036255 -3.148242524
H 4.044313445 -0.422226478 -4.407488241
H 4.384582969 -0.824734259 -2.709621404
H 4.968391929 4.004766025 -3.090291183
H 5.134354046 3.630595179 -4.818116625
H 3.721376338 4.535871091 -4.228200755
H 2.329013843 2.906019894 -5.440661210
H 3.624968916 1.760694414 -5.844472857
H 2.202638203 1.228842976 -4.914770954

================================

11

================================

Mn -0.877034966 0.194203656 -0.881650217
C -1.900003173 1.217275471 -2.303603515
O -2.544797162 1.794021933 -3.071152613
N 1.131557387 -0.396731541 -0.709219026
C 1.971678038 0.635218815 -0.206666845
C 1.389155238 1.736773916 0.472475006
C 2.250111110 2.757360504 0.911828547
C 3.625133700 2.693778420 0.701645384
C 4.188046959 1.601500249 0.034660370
C 3.358808251 0.577853404 -0.414987772
H 3.772412363 -0.259860684 -0.970199311
H 5.257246534 1.555460910 -0.149246278
H 4.258568003 3.503912120 1.052834013
H 1.816352187 3.606139282 1.431777044
S -0.349385481 1.822209598 0.816738393
C 1.512904540 -1.636541103 -0.496680280
C 0.897480384 -2.852171114 -0.992785729
C -0.026285606 -2.989316069 -2.072215445
C -0.582032103 -4.243403079 -2.360457042
C -0.243562841 -5.377183451 -1.625363962
C 0.692007749 -5.267980328 -0.589259914
C 1.250424724 -4.035170554 -0.293964324
H 1.967432266 -3.955208745 0.519168945
H 0.982199533 -6.143649848 -0.016381720
H -0.685687677 -6.337309733 -1.873228194
H -1.275231676 -4.323582511 -3.192630722
S -0.597565540 -1.639152920 -3.089821381
C 0.941155042 -0.963890608 -3.818531428
H 0.616367600 -0.224206497 -4.554150532
H 1.542109786 -0.474847206 -3.052464984
H 1.496500572 -1.761361825 -4.316257376
H 2.354518032 -1.798143701 0.182689436
C -3.437265019 -2.901556725 1.218241407
C -3.345487200 -1.718215288 0.189794394
O -1.897164725 -1.406738060 0.244046632
C -3.707829356 -2.072433779 -1.245292558
C -4.064292179 -0.444369689 0.637997530
O -2.325709042 -2.628829059 2.125386486
C -3.155402255 -4.270971045 0.593133140
C -4.727398542 -2.932273796 2.030494491
B -1.421285582 -1.859967958 1.471922216
H -0.342837185 -1.593218717 1.872247619
H -3.083292212 -2.878708670 -1.631017023

H -4.757695641 -2.376983475 -1.302134948
H -3.568089829 -1.197293994 -1.885964861
H -3.045050276 -5.005643741 1.395008424
H -3.975374971 -4.588046418 -0.057662503
H -2.229380701 -4.260397020 0.012801537
H -3.772490927 0.377815225 -0.023279409
H -5.149557895 -0.563361215 0.582879577
H -3.785989595 -0.170513513 1.658970041
H -4.856817367 -2.019329699 2.613499963
H -5.592764455 -3.055429014 1.371042058
H -4.702478107 -3.777408469 2.723419415

==============================

12

==============================

Mn -0.989058914 0.030568623 -1.124116246
C -1.321926645 1.430045671 -2.514109051
O -1.538683500 2.207207095 -3.345786138
N 0.996613425 -0.628276115 -0.761983174
C 1.694036872 0.308486175 0.060263147
C 0.972072441 1.065680246 1.012642214
C 1.674738867 1.992442043 1.792984719
C 3.048069139 2.177228066 1.635956319
C 3.751152282 1.428426854 0.691035751
C 3.073968156 0.495306484 -0.090646979
H 3.601291913 -0.077146649 -0.848895840
H 4.818976485 1.573601010 0.555509532
H 3.566337236 2.907502930 2.250713910
H 1.126758195 2.568139307 2.532206693
S -0.797756030 0.894922877 1.230761483
C 1.466032152 -1.845762362 -0.771420785
C 0.914816471 -3.024337670 -1.424691823
C -0.054535536 -3.089011153 -2.462493189
C -0.573326627 -4.326813478 -2.858133889
C -0.143534662 -5.514539926 -2.270035474
C 0.849110680 -5.474300578 -1.287913329
C 1.369705328 -4.252039750 -0.888530037
H 2.125038122 -4.224897676 -0.107632819
H 1.211358319 -6.391136919 -0.832906747
H -0.563983612 -6.461936721 -2.593322211
H -1.315788724 -4.351084639 -3.649770569
S -0.721994977 -1.668370062 -3.315194521
C 0.773026622 -0.894290281 -4.037891725
H 0.415288248 -0.041938119 -4.619563737
H 1.430975033 -0.537461652 -3.245583374
H 1.281027643 -1.606157516 -4.691453557
H 2.321476225 -2.059145639 -0.125226273
C -3.214677269 -2.324880763 1.700840670
C -3.137569748 -1.899716648 0.193670859
O -1.713951639 -1.628556650 0.050347882
C -3.509722679 -2.977525236 -0.819335465
C -3.917549381 -0.608146054 -0.089190750
O -2.217646695 -1.498562099 2.315863960
C -2.808909182 -3.794112791 1.907339088
C -4.559796672 -2.050682181 2.370837912
B -1.175449690 -1.283570914 1.393678364
H -0.060833719 -1.666033057 1.612896309
H -2.838098698 -3.834686703 -0.749671946
H -4.539806437 -3.314335517 -0.663718467
H -3.435058781 -2.573882870 -1.833744089
H -2.665998138 -3.965671962 2.977741777
H -3.573452530 -4.486808714 1.541553085
H -1.865134999 -4.012001763 1.398817134
H -3.699376478 -0.255364488 -1.105877495
H -4.997210231 -0.770806085 -0.027926306
H -3.642066515 0.179559229 0.616456618
H -4.784901697 -0.983102186 2.382952279
H -5.369893045 -2.580760785 1.857849124
H -4.527068889 -2.397819870 3.407584056

==============================

12-TS

==============================

Mn -0.847402469 -0.063001645 -1.606553426
C -1.411997722 1.391740391 -2.736334152
O -1.781597096 2.274562072 -3.399572421
N 0.918249700 -0.709665830 -0.731884716
C 1.418804084 0.409079105 -0.008657039
C 1.062846611 0.744054238 1.323682490
C 1.685937820 1.863280145 1.912704835
C 2.575237271 2.664612745 1.209812546
C 2.871043536 2.372810021 -0.126787067
C 2.294352391 1.257695464 -0.716011423
H 2.521026733 0.988649892 -1.744227762
H 3.551536658 3.000402178 -0.694063741
H 3.021790348 3.526500848 1.697035166
H 1.429681501 2.116336467 2.938085276
S -0.170764589 -0.040865220 2.360682175
C 0.932180765 -1.904032496 -0.118775141
C 0.705794056 -3.200997008 -0.832071449
C 0.064074638 -3.407746890 -2.075168617
C -0.125151191 -4.711520590 -2.555448376
C 0.311274905 -5.820486740 -1.835112982
C 0.953605933 -5.629739223 -0.612006798
C 1.140903123 -4.339507524 -0.129334105
H 1.624459830 -4.195989891 0.833421578
H 1.303286793 -6.479448569 -0.033617366
H 0.149754861 -6.820026341 -2.227378894
H -0.626581873 -4.846017141 -3.509046833
S -0.595310969 -2.102730374 -3.135949883
C 0.947634753 -1.522414960 -3.946995837
H 0.659176293 -0.719332052 -4.628739784
H 1.622851992 -1.140599433 -3.179534713
H 1.401659772 -2.345187338 -4.502134442
H 1.634930528 -2.008477952 0.714635007
C -3.239499575 -2.263752989 1.211303797
C -3.264342707 -1.030692376 0.246612426
O -1.834624705 -0.784953744 0.074837173
C -3.878533544 -1.288983822 -1.125173660
C -3.858991648 0.227169393 0.884907874
O -2.110647913 -1.974413676 2.055949236
C -2.963460997 -3.581941169 0.471099915
C -4.481157985 -2.395636864 2.088806380
B -1.156345928 -1.270914913 1.299538332
H -0.192060631 -2.050264530 0.804062510
H -3.342458892 -2.073888976 -1.662553149
H -4.927284905 -1.586118455 -1.026344005
H -3.844725402 -0.376416041 -1.730814483
H -2.732111650 -4.353765619 1.209752419
H -3.828716972 -3.908699613 -0.114205969

H -2.104084025 -3.487287276 -0.197389147
H -3.625134624 1.088904395 0.252276573
H -4.946111449 0.149536233 0.979534583
H -3.424279766 0.401170471 1.871696443
H -4.596559580 -1.528495624 2.741077169
H -5.381768394 -2.500530200 1.473949091
H -4.392433322 -3.284654944 2.719394564

==============================

13

==============================

Mn -0.597989032 -0.469773098 -1.541360456
C -1.091371619 0.563404157 -3.090374164
O -1.430269235 1.204399238 -4.008271484
N 0.913858943 -1.015339320 -0.413570823
C 1.328177729 0.052779482 0.376730116
C 0.888368786 0.316231269 1.703578634
C 1.340620411 1.441707111 2.402293830
C 2.200828245 2.359255849 1.803528286
C 2.602193432 2.151633761 0.481218991
C 2.178795096 1.022373029 -0.208087060
H 2.505459379 0.832343729 -1.226592066
H 3.262893300 2.863670686 -0.007613231
H 2.536874740 3.231434866 2.356079577
H 0.986770913 1.608848434 3.415629939
S -0.286407679 -0.743235637 2.572150197
C 1.004478784 -2.381511408 0.098965367
C 1.470764342 -3.306820543 -1.012977837
C 0.632293935 -3.659261569 -2.087960784
C 1.093192871 -4.486841072 -3.117123173
C 2.404667889 -4.958678928 -3.099007656
C 3.250951693 -4.614053740 -2.047256724
C 2.777341367 -3.799894913 -1.018730102
H 3.437126559 -3.522283989 -0.201038629
H 4.274679189 -4.976332197 -2.026208968
H 2.755482215 -5.596699349 -3.905627736
H 0.440925549 -4.773663843 -3.934403428
S -1.061874262 -3.032118945 -2.092130442
C -1.508264464 -3.046906584 -3.865518825
H -2.416832258 -2.447515548 -3.948328787
H -0.714990381 -2.574921925 -4.447567437
H -1.705864793 -4.060406419 -4.220965065
H 1.715922623 -2.439997941 0.932719246
C -3.972058518 -1.274066953 1.139241469
C -3.481030761 -0.212163802 0.102966347
O -2.006775964 -0.333915755 0.249794981
C -3.872682200 -0.502264802 -1.338222832
C -3.826277930 1.229028860 0.477949522
O -2.943669211 -1.193599443 2.163921097
C -3.950111599 -2.703995337 0.590844178
C -5.318798532 -0.957339303 1.777526447
B -1.797181728 -0.737387873 1.577554788
H 0.052515725 -2.761145868 0.504140983
H -3.467036759 -1.454591749 -1.683740386
H -4.963169072 -0.542203438 -1.421844243
H -3.512518534 0.290009528 -1.998621040
H -4.084087208 -3.399184740 1.422350518
H -4.753589613 -2.869004860 -0.132572444
H -2.995449155 -2.929297606 0.109015756
H -3.252610357 1.906141957 -0.160146790
H -4.891675538 1.428406982 0.330502618
H -3.571567578 1.438066827 1.520254975
H -5.292477514 -0.008323374 2.314930545
H -6.103525897 -0.911505346 1.015427274
H -5.579353534 -1.744289993 2.490365902

==============================

14

==============================

Mn -1.097260732 -0.458628016 -1.525754678
C -0.958995905 0.407003361 -3.241848226
O -0.898807845 0.945827140 -4.271968092
N -0.226548270 -1.179411332 0.317198293
C 0.888456064 -0.274769968 0.481402869
C 0.767467887 0.663252854 1.529586896
C 1.786827596 1.599323611 1.732073015
C 2.907502974 1.604463587 0.900044737
C 3.016105769 0.690237782 -0.149407177
C 2.001984924 -0.246149153 -0.364877683
H 2.070908644 -0.947411002 -1.189249417
H 3.879021041 0.709245844 -0.808099834
H 3.692566210 2.336647858 1.065282341
H 1.695953640 2.323572256 2.536191785
S -0.714874084 0.534380206 2.475291243
C 0.073203114 -2.617227388 0.560489445
C 1.030849696 -3.229485764 -0.439320399
C 0.705021942 -3.415143858 -1.801195892
C 1.652100122 -3.935176135 -2.690404475
C 2.926197031 -4.281548444 -2.239609391
C 3.257911148 -4.118891128 -0.897204663
C 2.312300255 -3.596902884 -0.016718832
H 2.575120425 -3.439390133 1.024768979
H 4.247440661 -4.385268731 -0.538539488
H 3.650917510 -4.681133789 -2.943086534
H 1.409113242 -4.083573997 -3.735772679
S -0.954701706 -2.984541015 -2.357882061
C -0.780787805 -2.860304825 -4.171821810
H -1.719159091 -2.426714966 -4.521632088
H 0.040770933 -2.190048275 -4.431609653
H -0.643189464 -3.842864928 -4.627370730
H 0.481615538 -2.737266120 1.571904750
C -3.678563072 -1.566548654 1.088495899
C -3.710963176 -0.157261174 0.381500358
O -2.311891853 0.041193032 0.072402044
C -4.503948420 -0.103519536 -0.923165063
C -4.148504981 0.981692283 1.308697083
O -2.385170940 -1.611950035 1.705659660
C -3.777658895 -2.729494663 0.091470954
C -4.738679291 -1.737176498 2.176930940
B -1.494595322 -0.658093322 1.138915316
H -0.890396472 -3.133672367 0.549207043
H -4.100017367 -0.796823625 -1.667636185
H -5.554806747 -0.358633532 -0.752756128
H -4.460748357 0.906965748 -1.341892522
H -3.553074053 -3.661867969 0.616965359
H -4.775935556 -2.812999131 -0.350049375
H -3.051976856 -2.620523069 -0.716058086
H -3.934972795 1.935501498 0.819935881
H -5.220209782 0.927108992 1.520865781
H -3.599834654 0.961299191 2.251648503

H -4.595282583 -1.015144160 2.982035471
H -5.747687310 -1.617685478 1.766191333
H -4.660981617 -2.739958369 2.606908618

Frequencies

================================
Benzene
================================
414.09 414.12 620.41 620.45 693.76 717.37
864.13 864.13 975.64 975.68 1013.80 1015.93
1020.09 1067.75 1067.85 1189.33 1208.45 1208.57
1358.35 1378.11 1523.64 1523.80 1654.00 1654.01
3171.52 3181.53 3181.57 3198.10 3198.20 3209.20
================================
Benzonitrile
================================
148.48 167.06 391.82 410.78 465.11 562.93
572.21 637.02 703.78 774.07 778.23 860.92
942.85 982.86 1011.12 1015.62 1054.93 1113.23
1200.40 1212.02 1226.29 1336.89 1364.23 1486.12
1534.22 1631.88 1658.06 2347.81 3186.78 3197.78
3206.71 3216.91 3219.82
================================
CO
================================
2203.61
================================
Diborylamine
================================
20.69 27.27 29.34 31.16 53.56 58.12
88.06 111.03 114.57 120.82 157.32 193.16
203.16 225.46 228.66 239.92 243.28 252.89
284.03 292.76 294.82 305.45 322.52 323.83
326.82 329.44 337.19 337.83 366.63 370.49
379.70 379.93 403.03 410.86 419.19 440.60
454.90 473.52 494.49 511.32 525.71 528.04
568.64 585.60 587.16 631.05 639.90 657.98
661.54 677.02 690.36 710.49 713.57 751.06
807.83 840.85 863.02 867.86 869.12 869.95
893.66 913.98 939.65 940.71 946.27 947.25
955.08 976.02 977.81 983.67 991.07 997.23
1006.57 1013.56 1022.24 1022.34 1033.21 1033.54
1055.68 1078.51 1112.58 1146.79 1147.21 1151.94
1182.83 1192.28 1192.44 1193.88 1196.89 1215.89
1221.25 1249.86 1250.54 1270.26 1270.46 1293.90
1302.66 1308.20 1323.08 1341.53 1355.08 1364.08
1398.13 1414.82 1414.93 1416.97 1417.46 1427.30
1427.41 1436.76 1437.10 1461.26 1485.88 1486.17
1492.15 1493.05 1493.14 1496.51 1498.17 1499.59
1501.85 1502.11 1511.98 1517.36 1519.03 1520.64
1523.02 1523.64 1524.02 1539.43 1540.95 1541.66
1636.22 1662.53 3048.31 3048.39 3050.27 3050.45
3052.97 3053.24 3057.08 3057.14 3057.57 3099.95
3120.74 3121.04 3121.95 3122.29 3127.81 3127.85
3129.27 3129.96 3132.90 3134.48 3135.02 3135.35
3148.51 3150.16 3150.50 3151.59 3152.92 3164.41
3168.55 3183.25 3195.69
================================

HBpin
================================
95.08 231.19 246.71 285.05 292.10 296.30
321.41 332.14 361.37 372.42 398.72 449.58
512.93 523.12 583.09 667.74 748.51 806.37
867.91 905.22 937.33 946.66 964.64 977.77
1022.53 1031.00 1065.17 1144.70 1183.05 1190.97
1206.98 1244.25 1272.06 1301.97 1383.03 1415.48
1418.64 1428.49 1438.59 1484.91 1494.32 1497.09
1501.96 1518.11 1520.71 1522.99 1541.89 2714.12
3051.59 3054.41 3055.97 3060.55 3124.46 3125.12
3133.34 3133.99 3137.19 3137.85 3153.73 3154.06
================================
Off-cycle intermediate, 9
================================
20.06 24.65 36.95 42.78 48.98 53.92
68.73 73.43 89.66 100.65 109.48 115.05
131.14 143.16 155.70 158.12 182.00 185.67
212.15 234.90 237.39 244.69 258.69 271.01
292.53 296.19 301.96 302.48 308.03 320.91
333.04 341.54 356.75 371.94 380.94 395.21
405.45 412.35 432.31 439.61 445.51 448.96
473.25 490.26 514.41 521.35 534.93 560.56
580.77 591.31 593.27 651.08 669.91 678.90
691.18 699.47 707.00 739.91 751.27 764.41
780.94 795.58 842.70 854.18 869.14 872.85
880.50 938.30 941.39 944.33 947.40 954.74
971.33 983.92 986.93 990.91 991.85 999.81
1005.94 1024.63 1029.34 1050.25 1062.29 1065.63
1081.83 1091.07 1150.42 1156.76 1166.21 1181.52
1192.49 1196.42 1204.12 1206.26 1235.11 1244.82
1269.24 1274.62 1289.53 1295.09 1318.98 1329.95
1357.88 1365.76 1389.43 1400.94 1404.33 1417.15
1420.61 1429.85 1440.24 1469.75 1478.33 1484.55
1485.72 1489.75 1494.22 1496.51 1501.98 1508.05
1517.12 1518.81 1520.95 1522.21 1525.06 1543.42
1609.60 1630.31 1639.61 1651.14 2013.76 3051.76
3055.24 3056.32 3057.53 3060.14 3064.25 3113.62
3124.58 3126.43 3129.51 3133.70 3139.21 3151.87
3154.27 3160.29 3162.97 3166.29 3169.04 3177.73
3184.41 3193.56 3198.57 3206.21 3208.12 3219.17
================================
Off-cycle intermediate, 9'
================================
14.57 23.25 33.31 42.36 46.56 49.87
54.20 57.54 65.26 74.47 98.08 100.29
112.94 120.16 124.89 132.00 137.81 161.85
184.50 196.11 217.61 226.35 232.89 253.67
254.56 267.05 268.04 278.16 288.70 290.63
300.35 308.28 318.09 320.40 330.47 335.94
344.25 353.40 371.24 376.03 394.87 405.61
408.85 416.11 435.72 438.83 449.28 472.30
485.03 514.50 522.19 524.80 552.78 582.55
596.23 607.71 648.41 674.53 680.46 686.26
695.80 718.84 737.44 748.71 760.06 780.64
801.15 839.87 861.94 869.79 871.82 875.18
930.34 939.62 940.99 944.21 948.05 974.32
982.54 983.86 989.23 990.32 994.74 1013.86
1022.65 1033.20 1056.85 1064.74 1082.21 1087.39

1099.39 1147.14 1154.03 1165.28 1186.56 1192.39
1195.08 1206.28 1227.84 1236.83 1249.48 1270.64
1275.81 1289.92 1302.65 1322.28 1323.04 1355.77
1361.64 1368.64 1406.08 1414.72 1418.18 1428.67
1438.89 1466.64 1472.63 1477.15 1486.02 1488.74
1493.67 1495.39 1496.78 1499.99 1506.31 1511.06
1515.77 1519.66 1520.51 1523.68 1539.84 1609.42
1632.05 1638.79 1650.66 2038.88 2119.61 3035.05
3049.84 3051.13 3055.82 3059.40 3065.42 3107.67
3122.76 3124.51 3127.64 3128.57 3134.62 3137.01
3151.62 3162.57 3167.08 3167.51 3170.18 3177.31
3181.99 3190.51 3196.51 3204.67 3207.57 3225.10
================================
Mn–H species, 10
================================
19.83 31.10 33.98 39.81 50.08 52.43
59.32 72.45 98.99 103.22 114.62 116.68
120.27 131.86 144.37 159.41 168.89 179.03
197.18 209.87 235.64 238.58 247.85 254.34
280.74 283.84 300.03 301.26 317.88 321.60
326.25 337.27 342.42 351.02 357.08 367.97
370.72 393.38 419.00 429.04 450.15 455.29
460.28 480.77 498.62 521.73 528.00 529.31
553.24 575.76 580.51 604.77 620.68 644.17
659.71 672.02 673.39 684.22 706.35 714.17
744.48 750.73 768.40 802.27 815.62 831.02
853.72 858.11 865.04 875.54 913.85 944.08
945.66 949.32 953.94 970.66 976.26 982.09
985.12 1026.59 1037.18 1038.43 1046.99 1064.85
1080.97 1128.79 1133.83 1134.44 1151.07 1158.19
1169.96 1193.44 1195.15 1198.16 1205.60 1233.58
1242.72 1275.24 1279.20 1286.43 1298.15 1302.91
1320.32 1343.02 1376.65 1381.43 1411.50 1418.54
1423.89 1431.21 1435.84 1444.87 1448.89 1470.12
1485.96 1490.54 1495.31 1497.36 1502.76 1506.26
1510.19 1521.37 1522.48 1524.88 1529.97 1535.23
1546.88 1560.97 1601.53 1620.97 1636.59 2207.07
2548.00 2632.13 2692.75 3042.32 3054.05 3057.50
3061.91 3098.44 3125.35 3127.15 3130.49 3133.87
3136.43 3138.89 3151.03 3154.07 3168.07 3175.33
3184.38 3190.24 3190.61 3196.97 3208.76 3210.26
================================
THF
================================
50.37 263.18 583.02 673.58 852.73 891.26
911.25 922.43 931.06 977.19 1035.46 1112.85
1176.82 1190.73 1196.11 1262.02 1265.07 1321.31
1353.47 1375.72 1413.68 1500.75 1511.86 1537.67
1551.82 2987.21 2993.35 3051.90 3053.98 3067.95
3068.58 3113.89 3122.63
================================
1
================================
37.57 50.11 56.53 74.09 83.99 87.58
96.57 100.98 106.61 113.08 141.31 162.22
173.45 189.50 214.14 222.23 240.11 265.00
272.90 279.70 311.43 350.54 371.33 395.21
428.72 443.25 458.55 461.49 471.20 474.43
476.52 479.64 517.00 525.39 535.50 567.76
579.68 591.64 639.98 645.48 660.00 684.03
690.43 712.57 718.82 737.92 742.17 763.28
772.46 847.66 868.22 879.55 919.73 937.18
950.80 972.19 984.65 987.98 996.53 997.84
1062.31 1063.14 1082.76 1092.23 1154.36 1164.63
1195.15 1207.03 1207.80 1264.88 1289.57 1304.01
1337.12 1347.67 1369.59 1414.08 1473.94 1477.14
1479.85 1489.30 1492.91 1515.99 1602.25 1615.06
1629.50 1643.64 1666.18 2045.05 2067.10 2118.60
3065.78 3114.73 3165.79 3170.91 3178.03 3181.61
3184.22 3194.96 3201.87 3209.93 3214.19 3228.99
================================
2
================================
25.50 33.15 45.91 48.24 50.77 88.15
109.92 122.58 150.35 153.58 179.65 194.11
214.82 229.52 258.20 270.64 282.24 311.20
321.49 342.62 357.36 392.75 408.71 426.87
440.18 449.21 479.15 518.71 558.18 573.64
580.65 652.40 676.96 709.41 713.86 725.73
736.77 762.74 770.59 846.27 865.11 872.17
911.82 937.54 941.48 960.41 980.10 986.64
993.19 994.94 1057.12 1062.99 1079.54 1091.66
1152.27 1165.94 1197.68 1208.14 1210.38 1260.40
1291.02 1302.85 1327.89 1343.17 1367.56 1403.64
1471.87 1473.66 1481.51 1487.09 1492.54 1507.34
1579.80 1602.11 1618.84 1639.51 1644.71 2008.41
3057.48 3096.01 3155.91 3159.60 3179.69 3180.27
3187.83 3195.08 3202.99 3210.29 3214.21 3234.39
================================
^{3}2
================================
28.14 41.78 58.16 79.14 90.61 103.49
132.89 156.80 162.81 186.80 209.64 218.74
243.18 250.13 265.92 319.39 332.73 339.77
371.93 399.02 428.87 442.55 463.75 473.88
496.29 506.06 538.82 559.21 577.33 581.44
605.74 658.28 682.22 700.48 720.38 731.19
743.10 764.73 772.98 849.03 867.43 875.14
924.52 938.48 950.03 969.46 983.75 985.22
996.25 1002.99 1065.41 1066.72 1083.25 1093.37
1154.08 1169.77 1191.49 1202.50 1208.53 1253.15
1289.34 1301.77 1344.04 1357.40 1369.46 1434.17
1468.24 1474.12 1475.22 1490.29 1496.28 1515.52
1574.85 1610.62 1617.51 1635.28 1643.73 1989.49
3073.57 3128.60 3173.51 3178.77 3182.47 3186.65
3187.02 3195.34 3200.60 3208.78 3211.44 3223.32
================================
^{1}2
================================
31.36 47.32 57.10 72.03 85.18 101.24
128.05 149.52 185.00 190.62 205.96 221.07
239.98 262.24 267.80 317.19 333.62 349.51
377.56 400.66 426.02 443.93 457.26 472.22
497.11 515.34 530.60 551.72 566.11 579.98
608.23 656.15 678.02 698.04 717.86 731.54
739.88 764.42 772.37 847.25 865.77 873.52
919.18 935.01 944.32 960.50 976.66 985.55
988.29 993.86 1060.34 1064.46 1080.75 1090.44

1152.15 1167.17 1193.59 1205.95 1208.20 1260.11
1289.06 1302.88 1339.01 1354.19 1361.25 1424.78
1469.44 1473.28 1474.93 1485.95 1493.44 1514.33
1585.55 1606.58 1620.46 1638.83 1646.80 1993.94
3063.91 3127.08 3159.61 3174.71 3180.12 3180.84
3190.57 3194.04 3202.70 3210.17 3211.22 3220.89
================================

3

================================

26.09 29.82 42.62 51.86 59.20 66.26
79.56 89.64 92.30 106.80 112.85 118.70
129.71 139.61 140.93 147.71 158.17 170.19
185.86 203.99 211.08 245.07 271.34 289.79
302.09 316.30 323.88 333.22 340.56 358.11
366.43 412.91 446.60 465.28 480.31 522.09
558.75 569.80 585.85 588.36 649.21 677.14
690.00 691.16 706.54 735.30 744.72 764.86
774.24 842.13 845.38 869.13 879.90 883.09
901.83 906.89 923.61 925.16 931.35 944.86
963.50 979.58 981.84 986.48 992.01 998.71
1047.13 1055.84 1059.38 1072.92 1076.43 1081.41
1148.81 1160.00 1172.63 1194.11 1194.45 1200.29
1207.97 1218.27 1256.34 1259.60 1274.24 1285.89
1293.26 1327.28 1327.71 1348.69 1358.44 1363.93
1379.54 1397.26 1406.95 1463.50 1467.93 1468.95
1478.03 1490.93 1502.14 1502.77 1512.90 1532.62
1544.48 1556.62 1599.75 1605.35 1631.89 1636.83
2004.15 3019.52 3055.79 3063.99 3070.45 3083.00
3086.66 3104.59 3121.97 3133.89 3143.30 3169.14
3172.20 3174.70 3181.93 3185.81 3189.21 3197.69
3201.69 3205.93 3211.95
===============================

^{3}3

===============================

26.27 46.35 56.51 61.51 70.03 78.07
94.33 102.48 115.02 120.98 128.55 138.22
151.55 161.81 176.28 181.74 184.66 201.04
207.61 216.03 250.37 259.06 280.12 302.80
315.55 340.61 357.08 364.34 416.03 437.20
462.69 476.48 482.81 497.17 527.89 549.69
561.12 570.68 584.39 586.03 652.97 677.60
688.92 690.64 708.38 739.55 746.85 766.78
776.73 842.83 851.46 870.40 884.67 886.98
897.72 910.19 927.20 931.77 934.64 949.65
967.99 980.12 984.36 985.84 994.04 999.65
1048.91 1058.00 1058.48 1068.00 1079.15 1082.83
1147.54 1158.71 1172.78 1193.08 1195.04 1202.03
1207.87 1214.67 1257.85 1264.61 1272.78 1288.24
1294.47 1327.63 1331.51 1344.14 1358.97 1361.17
1383.11 1405.24 1408.51 1463.49 1470.69 1471.75
1473.06 1487.21 1501.58 1508.74 1511.90 1531.72
1544.01 1591.75 1602.45 1622.45 1640.34 1650.00
1966.88 3022.32 3060.12 3072.29 3073.12 3074.56
3076.07 3111.11 3126.03 3134.11 3154.11 3173.18
3175.44 3178.10 3179.20 3191.36 3192.63 3196.26
3203.67 3205.27 3213.52
===============================

^{1}3

===============================

32.52 47.97 61.31 64.36 72.08 86.01
97.31 104.89 121.56 125.72 135.21 151.22
163.75 176.60 186.30 192.47 200.66 214.34
218.70 257.49 266.42 278.22 278.42 304.50
335.19 347.02 364.82 399.58 426.85 459.09
476.24 486.97 503.22 529.16 540.79 572.19
575.85 586.11 588.60 627.76 656.37 677.62
689.79 691.70 712.89 740.10 747.36 766.38
776.72 841.54 847.85 870.02 883.66 886.93
900.03 902.45 926.80 928.26 932.83 944.13
962.95 979.59 984.26 985.33 992.07 997.78
1050.21 1058.54 1060.42 1071.39 1080.50 1083.06
1147.92 1158.14 1172.00 1192.42 1196.08 1201.03
1204.18 1218.35 1260.94 1264.00 1274.28 1286.48
1294.96 1327.78 1334.00 1345.12 1358.07 1358.51
1383.15 1405.62 1408.73 1464.42 1471.60 1473.28
1473.76 1488.11 1501.79 1510.70 1512.40 1531.34
1544.09 1589.50 1603.87 1620.94 1635.66 1643.57
1989.80 3011.96 3058.97 3070.23 3075.51 3077.16
3110.32 3124.73 3127.42 3134.79 3153.06 3174.51
3175.33 3175.62 3180.94 3186.59 3189.68 3197.26
3201.11 3206.22 3211.56
================================

4''

================================

12.79 15.80 18.32 30.58 38.90 43.75
45.91 54.33 61.37 64.31 69.44 80.87
86.18 89.30 94.15 102.25 109.29 114.53
131.83 137.32 143.46 155.12 163.16 173.00
179.49 192.30 207.75 212.47 234.48 244.17
252.53 259.76 286.76 319.42 330.16 357.97
380.02 395.71 409.64 413.93 421.10 425.10
449.05 463.38 475.53 476.52 528.18 557.52
567.18 569.01 575.96 601.82 617.92 619.86
636.09 649.47 673.78 687.94 701.14 709.76
710.77 714.22 716.23 744.23 745.97 765.29
774.95 780.77 813.79 849.24 859.46 866.19
870.06 870.46 886.44 917.40 938.70 943.16
951.88 966.35 974.37 985.98 986.43 993.39
993.65 998.69 1012.87 1014.72 1017.39 1020.41
1022.22 1041.02 1053.78 1060.88 1063.87 1067.00
1069.39 1078.36 1117.51 1148.68 1159.36 1192.64
1192.78 1195.25 1201.00 1207.06 1208.75 1210.74
1214.40 1230.31 1268.40 1279.30 1294.88 1322.97
1341.89 1343.20 1355.09 1358.15 1371.22 1380.71
1386.72 1449.86 1466.96 1468.37 1485.91 1486.68
1492.18 1497.06 1518.21 1521.78 1527.45 1531.48
1563.67 1605.24 1624.78 1625.15 1626.44 1642.28
1646.11 1651.44 2182.08 2304.97 3067.93 3110.61
3166.01 3167.91 3169.45 3177.71 3179.72 3180.23
3180.34 3182.06 3187.41 3187.72 3189.52 3192.51
3196.12 3198.60 3200.63 3206.48 3207.01 3209.49
3209.85 3217.19 3220.29
===============================

4-TS'''

===============================

-335.13 15.95 30.30 33.00 37.59 42.04
47.36 50.05 52.37 60.93 73.16 89.31
90.58 94.87 109.49 126.50 131.39 136.57

139.46 157.18 174.85 180.26 183.70 188.71
197.58 209.01 219.41 236.50 241.85 254.32
263.81 268.31 272.82 292.85 301.52 305.46
318.36 321.26 329.64 335.69 351.14 353.19
357.06 361.75 384.28 393.15 397.85 406.58
417.21 430.44 433.35 437.62 464.36 492.58
496.52 512.03 521.20 531.69 534.21 557.31
566.72 579.63 582.15 591.10 626.25 642.18
676.40 678.78 691.58 703.17 710.07 716.26
727.87 743.68 749.34 767.97 773.81 775.51
798.20 842.52 856.24 856.93 866.06 872.62
876.63 896.69 919.89 929.31 938.67 946.15
947.20 950.70 958.51 965.17 978.16 979.37
986.89 991.04 993.42 998.40 1001.68 1008.27
1010.34 1022.30 1033.29 1051.89 1058.84 1061.03
1077.75 1082.13 1084.37 1117.02 1144.52 1153.90
1157.94 1187.81 1192.13 1192.92 1197.11 1200.58
1202.96 1211.81 1218.29 1245.64 1246.17 1259.08
1266.73 1284.65 1293.78 1307.15 1315.31 1328.70
1336.05 1350.11 1353.10 1367.06 1412.89 1418.53
1427.35 1437.86 1451.38 1460.56 1469.43 1477.79
1485.88 1488.69 1491.05 1492.17 1493.76 1500.07
1501.24 1510.86 1513.52 1518.13 1522.55 1523.65
1539.19 1575.07 1609.99 1611.70 1633.17 1636.34
1642.51 1655.62 2047.42 2129.81 3048.71 3049.96
3053.93 3059.19 3066.27 3088.35 3115.17 3122.02
3128.78 3130.96 3138.54 3147.94 3151.81 3155.62
3165.20 3173.76 3175.80 3178.98 3187.48 3189.31
3193.59 3194.80 3196.94 3197.65 3206.55 3206.80
3209.95 3211.82 3214.05

================================

4-TS''

================================

-182.75 18.21 23.57 30.99 37.41 42.34
45.72 47.61 50.48 53.83 60.12 70.10
72.02 81.20 91.64 92.54 97.58 98.43
104.73 108.93 117.18 123.70 124.89 131.54
140.80 142.37 151.42 171.35 179.94 186.04
192.62 197.55 219.66 226.28 232.82 238.39
246.76 250.41 259.09 263.85 274.61 292.54
305.78 318.93 329.04 333.43 334.63 347.94
357.53 371.83 376.69 385.77 392.97 407.11
417.03 420.06 421.75 425.06 432.49 448.83
450.31 464.65 483.77 504.31 524.09 525.82
527.18 552.27 574.84 577.10 585.25 599.12
617.22 618.39 630.08 650.06 669.70 673.49
688.07 698.26 708.02 709.69 713.62 717.69
722.56 729.19 748.56 750.11 772.41 774.53
790.88 811.41 845.79 852.75 861.46 870.36
873.33 879.31 879.99 899.52 916.99 931.82
943.72 945.74 952.01 959.44 962.57 976.30
977.81 985.47 986.26 992.31 995.03 998.86
1000.51 1008.80 1009.76 1014.37 1016.79 1018.44
1020.75 1029.91 1040.35 1042.41 1054.87 1056.10
1061.08 1066.37 1068.33 1071.36 1084.86 1090.13
1120.47 1153.54 1160.08 1172.01 1184.66 1188.39
1196.59 1197.53 1199.17 1199.94 1203.33 1206.64
1212.37 1219.08 1220.46 1244.61 1257.47 1264.38
1278.20 1281.63 1304.89 1310.05 1327.97 1339.82
1347.97 1356.85 1359.50 1370.33 1372.59 1386.63
1411.66 1415.58 1425.21 1435.09 1451.32 1464.40
1473.23 1480.65 1484.40 1487.71 1492.85 1495.01
1498.65 1501.37 1509.11 1512.33 1516.66 1517.98
1518.66 1524.60 1526.74 1531.81 1540.15 1566.37
1609.77 1613.05 1625.04 1633.61 1640.67 1644.37
1655.44 1656.65 2100.36 2180.72 3046.00 3047.61
3050.36 3054.25 3068.25 3109.46 3113.09 3115.42
3121.74 3126.49 3134.94 3138.22 3147.11 3149.68
3167.08 3168.31 3174.75 3177.44 3180.42 3181.32
3183.84 3188.35 3188.95 3190.00 3192.34 3196.24
3200.05 3202.82 3206.18 3207.35 3211.21 3216.86
3221.34 3229.21 3239.40

================================

4-TS'

================================

-253.20 21.88 26.08 35.89 36.97 43.50
46.11 48.44 54.86 57.92 70.28 74.88
76.50 80.52 90.74 92.54 101.83 104.64
112.73 121.15 134.13 139.02 139.56 148.78
155.19 159.10 169.96 176.89 188.48 192.92
198.48 209.90 220.39 231.84 234.39 240.42
246.27 247.59 258.91 265.97 273.38 280.89
289.22 298.97 300.75 309.12 322.18 335.73
338.45 347.67 356.77 365.24 379.81 382.31
392.00 405.45 418.50 425.35 431.44 444.87
452.69 457.73 483.42 506.18 519.24 522.51
523.96 550.03 573.97 578.62 581.98 586.43
594.76 630.71 650.28 669.66 674.86 681.31
691.99 704.54 707.28 711.81 714.18 729.57
739.64 747.46 770.84 774.59 794.58 795.35
849.28 853.53 855.67 856.44 869.14 874.37
875.91 881.46 888.34 919.49 928.67 931.10
931.14 934.34 943.11 948.90 960.37 965.46
970.92 978.10 981.56 983.58 985.48 990.20
998.30 1008.34 1014.66 1017.95 1022.61 1029.86
1041.68 1047.59 1051.88 1055.23 1055.76 1073.07
1076.84 1082.86 1092.84 1118.05 1151.00 1160.40
1163.75 1176.74 1187.16 1196.61 1197.56 1197.99
1198.98 1200.17 1209.94 1214.14 1216.83 1222.74
1246.93 1264.30 1267.30 1272.13 1277.41 1281.99
1285.91 1303.07 1307.65 1326.14 1331.53 1332.21
1340.11 1362.29 1368.01 1372.51 1392.09 1392.85
1411.80 1414.21 1420.79 1424.74 1434.69 1451.23
1456.94 1474.17 1480.87 1487.29 1491.14 1492.26
1496.44 1500.22 1501.63 1502.06 1507.27 1509.19
1512.31 1518.42 1520.57 1524.30 1525.89 1531.13
1537.85 1540.90 1556.07 1570.35 1608.72 1623.87
1632.19 1639.61 1655.74 2065.10 2088.42 3023.06
3045.03 3047.75 3050.98 3052.37 3055.16 3068.57
3070.87 3081.91 3113.85 3116.27 3120.08 3122.24
3124.36 3127.41 3134.34 3137.10 3139.44 3145.64
3148.14 3150.14 3155.99 3159.21 3168.19 3175.46
3178.06 3182.95 3184.64 3189.17 3191.02 3193.68
3200.71 3201.47 3207.77 3209.73 3211.88 3226.19

================================

4-TS

================================

-200.09 19.96 26.30 27.36 34.73 38.12

42.54 47.53 49.20 50.73 56.10 65.16
72.42 77.45 92.49 95.92 103.59 107.95
108.99 121.60 128.70 129.67 133.17 141.49
144.03 156.23 168.33 171.65 176.75 183.23
185.50 193.43 195.33 207.23 220.49 232.78
233.55 243.47 250.90 254.92 262.43 274.65
291.18 298.64 306.24 317.43 328.52 331.32
336.81 347.20 356.11 369.88 376.89 384.49
390.01 405.73 418.89 423.46 432.52 446.78
450.43 463.58 482.44 505.47 519.75 524.67
527.09 551.59 574.52 578.06 583.34 587.31
598.61 630.57 649.04 669.48 672.77 685.04
686.00 704.60 709.40 710.93 715.12 727.66
743.94 749.66 771.09 774.24 792.30 806.98
849.83 852.17 853.60 856.26 868.78 873.61
877.47 884.33 893.78 915.53 929.81 932.07
933.44 943.25 943.36 949.61 959.99 964.66
972.29 977.99 982.66 987.27 987.93 989.64
1001.52 1010.14 1015.31 1016.21 1021.18 1030.02
1037.53 1049.88 1053.10 1055.56 1061.43 1065.57
1071.97 1082.99 1089.75 1119.60 1152.11 1158.90
1167.86 1174.61 1187.47 1193.84 1196.42 1197.12
1198.90 1200.31 1208.79 1218.14 1218.89 1219.57
1246.59 1262.70 1264.60 1275.03 1277.07 1279.63
1282.07 1304.00 1308.49 1327.52 1331.43 1337.18
1345.31 1358.34 1369.45 1370.01 1389.81 1391.14
1411.73 1413.82 1417.09 1424.48 1434.38 1448.40
1462.72 1472.29 1477.09 1484.65 1487.19 1491.69
1494.00 1496.40 1500.00 1501.39 1507.45 1509.57
1518.06 1518.47 1521.71 1523.89 1526.60 1537.46
1540.21 1541.31 1559.76 1591.00 1608.42 1622.60
1633.83 1645.36 1655.61 2086.34 2209.27 3029.77
3045.26 3047.82 3051.02 3055.00 3056.25 3067.82
3069.08 3080.58 3106.58 3113.75 3115.79 3119.85
3121.96 3128.08 3134.92 3135.26 3136.28 3143.32
3146.49 3148.12 3149.72 3165.58 3169.65 3173.29
3179.63 3179.77 3181.46 3187.99 3189.42 3190.87
3198.79 3201.75 3206.52 3210.92 3212.59 3221.92

==============================

^{3}4-TS

==============================

-368.08 23.95 33.72 34.77 36.36 39.93
42.57 55.31 59.82 63.85 76.57 83.84
105.80 112.14 117.45 127.22 134.27 146.23
152.48 168.88 176.62 177.92 198.58 211.66
218.12 226.60 240.12 255.09 264.39 266.88
274.82 280.73 283.78 294.25 303.97 309.45
313.47 324.34 331.14 339.17 348.60 354.61
365.81 376.42 381.20 387.74 393.58 406.46
417.75 426.09 437.53 441.88 456.69 462.54
485.42 510.21 518.81 534.55 538.54 556.94
581.53 581.90 585.04 619.39 629.07 648.89
665.91 674.91 681.24 686.70 705.71 720.97
723.41 724.85 748.08 748.39 768.57 772.75
783.71 793.01 853.57 854.55 863.86 868.33
871.28 873.54 921.64 936.84 940.54 943.09
947.89 950.88 957.39 976.27 977.18 979.53
986.50 989.71 999.63 1007.22 1011.50 1015.42
1022.02 1029.91 1042.97 1054.02 1060.86 1070.90
1075.84 1084.56 1114.20 1126.49 1146.45 1155.63
1159.68 1184.54 1193.26 1195.64 1197.27 1198.51
1209.56 1211.81 1235.29 1240.37 1257.32 1265.13
1275.53 1280.72 1294.83 1299.54 1327.34 1338.12
1348.00 1357.16 1366.33 1373.57 1411.77 1416.20
1424.92 1436.13 1453.73 1468.39 1473.51 1482.04
1484.26 1486.30 1490.30 1495.54 1499.09 1503.88
1508.03 1515.76 1520.40 1521.22 1524.08 1528.04
1544.67 1575.29 1611.84 1621.04 1621.49 1631.64
1653.49 1669.01 2079.05 2095.53 3048.14 3050.45
3054.38 3061.59 3071.03 3114.60 3118.61 3127.12
3129.63 3137.04 3143.30 3148.77 3149.98 3170.75
3175.85 3181.40 3184.00 3185.26 3185.71 3190.87
3191.71 3195.20 3196.36 3204.06 3208.71 3209.68
3213.58 3213.69 3216.21

==============================

^{1}4-TS

==============================

-209.33 25.63 31.31 39.86 49.20 58.84
61.44 66.56 74.57 84.85 89.93 95.95
111.31 113.83 126.73 128.09 143.87 158.24
173.90 179.16 182.95 185.38 191.08 203.65
229.74 244.73 251.94 257.28 262.05 281.56
288.46 298.61 304.17 309.73 328.22 346.50
347.92 356.34 362.61 369.38 373.33 383.14
399.67 404.94 415.83 419.69 423.41 440.82
450.47 463.35 476.46 496.84 509.60 519.45
524.53 533.62 540.31 553.84 560.96 568.78
574.07 582.77 589.42 606.94 630.23 651.95
666.24 675.93 686.87 697.47 710.02 717.10
737.99 744.44 754.35 769.86 778.02 778.99
845.93 847.58 852.75 867.42 867.67 874.75
879.77 916.94 921.53 938.67 942.71 946.58
956.40 964.51 966.26 974.55 975.79 981.31
987.71 989.20 995.77 999.97 1010.99 1018.26
1022.14 1028.25 1036.98 1053.40 1057.71 1063.22
1079.19 1083.86 1111.15 1131.40 1156.36 1160.92
1188.35 1190.07 1194.08 1199.10 1201.37 1202.78
1212.49 1222.52 1239.78 1264.30 1269.35 1276.16
1289.97 1293.81 1299.82 1314.20 1341.15 1349.09
1352.91 1363.86 1404.17 1413.81 1418.53 1419.18
1426.59 1439.03 1470.83 1471.91 1475.40 1483.81
1486.95 1490.12 1495.40 1499.51 1500.20 1505.01
1511.71 1513.49 1519.43 1522.50 1525.37 1541.53
1545.47 1572.72 1614.52 1619.67 1635.56 1638.59
1641.24 1654.30 1916.97 2005.96 3052.75 3058.22
3061.44 3065.55 3068.80 3119.21 3122.81 3129.48
3132.59 3136.62 3151.40 3156.45 3162.86 3164.55
3170.32 3171.93 3175.06 3180.02 3183.26 3187.50
3189.07 3190.35 3195.03 3200.09 3200.60 3205.01
3210.62 3212.25 3213.41

==============================

4

==============================

11.15 21.70 23.62 25.73 30.24 41.43
43.98 46.95 50.81 62.26 67.49 74.08
86.87 96.64 102.14 106.58 121.64 130.24
139.97 145.83 150.68 156.15 159.75 167.89
181.99 189.70 192.90 200.20 206.05 213.46

229.80 257.14 262.65 281.89 285.78 318.21
326.94 357.49 380.16 396.74 414.31 424.26
448.23 463.11 475.01 475.61 527.52 557.02
567.23 569.49 576.47 584.13 602.52 634.65
648.45 675.51 686.86 687.49 702.74 710.17
713.68 741.24 743.66 764.04 774.51 780.77
808.54 848.92 849.62 854.32 866.05 869.50
886.06 892.44 916.48 926.27 930.36 938.17
939.54 951.92 963.77 971.21 982.11 986.47
993.79 999.12 1012.98 1017.49 1040.97 1046.95
1053.46 1059.99 1065.32 1066.56 1078.04 1116.80
1147.49 1158.82 1171.20 1191.25 1192.62 1193.73
1201.00 1205.76 1214.20 1216.87 1231.15 1263.83
1268.97 1277.24 1279.05 1294.51 1322.00 1328.75
1340.87 1341.78 1354.20 1357.25 1369.79 1384.11
1390.31 1412.98 1448.39 1466.23 1468.45 1485.72
1488.96 1494.61 1496.41 1499.02 1510.89 1527.74
1531.36 1538.37 1541.96 1558.58 1603.00 1623.86
1625.12 1625.67 1651.01 2200.33 2312.02 3035.41
3058.98 3064.71 3065.89 3066.03 3107.48 3110.00
3121.51 3130.09 3141.62 3164.45 3165.76 3167.90
3176.07 3176.85 3178.34 3183.50 3185.79 3186.17
3193.81 3196.82 3198.31 3205.56 3207.06 3213.28

===============================

^{3}4

===============================

11.63 12.08 23.62 30.43 34.44 39.32
43.80 51.56 58.24 68.26 73.32 81.75
90.75 94.52 101.39 104.36 119.35 123.52
141.92 151.77 153.81 163.92 166.81 180.97
184.37 189.06 199.68 204.12 215.21 220.67
247.05 270.48 271.59 312.27 318.25 329.80
347.22 373.75 389.53 394.81 407.20 435.64
451.07 467.85 471.35 477.05 531.33 559.68
564.81 571.92 580.45 585.73 610.51 637.21
651.97 678.37 688.95 698.92 699.83 715.09
718.82 738.99 746.15 764.11 777.07 779.93
795.31 851.70 853.71 860.91 864.67 866.81
883.23 899.68 922.15 925.67 933.36 934.80
937.85 944.69 961.26 971.90 980.89 986.30
987.43 996.20 1012.94 1016.41 1041.87 1050.61
1051.87 1062.64 1064.21 1075.43 1078.84 1116.73
1148.63 1158.17 1171.19 1189.25 1193.27 1194.41
1198.59 1201.26 1204.19 1213.19 1229.48 1263.33
1270.84 1274.05 1280.31 1294.14 1328.63 1331.95
1339.37 1345.30 1351.33 1358.65 1365.55 1382.58
1386.07 1407.95 1450.73 1468.27 1471.78 1485.38
1486.17 1495.60 1496.54 1500.38 1509.78 1530.98
1531.39 1543.21 1545.94 1560.53 1602.49 1615.75
1623.82 1628.70 1655.44 2170.50 2332.82 3058.21
3059.56 3064.42 3067.95 3073.25 3117.68 3122.54
3131.61 3134.59 3137.64 3166.18 3169.49 3176.04
3179.14 3181.29 3186.62 3186.79 3187.22 3191.14
3198.51 3199.99 3207.44 3208.78 3214.76 3255.47

===============================

^{1}4

===============================

19.77 29.81 30.76 34.54 41.10 47.23
62.51 66.47 70.10 79.47 90.91 100.02
104.25 111.06 114.75 121.76 138.24 156.74
164.25 173.24 182.62 199.14 205.83 222.90
237.11 252.05 256.60 270.63 277.16 286.16
324.93 329.19 338.04 373.90 392.19 406.27
410.31 417.73 435.04 467.89 477.81 482.87
496.77 507.32 537.99 541.57 551.75 569.12
577.62 585.25 587.04 605.85 610.23 635.08
657.75 676.53 690.93 701.52 722.72 738.07
743.96 750.10 765.14 771.79 774.12 790.71
848.79 860.89 864.74 872.35 878.04 890.86
919.45 921.69 922.79 933.45 941.11 944.27
960.04 973.19 975.78 981.12 982.17 983.74
987.45 993.24 1010.17 1013.27 1052.40 1054.07
1060.57 1063.65 1083.10 1087.70 1091.98 1115.43
1148.77 1159.19 1175.33 1190.46 1195.07 1199.04
1199.79 1202.48 1204.90 1214.45 1236.42 1258.67
1259.95 1269.61 1283.22 1295.05 1326.13 1335.19
1336.85 1350.21 1351.08 1356.56 1366.60 1380.20
1408.04 1419.14 1466.58 1470.76 1472.30 1482.97
1484.59 1484.86 1500.01 1510.45 1511.27 1532.07
1536.50 1547.47 1572.38 1595.92 1614.98 1625.12
1631.39 1641.73 1652.54 2008.47 2312.22 3041.47
3054.05 3056.42 3061.11 3066.41 3102.01 3115.88
3122.16 3123.30 3131.81 3168.61 3169.89 3172.12
3174.98 3179.44 3186.38 3187.91 3193.80 3197.06
3197.92 3203.31 3205.81 3209.95 3213.67 3220.92

===============================

4-TSa

===============================

-221.21 14.17 18.39 26.79 31.64 35.36
38.65 42.95 46.47 49.13 53.21 61.69
68.29 70.20 76.15 80.55 88.34 91.77
93.05 96.91 101.82 104.49 108.07 111.05
118.20 126.27 129.08 133.84 138.30 141.25
143.99 148.20 150.19 165.78 174.88 180.28
187.32 188.60 197.76 204.34 219.66 223.91
236.88 239.30 248.60 257.49 261.28 273.53
278.28 281.32 288.74 294.40 303.54 308.25
318.55 322.87 335.44 345.28 367.33 373.31
376.66 382.05 387.39 405.22 419.93 421.14
434.60 445.01 448.60 463.08 484.88 506.86
520.28 525.92 535.58 557.51 575.78 578.74
584.90 584.98 589.14 612.41 626.68 641.03
673.00 674.03 684.21 693.28 695.89 700.65
708.35 713.10 721.34 729.66 741.40 747.21
765.20 771.02 785.05 792.16 851.66 854.16
856.09 856.84 863.11 866.33 866.87 875.85
883.57 885.16 896.24 896.31 899.61 924.39
925.92 930.94 933.46 934.70 943.41 944.85
945.68 947.79 964.44 968.28 969.68 972.49
978.87 984.53 988.57 990.81 991.65 1006.23
1009.74 1015.91 1021.42 1030.61 1042.30 1047.84
1048.40 1051.95 1053.50 1069.75 1070.86 1072.55
1076.13 1085.28 1092.40 1118.42 1149.65 1154.31
1166.39 1175.74 1177.28 1187.66 1189.60 1195.38
1197.01 1197.36 1198.01 1200.81 1201.89 1203.20
1209.38 1219.09 1221.21 1246.07 1260.94 1264.20
1266.01 1267.70 1270.77 1271.61 1275.31 1284.92
1302.36 1304.65 1324.40 1327.85 1328.97 1335.45

1340.90 1358.15 1359.19 1361.44 1369.47 1379.82
1381.48 1384.14 1407.33 1409.49 1411.73 1414.07
1424.45 1434.38 1447.12 1469.07 1473.25 1475.62
1486.34 1487.42 1490.72 1492.87 1498.65 1500.76
1502.31 1505.61 1511.41 1512.50 1513.73 1518.03
1519.93 1521.06 1523.69 1525.05 1525.53 1530.85
1540.52 1542.30 1549.54 1563.74 1584.42 1604.46
1617.91 1635.03 1644.64 1655.85 2087.16 2193.64
3019.47 3044.69 3046.93 3049.59 3050.33 3051.66
3052.57 3054.09 3059.76 3065.05 3065.83 3066.03
3081.78 3081.85 3106.39 3110.46 3113.03 3115.01
3120.50 3120.69 3123.32 3127.88 3135.65 3136.75
3138.39 3144.40 3147.57 3147.64 3151.68 3152.44
3154.73 3158.26 3169.62 3176.94 3178.42 3183.55
3189.53 3191.43 3199.10 3200.18 3201.84 3202.71
3208.24 3217.49 3222.86
================================

4-TSb
================================
-369.02 21.87 25.00 26.75 31.02 35.81
41.40 44.37 47.28 53.39 60.04 66.20
71.53 75.41 78.56 93.47 96.89 111.71
115.35 120.61 124.47 133.78 137.62 146.21
152.64 159.08 173.70 179.97 187.71 189.98
203.95 210.18 215.66 218.00 223.79 236.68
247.64 253.06 264.03 270.99 274.64 279.90
283.03 296.98 305.76 314.94 321.87 331.16
341.71 344.72 368.14 368.91 386.83 395.17
397.03 411.91 413.83 425.32 429.10 438.53
452.57 467.49 497.06 500.95 519.44 525.15
549.09 559.35 565.66 581.03 585.22 587.95
592.15 630.93 634.13 673.38 676.24 694.78
696.84 703.18 704.81 708.56 731.50 733.63
744.21 756.71 765.43 777.21 794.48 804.98
841.83 853.88 861.39 863.39 866.11 868.12
873.16 883.40 884.01 887.70 924.90 930.00
933.65 936.70 939.86 946.18 946.90 948.46
966.79 968.90 972.55 973.90 978.37 980.94
981.80 982.54 1005.28 1009.32 1017.45 1023.72
1032.17 1048.70 1051.41 1054.96 1055.70 1060.09
1077.18 1087.08 1106.90 1117.98 1143.87 1152.80
1156.91 1170.67 1186.89 1187.95 1189.88 1194.66
1196.88 1198.44 1201.22 1212.35 1212.60 1221.37
1243.26 1251.16 1260.12 1261.91 1266.67 1272.97
1285.15 1291.81 1296.34 1314.46 1328.03 1334.06
1336.81 1356.42 1358.01 1366.70 1366.80 1381.13
1407.21 1411.93 1415.80 1425.21 1435.93 1446.93
1469.54 1478.60 1482.82 1485.03 1487.95 1492.40
1495.52 1496.99 1500.33 1501.22 1504.69 1507.13
1510.70 1517.36 1521.38 1522.29 1524.81 1530.87
1541.99 1546.02 1567.28 1593.91 1605.24 1625.11
1633.85 1642.05 1656.78 2033.96 2221.85 3044.32
3049.51 3051.73 3054.94 3056.16 3059.19 3060.27
3064.57 3075.30 3079.80 3119.51 3120.11 3122.37
3128.96 3129.56 3131.10 3131.45 3142.15 3146.13
3147.16 3148.39 3150.75 3152.07 3158.15 3162.42
3172.18 3177.93 3179.87 3189.11 3189.41 3195.72
3198.73 3199.56 3207.26 3208.36 3211.96 3225.32
================================

5-TS
================================
-116.34 20.60 24.33 29.43 37.52 39.84
47.30 51.68 55.09 64.85 71.07 74.20
78.25 83.96 91.17 94.88 96.52 106.64
113.69 119.47 125.40 132.33 146.20 148.46
154.14 159.62 165.02 168.78 174.45 187.45
190.05 196.03 210.70 223.71 240.12 242.49
255.27 263.81 265.79 276.26 296.10 302.36
306.66 311.28 313.60 317.94 322.80 329.17
334.48 338.34 358.41 364.81 374.73 382.95
394.66 407.78 420.52 421.91 442.32 456.20
466.81 475.42 480.96 514.59 524.28 528.17
530.08 551.59 571.07 574.17 586.74 591.39
595.97 629.17 642.47 658.33 668.27 674.51
685.03 686.19 705.37 708.01 714.57 741.93
748.59 761.94 764.62 798.28 805.28 830.13
832.08 841.22 848.64 854.09 866.62 870.15
872.80 884.85 900.01 911.03 923.71 926.68
939.04 939.54 942.21 943.03 948.23 970.33
972.04 974.19 977.10 977.95 981.22 989.99
990.84 999.92 1004.35 1013.16 1022.30 1032.28
1032.61 1049.64 1051.54 1051.57 1063.76 1065.76
1079.74 1105.18 1136.82 1151.88 1158.47 1167.94
1169.86 1187.87 1190.75 1193.50 1196.54 1197.63
1201.65 1206.81 1219.74 1221.83 1235.16 1245.15
1262.48 1270.66 1275.42 1283.25 1284.31 1300.14
1305.64 1324.61 1326.84 1326.91 1331.96 1345.63
1351.60 1360.82 1362.56 1379.98 1387.92 1407.21
1416.47 1416.62 1419.64 1429.19 1439.54 1448.86
1471.35 1473.68 1485.63 1490.27 1492.37 1493.45
1493.77 1495.81 1500.05 1501.86 1505.83 1507.80
1516.08 1519.49 1522.47 1525.39 1530.23 1539.10
1543.35 1544.91 1559.36 1605.39 1622.84 1634.15
1636.44 1652.35 1711.37 2116.43 2913.59 3022.05
3050.93 3053.26 3053.99 3057.09 3063.00 3066.44
3070.96 3081.25 3104.35 3119.74 3123.23 3126.73
3132.27 3133.76 3135.26 3140.99 3150.40 3151.99
3155.08 3160.09 3162.24 3163.91 3165.82 3171.91
3173.42 3173.70 3179.04 3182.99 3184.85 3185.92
3192.11 3196.67 3199.63 3203.36 3206.01 3215.80
================================

^{3}5-TS
================================
-263.58 27.20 35.48 37.83 47.89 49.62
55.10 61.25 65.00 74.13 76.25 81.66
89.00 92.90 97.85 108.32 111.20 118.46
124.41 134.17 147.64 150.77 155.42 166.33
181.22 185.20 190.82 198.55 207.80 228.27
233.71 240.51 241.74 264.67 265.33 271.55
275.04 280.70 287.87 298.71 304.41 310.17
319.84 333.49 335.00 339.96 352.33 356.18
375.84 380.85 393.69 400.69 401.58 416.02
424.12 427.05 443.73 455.95 464.73 472.03
476.70 514.16 520.21 525.24 531.53 533.85
545.16 552.87 575.14 582.03 586.63 592.27
606.55 628.13 648.13 669.18 674.77 677.11
691.17 709.08 710.45 716.79 741.89 748.67
761.64 762.15 770.56 784.37 804.44 844.61

852.29 855.21 856.87 867.41 869.62 872.64
878.95 886.97 918.82 926.01 928.76 934.08
935.88 940.36 942.79 947.47 973.04 973.27
974.80 982.66 984.74 986.23 986.91 989.81
991.06 1011.48 1014.35 1016.28 1021.10 1032.52
1038.42 1050.78 1054.50 1063.69 1070.81 1075.33
1077.62 1106.93 1120.55 1133.60 1153.63 1159.40
1177.27 1190.24 1191.12 1191.66 1197.04 1197.55
1198.46 1202.28 1206.11 1213.70 1228.55 1245.97
1258.97 1268.91 1275.60 1277.71 1283.02 1291.69
1304.52 1306.95 1328.17 1330.75 1331.33 1348.60
1351.59 1359.28 1365.48 1382.19 1384.35 1413.94
1415.27 1417.19 1426.34 1427.86 1437.00 1451.61
1475.95 1476.85 1485.89 1489.86 1492.49 1494.19
1494.95 1497.31 1499.76 1501.61 1507.51 1510.52
1519.37 1522.21 1522.82 1529.91 1530.61 1536.10
1544.18 1547.37 1559.39 1604.94 1618.30 1625.46
1635.63 1649.15 1671.27 2049.55 2958.46 3048.75
3048.82 3050.73 3054.28 3058.54 3062.19 3067.75
3076.62 3085.24 3112.92 3116.13 3120.20 3122.90
3129.17 3129.54 3134.99 3136.29 3144.08 3148.85
3149.29 3159.83 3167.30 3167.85 3170.50 3174.44
3174.85 3175.75 3181.49 3185.33 3189.48 3194.18
3195.88 3202.13 3202.95 3205.08 3208.55 3212.99

==============================

^{1}5-TS

==============================

-260.27 14.13 30.52 38.78 44.58 48.48
55.88 57.76 66.18 72.84 77.26 83.99
90.42 98.25 103.36 106.75 117.48 119.60
126.64 133.61 142.25 152.11 155.54 162.49
173.29 181.38 185.70 200.95 203.63 213.11
225.13 242.92 247.86 259.96 262.31 272.41
276.26 283.66 285.10 299.99 306.90 321.43
326.82 333.62 334.99 345.93 362.50 368.64
373.77 383.90 396.97 403.09 405.68 420.97
427.78 429.17 449.05 465.39 472.66 478.68
491.13 510.36 517.45 521.56 534.43 541.44
564.23 568.66 573.47 583.38 587.96 589.89
607.21 628.45 648.87 652.89 671.34 673.44
687.77 711.67 713.36 728.09 748.71 753.35
754.26 760.30 768.50 796.25 815.95 817.57
847.85 851.25 862.41 869.68 872.80 876.06
877.38 892.68 916.71 923.52 926.03 935.04
940.54 944.35 945.29 948.85 968.43 969.76
975.94 979.31 981.01 982.24 983.00 990.71
992.20 994.36 1009.99 1012.73 1022.41 1031.51
1045.26 1049.96 1055.37 1058.33 1071.70 1078.12
1085.25 1105.54 1137.20 1153.06 1157.03 1164.01
1175.83 1183.98 1188.89 1193.01 1193.44 1194.08
1196.53 1208.41 1209.32 1214.33 1216.89 1241.37
1253.13 1270.03 1274.84 1279.06 1281.32 1296.76
1301.26 1312.53 1320.97 1325.46 1327.76 1345.04
1355.31 1355.99 1360.49 1362.69 1379.37 1412.51
1414.64 1418.63 1421.31 1427.39 1438.57 1449.54
1468.28 1474.62 1478.04 1484.56 1487.51 1492.31
1494.50 1496.70 1498.42 1502.80 1508.14 1509.27
1514.74 1519.17 1521.54 1522.16 1528.13 1530.83
1543.76 1545.31 1582.99 1616.60 1623.06 1632.66
1636.91 1650.52 1723.73 2009.44 2857.44 3050.17
3051.04 3053.54 3055.93 3056.17 3060.02 3063.29
3081.20 3081.51 3113.37 3120.30 3122.50 3128.38
3130.86 3131.70 3132.19 3134.60 3137.27 3150.83
3151.61 3160.18 3164.63 3168.79 3172.20 3175.14
3178.75 3181.96 3183.70 3184.06 3190.94 3196.18
3197.83 3204.99 3207.68 3208.76 3214.22 3216.33

==============================

5

==============================

15.61 19.50 22.99 25.74 28.50 31.59
39.24 43.10 45.31 50.26 54.04 64.26
67.92 76.55 82.60 87.30 93.59 103.31
106.88 115.78 121.74 130.02 134.54 142.37
144.41 151.20 154.21 162.22 170.90 173.98
179.45 186.00 193.36 197.02 218.65 225.94
231.47 239.22 242.91 252.40 262.17 268.02
291.24 301.05 307.14 312.10 321.69 325.97
332.02 334.68 345.75 359.76 373.30 382.09
389.98 413.75 422.27 428.36 441.70 458.17
470.00 478.96 484.17 516.46 525.89 529.23
536.12 550.27 572.95 584.53 593.52 595.79
606.57 627.24 642.56 669.03 677.26 679.03
684.57 692.31 703.80 708.67 714.87 742.09
746.25 761.35 764.78 800.20 821.60 824.12
828.88 834.55 848.84 854.35 869.70 869.90
871.05 882.27 904.99 909.48 920.13 925.22
940.00 941.50 943.82 945.53 951.12 970.28
971.68 975.31 977.64 982.11 982.60 984.43
989.45 1002.34 1004.42 1007.04 1023.65 1032.13
1033.18 1046.81 1048.93 1054.47 1065.17 1065.49
1082.50 1103.52 1140.33 1151.90 1158.24 1167.55
1172.90 1186.24 1192.40 1194.49 1194.89 1199.70
1204.47 1206.32 1219.57 1239.16 1245.37 1251.29
1257.74 1275.54 1276.31 1285.63 1292.25 1302.70
1308.60 1326.40 1327.70 1330.31 1345.43 1345.78
1356.17 1363.37 1371.30 1378.24 1391.77 1413.24
1417.13 1419.10 1421.94 1432.36 1442.61 1448.08
1466.40 1474.00 1484.90 1485.22 1492.29 1493.03
1494.07 1494.94 1498.24 1501.80 1505.05 1508.95
1513.68 1517.63 1521.00 1525.71 1528.56 1537.83
1540.94 1544.94 1559.13 1602.86 1621.58 1635.02
1636.76 1652.87 1709.97 2200.92 2886.16 3020.20
3049.99 3052.69 3056.06 3059.02 3065.70 3067.21
3069.08 3081.22 3099.79 3121.46 3124.33 3127.30
3133.51 3135.34 3136.11 3142.68 3144.72 3148.48
3151.85 3159.60 3159.68 3164.40 3167.60 3169.62
3172.57 3174.76 3178.55 3179.09 3179.83 3187.38
3190.10 3194.94 3196.84 3202.88 3206.37 3223.25

==============================

^{3}5

==============================

26.61 31.38 36.86 39.95 46.32 49.06
52.93 59.91 62.37 63.75 70.87 75.47
76.96 81.06 93.32 98.92 104.86 112.43
115.93 122.97 132.68 144.55 151.84 158.76
165.91 172.46 174.45 187.29 187.46 198.75
225.40 233.70 237.20 246.59 253.68 261.70
268.34 276.80 286.68 298.60 306.81 313.91
329.87 330.97 332.66 339.22 340.93 349.43
363.58 373.30 379.93 382.95 394.31 417.46
420.30 429.75 432.83 447.48 462.11 469.18
472.06 484.30 518.98 526.20 528.83 533.32
553.54 560.42 579.46 584.10 590.21 598.74
606.79 629.67 644.24 672.99 677.72 688.53
691.52 704.22 710.65 712.10 720.88 746.09
747.90 767.21 768.92 799.61 821.84 829.61

838.42 843.35 850.75 860.08 865.87 872.03
873.69 883.49 898.40 914.07 920.18 925.11
939.37 941.91 946.65 948.01 950.84 971.82
972.24 974.20 975.21 978.29 978.32 982.96
984.10 995.09 999.73 1013.52 1023.73 1033.55
1038.96 1051.30 1052.52 1052.93 1063.05 1070.23
1085.45 1108.31 1141.91 1153.16 1159.03 1164.04
1173.23 1191.71 1193.14 1195.16 1199.99 1200.39
1202.23 1204.26 1233.04 1235.54 1244.76 1250.59
1258.38 1276.09 1277.30 1279.83 1288.89 1301.88
1307.81 1324.46 1325.04 1329.58 1341.32 1346.46
1354.78 1363.25 1368.32 1377.95 1387.85 1402.46
1408.39 1418.42 1421.55 1431.38 1441.78 1451.67
1470.37 1472.26 1484.52 1487.80 1490.89 1492.25
1492.69 1495.37 1497.18 1499.80 1500.98 1508.15
1511.36 1517.48 1521.07 1523.51 1523.80 1532.82
1537.34 1541.31 1569.11 1606.12 1622.07 1630.37
1636.74 1650.41 1689.76 2055.05 2977.05 3026.82
3051.80 3055.43 3056.02 3057.98 3067.33 3068.03
3068.64 3075.70 3115.31 3121.34 3126.59 3132.75
3132.82 3135.47 3137.01 3145.45 3150.94 3155.55
3160.56 3161.21 3164.42 3166.09 3166.56 3169.07
3171.48 3176.96 3177.75 3182.15 3182.43 3188.74
3195.58 3196.69 3202.68 3206.41 3206.86 3221.80

==============================

^{1}5

==============================

27.26 28.13 35.81 39.72 45.32 53.22
56.32 61.27 62.47 70.55 79.60 81.46
83.68 87.46 101.58 103.10 113.10 118.92
129.33 134.10 141.96 149.24 166.00 173.24
175.88 182.61 192.05 195.31 215.80 225.15
234.53 236.21 254.19 259.78 262.38 268.98
270.53 281.05 289.97 304.45 305.91 311.88
323.51 333.32 341.41 347.06 353.27 363.13
371.92 382.99 390.48 392.88 412.70 420.48
422.26 433.07 452.22 470.64 475.11 486.70
495.65 510.12 518.64 527.25 532.54 545.04
565.57 576.22 583.09 585.78 592.00 605.35
608.70 627.26 651.05 676.89 678.51 680.72
687.48 697.85 712.65 715.73 730.76 748.19
758.98 767.18 772.07 819.80 823.58 826.60
829.21 836.09 847.69 867.10 869.77 873.36
881.25 888.90 911.51 919.87 920.32 926.10
940.74 941.75 951.33 952.28 954.82 973.11
973.54 975.91 979.66 981.66 982.83 983.95
984.34 986.69 998.62 1007.58 1023.42 1034.02
1047.65 1050.42 1054.63 1062.10 1078.57 1082.01
1083.11 1105.27 1141.86 1155.61 1160.83 1163.03
1173.22 1189.44 1189.79 1195.52 1196.97 1199.94
1202.09 1203.62 1223.28 1232.47 1245.33 1250.44
1256.72 1259.61 1277.23 1284.00 1288.66 1294.78
1309.47 1320.22 1325.80 1329.19 1345.38 1346.81
1353.86 1359.47 1362.52 1371.90 1377.32 1406.61
1410.36 1418.79 1421.80 1432.14 1442.17 1450.76
1469.50 1470.28 1478.61 1483.01 1484.90 1486.17
1489.14 1492.62 1494.89 1498.73 1500.87 1510.49
1514.90 1517.73 1520.53 1520.95 1523.87 1531.03
1538.85 1540.83 1596.98 1621.60 1626.79 1631.75
1637.14 1652.24 1697.50 1991.11 2897.86 3009.11
3044.43 3051.98 3055.05 3058.78 3063.80 3065.61
3068.71 3075.67 3122.33 3126.09 3128.22 3132.81
3134.05 3135.98 3136.98 3144.04 3145.11 3148.76
3158.13 3158.69 3162.04 3167.07 3169.35 3171.25
3171.47 3177.95 3181.35 3183.41 3186.10 3192.43
3194.89 3198.45 3199.20 3209.15 3211.79 3223.92

==============================

6-TS

==============================

-205.01 12.23 22.30 27.63 30.08 38.72
39.29 42.07 45.93 48.26 55.39 57.74
63.64 70.11 71.79 74.50 82.29 83.20
91.44 99.01 105.99 108.60 113.24 122.24
125.26 127.63 131.25 136.35 140.70 155.39
160.12 162.37 168.59 169.32 179.06 186.06
198.73 201.82 205.93 218.35 224.61 228.65
231.07 237.65 242.90 244.11 249.32 253.64
266.01 270.96 284.49 287.77 299.39 301.23
303.56 310.51 324.24 326.80 330.31 336.45
338.94 340.27 347.49 362.14 367.77 373.15
377.52 387.43 392.83 404.63 410.66 417.43
420.10 425.95 441.75 451.46 464.89 478.40
489.50 498.69 519.22 522.23 524.96 525.64
551.94 556.49 577.90 581.64 585.37 586.21
604.11 609.14 628.52 641.53 648.17 655.46
669.20 674.38 681.53 682.05 683.32 706.48
709.81 711.92 717.96 732.27 741.85 747.88
766.05 771.61 784.83 815.13 850.03 852.14
853.11 857.57 860.48 863.49 869.31 875.18
877.52 879.00 882.49 891.08 913.69 925.85
929.06 931.17 936.18 937.47 937.82 941.89
943.13 948.81 959.37 971.60 974.59 977.62
978.18 979.04 979.45 979.98 985.38 992.68
998.90 1001.30 1014.75 1018.71 1022.83 1028.82
1036.72 1040.68 1048.02 1050.66 1054.36 1058.20
1069.22 1071.41 1082.99 1097.75 1114.86 1129.60
1143.50 1153.90 1160.40 1175.62 1184.88 1186.98
1191.59 1191.99 1192.94 1196.31 1196.51 1200.81
1210.83 1212.45 1216.65 1220.37 1222.94 1249.14
1250.38 1263.55 1270.21 1273.80 1274.90 1278.06
1278.87 1288.63 1302.39 1305.49 1306.72 1311.28
1329.85 1330.35 1334.72 1337.79 1346.74 1357.50
1366.74 1370.13 1390.94 1411.08 1412.29 1413.00
1414.63 1416.00 1419.30 1423.33 1429.01 1429.46
1433.42 1439.12 1446.86 1465.48 1471.60 1475.40
1486.07 1487.04 1491.69 1491.83 1492.81 1495.86
1496.63 1499.41 1499.69 1501.50 1504.09 1506.96
1510.04 1514.35 1517.11 1518.28 1519.37 1519.68
1522.34 1522.82 1527.91 1538.44 1539.34 1540.79
1546.65 1548.80 1561.49 1604.97 1626.21 1633.23
1639.43 1657.78 1911.81 2169.77 3018.52 3042.76
3046.06 3046.12 3047.86 3051.15 3051.89 3052.82
3055.02 3057.49 3065.00 3075.07 3080.67 3090.65
3098.61 3109.31 3115.01 3117.36 3117.51 3118.56
3124.03 3126.30 3126.50 3129.44 3130.35 3134.67
3135.45 3139.02 3142.95 3143.60 3145.66 3147.14
3150.91 3153.88 3159.99 3162.50 3164.51 3166.14
3169.52 3169.75 3171.52 3172.09 3179.74 3185.22
3189.39 3191.78 3199.29 3205.42 3209.94 3220.19

==============================

6-TS'

==============================

-565.40 15.20 19.86 26.74 37.00 41.20
41.56 46.48 54.05 58.36 61.71 65.85
68.96 73.40 80.95 88.86 101.37 104.34
115.41 117.63 124.72 130.97 140.65 144.20
144.51 155.01 160.82 179.32 184.02 191.38
199.28 205.56 214.37 219.07 230.09 230.38
235.87 239.60 248.28 255.26 257.58 261.18
263.95 280.49 298.34 300.84 303.19 305.39
317.67 323.26 329.67 330.85 339.60 340.97
343.20 348.50 374.97 377.86 379.76 382.59
395.84 399.00 406.60 419.94 423.08 438.06
452.08 466.17 478.24 483.28 495.34 501.10
507.41 520.89 524.31 529.05 555.16 557.78
568.62 578.02 585.57 591.69 599.96 618.35

629.76 656.15 661.42 675.10 677.98 678.28
679.76 687.99 696.31 705.14 712.25 734.87
739.28 741.30 762.77 773.27 776.01 822.91
834.11 843.30 854.36 860.41 862.31 868.86
871.88 874.49 878.18 890.09 923.99 926.04
937.28 942.26 947.39 947.73 949.33 954.36
954.52 966.35 973.06 974.82 977.33 979.72
985.23 994.06 996.72 1000.81 1001.13 1017.13
1021.41 1023.72 1032.69 1035.69 1047.21 1048.00
1061.46 1066.36 1084.82 1088.66 1117.64 1139.20
1140.56 1148.78 1160.10 1185.00 1187.19 1188.50
1190.28 1191.33 1192.03 1194.23 1195.00 1206.52
1223.13 1225.64 1231.05 1248.33 1248.63 1255.13
1266.83 1269.59 1281.02 1284.59 1292.83 1302.59
1309.33 1309.90 1327.85 1336.54 1348.04 1360.70
1367.04 1381.63 1413.99 1414.53 1416.56 1416.64
1427.09 1428.22 1437.03 1437.89 1448.27 1457.04
1466.84 1474.23 1479.27 1486.59 1487.86 1490.82
1493.29 1494.01 1496.08 1498.02 1498.89 1500.83
1502.37 1504.46 1510.63 1517.50 1517.72 1518.06
1519.66 1521.60 1524.78 1526.02 1529.98 1539.58
1542.27 1595.15 1600.91 1617.27 1626.97 1640.17
1645.18 1761.90 2214.60 3048.69 3050.34 3051.42
3051.46 3053.94 3054.78 3058.19 3059.22 3062.18
3067.70 3115.96 3121.49 3121.76 3122.28 3126.77
3127.44 3127.71 3131.48 3131.70 3134.08 3140.20
3143.77 3144.86 3149.29 3151.29 3158.59 3159.14
3159.32 3166.31 3168.14 3174.53 3175.43 3177.55
3179.39 3184.37 3189.03 3195.42 3198.49 3200.37
3205.92 3211.56 3222.29

==============================

6-TS"

==============================

-388.12 20.42 21.35 28.02 30.85 40.52
42.35 51.36 54.38 61.40 65.87 68.41
75.07 77.37 82.94 88.84 93.03 94.59
102.20 116.59 119.45 128.80 137.49 140.02
149.31 159.71 170.32 173.44 175.81 184.38
212.02 218.54 230.35 237.94 240.78 245.16
252.26 254.14 257.21 266.03 267.04 275.75
292.51 298.66 302.01 306.63 312.95 318.33
326.31 329.34 331.20 336.46 338.00 340.20
349.80 358.66 371.41 376.65 382.67 385.24
393.86 394.87 416.22 417.46 422.01 438.31
451.32 458.63 463.06 474.58 489.23 493.40
515.23 521.64 522.73 528.86 552.00 561.41
573.17 577.21 583.37 587.03 602.98 624.20
633.07 649.55 672.34 674.06 677.84 681.42
685.18 702.50 709.98 710.24 719.13 730.07
749.47 751.06 758.38 772.58 777.38 812.32
849.28 854.35 861.66 863.06 864.42 865.14
872.01 876.81 883.10 923.84 925.30 938.68
942.39 943.23 946.13 946.96 950.12 952.09
963.42 973.85 975.51 977.40 979.19 980.20
984.06 986.11 998.24 999.85 1003.86 1016.13
1019.53 1026.11 1027.76 1038.08 1045.15 1057.74
1059.32 1074.69 1077.65 1116.56 1129.02 1146.73
1150.15 1158.10 1163.31 1185.68 1186.60 1187.45
1190.71 1193.72 1196.20 1196.52 1205.72 1217.33
1219.99 1225.98 1244.49 1248.94 1258.81 1264.68
1273.04 1281.68 1293.58 1294.56 1295.39 1305.64
1317.22 1318.80 1335.37 1337.36 1353.43 1363.05
1365.37 1402.82 1412.85 1415.39 1418.18 1420.85
1426.61 1429.22 1438.13 1440.21 1449.85 1453.02
1457.87 1467.69 1474.57 1485.06 1485.65 1488.57
1491.88 1493.81 1494.49 1496.68 1497.89 1500.02
1501.56 1501.71 1502.05 1518.66 1518.76 1519.85
1520.59 1521.06 1524.46 1529.69 1537.79 1542.12
1542.16 1570.60 1602.48 1624.94 1629.88 1632.77
1660.01 1705.68 2083.84 3047.91 3050.50 3052.89
3053.30 3054.62 3057.61 3059.97 3061.91 3069.11
3086.08 3112.96 3120.58 3121.06 3121.87 3125.56
3129.75 3131.33 3133.07 3140.09 3140.36 3144.81
3146.13 3149.47 3153.22 3154.22 3154.45 3157.83
3165.45 3167.62 3174.52 3175.94 3176.37 3178.21
3184.14 3188.47 3189.25 3194.70 3197.91 3203.58
3206.97 3208.61 3209.72

==============================

6

==============================

17.57 22.99 27.50 31.88 35.55 36.97
46.47 53.79 55.25 56.55 65.05 67.22
74.91 77.25 80.14 89.90 98.90 104.48
107.40 115.75 117.17 125.72 133.93 135.82
150.14 156.43 157.61 172.46 180.17 190.54
195.23 199.95 203.96 223.69 228.30 240.17
252.91 259.26 274.53 278.67 286.09 299.58
301.78 317.36 323.13 329.74 333.29 340.08
341.31 354.52 358.21 374.40 377.72 385.77
398.01 419.67 423.51 433.72 456.67 467.44
485.61 489.34 501.64 522.14 529.55 558.20
564.43 567.60 583.02 586.06 586.75 596.59
630.69 652.18 654.04 678.31 679.39 685.53
689.95 694.78 714.13 716.77 733.59 738.22
748.19 768.00 771.08 778.70 828.12 830.50
845.03 859.90 860.94 867.57 869.64 878.18
884.31 890.59 892.39 918.48 921.76 923.10
931.49 937.02 941.01 947.47 954.95 966.06
972.71 979.22 979.93 983.63 993.41 997.08
1000.41 1005.77 1006.85 1021.58 1023.58 1035.42
1045.63 1046.01 1061.61 1064.76 1066.71 1081.37
1089.94 1116.14 1139.79 1152.78 1160.98 1173.19
1186.00 1189.03 1190.64 1195.02 1196.09 1196.22
1197.55 1200.19 1205.16 1226.10 1250.30 1254.35
1266.11 1267.99 1269.34 1287.98 1288.31 1295.74
1303.37 1311.29 1327.93 1331.65 1346.61 1350.52
1360.09 1366.86 1375.68 1382.45 1409.22 1412.30
1416.14 1427.83 1437.55 1446.20 1458.20 1468.15
1470.28 1479.55 1486.03 1489.96 1491.15 1493.64
1499.12 1499.51 1500.53 1502.26 1509.90 1513.93
1514.34 1518.12 1522.18 1523.29 1533.89 1539.18
1543.22 1544.87 1589.91 1595.28 1607.12 1624.39
1626.65 1639.63 1659.52 2001.97 3043.88 3047.49
3050.19 3051.90 3056.77 3059.15 3062.13 3065.99
3066.57 3069.38 3109.40 3119.18 3120.73 3121.68
3121.90 3124.40 3126.16 3128.04 3131.77 3135.71
3138.40 3144.60 3150.82 3163.71 3164.72 3172.35
3172.55 3174.26 3177.68 3178.82 3179.28 3191.42
3197.45 3198.35 3202.07 3205.43 3213.64 3230.80

==============================

^{3}6

==============================

16.04 20.87 25.85 30.43 34.03 39.21
42.01 48.92 55.05 60.67 61.86 64.46
75.22 79.71 86.99 88.66 92.70 98.34
106.72 113.82 120.53 126.59 128.92 132.72
145.60 150.73 161.78 163.67 173.71 181.58
191.59 197.25 209.33 216.06 229.65 242.43
243.24 254.99 271.01 272.93 284.15 300.86
302.33 303.64 315.07 317.09 331.58 336.30
351.52 354.18 365.51 373.38 392.09 396.75
404.51 415.50 431.81 435.05 447.97 462.68
475.59 484.96 491.06 521.43 531.39 558.96
562.53 568.80 579.21 582.83 583.62 606.26
632.61 649.99 650.79 672.06 674.99 683.25
688.53 700.39 712.53 717.83 720.12 736.80

743.82 764.20 771.80 801.42 839.02 851.63
853.07 854.71 858.80 863.39 866.95 867.81
882.32 889.68 914.21 923.13 925.13 932.76
934.84 936.60 939.76 944.94 949.49 961.06
970.31 976.56 981.87 983.06 992.71 995.62
996.63 1003.94 1012.83 1018.70 1026.90 1035.74
1041.66 1048.00 1059.39 1063.72 1064.71 1067.57
1079.54 1125.23 1148.59 1149.55 1158.15 1171.87
1178.53 1189.09 1192.51 1193.45 1195.56 1200.50
1202.16 1203.96 1217.80 1231.64 1241.87 1265.86
1273.55 1276.41 1277.33 1278.40 1291.82 1295.82
1329.17 1331.52 1335.60 1348.02 1354.25 1357.64
1359.49 1370.30 1379.38 1385.21 1389.89 1413.10
1417.14 1421.76 1432.52 1443.28 1449.56 1467.71
1471.09 1474.86 1478.73 1483.37 1493.78 1495.74
1497.31 1499.10 1499.24 1501.13 1509.32 1516.39
1517.23 1521.97 1523.06 1530.22 1540.12 1541.56
1542.32 1549.97 1557.13 1608.65 1620.00 1624.48
1635.06 1656.36 1739.27 2189.60 3028.50 3047.04
3054.93 3055.56 3055.81 3063.41 3065.39 3073.02
3073.74 3097.69 3116.76 3120.81 3122.36 3122.70
3125.83 3130.81 3132.51 3134.96 3135.03 3135.29
3143.94 3164.80 3168.09 3172.60 3174.32 3175.26
3179.36 3179.93 3183.91 3183.95 3185.58 3190.41
3191.67 3195.62 3199.54 3205.69 3207.36 3238.91

===============================

^{1}6

===============================

20.11 34.74 39.29 43.45 47.01 54.80
56.95 62.95 65.45 70.25 74.40 85.28
88.65 92.67 97.05 98.86 109.83 115.21
122.27 127.31 138.86 146.92 153.16 155.08
167.49 175.48 180.09 198.42 205.68 214.05
215.10 235.39 243.96 248.62 270.11 274.47
281.17 294.62 301.31 309.28 312.78 317.67
326.97 331.22 337.51 355.22 362.26 379.64
384.21 407.27 410.83 413.41 423.84 433.87
444.05 468.28 475.14 478.13 493.54 504.30
514.29 518.71 541.02 553.25 565.46 568.58
575.86 584.69 587.02 595.20 603.61 615.57
631.48 640.11 654.77 664.46 672.99 694.38
706.53 710.87 723.72 736.89 738.76 743.96
765.54 768.24 774.51 840.34 847.30 850.20
858.43 862.24 867.62 873.90 875.37 887.38
907.09 917.09 922.96 924.81 926.44 936.06
937.43 945.53 947.07 949.78 958.09 971.10
976.01 981.07 983.90 985.84 987.09 991.03
991.69 1000.06 1009.90 1015.69 1026.58 1035.20
1047.74 1057.10 1063.97 1066.70 1082.69 1086.07
1088.76 1117.90 1147.96 1152.42 1161.19 1167.66
1180.63 1189.74 1191.03 1196.95 1197.30 1199.07
1201.53 1217.59 1226.65 1239.78 1241.61 1253.16
1257.23 1274.48 1275.92 1281.24 1289.49 1293.61
1326.03 1326.87 1340.74 1348.66 1349.47 1354.47
1355.75 1368.34 1379.71 1385.25 1407.35 1415.28
1421.28 1421.68 1432.35 1443.57 1458.04 1466.05
1469.22 1480.26 1483.85 1487.83 1489.01 1491.87
1496.05 1498.54 1499.01 1504.11 1512.48 1513.68
1518.75 1519.12 1525.47 1535.33 1537.39 1545.63
1548.05 1562.74 1597.97 1615.06 1630.71 1631.46
1640.42 1655.86 1677.67 1982.75 3044.76 3052.18
3053.33 3054.32 3060.12 3061.39 3063.73 3068.02
3069.66 3105.50 3117.08 3119.89 3120.44 3123.37
3127.51 3130.80 3133.43 3136.10 3145.20 3155.29
3159.63 3170.13 3172.41 3172.86 3175.00 3175.30
3180.43 3181.67 3183.12 3187.24 3192.35 3193.56
3196.81 3197.54 3202.71 3206.01 3209.75 3213.77

===============================

7-TS

===============================

-97.30 14.83 25.91 27.92 35.31 36.56
38.63 44.19 51.76 54.80 58.59 63.43
71.74 74.06 80.10 82.58 85.00 88.43
93.60 98.88 100.70 105.46 109.37 111.74
114.12 122.03 123.02 128.34 132.58 142.77
149.17 157.88 165.89 171.63 179.11 184.24
195.76 200.53 212.24 229.04 232.61 241.68
245.97 250.17 257.99 274.14 275.99 281.94
283.39 289.57 306.27 308.21 311.90 313.59
318.12 326.64 327.28 332.29 333.89 337.80
342.59 345.39 353.93 362.77 365.33 378.58
382.02 387.48 392.67 395.54 410.57 427.89
429.16 441.23 446.86 465.57 468.03 482.12
491.36 502.46 514.48 516.50 520.91 523.60
542.75 551.68 564.39 579.24 582.31 587.16
590.19 594.91 595.93 633.44 645.59 647.81
667.55 673.92 678.46 680.92 689.19 712.79
714.36 715.42 732.05 744.31 745.44 751.66
762.26 794.00 812.51 818.12 834.63 842.92
851.55 856.70 858.63 870.21 871.64 873.15
874.38 882.59 896.78 912.64 914.65 925.67
927.45 934.90 939.03 939.90 941.14 942.73
944.67 944.76 969.93 974.06 975.18 975.48
978.59 980.00 981.76 983.42 989.86 998.84
1012.90 1015.18 1021.84 1025.71 1030.55 1033.94
1039.70 1040.74 1050.69 1054.19 1059.50 1063.41
1069.66 1084.03 1101.48 1124.29 1141.21 1143.14
1153.31 1159.38 1163.40 1164.50 1187.61 1188.25
1189.63 1191.06 1193.37 1201.67 1203.01 1207.17
1211.71 1214.09 1218.01 1233.21 1235.66 1247.94
1261.76 1266.62 1268.38 1271.44 1275.30 1276.14
1283.11 1291.71 1298.91 1299.34 1303.60 1325.13
1326.99 1329.21 1343.65 1350.13 1351.71 1354.81
1366.13 1379.94 1383.77 1401.85 1410.31 1412.39
1415.24 1415.79 1420.50 1422.01 1425.63 1430.04
1435.35 1441.58 1447.73 1471.67 1473.59 1484.60
1486.19 1487.07 1490.74 1492.21 1493.56 1494.10
1497.71 1498.40 1499.64 1500.11 1502.44 1503.84
1507.93 1511.18 1518.87 1520.34 1522.19 1522.99
1523.40 1525.59 1528.25 1539.37 1540.76 1541.58
1543.33 1547.77 1563.33 1606.42 1625.21 1639.87
1640.80 1661.37 2046.41 2939.13 3003.46 3030.13
3040.88 3045.29 3048.14 3049.13 3052.08 3052.39
3053.14 3056.04 3056.40 3061.45 3069.24 3075.60
3099.01 3113.40 3115.11 3116.33 3119.25 3121.21
3124.91 3125.56 3127.93 3129.25 3130.80 3134.33
3142.20 3146.38 3147.84 3151.76 3153.08 3154.51
3163.40 3166.20 3166.34 3166.69 3168.08 3174.97
3178.66 3179.22 3179.78 3180.88 3187.36 3192.43
3193.24 3194.50 3201.14 3207.60 3207.99 3220.45

===============================

7

===============================

15.13 22.14 27.54 34.01 35.05 35.67
40.38 44.83 48.07 55.08 56.11 61.40
64.58 68.98 71.43 73.15 77.18 81.85
84.85 87.41 95.33 97.60 101.86 106.85
114.23 117.99 121.82 126.24 127.58 135.88
145.08 151.96 157.96 165.10 169.52 181.44
193.47 200.95 210.18 213.40 216.91 230.56
236.77 237.88 248.50 255.05 258.86 262.95
268.18 277.52 293.02 298.94 300.13 308.87
311.15 315.89 320.01 322.74 328.05 331.31
333.53 334.73 341.26 358.98 368.77 372.46
376.33 388.39 392.50 397.39 418.52 420.00
424.05 433.11 443.68 465.78 471.95 473.21

479.18 492.74 517.67 520.44 525.03 527.75
544.77 549.76 574.21 582.45 585.44 596.66
597.39 598.80 609.47 627.64 647.55 670.22
673.52 676.21 679.64 685.86 690.34 696.32
708.88 710.29 711.21 740.49 741.46 748.28
762.87 795.19 800.23 814.95 825.12 833.51
849.75 853.35 853.68 864.30 867.21 869.19
875.69 881.44 906.77 908.79 915.32 921.45
925.05 939.08 939.73 940.48 942.48 944.39
946.05 951.09 968.92 969.29 974.94 975.83
976.50 978.74 980.26 981.66 984.06 991.74
995.98 999.33 1023.45 1024.18 1032.86 1033.86
1039.97 1041.78 1048.20 1049.00 1052.79 1065.46
1071.35 1083.63 1109.60 1136.19 1141.54 1142.21
1151.91 1159.99 1164.10 1171.71 1186.11 1191.13
1191.79 1192.53 1194.84 1202.34 1203.97 1207.08
1210.13 1215.48 1241.16 1243.04 1249.97 1251.66
1256.37 1267.23 1273.61 1277.38 1277.96 1278.66
1289.10 1297.27 1302.18 1304.05 1306.25 1326.08
1327.94 1332.56 1339.66 1346.69 1351.22 1363.72
1375.25 1382.03 1384.35 1407.78 1411.77 1413.90
1415.41 1418.66 1422.60 1425.31 1431.60 1435.12
1442.46 1447.98 1471.47 1474.10 1475.27 1484.86
1486.42 1490.17 1490.63 1492.27 1492.66 1494.74
1495.61 1498.60 1500.39 1500.69 1502.91 1505.13
1508.73 1510.15 1516.48 1518.32 1520.27 1520.78
1521.44 1523.66 1528.19 1533.58 1540.28 1540.57
1541.95 1549.63 1561.37 1599.60 1624.29 1634.41
1640.25 1659.43 2134.59 2966.00 2994.17 3009.55
3043.82 3046.31 3046.70 3048.58 3051.21 3053.69
3056.04 3057.76 3063.30 3067.79 3069.05 3083.42
3114.02 3117.17 3121.30 3122.84 3122.86 3123.74
3127.33 3127.79 3128.53 3131.22 3134.66 3138.93
3139.75 3144.91 3147.13 3147.28 3149.13 3149.23
3150.93 3154.41 3157.65 3157.82 3163.96 3170.42
3170.64 3171.98 3176.13 3178.49 3183.03 3185.26
3194.53 3198.47 3206.62 3224.10 3235.80 3238.81

================================

^{3}7

================================

-36.46 3.70 14.10 25.00 30.45 37.99
40.10 44.06 44.73 51.14 54.99 56.27
62.29 66.67 69.76 71.92 77.12 80.69
83.25 88.24 92.57 96.70 102.78 105.95
112.37 115.01 117.91 124.45 125.37 135.28
136.71 148.30 152.53 159.33 167.40 174.05
183.67 191.87 210.58 217.55 225.22 227.91
235.45 235.82 238.67 251.44 257.46 269.32
276.95 280.83 295.35 298.70 300.14 307.16
314.76 315.68 321.61 322.82 330.97 332.87
336.04 342.80 346.73 362.05 372.88 375.87
377.95 392.49 393.79 398.96 418.76 420.95
429.27 431.86 448.28 464.61 471.08 473.76
482.01 492.73 518.35 520.75 526.19 529.93
543.85 553.46 578.72 582.65 587.03 594.86
597.38 608.44 612.12 627.90 650.37 668.05
673.34 679.48 683.79 687.26 689.71 695.49
709.35 713.78 715.07 739.16 744.01 747.91
764.21 791.63 795.35 813.25 824.38 834.05
850.86 853.02 855.85 863.78 868.82 870.56
876.83 882.89 907.07 908.39 920.81 922.68
924.80 936.18 938.49 939.91 943.05 945.58
946.21 950.21 967.44 969.17 975.43 976.03
976.98 978.79 979.79 982.01 984.84 990.73
995.84 998.82 1023.55 1023.79 1032.28 1032.85
1039.53 1047.91 1048.27 1051.78 1055.40 1068.06
1073.69 1083.89 1105.92 1132.11 1139.04 1143.14
1154.12 1161.51 1163.65 1171.41 1189.58 1190.71
1191.54 1193.28 1195.03 1203.05 1204.37 1206.02
1209.49 1215.65 1239.04 1242.03 1250.63 1252.14
1255.63 1266.40 1268.21 1273.06 1278.32 1279.78
1285.99 1299.07 1301.54 1303.61 1306.48 1325.81
1330.42 1331.34 1342.70 1345.42 1348.34 1363.47
1374.80 1382.20 1385.05 1404.79 1408.63 1411.54
1414.70 1418.68 1422.80 1425.29 1431.64 1434.85
1442.59 1451.99 1455.42 1475.30 1476.33 1484.51
1486.82 1489.69 1491.06 1491.88 1493.25 1494.82
1495.67 1498.50 1499.66 1500.85 1500.99 1507.78
1508.95 1510.61 1516.55 1519.16 1520.26 1520.64
1521.62 1523.85 1526.45 1533.69 1539.53 1540.77
1541.21 1550.15 1566.63 1606.45 1624.06 1635.47
1640.33 1659.60 2188.42 2961.18 2996.68 3008.18
3042.97 3045.15 3045.84 3047.93 3051.21 3052.96
3056.22 3058.05 3067.83 3067.94 3068.83 3085.87
3112.48 3121.68 3121.80 3123.41 3123.52 3127.20
3127.51 3128.71 3130.93 3134.84 3134.99 3143.49
3144.94 3146.23 3147.08 3150.42 3150.75 3151.65
3154.53 3155.23 3156.37 3162.70 3163.67 3169.10
3173.30 3178.52 3178.81 3181.18 3182.21 3184.47
3193.89 3198.61 3205.48 3229.05 3235.82 3251.46

================================

^{1}7

================================

28.72 31.44 33.31 38.94 40.42 43.41
51.75 55.05 57.71 61.72 66.80 71.11
77.70 83.07 87.23 94.34 95.98 102.06
104.45 107.07 113.51 117.14 122.24 129.84
131.64 145.61 153.96 156.51 165.94 171.08
177.82 182.76 193.37 204.20 208.53 224.04
226.13 232.66 239.92 244.04 251.20 251.71
261.36 278.35 281.40 284.62 293.21 299.77
301.93 307.92 313.84 318.85 321.27 324.97
330.93 333.17 339.03 340.41 349.14 362.46
366.81 373.60 383.09 385.50 399.12 404.97
415.57 422.19 424.74 431.24 434.90 452.34
476.46 478.72 481.63 489.50 495.64 519.00
520.61 522.91 526.11 541.05 548.82 562.38
578.37 582.67 588.05 592.58 598.07 605.97
616.71 617.86 622.68 636.16 658.16 665.76
671.74 679.74 680.09 691.66 695.41 710.94
714.32 718.16 735.59 744.79 750.87 768.84
778.24 817.59 821.63 831.41 841.15 847.89
853.23 869.29 872.02 872.35 877.97 883.08
890.46 912.95 915.61 925.91 926.86 928.61
934.19 938.41 938.76 942.26 946.89 949.04
957.77 964.01 971.59 976.70 978.23 982.28
985.90 987.00 987.90 991.72 994.71 995.55
1005.00 1017.83 1020.20 1027.98 1031.21 1033.13
1043.90 1047.70 1056.11 1064.08 1069.38 1086.56
1087.24 1093.69 1101.63 1128.49 1131.38 1149.27
1161.56 1168.05 1168.30 1172.13 1187.58 1189.60
1194.40 1196.52 1198.75 1201.38 1204.02 1205.85
1211.97 1214.82 1228.69 1237.01 1248.77 1253.50
1255.99 1261.49 1263.74 1266.65 1275.19 1279.87
1295.37 1296.45 1298.15 1301.18 1312.28 1323.64
1327.01 1343.47 1347.17 1360.54 1362.56 1363.80
1381.42 1382.99 1395.33 1406.92 1408.61 1410.95
1417.55 1423.01 1423.04 1431.92 1432.12 1443.88
1450.14 1468.50 1477.90 1478.68 1480.54 1483.53
1486.28 1487.40 1491.42 1492.23 1494.56 1496.70
1497.25 1497.81 1500.92 1501.15 1510.21 1512.95
1516.86 1517.27 1518.12 1521.70 1522.02 1523.16
1523.44 1525.77 1531.85 1536.79 1540.49 1543.97
1545.51 1591.29 1620.33 1624.46 1637.17 1642.88
1650.52 1658.03 1958.95 2915.99 2990.55 3041.03
3043.96 3046.07 3051.03 3051.28 3052.51 3055.19

3058.30 3064.32 3068.08 3072.77 3077.93 3095.06
3109.14 3111.62 3113.29 3118.39 3119.71 3121.41
3122.75 3126.31 3126.73 3131.30 3131.73 3133.41
3134.95 3138.56 3145.46 3148.59 3150.58 3152.42
3155.20 3158.67 3166.62 3170.76 3174.25 3176.07
3177.04 3184.66 3185.92 3186.15 3192.89 3198.07
3201.73 3205.04 3209.87 3237.99 3245.43 3251.17

================================

8

================================

20.07 22.29 26.80 30.12 33.36 36.12
41.43 46.01 48.48 52.12 56.78 59.31
64.73 68.88 72.06 75.62 81.57 82.85
85.97 92.57 99.37 101.69 108.07 110.66
114.70 123.19 129.30 133.92 136.46 144.36
149.84 157.26 166.48 169.36 179.16 186.51
195.16 200.49 204.26 207.31 210.34 236.85
238.06 240.39 256.90 260.61 262.93 276.64
289.12 292.45 300.12 303.53 305.41 310.21
314.68 319.02 326.74 329.95 331.97 335.70
339.68 348.54 358.63 362.41 366.31 380.63
388.94 390.85 397.58 413.69 422.38 424.16
431.65 448.05 452.25 464.63 469.54 477.77
482.22 494.25 511.58 520.71 523.74 526.08
556.27 574.46 579.07 583.79 586.13 587.34
606.13 613.26 630.68 646.63 652.91 658.48
669.77 673.27 677.59 680.38 686.33 711.81
713.00 714.35 722.34 743.50 748.68 751.48
764.46 809.43 818.21 841.10 844.19 847.84
857.60 858.78 864.61 869.34 870.10 871.63
881.55 891.17 892.15 915.56 919.44 930.41
934.52 940.81 943.19 943.96 944.84 946.43
949.13 950.86 967.49 972.75 973.43 977.07
979.34 979.64 984.14 985.46 991.14 1000.17
1009.54 1011.31 1025.20 1027.44 1033.33 1035.68
1040.81 1046.16 1057.47 1057.77 1063.03 1067.75
1081.13 1081.66 1103.55 1129.31 1151.26 1152.29
1158.06 1159.46 1168.14 1178.99 1188.06 1189.01
1191.41 1194.08 1194.35 1196.71 1196.91 1206.35
1216.35 1221.78 1227.90 1238.57 1246.19 1258.94
1267.71 1273.56 1274.37 1274.75 1276.03 1281.23
1289.38 1295.72 1301.45 1325.78 1327.04 1327.20
1342.45 1350.64 1353.10 1359.82 1374.48 1375.96
1385.74 1402.36 1407.38 1414.46 1415.51 1416.67
1420.48 1420.62 1428.71 1430.02 1430.75 1439.46
1441.14 1442.29 1446.14 1467.32 1467.89 1482.77
1484.03 1487.31 1493.52 1493.85 1494.65 1495.81
1497.76 1498.51 1499.28 1501.32 1502.19 1503.22
1506.97 1512.31 1519.78 1520.49 1521.36 1522.90
1523.07 1524.07 1533.26 1543.62 1543.94 1545.22
1545.78 1550.03 1561.20 1606.62 1625.56 1629.51
1642.55 1665.29 2168.82 3009.39 3048.22 3049.46
3052.79 3052.80 3053.30 3056.14 3059.42 3060.18
3064.93 3068.75 3071.48 3087.76 3106.31 3111.37
3115.89 3117.74 3120.80 3121.61 3124.98 3127.19
3128.18 3130.63 3132.92 3134.62 3135.79 3137.68
3138.74 3146.70 3147.54 3149.51 3156.77 3160.37
3162.14 3166.46 3167.50 3169.40 3171.73 3175.32
3175.35 3178.05 3181.15 3181.20 3183.08 3185.53
3186.76 3191.81 3199.76 3201.24 3206.51 3207.57

================================

^{3}8

================================

14.12 22.27 25.62 28.24 32.08 37.90
39.28 42.16 44.21 49.50 58.57 60.78
65.20 66.09 71.52 77.28 84.25 88.40
92.84 99.49 109.36 114.37 115.73 120.42
126.31 128.34 132.36 135.92 153.73 158.62
166.21 167.77 171.36 178.58 190.92 192.81
199.91 204.88 209.74 213.80 230.98 237.39
239.56 251.90 254.16 257.58 277.57 281.40
289.69 300.96 301.82 303.96 312.37 316.27
321.35 326.13 327.76 331.46 335.66 337.93
347.43 354.33 354.84 366.75 377.38 385.10
389.13 392.80 400.97 408.19 418.04 424.75
444.17 447.08 458.25 460.99 465.31 476.29
481.05 493.37 511.74 524.02 525.21 528.37
559.22 572.22 584.59 584.80 586.00 587.66
617.04 618.78 631.25 653.05 656.39 664.32
671.96 675.42 685.84 687.71 688.52 712.02
716.87 719.97 723.83 742.44 747.50 750.90
762.75 806.12 814.94 840.64 851.92 853.51
857.01 861.32 866.48 869.72 870.92 871.21
883.57 890.26 893.66 919.61 922.73 935.62
936.63 939.44 940.09 941.96 942.19 947.91
949.10 952.93 966.10 973.33 977.48 978.23
980.27 983.88 985.08 987.03 992.86 1001.36
1010.60 1012.21 1024.07 1026.05 1034.77 1035.13
1046.54 1049.89 1054.74 1057.57 1069.13 1071.97
1080.43 1082.95 1115.29 1141.61 1150.60 1153.69
1156.79 1162.56 1169.40 1180.96 1190.78 1191.47
1193.09 1194.71 1195.43 1196.16 1197.65 1205.78
1212.38 1220.05 1223.76 1243.36 1247.71 1259.68
1271.20 1273.94 1274.47 1275.45 1277.40 1282.35
1293.02 1296.09 1301.39 1324.13 1330.31 1334.94
1347.56 1351.12 1355.09 1358.19 1364.33 1371.94
1388.12 1397.24 1399.82 1413.00 1415.88 1416.24
1419.12 1420.03 1428.70 1430.71 1438.78 1441.24
1444.01 1450.14 1458.59 1467.96 1472.28 1483.74
1485.01 1485.29 1493.55 1493.71 1494.61 1496.35
1497.55 1498.23 1501.51 1502.07 1504.67 1506.07
1507.69 1511.27 1520.36 1521.03 1521.37 1522.06
1522.52 1524.54 1531.07 1542.15 1542.74 1543.62
1546.79 1547.11 1566.35 1615.19 1625.46 1628.22
1642.19 1664.81 2157.46 3022.28 3047.86 3048.59
3050.89 3051.51 3053.63 3055.26 3057.95 3058.34
3063.04 3071.80 3075.44 3096.32 3100.00 3114.08
3117.93 3119.43 3120.19 3122.93 3127.96 3129.71
3131.09 3132.83 3134.67 3137.78 3141.11 3142.06
3146.90 3147.13 3147.65 3149.54 3151.34 3154.31
3156.98 3166.94 3167.30 3169.94 3172.64 3173.55
3173.58 3175.07 3183.52 3184.77 3186.79 3190.69
3194.23 3196.92 3198.68 3203.92 3206.23 3206.62

================================

^{1}8

================================

18.35 25.20 29.34 34.56 40.41 42.31
43.66 46.12 54.15 59.09 60.45 62.25
66.48 69.03 80.10 86.45 88.25 94.82
100.17 107.35 111.28 118.83 120.69 125.00
130.43 136.03 144.06 162.08 163.76 169.14
169.80 177.35 181.03 198.15 201.13 209.18
213.92 228.81 230.84 239.80 240.91 253.77
255.07 265.66 281.41 287.39 291.38 301.12
304.82 314.05 315.43 317.22 329.06 329.30
332.54 338.41 341.18 349.47 356.77 361.38

366.76 376.43 385.35 390.06 395.11 405.00
410.37 419.89 426.83 439.45 444.41 459.39
461.94 473.75 477.79 488.91 493.89 512.19
520.70 523.10 525.77 538.08 545.43 557.10
569.95 573.94 581.06 585.10 587.53 595.70
604.53 620.20 631.46 655.52 657.63 662.83
672.15 674.98 688.53 691.52 697.02 712.56
719.23 723.24 738.11 745.07 751.32 765.60
773.68 804.98 840.76 845.37 850.66 862.29
863.93 867.91 870.07 872.52 876.01 892.25
894.41 895.73 919.45 922.26 933.74 936.69
941.43 941.52 941.81 947.74 947.93 951.10
953.05 964.94 977.90 978.96 981.22 984.89
987.77 988.79 992.66 993.93 996.01 1002.30
1010.32 1013.69 1023.47 1026.04 1035.31 1035.75
1054.86 1057.56 1063.25 1069.03 1070.69 1080.74
1087.49 1091.55 1115.03 1143.15 1149.89 1154.38
1155.30 1166.73 1166.97 1181.68 1189.77 1191.27
1192.63 1195.04 1195.78 1198.92 1202.62 1205.70
1219.26 1221.21 1223.46 1242.82 1248.28 1253.26
1262.22 1273.08 1273.23 1276.62 1281.34 1285.28
1292.75 1295.06 1301.38 1323.34 1328.08 1347.33
1349.04 1350.04 1356.23 1365.04 1366.63 1371.64
1380.77 1393.37 1409.09 1414.78 1415.66 1417.84
1419.73 1427.45 1430.15 1431.56 1437.63 1440.97
1442.23 1462.25 1463.88 1470.58 1477.47 1483.99
1484.50 1484.69 1492.49 1493.12 1494.12 1494.87
1497.09 1497.54 1498.31 1501.51 1502.05 1508.95
1513.02 1517.30 1520.16 1520.30 1521.72 1522.26
1522.75 1525.13 1532.30 1542.38 1543.34 1544.75
1547.95 1566.63 1603.72 1614.08 1637.01 1641.48
1642.90 1664.82 1977.10 3003.99 3048.24 3049.45
3051.07 3052.08 3054.03 3056.49 3059.44 3060.76
3062.86 3069.63 3080.83 3089.06 3103.92 3113.38
3115.96 3118.94 3122.03 3122.43 3125.11 3128.45
3129.52 3130.72 3131.59 3132.96 3136.95 3138.32
3141.57 3147.22 3150.92 3151.04 3155.61 3156.34
3162.51 3165.45 3170.96 3172.25 3175.53 3177.74
3178.54 3182.47 3184.57 3190.06 3196.97 3197.32
3199.53 3202.82 3204.65 3204.87 3209.18 3211.10

==============================

11

==============================

22.48 27.53 35.69 40.92 44.42 48.15
61.99 67.27 80.45 90.71 96.66 106.62
120.49 124.70 131.17 139.72 149.02 171.91
193.13 202.92 219.30 237.87 251.28 261.17
271.08 283.48 289.63 293.76 298.97 310.04
317.41 321.94 328.28 332.06 340.76 367.85
369.42 377.55 380.97 400.63 419.80 457.06
459.08 464.73 480.44 515.92 525.08 532.74
560.97 573.29 586.37 595.36 646.73 668.42
671.03 695.59 709.12 731.82 744.70 745.51
763.76 769.76 793.02 845.65 862.48 865.79
874.81 887.69 915.72 921.33 939.68 941.77
953.12 963.46 966.44 977.73 979.12 981.41
988.95 998.45 1027.60 1035.47 1048.23 1052.44
1061.47 1078.31 1080.83 1121.66 1151.87 1161.28
1176.02 1189.74 1194.94 1197.38 1200.03 1206.99
1243.56 1255.81 1278.82 1285.16 1295.24 1307.84
1328.94 1350.93 1355.57 1371.65 1402.42 1420.35
1423.02 1433.19 1442.57 1459.36 1467.51 1468.72
1483.95 1486.29 1493.19 1493.30 1494.82 1497.01
1503.58 1516.60 1521.80 1525.37 1535.90 1543.15
1592.81 1603.59 1631.37 1633.78 1996.91 2772.77
3051.20 3056.59 3058.79 3062.58 3064.25 3091.67
3124.19 3128.93 3130.65 3137.51 3143.49 3147.34
3157.11 3159.44 3163.02 3173.63 3177.17 3181.22
3182.87 3187.54 3197.33 3197.93 3205.80 3210.80

==============================

12

==============================

23.77 30.58 33.29 44.95 49.16 54.21
58.78 67.90 89.26 102.00 115.22 119.07
128.76 141.48 154.54 162.79 172.63 178.78
193.75 206.97 214.43 233.78 246.10 251.18
270.34 293.55 296.56 309.08 312.35 324.99
332.79 333.02 340.31 352.80 359.19 367.43
382.24 386.90 391.21 413.80 423.66 462.79
465.14 480.60 492.35 521.10 529.60 555.70
565.56 576.55 587.78 592.05 651.44 672.25
675.20 696.65 703.89 714.44 735.20 747.80
769.41 773.38 824.35 845.22 856.53 874.22
879.84 912.23 918.87 934.98 939.89 944.12
951.63 962.63 967.67 978.46 983.80 987.22
995.13 1004.40 1008.00 1020.91 1030.67 1055.63
1063.82 1079.75 1085.96 1090.50 1122.57 1155.17
1162.89 1187.97 1190.85 1197.30 1202.10 1209.80
1231.77 1251.64 1258.25 1264.71 1290.62 1298.93
1305.27 1329.00 1351.71 1361.69 1412.54 1415.73
1426.09 1430.16 1436.09 1465.23 1470.93 1473.87
1485.32 1487.19 1491.79 1496.23 1498.78 1503.39
1505.68 1516.68 1521.23 1525.06 1540.81 1560.85
1607.58 1614.90 1636.61 1640.60 2002.99 2587.05
3026.64 3046.79 3051.00 3053.90 3066.28 3097.86
3102.64 3117.82 3119.72 3126.59 3133.39 3141.88
3151.59 3153.44 3163.47 3177.77 3180.39 3180.77
3187.52 3191.23 3200.07 3201.89 3209.76 3213.16

==============================

12-TS

==============================

-571.79 21.88 35.49 41.74 46.37 54.94
64.71 75.10 80.04 108.46 121.14 130.40
133.93 141.55 159.22 178.53 184.85 193.79
202.30 211.18 226.68 240.24 245.86 258.16
261.89 297.31 304.56 312.95 317.60 320.21
334.15 338.92 349.40 368.38 372.52 376.89
391.86 395.63 425.40 437.54 456.32 463.97
480.15 494.32 507.33 522.94 527.86 555.67
568.00 575.15 591.37 612.55 655.47 675.80
677.11 687.66 702.71 711.36 736.73 741.49
769.90 775.83 842.41 857.95 861.48 867.93
878.99 890.39 920.17 937.33 943.79 947.63
949.42 966.27 971.89 979.18 983.91 991.74
1000.65 1006.63 1020.93 1034.06 1054.35 1059.04
1070.47 1083.10 1090.78 1140.04 1149.89 1159.01
1185.90 1190.65 1191.05 1194.52 1205.50 1221.88
1234.07 1247.95 1254.82 1267.68 1281.45 1293.12

1308.50 1326.36 1335.14 1353.87 1383.58 1414.43
1417.29 1427.08 1436.82 1447.21 1465.39 1467.75
1487.27 1490.97 1494.08 1498.03 1498.68 1500.96
1502.57 1514.55 1517.66 1519.45 1523.66 1539.29
1600.62 1615.86 1641.56 1645.04 1745.30 2024.72
3046.33 3050.97 3053.81 3057.46 3060.74 3066.67
3112.90 3122.21 3126.83 3131.53 3142.97 3146.87
3147.55 3153.26 3165.10 3170.92 3175.68 3179.84
3180.81 3190.19 3193.32 3201.34 3207.70 3212.66
==============================
13
==============================
18.55 34.17 38.90 46.71 48.05 51.29
66.61 76.27 88.85 91.19 111.72 121.37
127.82 136.18 143.83 162.32 170.42 185.07
212.35 227.33 230.10 250.04 257.29 270.84
289.59 298.05 302.47 310.90 318.93 324.72
338.12 348.54 352.74 374.00 377.52 384.49
391.38 402.17 427.36 431.34 446.82 455.99
475.08 491.01 497.74 519.91 530.67 542.60
565.62 583.61 594.94 611.21 638.77 679.66
682.89 688.78 713.09 739.33 744.96 764.19
772.68 812.69 826.12 830.99 857.87 875.34
877.33 895.26 940.99 945.19 948.51 953.76
970.17 980.06 981.87 982.91 988.11 996.20
1000.41 1023.81 1036.20 1046.52 1050.62 1056.36
1080.81 1089.60 1119.71 1146.54 1163.30 1175.69
1190.95 1197.30 1203.81 1224.15 1224.91 1236.76
1248.04 1277.12 1277.70 1301.79 1314.05 1316.65
1326.48 1340.00 1352.73 1367.85 1381.86 1419.73
1421.22 1431.05 1441.01 1469.22 1469.30 1484.20
1487.29 1488.05 1494.63 1495.34 1501.02 1501.51
1509.53 1518.64 1519.53 1522.09 1525.96 1541.99
1597.33 1627.78 1634.29 1649.28 1997.53 2954.31
3042.80 3055.83 3058.22 3061.57 3061.99 3066.14
3129.12 3133.13 3137.86 3141.07 3141.70 3149.53
3156.81 3157.50 3157.78 3169.80 3170.75 3177.39
3184.46 3185.99 3193.90 3202.08 3204.91 3214.84
==============================
14
==============================
21.33 27.06 38.51 49.24 53.42 66.82
84.04 90.22 97.33 120.54 130.08 138.48
147.47 166.36 173.60 197.23 211.51 218.73
227.95 238.90 243.90 250.40 259.13 274.62
287.47 300.21 304.53 312.46 315.91 337.34
339.51 369.23 372.59 375.41 388.72 406.07
412.27 423.46 432.37 444.96 457.93 467.78
476.64 486.71 521.39 531.97 541.21 571.11
577.31 584.73 608.28 662.09 676.24 684.46
697.98 714.03 733.56 742.51 743.85 763.85
766.09 806.12 827.65 854.38 869.97 874.93
879.44 890.02 915.39 938.25 941.82 947.39
951.27 975.29 976.36 982.70 985.13 992.54
994.65 1010.91 1020.37 1031.07 1042.51 1059.27
1063.90 1088.63 1098.15 1129.60 1161.63 1166.03
1188.27 1191.85 1194.69 1205.73 1210.48 1227.32
1234.32 1243.37 1267.73 1275.54 1293.70 1296.88
1318.44 1343.80 1356.28 1368.80 1396.70 1412.16
1417.89 1426.02 1437.41 1470.35 1481.05 1483.39
1485.11 1489.03 1494.10 1496.17 1498.90 1501.52
1509.19 1516.90 1519.41 1521.92 1523.14 1540.82
1619.06 1625.77 1634.37 1648.34 2028.29 3039.46
3043.17 3047.89 3056.56 3062.30 3063.72 3109.12
3115.28 3117.72 3121.13 3127.16 3136.59 3152.23
3155.16 3158.06 3161.58 3169.41 3180.57 3185.85
3192.92 3195.84 3203.37 3209.65 3214.22 3224.51

VIII. References

(1) S. Stoll and A. Schweiger. EasySpin, a Comprehensive Software Package for Spectral Simulation and Analysis in EPR. *J. Magn. Reson.* 2006, **178**, 42-55.
(2) U. K. Das, S. L. Daifuku, T. E. Iannuzzi, S. I. Gorelsky, I. Korobkov, B. Gabidullin, M. L. Neidig and R. T. Baker. *Inorg. Chem.,* 2017, **56**, 13766-13776.
(3) T. T. Ngyuen, J. H. Kim, S. Kim, C. Oh, M. Flores, T. L. Groy, M. H. Baik and R. J. Trovitch. *Chem. Commun.* 2020, **56**, 3959-3962.
(4) R. K. Sahoo, M. Mahato, A. Jana and S. Nembenna. *J. Org. Chem.* 2020, **85**, 11200-11210.
(5) APEX 2, Bruker AXS Inc., Madison, Wisconsin, USA, 2012
(6) Sheldrick, G. M. SADABS, Program for empirical absorption correction of area detector data, University of Göttingen, Germany, 1996.
(7) G. M. Sheldrick *Acta Cryst.* 2015, **C71**, 3-8.
(8) P. G. Parr and W. Yang. *Density Functional Theory of Atoms and Molecules*, Oxford University Press: New York,1989, pp 1–325.
(9) A. D. Bochevarov, E. Harder, T. F. Hughes, J. R. Greenwood, D. A. Braden, D. M. Philipp, D. Rinaldo, M. D. *Int. J. Quantum Chem.* 2013, **113** (18), 2110–2142.
(10) J. C. Slater. *The Self-Consistent Field for Molecules and Solids: Quantum Theory of Molecules and Solids*; McGraw-Hill Book Company, 1974.
(11) S. H. Vosko, L. Wilk and M. Nusair. *Can. J. Phys.* 1980, **58**, 80-159.
(12) A. D. Becke. *Phys.Rev. A Gen. Phys.* 1988, **38** (6), 3098–3100.
(13) C. Lee, W. Yang, and R. G. Parr. *Phys. Rev. B Condens. Matter.* 1988, **37** (2), 785–789.
(14) A. D. Becke. *J. Chem. Phys*. 1993, **98**, 5648–5652.
(15) S. Grimme, J. Antony, S. Ehrlich and H. Krieg. *J. Chem. Phys.* 2010, **132** (15), 154104.
(16) T. H. Dunning. *J. Chem. Phys.* 1989, **90** (2), 1007–1023.
(17) B. Marten, K. Kim, C. Cortis, R. A. Friesner, R. B. Murphy, M. N. Ringnalda, D. Sitkoff and B. Honig. *J. Phys. Chem.* 1996, **100** (28), 11775–11788.
(18) M. Friedrichs, R. Zhou, S. R. Edinger and R. A. Friesner. *J. Phys. Chem. B.* 1999, **103** (16), 3057–3061.
(19) S. R. Edinger, C. Cortis, P. S. Shenkin, and R. A. Freisner. *J. Phys. Chem. B.* 1997, **101**, 1190-1197.
(20) A. A. Rashin and B. Honig. *J. Phys. Chem.* 1985, **89** (26), 5588-5593.Edinger, S. R.; Cortis, C.; Shenkin, P. S.; Friesner, R. A. Solvation Free Energies of Peptides: Comparison ofApproximate Continuum Solvation Models with Accurate Solution of the Poisson−Boltzmann Equation. *J. Phys. Chem. B*. **1997**, *101,* 1190–1197.
(21) Rashin, A. A.; Honig, B. Reevaluation of the Born Model of Ion Hydration. *J. Phys. Chem.* **1985**, *89* (26), 5588–5593.
